# Characterisation of the *N*‐Methyltransferase *Sg*PsmC: Application in the Kinetic Resolution of Pyrroloindolines

**DOI:** 10.1002/anie.202515459

**Published:** 2025-11-22

**Authors:** Benjamin Panagiotis Chapple, Lia Nitz, Pascal Schneider, Birgit Henßen, Sebastian Myllek, Mona Haase, Thomas Classen, Jörg Pietruszka

**Affiliations:** ^1^ Institute for Bioorganic Chemistry Heinrich‐Heine‐Universität Düsseldorf am Forschungszentrum Jülich Jülich 52426 Germany; ^2^ Institute of Bio‐ and Geosciences (IBG‐1: Bioorganic Chemistry) Forschungszentrum Jülich Jülich 52426 Germany

**Keywords:** Biocatalysis, Enzymes, Heterocycles, Kinetic resolution

## Abstract

Many natural products and pharmaceutical compounds bear the pyrroloindoline scaffold, highlighting the importance of the heterocyclic motif. Here, we aim at expanding the toolset for the selective synthesis of pyrroloindolines by characterising and employing the *N*‐methyltransferase *Sg*PsmC from *Streptomyces griseofuscus*, an enzyme involved in the biosynthesis of physostigmine, in a selective kinetic resolution of pyrroloindolines performed at a laboratory preparative scale.

## Graphical abstract

The N‐methyltransferase SgPsmC was characterised and applied in the kinetic resolution (KR) of easily obtainable pyrroloindolines. Substitution at 3a‐position proved to be a principal determinant for the enzyme's enantioselectivity. While moving toward laboratory scale, the reaction was coupled with a methionine adenosyl transferase for in situ generation of S‐adenosyl methionine, allowing the KR to proceed without any background reaction.

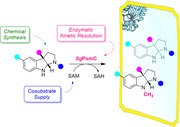

## Introduction

The necessity for the highest optical purity for increasingly complex synthetic targets for pharmaceutical compounds has created the need for new and tailored synthetic tools.^[^ ^1^ ^]^ With regard to the latter, enzymes have proved themselves a useful addition to the organic chemist's toolbox,^[^ ^2^ ^]^ mainly due to their high selectivity, their adaptability through rational engineering and directed evolution,^[^ ^3^ ^]^ as well as their potential for late‐stage modifications^[^ ^4^ ^]^ and for making chemical processes more environmentally benign.^[^ ^5^ ^]^

One enzyme class that has enjoyed an increased interest in the last years involves the *S*‐adenosyl methionine (SAM)‐dependent methyltransferases, whose primary function is the selective transfer of an activated methyl group from their cosubstrate SAM to a variety of acceptor substrates.^[^ ^6^ ^]^ It is not only their large substrate panel that makes them useful catalysts but also their promiscuity to catalyse transfers other than “simple” methylation.^[^ ^7^, ^8^, ^9^, ^10^, ^11^, ^12^, ^13^, ^14^ ^]^ Assuming that the respective (unnatural) SAM analogue can be provided, methyltransferases have the potential to be used as general‐purpose alkyl transferases.^[^ ^15^, ^16^ ^]^ The increased attention to methyltransferases is also due to the development of various cosubstrate (re)generation systems^[^ ^17^, ^18^, ^19^, ^20^ ^]^ that try to overcome the chemical instability^[^ ^21^ ^]^ and the difficult synthesis^[^ ^7^ ^]^ of the cosubstrate and its analogues. The most commonly used systems include a linear supply cascade employing a methionine adenosyl transferase (MAT) for the formation of SAM from adenosine triphosphate (ATP) and l‐methionine,^[^ ^10^, ^18^, ^22^ ^]^ and a true recycling system based on a halide methyltransferase (HMT) and a sacrificial methyl donor (such as CH_3_I or CH_3_OTs^[^ ^23^ ^]^) for the re‐methylation of *S*‐adenosyl homocysteine (SAH) to SAM.^[^ ^24^ ^]^

Besides producing SAM in situ and from relatively inexpensive starting materials, these systems can also be used to access SAM analogues and thus broaden the targetable chemical space.^[^ ^10^, ^25^ ^]^ Although still at an early stage, the application of methyltransferases has been strongly facilitated by the development and optimisation of said cosubstrate systems, making it possible to highlight their high chemo‐,^[^ ^20^, ^26^, ^27^, ^28^ ^]^ regio‐,^[^ ^10^, ^27^, ^29^, ^30^, ^31^, ^32^, ^33^ ^]^ and enantioselectivity.^[^ ^34^, ^35^, ^36^, ^37^, ^38^ ^]^

**Figure 1:**
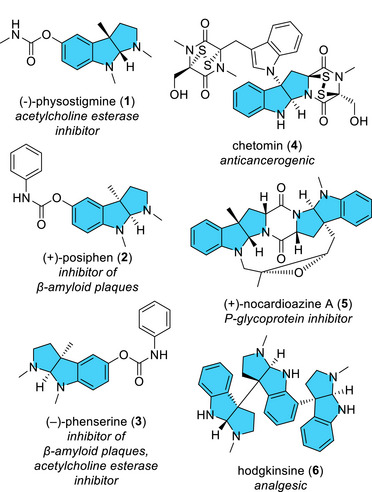
Examples of naturally (apart from compound **2**) occurring alkaloids bearing the pyrroloindoline scaffold (highlighted in blue).

Pyrroloindolines (for selected structures **1**–**6** see Figure 1) form an extensive and large group of naturally occurring alkaloids^[^ ^39^ ^]^ and exhibit various biological activities, ranging from analgetic and anticancerogenic to antimicrobial and anticholinergic properties,^[^ ^40^, ^41^, ^42^, ^43^, ^44^ ^]^ explaining the interest in their synthesis and derivatisation. Unsurprisingly, a number of chemical syntheses have been described – both symmetric,^[^ ^45^, ^46^ ^]^ and asymmetric^[^ ^47^, ^48^, ^49^ ^]^ – to access the tricyclic scaffold.

Recently, enantio‐ and diastereoselective access to the scaffold has been made possible also using chemo‐enzymatic routes employing *C*‐methyltransferases.^[^ ^34^, ^35^, ^36^ ^]^ These catalysts were used to form the (*S,S*)‐configured *C3*‐methylated scaffold in a highly selective manner and have been used successfully for the synthesis of physostigmine (**1**) derivatives in preparative lab scale. However, they do not allow the formation of the (*R,R*)‐enantiomer; access to both enantiomers is of great importance to take full advantage of the biological potential the pyrroloindoline motif has to offer. This is emphasised by the natural occurrence of biologically active pyrroloindolines of both configurations,^[^ ^40^, ^41^, ^42^, ^43^, ^44^ ^]^ and also by the enantiomeric pyrroloindoline pair (+)‐posiphen (**2**) and (–)‐phenserine (**3**). While both physostigmine derivatives have been shown to inhibit the translation of the amyloid‐precursor‐protein^[^ ^50^, ^51^ ^]^ and of α‐synuclein,^[^ ^52^ ^]^ (–)‐phenserine (**3**) additionally inhibits acetylcholine esterase, while the inhibitory effect of (+)‐posiphen (**2**) is much weaker.^[^ ^53^ ^]^ The different modes of action are conveyed by opposing configurations, which in this case would allow differently high dosages to be administered.

**Scheme 1:**
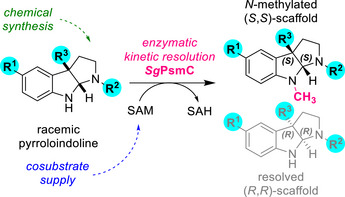
Schematic overview of this work. The *N*‐methyltransferase *Sg*PsmC can be used for the kinetic resolution (KR) of chemically accessible racemic pyrroloindolines, yielding the *N*‐methylated (*S,S*)‐scaffold and the resolved (*R,R*)‐scaffold.

In this work, we perform a systematic expression optimisation and biochemical characterisation of the *N*‐methyltransferase *Sg*PsmC and show the catalyst's benefit in accessing selected pyrroloindolines chemo‐enzymatically in an enantioselective fashion (Scheme 1). *Sg*PsmC is part of the physostigmine biosynthesis cluster of *Streptomyces griseofuscus*,^[^ ^54^ ^]^ acting directly after the enzyme‐catalysed and methylation‐induced formation of the pyrroloindoline motif by the *C*‐methyltransferase *Sg*PsmD. We take advantage of the high enantioselectivity of *Sg*PsmC for a methylation‐based kinetic resolution (KR) – a generally uncommon approach in chemical^[^ ^55^ ^]^ and underrepresented strategy in enzymatic KR^[^ ^37^ ^]^ – of easily accessible racemic pyrroloindolines. We show that *Sg*PsmC can be used as a complementary system to previous *Sg*PsmD‐based^[^ ^1^, ^34^, ^35^ ^]^ methods to also access the (*R,R*)‐configured scaffold, thus proposing its use as an additional tool for accessing the chemical space of this heterocyclic motif.

## Results and Discussion

### Biochemical Characterisation

#### Enzymatic Profiling

For biochemical characterisation, purified catalyst and the model substrate 5‐methylindoline (**7**) were used. *Sg*PsmC was expressed in *Escherichia coli* Tuner(DE3) under optimised expression conditions. These were obtained by a design‐of‐experiment guided approach using a three‐factor 5‐level central‐composite‐design which indicated low growth temperatures and low inducer (IPTG) concentrations to obtain highest enzyme activity. The optical density (OD_600_) at induction had minimal effect, allowing for a straightforward cultivation protocol at 22 °C, 104 µm IPTG and variable OD_600_ at induction (for details, see Figure S1a,d).

**Figure 2:**
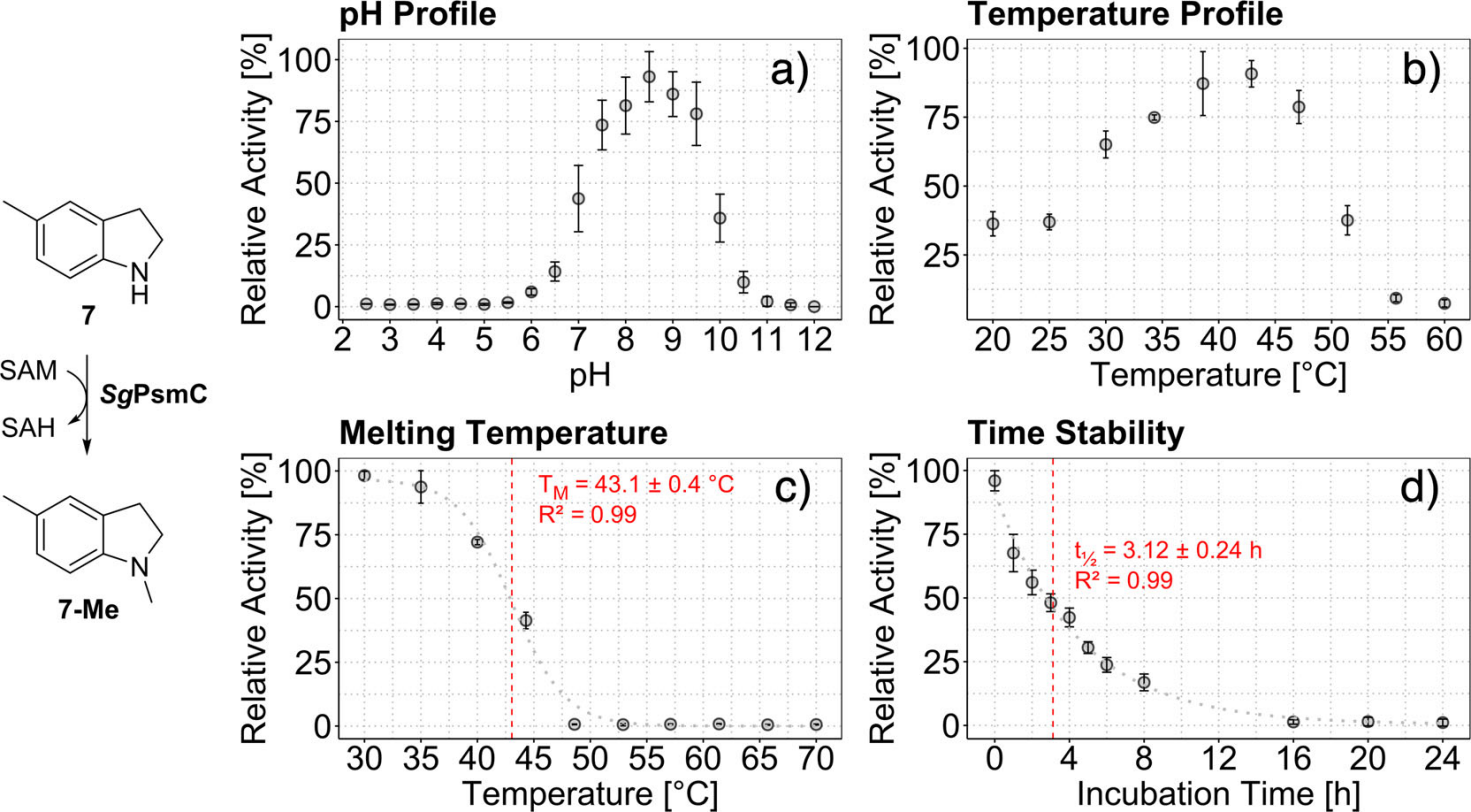
Results of the enzymatic profiling of *Sg*PsmC using the *N*‐methylation of 5‐methylindoline (**7**) as a model reaction (left). a) pH profile, indicating highest activity close to pH 8.5. b) Temperature profile, indicating highest activity at ∼45 °C. c) Melting temperature curve of *Sg*PsmC as determined via residual relative activity (melting temperature *T*_M_ = ∼43 °C). d) Time stability, half‐life *t*_1/2_ = ∼3 h. Activity determined with purified *Sg*PsmC with *n* = 3 replicates; error bars denote SD. Errors of determined parameters are SE. Relative activity normalised to maximum. See Supporting Information for fitting parameters.

The pH optimum was close to pH 8.5 (Figure 2a), and highest activity was observed at ∼45 °C (Figure 2b). However, *Sg*PsmC showed fast thermal inactivation (melting temperature *T*_M_ = ∼43 °C) after a 10 min incubation prior to performing the enzymatic reaction (Figure 2c). To strike a balance between maximum activity and stability, the half‐life of *Sg*PsmC was determined at 35 °C, at which the enzyme had exhibited ∼75% of maximum activity during temperature profiling. Half‐life at 35 °C was ∼3 h, indicating limited stability (Figure 2d). For solvent tolerance and oligomeric state, see Figures S10 and S11.

#### Kinetic Analysis

For kinetic characterisation, single‐substrate kinetics were recorded for indoline (**8**) as a “minimal model substrate” and for the natural pyrroloindoline substrate ***(S,S)*‐9** of *Sg*PsmC (Figure S13). The synthesis of the natural substrate ***(S,S)*‐9** has been described elsewhere.^[^ ^34^ ^]^ Briefly, starting from the respective tryptamine, carbamoylation was performed with methylaminoformyl chloride, followed by enzymatic cyclisation to the pyrroloindoline using the enantioselective *C*‐methyltransferase *Sg*PsmD. For kinetic analysis, the commercial luminescence‐based MTase‐Glo assay^[^ ^57^ ^]^ was used to determine the amount of formed SAH. Substrate concentrations were varied between 1 and 200 µm, and SAM concentration was held constant at 50 µm. The *K*_M_ for the pyrroloindoline ***(S,S)*‐9** was significantly lower (2.33 ± 0.27 µm versus 18.51 ± 3.46 µm) and *k*_cat_ was 205‐fold higher (3.52 × 10^−1^ s^−1^ versus 1.16 × 10^−3^ s^−1^) than for the model substrate **8** (Table 1). The resulting difference in the specificity constant was ∼2400‐fold.

**Table 1:** Results of single‐substrate Michaelis–Menten kinetics analysis using the minimal model substrate indoline (**8**) and the natural *Sg*PsmC substrate ***(S,S)*‐9**. *n* = 3 reactions per time‐point and substrate concentration. Error denotes SE.

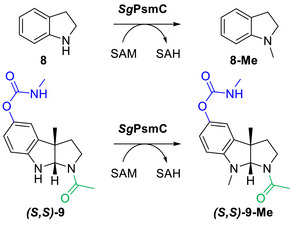				
	*V_max_* [µm/min]	*K_M_* [µm]	*k_cat_* [s^−1^]	*k_cat_/K_M_* [s^−1^ µm ^−1^]
**8**	2.30 × 10^−1^ ± 1.24 × 10^−2^	18.51 ± 3.46	1.16 × 10^−3^ ± 6.25 × 10^−5^	6.25 × 10^−5^ ± 1.22 × 10^−5^
***(S,S)*‐9**	3.50 × 10^−1^ ± 8.35 × 10^−3^	2.33 ± 2.70 × 10^−1^	3.52 × 10^−1^ ± 8.40 × 10^−3^	1.51 × 10^−1^ ± 1.79 × 10^−2^

#### Indoline Substrate Scope

**Figure 3:**
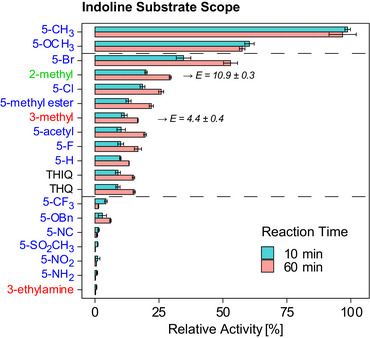
Relative activity of *Sg*PsmC against various substituted indolines. Results shown after 10 min and 60 min reaction time, respectively. For the racemic substrates 2‐methylindoline and 3‐methylindoline, *Sg*PsmC showed weak enantioselectivity (*E*‐values of 10.9 ± 0.3 and 4.4 ± 0.4, respectively). Activity determined with purified *Sg*PsmC with *n* = 3 replicates; error denotes SD. Relative activity normalised to maximum. Dashed lines separate clusters (k‐means, sampling after 10 min).

As indolines were shown to be converted by *Sg*PsmC in preliminary reactions, a larger indoline substrate panel was tested to assess the enzyme's substrate scope for the smaller scaffold and to gain insights regarding the effect of substitutions, primarily at *5*‐position (Figure 3). The substrates can be clustered into three groups based on relative activity. The best accepted substrates by far were 5‐methyl (= 100% relative activity) and 5‐methoxy indoline (60% relative activity). Compounds in the second cluster showed 9%–35% relative activity, including 1,2,3,4‐tetrahydroisoquinoline (THIQ, ∼9%) and 1,2,3,4‐tetrahydroquinoline (THQ, ∼9%). The remaining six compounds showed residual or (close to) zero relative activity. In general, activity varied strongly but no clear trend regarding activating and deactivating groups could be observed except for indolines halogenated at *5*‐position. Here, the relative activity followed the order F < Cl < Br as does the deactivating effect of said halogens.

However, the following points are noteworthy. First, *Sg*PsmC showed, albeit weak, selectivity for the two racemic substrates 2‐methyl and 3‐methyl indoline. *E*‐values could be determined using *ee* values of the respective substrates (*ee*_S_) and products (*ee*_P_) determined via chiral gas chromatography (GC), amounting to 10.9 ± 0.3 and 4.4 ± 0.4, for 2‐methyl and 3‐methyl indoline, respectively (Figures 3, S15, and S16a,b). An attempt to explain the difference in *E*‐values for the two indoline substrates can be made using a simple isosteric model (Figure S17): the methyl group of 2‐methyl indoline has – irrespectively of the absolute configuration of the *C2* centre – an isosteric atom on the *3a*‐methylated (*S,S*)‐configured pyrroloindoline scaffold of *Sg*PsmC's natural substrate, which is only the case for (*R*)‐ but not for (*S*)‐3‐methyl indoline. While not on par with nonenzymatic methods (showing a selectivity factor of *s* = 25 for 2‐methyl indoline^[^ ^58^ ^]^), it remains to be seen if *Sg*PsmC shows stronger stereo‐discrimination for more strongly decorated indolines.

Second, a comparison with the *C*‐methyltransferase *Sg*PsmD from the same biosynthesis cluster can be made. While the principal determinant for *Sg*PsmD for high conversion of tryptamine substrates (yielding pyrroloindoline products) besides substrate size is carbamoylation at 5‐position,^[^ ^34^, ^35^ ^]^ *Sg*PsmC does not appear to suffer from the latter restriction. However, this could be simply due to the lower steric demand of the indolines compared to the tryptamines, the former allowing a more forgiving productive substrate binding. Nevertheless, if carbamoylation were to be a non‐prerequisite for acceptance of pyrroloindolines, this would open an additional chemical space for the potential application of *Sg*PsmC.

### Selectivity Against Pyrroloindolines

#### Pyrroloindoline Substrate Scope and Relative Enantioselectivity

**Scheme 2:**
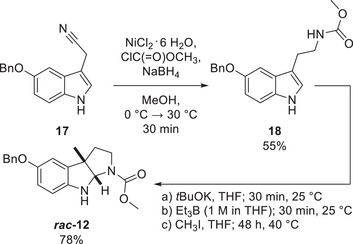
Synthesis of ***rac*‐12**, performed analogously to the synthesis of pyrroloindolines ***rac*‐9** through ***rac*‐11**, as described previously.^[^ ^34^ ^]^

To examine the enantioselectivity of *Sg*PsmC and for better comparison between *Sg*PsmC and *Sg*PsmD, a set of racemic pyrroloindolines was tested (***rac*‐9** to ***rac*‐16**, Table 2). These included the racemic natural substrate ***rac*‐9**, as well as derivative ***rac*‐10** with no modification at *5*‐position to determine the necessity of the carbamate functionality. Additionally, bulkier substrates carrying a *t*Bu‐amide (***rac*‐11**), as well as an *O*‐benzyl protected methyl carbamate (***rac*‐12**) were tested. Further substrates included *C3*‐cyclised melatonin‐derivatives with different modifications at *3a*‐position (***rac*‐13** to ***rac*‐15**). The compounds’ syntheses have been described previously,^[^ ^34^, ^59^ ^]^ based on which ***rac*‐12** and ***rac*‐16** were obtained similarly. Compound ***rac*‐12** was accessed in two steps from the respective *O*‐benzyl‐protected indole nitrile **17** after carbamoylation with methylaminoformyl chloride and cyclisation using CH_3_I with 43% yield over all steps (Scheme 2). The second step was also used to access ***rac*‐16** using ethyl iodide and starting from the respective *N*‐acetyl tryptamine in 77% yield (see Supporting Information).

**Table 2:** Substrate scope and enantioselectivity of *Sg*PsmC against a selection of pyrroloindolines. For determination of *E*‐values,^[^ ^62^, ^63^ ^]^ pairs of *ee*_S_ and *ee*_P_ of reaction samples were determined using chiral HPLC and the function *ee*_P_(*ee*_S_, *E*) was then fitted to the recorded data. For *ee* values > 98%, these were set to 98%. In cases where only determination of *ee*_S_ was possible, conversion was measured in parallel by achiral HPLC. The function conversion(*ee*_S_, *E*) was then fitted to this alternative data set. Due to the error‐prone determination of conversion values, the final *E*‐values yielded by the latter method should be regarded only as a qualitative measure. Due to the precision of the recorded data in general, *E*‐values > 100 are not reported here.^[^ ^61^ ^]^ The absolute configuration of the preferred substrate enantiomer was determined by comparing chiral HPLC‐CD traces with calculated ECD spectra. For more details, see Supporting Information.

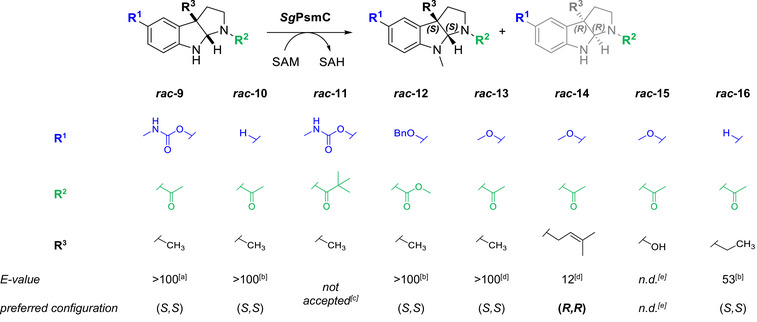

^a)^ *E*‐value derived by constant concentration of disfavoured substrate and product enantiomers.

^b)^ *E*‐value determined by fitting of *ee_P_* (*ee_S_*, *E*).

^c)^ No detection of SAH formation after 30 min.

^d)^ *E*‐value determined by fitting of conversion(*ee*_S_, *E*).

^e)^ Chiral separation not achieved.

**Figure 4:**
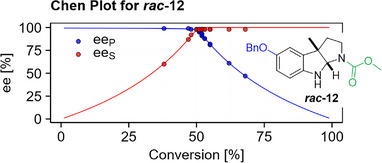
Chen plot^[^ ^60^ ^]^ for the *N*‐methylation of ***rac*‐12** as an example for the high enantioselectivity of *Sg*PsmC. Dots indicate experimental data pairs, which include data from reaction optimisation and of scale‐up (see further below). Due to limited precision, apparent *ee* values > 98% were set to 98%, and *E*‐values > 100 are not reported.^[^ ^61^ ^]^ *E*‐value determined by fitting of *ee_P_* (*ee_S_*, *E*) (see Supporting Information).

The substrate panel revealed that *Sg*PsmC exhibits high enantioselectivity – at least as can be reliably determined by chiral chromatography – without being restricted to the carbamoyl moiety of its natural substrate ***rac*‐9**, as the smaller 5‐methoxy (***rac*‐13**) and 5‐unsubstituted (***rac*‐10**) derivatives were also converted with equally high enantioselectivity (*E* > 100). While *Sg*PsmC tolerated additional steric bulk to a certain extent (***rac*‐12**, *5*‐benzyl ether and *N1*‐methyl carbamate, *E* > 100, see Figure 4), limits were set by the *N1*‐*t*Bu amide ***rac*‐11,** which was not converted.

Reactions with the melatonin‐derived substrates showed tolerance for residues other than methyl at *3a*‐position, albeit with diminishing conversion of the racemate and a penalty in selectivity (Table 2). Compared with the corresponding *3a*‐methylated substrates for which a selectivity of *E* > 100 was determined, *Sg*PsmC showed much lower selectivity against the *3a*‐ethylated ***rac*‐16** and *3a*‐prenylated ***rac*‐14** with *E* = ∼53 and *E* = ∼12, respectively. It appears that an important determining factor for the enantioselectivity of *Sg*PsmC lies in the size of the residue at *3a*‐position. While conversion of the *3a*‐hydroxy substrate ***rac*‐15** was observed, no chiral separation could be achieved and therefore no E‐value could be determined.

#### Absolute Configuration

As both physostigmine (**1**), the natural product of the *psm* gene cluster, as well as the natural substrate of *Sg*PsmC are (*S,S*)‐configurated,^[^ ^34^, ^54^ ^]^ one may assume the same for the general absolute stereo‐preference of *Sg*PsmC. To test this assumption, electronic circular dichroism (ECD) spectra of all converted pyrroloindolines were calculated. Calculated spectra were in qualitative agreement with experimental spectra recorded in stop‐flow mode during chiral high‐pressure liquid chromatography (HPLC) separation (Figures S21a–S28a). Due to limited sensitivity, the polarity of the weakest intensity peaks at ∼310 nm could not be given faithfully for all compounds (see Supporting Information). Therefore, configurational assignment relied primarily on the characteristic peak at ∼240 nm. Comparison of calculated ECD spectra to chiral HPLC‐CD traces of racemic substrates allowed configurational assignment to substrate peaks. In turn, comparison hereof with HPLC‐UV traces of enzymatic reactions showed that (inside the given panel) the preferred scaffold of *Sg*PsmC is indeed (*S,S*)‐configurated. An exception hereof was encountered for the *3a*‐prenylated ***rac*‐14** (Figure S26a), the very substrate for which the lowest selectivity of *E* = ∼12 was determined. These results for ***rac*‐14** were further validated by applying the same methodology to the *N*‐methylated product ***rac*‐14‐Me** (Figure S26b).

The high enantioselectivity shown by *Sg*PsmC for *3a*‐methylated pyrroloindolines in combination with their straightforward asymmetric chemical synthesis led us to examine the possibility of using *Sg*PsmC in an enzymatic KR. While *Sg*PsmD allows enantioselective access to the *3a*‐methylated (*S,S*)‐configured scaffold, a chemo‐enzymatic synthesis using *Sg*PsmC would allow access to the corresponding (*R,R*)‐configured substrate and also to the *N*‐methylated (*S,S*)‐product.

### Laboratory Preparative Scale‐Up

#### Choice of Substrate for Scale‐Up, Catalyst Formulation, and Reaction Optimisation

To examine the applicability of *Sg*PsmC for KR, we chose to focus on the substrate ***rac*‐12** as it is a) easily obtained chemically, and b) a flexible starting point for subsequent modification. For example, after the *Sg*PsmC reaction, the *N*‐methylated product and the resolved substrate could be used in a mirrored chemical synthesis yielding (+)‐posiphen (**2**) and (–)‐phenserine (**3**), respectively.

With applicability in mind, using purified enzyme would have been labour‐intensive: ideally, the catalysts formulation should be easily obtained. The use of cell‐free extracts (CFEs), while simple, allowed a maximum conversion of only ∼39% when using 1 mm of ***rac*‐12**, which even under ideal conditions, would only lead to an *ee*_S_ of ∼62% (assuming *E* = 100).

This may be due to the limited stability of the catalyst, which had already been noted during enzyme profiling (see Figure 2d). Furthermore, an apparent decrease in conversion over prolonged reaction time in selected cases may indicate degradation of product (see Figure S29). However, as no degradation products were observed, this indication is non‐conclusive, and the observation could also be explained by low recovery rates. Irrespective of the underlining cause, instead of increasing the catalyst density of the CFEs indefinitely to reach 50% conversion, and in order to precautionarily hinder compounds being exposed to prolonged reaction times, salted‐out *Sg*PsmC was tested instead.

Using salted‐out enzymes allows for a straightforward concentration of the catalysts and the formulation can have a stabilising effect on enzymes during storage.^[^ ^64^ ^]^ A precipitation‐profile for *Sg*PsmC using (NH_4_)_2_SO_4_ was recorded, and a highly concentrated and (albeit weakly, only ∼5.9‐fold) partially purified catalysts formulation was obtained (Figure S30a,b). With the salted‐out catalyst, conversions close to 50% were reached. To determine a sufficient catalyst loading and reaction time, *ee*_P_ was monitored during respective reactions (Figure S32). As expected,^[^ ^65^ ^]^ greatly prolonging reaction time or increasing catalyst loading too strongly led to a decrease in *ee*_P_. Similar observations were made for the smaller pyrroloindoline ***rac*‐10** (Figure S22b). Detrimental effects could be observed when increasing reaction time to 24 h with the lowest tested catalyst load (0.08 U mL^−1^ activity against 5‐Me‐indoline (**7**)), showing a decreased *ee*_P_ of ∼91%. Similar loss of chiral resolution was observed when using 0.16 U mL^−1^ of catalyst after 6 h reaction time (*ee*_P_ ∼93%). Close to optimal results were obtained for a catalyst load of 0.08 U mL^−1^ and 4 h reaction time.

#### SAM Generation

For scale‐up, cofactor generation had to be addressed. Different enzymatic systems for both cofactor generation and recycling have been described,^[^ ^10^, ^18^, ^19^, ^24^, ^66^, ^67^ ^]^ but the two simplest systems are at the same time the most widely used. One generates SAM from ATP and l‐methionine in a linear enzymatic cascade employing an MAT, potentially in accord with an SAH nucleosidase to hinder product inhibition.^[^ ^18^, ^22^ ^]^ The other system re‐methylates the formed SAH using an HMT and sacrificial CH_3_I.^[^ ^24^ ^]^ Under common conditions,^[^ ^34^ ^]^ using the recycling system rendered the KR by *Sg*PsmC ineffective due to non‐selective *N*‐methylation owing to the presence of CH_3_I (Figures S33 and S34a). The system employing *Tk*MAT from *Thermococcus kodakarensis*,^[^ ^68^ ^]^ on the other hand, did not lead to any background reaction and was thus chosen as the preferred cofactor system, as hindering unselective background methylation is essential for the KR to be of use.

While not pursued in this work, additional options would be available to reduce the background methylation of the HMT system. *Ct*HMT from *Chloracidobacterium thermophilum* used here is comparably low performing (*k*_cat_/*K*_M_ = 51 m ^−1^ s^−1^).^[^ ^24^ ^]^ The use of more performant HMTs such as from *Arabidopsis thaliana* (*k*_cat_/*K*_M_ = 4200 m ^−1^ s^−1^)^[^ ^13^ ^]^ or *Aspergillus clavatus* (full conversion of 1 mm SAH in ∼5 min^[^ ^33^ ^]^; *k*_cat_/*K*_M_ = 200 m ^−1^ s^−1^ for methyl tosylate^[^ ^23^ ^]^) could lead to lower background methylation by faster consumption of methyl iodide, provided that the clear bottleneck of the coupled system is not the methyltransferase. In the present case, 20 µm SAH (2 mol%) were used as in previous work.^[^ ^34^ ^]^ Increasing the amount of SAH (or SAM), in combination with the use of a more efficient HMT, would also be a sensible strategy to lower the background reaction. However, one should note, nevertheless, that both HMT‐ and MAT‐based systems have their respective advantages and disadvantages, making the final choice dependent on the individual case (see Supporting Information for a detailed discussion).

#### Final Scale‐Up

**Scheme 3:**
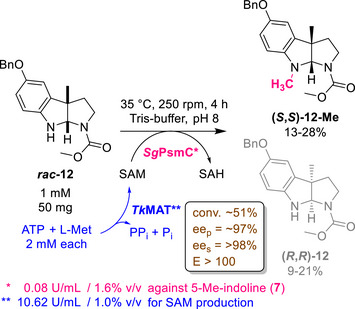
Final conditions for the KR of ***rac*‐12** using salted‐out *Sg*PsmC and *Tk*MAT at a laboratory scale. Isolated yield spans based on two syntheses.

Similarly to *Sg*PsmC, a precipitation‐profile was recorded for *Tk*MAT (Figure S35b,c). Due to fast thermal inactivation of *Sg*PsmC, a common reaction temperature of 35 °C was chosen for the coupled system, below the temperature at which *Tk*MAT showed highest activity (Figure S36). Similar pH profiles (Figure S37) allowed the reaction to perform close to the optimal pH of both enzymes. The final conditions for the up‐scaled reaction are shown in Scheme 3. After 4 h reaction time, conversion of ∼51% and an *ee*_P_ of ∼97% were reached at a 50 mg (1 mm) substrate scale, corresponding to an *E*‐value of > 100, thus highlighting the selectivity and scalability of the system. However, isolated yields were low (13% for **(*S,S*)‐12‐Me**, 9% for reisolated **(*R,R*)‐12**), primarily due to inefficient work‐up of the high protein‐density reaction mixture forming a pronounced interphase during extraction. Repetition of the up‐scaled reaction using centrifugation for phase‐separation and repeated washing of the resulting enzyme pellet with buffer and MeOH (see Supporting Information) led to improved, albeit still unsatisfactory, isolated yields of 28% for **(*S,S*)‐12‐Me** and 21% for **(*R,R*)‐12**. Alternative approaches to circumvent and/or improve the problematic work‐up could be the use of pre‐purified, immobilised enzyme used in batch or in continuous flow set‐ups, potentially facilitating catalyst separation.

## Conclusion

In this work, we have performed a biochemical characterisation of the *N*‐methyltransferase *Sg*PsmC and highlighted the catalyst's potential in accessing pyrroloindolines. *Sg*PsmC appears to be less restricted by the functionality at *5*‐position of the pyrroloindoline scaffold compared to the *C*‐methyltransferase *Sg*PsmD (acting one step earlier in the biosynthesis of physostigmine (**1**))^[^ ^34^, ^35^, ^54^ ^]^ and further accepts *3a*‐ethylated or even *3a*‐prenylated substrates, thus allowing the targeting of a different chemical space. However, a lower selectivity was observed for substrates with residues other than methyl at *3a*‐position. Interestingly, the general absolute stereo‐preference of *Sg*PsmC for the (*S,S*)‐configurated scaffold is inverted for the *3a*‐prenyltated substrate. The catalyst's high enantioselectivity against *3a*‐methylated pyrroloindolines revealed during substrate scope profiling pointed to its use in the KR of easily accessible racemic pyrroloindolines.

Catalytic KR is a well‐established strategy for accessing enantioenriched amines and, despite advances in asymmetric synthesis, is still used in cases where preparation of racemic amines is easier.^[^ ^69^ ^]^ However, using *N*‐alkylation,^[^ ^70^, ^71^ ^]^ or more specifically *N*‐methylation,^[^ ^55^ ^]^ is an uncommon approach in KR. This is presumably due to the strong nucleophilicity of amines in combination with the generally reactive alkylation/methylation agents, leading to a lack of selectivity and to background reactions. While enzymes (mainly hydrolases,^[^ ^72^ ^]^ but also oxygenases^[^ ^73^ ^]^ and others^[^ ^74^ ^]^), are commonly employed as catalysts in enzymatic KR, methyltransferases in general are here also underrepresented,^[^ ^37^ ^]^ and *N*‐methyltransferases have not yet been employed. The high enantioselectivity of *Sg*PsmC in combination with the absence of unselective background methylation thanks to the use of the linear MAT‐supply system for the cosubstrate SAM made the application of an *N*‐methylation‐based KR feasible in this work.

While enzyme engineering of pyrroloindoline‐forming *C3*‐methyltransferases to invert their enantioselectivity would be a further suitable strategy to access both scaffold enantiomers, we pursued enzymatic KR as an alternative approach and have demonstrated its applicability with the accessible substrate ***rac*‐12**. From this point, derivatisation toward the enantiomers (+)‐posiphen (**2**) and (–)‐phenserine (**3**)^[^ ^50^, ^52^ ^]^ is possible, as is the general access to both enantiomers of the *N*‐methylated scaffold by subsequent chemical methylation. Broad applicability could, however, be further widened by searching for more stable enzyme formulations and homologues, as well as promiscuous catalysts allowing resolution by other means than methylation alone. As the molecular basis of the selectivity of *Sg*PsmC is still unknown, crystallisation experiments and in silico analyses are in train which will also help determine how knowledge regarding mechanism and selectivity of different pyrroloindoline targeting methyltransferases can be transferred between catalysts for their optimisation and engineering to expand their applicability even further.

## Supporting Information

The authors have cited additional references within the Supporting Information.

## Conflict of Interests

The authors declare no conflict of interest.

## Supporting information



Supporting Information

## Data Availability

The data that support the findings of this study are available in the supplementary material of this article.

## References

[1] P. N. Devine , R. M. Howard , R. Kumar , M. P. Thompson , M. D. Truppo , N. J. Turner , Nat. Rev. Chem. 2018, 2, 409–421. 10.1038/s41570-018-0055-1.

[2] S. P. France , R. D. Lewis , C. A. Martinez , JACS Au 2023, 3, 715–735. 10.1021/jacsau.2c00712. 37006753 PMC10052283

[3] M. D. Truppo , ACS Med. Chem. Lett. 2017, 8, 476–480. 10.1021/acsmedchemlett.7b00114. 28523096 PMC5430392

[4] E. Romero , B. S. Jones , B. N. Hogg , A. R. Casamajo , M. A. Hayes , S. L. Flitsch , N. J. Turner , C. Schnepel , Angew. Chem., Int. Ed. 2021, 60, 16824–16855. 10.1002/anie.202014931 PMC8359417 33453143

[5] R. A. Sheldon , J. M. Woodley , Chem. Rev. 2018, 118, 801–838. 10.1021/acs.chemrev.7b00203. 28876904

[6] E. Abdelraheem , B. Thair , R. F. Varela , E. Jockmann , D. Popadić , H. C. Hailes , J. M. Ward , A. M. Iribarren , E. S. Lewkowicz , J. N. Andexer , P. Hagedoorn , U. Hanefeld , ChemBioChem 2022, 23, e202200212. 35691829 10.1002/cbic.202200212 PMC9539859

[7] C. Dalhoff , G. Lukinavičius , S. Klimas̆auskas , E. Weinhold , Nat. Chem. Biol. 2006, 2, 31–32. 10.1038/nchembio754. 16408089

[8] B. J. C. Law , A.‐W. Struck , M. R. Bennett , B. Wilkinson , J. Micklefield , Chem. Sci. 2015, 6, 2885–2892. 29403635 10.1039/c5sc00164a PMC5729408

[9] C. Zhang , R. L. Weller , J. S. Thorson , S. R. Rajski , J. Am. Chem. Soc. 2006, 128, 2760–2761. 10.1021/ja056231t. 16506729

[10] S. Singh , J. Zhang , T. D. Huber , M. Sunkara , K. Hurley , R. D. Goff , G. Wang , W. Zhang , C. Liu , J. Rohr , S. G. Van Lanen , A. J. Morris , J. S. Thorson , Angew. Chem., Int. Ed. 2014, 53, 3965–3969. 10.1002/anie.201308272. PMC4076696 24616228

[11] K. Hartstock , B. S. Nilges , A. Ovcharenko , N. V. Cornelissen , N. Püllen , A.‐M. Lawrence‐Dörner , S. A. Leidel , A. Rentmeister , Angew. Chem., Int. Ed. 2018, 57, 6342–6346. 10.1002/anie.201800188. 29461645

[12] C. Sommer‐Kamann , A. Fries , S. Mordhorst , J. N. Andexer , M. Müller , Angew. Chem., Int. Ed. 2017, 56, 4033–4036. 10.1002/anie.201609375. 28247461

[13] Q. Tang , C. W. Grathwol , A. S. Aslan‐Üzel , S. Wu , A. Link , I. V. Pavlidis , C. P. S. Badenhorst , U. T. Bornscheuer , Angew. Chem., Int. Ed. 2021, 60, 1524–1527. 10.1002/anie.202013871. PMC7839550 33108827

[14] H. Stecher , M. Tengg , B. J. Ueberbacher , P. Remler , H. Schwab , H. Griengl , M. Gruber‐Khadjawi , Angew. Chem., Int. Ed. 2009, 48, 9546–9548. 10.1002/anie.200905095. 19899087

[15] J. Peng , C. Liao , C. Bauer , F. P. Seebeck , Angew. Chem., Int. Ed. 2021, 60, 27178–27183. 10.1002/anie.202108802. 34597444

[16] H. Simon‐Baram , S. Roth , C. Niedermayer , P. Huber , M. Speck , J. Diener , M. Richter , S. Bershtein , ChemBioChem. 2022, 23, e202200162. 10.1002/cbic.202200162. 35785511 PMC9542197

[17] J. M. Lipson , M. Thomsen , B. S. Moore , R. P. Clausen , J. J. La Clair , M. D. Burkart , ChemBioChem 2013, 14, 950–953. 10.1002/cbic.201300221. 23650044 PMC3749784

[18] S. Mordhorst , J. Siegrist , M. Müller , M. Richter , J. N. Andexer , Angew. Chem., Int. Ed. 2017, 56, 4037–4041. 10.1002/anie.201611038. 28170142

[19] D. Popadić , D. Mhaindarkar , M. H. N. D. Thai , H. C. Hailes , S. Mordhorst , J. N. Andexer , RSC Chem. Biol. 2021, 2, 883–891. 34179784 10.1039/d1cb00033k PMC8190896

[20] E. Jockmann , F. Subrizi , M. K. F. Mohr , E. M. Carter , P. M. Hebecker , D. Popadić , H. C. Hailes , J. N. Andexer , ChemCatChem 2023, 15, e202300930. 10.1002/cctc.202300930.

[21] J. L. Hoffman , Biochemistry 1986, 25, 4444–4449. 10.1021/bi00363a041. 3530324

[22] J. Siegrist , S. Aschwanden , S. Mordhorst , L. Thöny‐Meyer , M. Richter , J. N. Andexer , ChemBioChem 2015, 16, 2576–2579. 10.1002/cbic.201500410. 26437744

[23] X. Wen , F. Leisinger , V. Leopold , F. P. Seebeck , Angew. Chem., Int. Ed. 2022, 61, e202208746. 10.1002/anie.202208746. 35989225

[24] C. Liao , F. P. Seebeck , Nat. Catal. 2019, 2, 696–701. 10.1038/s41929-019-0300-0.

[25] K. H. Schülke , J. S. Fröse , A. Klein , M. Garcia‐Borràs , S. C. Hammer , ChemBioChem 2024, 25, e202400079. 38477872 10.1002/cbic.202400079

[26] H. Coiner , G. Schröder , E. Wehinger , C.‐J. Liu , J. P. Noel , W. Schwab , J. Schröder , Plant J. Cell Mol. Biol. 2006, 46, 193–205. 10.1111/j.1365-313X.2006.02680.x. PMC2860623 16623883

[27] F. Ospina , K. H. Schülke , M. Schnutenhaus , A. Klein , O. Desai , S. Jain , C. Krofta , L. Stratmann , J. Yang , H. Gröger , S. C. Hammer , Angew. Chem. 2025, 137, e202510300. 10.1002/anie.202510300 PMC12402838 40631893

[28] E. Jockmann , H. Girame , W. Steinchen , K. Kind , G. Bange , K. Tittmann , M. Müller , F. Feixas , M. Garcia‐Borràs , J. N. Andexer , ACS Catal. 2025, 15, 6410–6425. 10.1021/acscatal.5c00834. 40270878 PMC12013660

[29] L. L. Bengel , B. Aberle , A. Egler‐Kemmerer , S. Kienzle , B. Hauer , S. C. Hammer , Angew. Chem., Int. Ed. 2021, 60, 5554–5560. 10.1002/anie.202014239. PMC7986378 33300646

[30] Q. Tang , U. T. Bornscheuer , I. V. Pavlidis , ChemCatChem 2019, 11, 3227–3233. 10.1002/cctc.201900606.

[31] B. J. C. Law , M. R. Bennett , M. L. Thompson , C. Levy , S. A. Shepherd , D. Leys , J. Micklefield , Angew. Chem. 2016, 128, 2733–2737. 10.1002/anie.201508287 PMC4770447 26797714

[32] R. Roddan , A. Sula , D. Méndez‐Sánchez , F. Subrizi , B. R. Lichman , J. Broomfield , M. Richter , J. N. Andexer , J. M. Ward , N. H. Keep , H. C. Hailes , Commun. Chem. 2020, 3, 1–10. 10.1038/s42004-020-00416-8. 36703392 PMC9814250

[33] F. Ospina , K. H. Schülke , J. Soler , A. Klein , B. Prosenc , M. Garcia‐Borràs , S. C. Hammer , Angew. Chem., Int. Ed. 2022, 61, e202213056. 10.1002/anie.202213056. PMC9827881 36202763

[34] P. Schneider , B. Henßen , B. Paschold , B. P. Chapple , M. Schatton , F. P. Seebeck , T. Classen , J. Pietruszka , Angew. Chem., Int. Ed. 2021, 60, 23412–23418. 10.1002/anie.202107619 PMC8596708 34399441

[35] D. A. Amariei , N. Pozhydaieva , B. David , P. Schneider , T. Classen , H. Gohlke , O. H. Weiergräber , J. Pietruszka , ACS Catal. 2022, 12, 14130–14139. 10.1021/acscatal.2c04240.

[36] M. Haase , B. David , B. Paschold , T. Classen , P. Schneider , N. Pozhydaieva , H. Gohlke , J. Pietruszka , ACS Catal. 2024, 14, 227–236. 10.1021/acscatal.3c04952. 38205025 PMC10775177

[37] S. Rydzek , F. Guth , S. Friedrich , J. Noske , B. Höcker , F. Hahn , ChemCatChem 2024, 16, e202400883. 10.1002/cctc.202400883.

[38] S. Ju , K. P. Kuzelka , R. Guo , B. Krohn‐Hansen , J. Wu , S. K. Nair , Y. Yang , Nat. Commun. 2023, 14, 5704. 10.1038/s41467-023-40980-w. 37709735 PMC10502145

[39] C. Sun , W. Tian , Z. Lin , X. Qu , Nat. Prod. Rep. 2022, 39, 1721–1765. 10.1039/D2NP00030J. 35762180

[40] R. Raju , A. M. Piggott , X.‐C. Huang , R. J. Capon , Org. Lett. 2011, 13, 2770–2773. 10.1021/ol200904v. 21513295

[41] P. W. Moore , J. J. Rasimas , J. W. Donovan , J. Med. Toxicol. 2015, 11, 159–160. 10.1007/s13181-014-0442-z. 25339374 PMC4371033

[42] E. Viziteu , C. Grandmougin , H. Goldschmidt , A. Seckinger , D. Hose , B. Klein , J. Moreaux , Br. J. Cancer 2016, 114, 519–523. 10.1038/bjc.2016.20. 26867162 PMC4782210

[43] T. A. Amador , L. Verotta , D. S. Nunes , E. Elisabetsky , Planta Med. 2000, 66, 770–772. 10.1055/s-2000-9604. 11199142

[44] A. Galli , G. Renzi , E. Grazzini , R. Bartolini , P. Aiello‐Malmberg , A. Bartolini , Biochem. Pharmacol. 1982, 31, 1233–1238. 10.1016/0006-2952(82)90009-0. 7092918

[45] M. G. Kulkarni , A. P. Dhondge , A. S. Borhade , D. D. Gaikwad , S. W. Chavhan , Y. B. Shaikh , V. B. Ningdale , M. P. Desai , D. R. Birhade , M. P. Shinde , Tetrahedron Lett. 2009, 50, 2411–2413. 10.1016/j.tetlet.2009.03.012.

[46] J.‐C. Yi , C. Liu , L.‐X. Dai , S.‐L. You , Chem. Asian J. 2017, 12, 2975–2979. 10.1002/asia.201701151. 28967186

[47] E. C. Gentry , L. J. Rono , M. E. Hale , R. Matsuura , R. R. Knowles , J. Am. Chem. Soc. 2018, 140, 3394–3402. 10.1021/jacs.7b13616. 29432006 PMC5896747

[48] T. Bui , S. Syed , C. F. I. Barbas , J. Am. Chem. Soc. 2009, 131, 8758–8759. 10.1021/ja903520c. 19499923

[49] J. E. Spangler , H. M. L. Davies , J. Am. Chem. Soc. 2013, 135, 6802–6805. 10.1021/ja4025337. 23607705

[50] A. F. Teich , E. Sharma , E. Barnwell , H. Zhang , A. Staniszewski , T. Utsuki , V. Padmaraju , C. Mazell , A. Tzekou , K. Sambamurti , O. Arancio , M. L. Maccecchini , Alzheimers Dement. N. Y. N 2018, 4, 37–45. 10.1016/j.trci.2017.12.001 PMC6021259 29955650

[51] K. T. Y. Shaw , T. Utsuki , J. Rogers , Q.‐S. Yu , K. Sambamurti , A. Brossi , Y.‐W. Ge , D. K. Lahiri , N. H. Greig , Proc. Natl. Acad. Sci. USA 2001, 98, 7605–7610. 10.1073/pnas.131152998. 11404470 PMC34715

[52] S. Mikkilineni , I. Cantuti‐Castelvetri , C. M. Cahill , A. Balliedier , N. H. Greig , J. T. Rogers , Park. Dis 2012, 2012, 142372. 10.1155/2012/142372 PMC3368596 22693681

[53] Q. Yu , X.‐F. Pei , H. W. Holloway , N. H. Greig , A. Brossi , J. Med. Chem. 1997, 40, 2895–2901. 10.1021/jm970210v. 9288171

[54] J. Liu , T. Ng , Z. Rui , O. Ad , W. Zhang , Angew. Chem., Int. Ed. 2014, 53, 136–139. 10.1002/anie.201308069. 24227628

[55] J. Blum , D. Gelman , Z. Aizenshtat , S. Wernik , H. Schumann , Tetrahedron Lett. 1998, 39, 5611–5614. 10.1016/S0040-4039(98)01054-5.

[56] D. A. Amariei , J. Tenhaef , T. Classen , B. David , T. M. Rosch , H. Gohlke , S. Noack , J. Pietruszka , Catal. Sci. Technol. 14, 6298–6306, 2024, 10.1039/D4CY00657G.

[57] K. Hsiao , H. Zegzouti , S. A. Goueli , Epigenomics 2016, 8, 321–339. 10.2217/epi.15.113. 26950288

[58] F. O. Arp , G. C. Fu , J. Am. Chem. Soc. 2006, 128, 14264–14265. 10.1021/ja0657859. 17076493 PMC2569996

[59] D. A. Amariei , M. Haase , M. K. T. Klischan , M. Wäscher , J. Pietruszka , ChemCatChem 2024, 16, e202400052. 10.1002/cctc.202400052.

[60] C. S. Chen , Y. Fujimoto , G. Girdaukas , C. J. Sih , J. Am. Chem. Soc. 1982, 104, 7294–7299. 10.1021/ja00389a064.

[61] J. Pietruszka , A. C. M. Rieche , T. Wilhelm , A. Witt , Adv. Synth. Catal. 2003, 345, 1273–1286. 10.1002/adsc.200303137.

[62] J. L. L. Rakels , A. J. J. Straathof , J. J. Heijnen , Enzyme Microb. Technol. 1993, 15, 1051–1056. 10.1016/0141-0229(93)90053-5. 7505594

[63] A. J. J. Straathof , J. A. Jongejan , Enzyme Microb. Technol. 1997, 21, 559–571. 10.1016/S0141-0229(97)00066-5.

[64] K. C. Duong‐Ly , S. B. Gabelli in Methods Enzymol. (Ed.: J. Lorsch ), Academic Press, San Diego, Waltham, Amsterdam, Oxford, London, 2014, pp. 85–94.

[65] K. Faber , Biotransformations in Organic Chemistry, Springer‐Verlag, Berlin, Heidelberg 2011. 10.1007/978-3-642-17393-6.

[66] M. Thomsen , S. B. Vogensen , J. Buchardt , M. D. Burkart , R. P. Clausen , Org. Biomol. Chem. 2013, 11, 7606. 10.1039/c3ob41702f. 24100405 PMC3871182

[67] L. Gericke , D. Mhaindarkar , L. C. Karst , S. Jahn , M. Kuge , M. K. F. Mohr , J. Gagsteiger , N. V. Cornelissen , X. Wen , S. Mordhorst , H. J. Jessen , A. Rentmeister , F. P. Seebeck , G. Layer , C. Loenarz , J. N. Andexer , ChemBioChem 2023, 24, e202300133. 36942622 10.1002/cbic.202300133

[68] J. Schlesier , J. Siegrist , S. Gerhardt , A. Erb , S. Blaesi , M. Richter , O. Einsle , J. N. Andexer , BMC Struct. Biol. 2013, 13, 22. 10.1186/1472-6807-13-22. 24134203 PMC3853416

[69] W. Liu , D. Wang , D. Zhang , X. Yang , Synlett 2022, 33, 1788–1812.

[70] S. Shirakawa , X. Wu , K. Maruoka , Angew. Chem., Int. Ed. 2013, 52, 14200–14203. 10.1002/anie.201308237. 24222438

[71] X. L. Hou , B. H. Zheng , Org. Lett. 2009, 11, 1789–1791. 10.1021/ol9002543. 19301928

[72] M. Ahmed , T. Kelly , A. Ghanem , Tetrahedron 2012, 68, 6781–6802. 10.1016/j.tet.2012.05.049.

[73] L. A. Harwood , L. L. Wong , J. Robertson , Angew. Chem., Int. Ed. 2021, 60, 4434–4447. 10.1002/anie.202011468. PMC7986699 33037837

[74] C. Aranda , G. Oksdath‐Mansilla , F. R. Bisogno , G. De Gonzalo , Adv. Synth. Catal. 2020, 362, 1233–1257. 10.1002/adsc.201901112.

