# Supplementary material for: Characterisation of the N‐Methyltransferase SgPsmC: Application in the Kinetic Resolution of Pyrroloindolines
Source: Angew Chem Int Ed Engl. 2025 Nov 22;65(2):e15459. doi: 10.1002/anie.202515459 (PMC12790363; doi:10.1002/anie.202515459)
Supplement: Supplementary file 1 — Supporting Information [file ANIE-65-e15459-s001.pdf]

Supporting Information

Characterisation of the *N*-Methyltransferase *Sg*PsmC: Application in the Kinetic Resolution of Pyrroloindolines

Benjamin Panagiotis Chapple[a], Lia Nitz[a], Pascal Schneider[a], Birgit Henßen[b], Sebastian Myllek[a], Mona Haase[a], Thomas Classen[b] and Jörg Pietruszka*[a,b]

Table of contents

General considerations and software S3

(A) General notes S3

(B) Software used S3

Experimental section and computational methods S4

(A) Cultivation media and antibiotics S4

(B) Generation of chemically competent cells S4

(C) Transformation of chemically competent cells S4

(D) Preparation of *E. coli* glycerol stocks for cultivations S5

(E) Non-optimised heterologous expression of *Sg*PsmC S5

(F) Heterologous expression of *Tk*MAT and *Ct*HMT S6

(G) Cell disruption S6

(H) Enzyme purification *via* immobilised metal-ion affinity chromatography (IMAC) S6

(I) Precipitation profiles of *Sg*PsmC and *Tk*MAT S7

(J) DoE-guided expression optimisation S8

(K) Bradford assay S10

(L) Sodium dodecyl sulphate-polyacrylamide gel electrophoresis (SDS-PAGE)[26] S10

(M) Determination of *Sg*PsmC protein content using dot-blot S11

(N) Biochemical characterisation: General procedure for analytical-scale enzyme reactions S12

(O) Biochemical characterisation of *Sg*PsmC: Procedures for enzyme profiling S12

(P) Kinetic analysis of *Sg*PsmC S13

(Q) Biochemical characterisation of *Tk*MAT: Temperature and pH profile S14

(R) Oligomerisation state of *Sg*PsmC: Size-exclusion chromatography and peak deconvolution S14

(S) Analytical scale reactions of *Sg*PsmC with pyrroloindolines S15

(T) Determination and calculation of E-values S15

(U) Calculation of Chen plots for given E-values S16

(V) Unselective chemical methylation of pyrroloindolines and racemic indolines S16

(W) Calculation of electronic circular dichroism (ECD) spectra and determination of absolute configuration S16
Analytical methods S18
(A) HPLC Program: SAH/SAM S18
(B) HPLC-MS Program: Pyrroloindolines (achiral separation) S18
(C) HPLC Program: Pyrroloindolines (chiral separation) S19
(D) GC-MS Program: Indoline S20
(E) GC Program: 2-Me-indoline and 3-Me-indoline (chiral separation) S21
(F) Nuclear magnetic resonance (NMR) spectroscopy S21
Gene and protein sequences S21
*Sg*PsmC – *N*-Methyl transferase S21
*Tk*MAT – Methionine adenosyl transferase S22
*Ct*HMT – Halide methyl transferase S24
Results S25
DoE-guided expression optimisation of *Sg*PsmC S25
Enzyme purification S36
Additional enzymatic profiling of *Sg*PsmC and oligomeric state S37
Single-substrate Michaelis-Menten kinetics S39
Indoline substrate scope S40
Chiral GC analysis of 2-methyl indoline and 3-methyl indoline substrates. S42
Analysis of the relative enantioselectivity of *Sg*PsmC S43
Chromatographic analysis of pyrroloindolines S46
Precipitation of *Sg*PsmC and optimisation of reaction conditions S73
Discussion on advantages and disadvantages of main SAM (re)generation systems.. S77
Precipitation, pH, and temperature profiles of *Tk*MAT S79
Upscaled kinetic resolution of *rac*-12 .... S81
Chemical syntheses S82
Compound 18 .... S82
Compound *rac*-12 .... S84
Kinetic resolution of *rac*-12 to (*S,S*)-12-Me and (*R,R*)-12 .... S88
Compound *rac*-16 .... S90
Electronic energies, Gibbs free energies, Boltzmann weights, and coordinates of optimised configurations S92
Additional references S167

General considerations and software

(A) General notes

Unless noted otherwise, chemicals and solvents were purchased from Alfa Aesar (Karlsruhe, Germany), Carl Roth (Karlsruhe, Germany), Sigma-Aldrich (Steinheim, Germany), TCI Europe (Zwijndrecht, Belgium), Thermo Fisher Scientific (Schwerte, Germany), and VWR International (Langenfeld, Germany). High-pressure liquid chromatography (HPLC) grade solvents were purchased from Carl Roth (Karlsruhe, Germany). Compounds ***rac*-9** to ***rac*-11**, and compounds ***rac*-13** to ***rac*-15** were synthesized as described previously.[1,2] Chemicals used for synthesis were obtained from commercial sources and were used without further purification. To obtain inert conditions, flame dried glassware and an inert atmosphere (N_2 or Ar) were used. NMR spectra were measured using a Avance DRX 600 (Bruker, Billerica, USA) in deuterated solvents. IR spectra were measured using a Spectrum Two spectrometer (Waltham, USA). High resolution ESI mass spectra were measured by the HHU Center of Molecular and Structural Analytics (Heinrich Heine University, Germany) with a MDS Q Model Trap 4000 mass spectrometer (SCIEX, Toronto, Canada).

(B) Software used

Data were recorded using the software provided by the respective device manufacturer. Data sorting and analysis was done with Microsoft Excel, Origin, and R. Densitometric analysis was carried out with ImageJ. Plots were created using Origin and ggplot2, the latter assisted by ChatGPT.

Table S1: Overview of software used in this work.

Software	Application	Developer / Reference
Design Expert (Version 12.0.7.0)	Design-of-Experiment: planning and analysis	Stat-Ease (Minneapolis, USA)
R and packages (ggplot2, RColorBrewer, ggtext, dplyr, ggrepel, scales, magick, tidyverse, forcats, ggbreak, patchwork, gridExtra, stats, readr, cowplot, biogrowth)	Data analysis and plotting	[3–18]
R-Studio	Scripting	[19]
Microsoft Office	Data analysis and plotting	Microsoft Corporation (Redmond, USA)
Origin 2024b	Data analysis and plotting	OriginLab Corporation (Northampton, USA)
ImageJ (Version 1.8.0_322)	Densitometric analysis	[20,21]
Tecan i-control 2.0	Plate reader control	Tecan (Männedorf, Switzerland)
Unicorn (Version 7.10.0.2756)	Protein purification control	Cytiva (Washington, USA)
INTAS Chemo Star Professional (Version 0.4.33)	Dot blot recording	Intas Science Imaging Instruments (Göttingen, Germany)
SnapGene	Cloning and figures	GSL Biotech LLC (Boston, USA)
ChatGPT	Assistance in plotting with ggplot2 and scripting	OpenAI (San Francisco, USA)

Experimental section and computational methods

(A) Cultivation media and antibiotics

Media were autoclaved before use. TB-medium (terrific broth-medium) was purchased from Carl-Roth (Karlsruhe, Germany), prepared as indicated by the manufacturer, and used for expression cultures. SOC-medium (super optimal broth with catabolic repressor-medium) was used for cell regeneration after chemical transformation. For the SOC-medium, sterile filtered glucose was added after autoclavation. For LB-agar plates (lysogeny broth-agar plates), 1.5% w/v agar-agar was used. Antibiotics were sterile filtered before use. All solutions were prepared with deionised water (dH_2O).

Table S2: Media composition and concentration of antibiotic stocks.

LB medium		
Peptone	10	g/L
Yeast extract	5	g/L
NaCl	10	g/L
Agar-agar (for agar-plates)	1.5	% w/v
TB medium		
Commercial mixture	50.8	g/L
Glycerol	4	mL/L
SOC medium		
Tryptone	2	% w/v
Yeast extract	0.5	% w/v
NaCl	10	mM
KCl	2.5	mM
$MgCl_2$	10	mM
$MgSO_4$	10	mM
Glucose	20	mM
Antibiotics		
Ampicillin (Amp, 1000X stock)	100	mg/mL
Kanamycin (Kan, 1000X stock)	50	mg/mL

(B) Generation of chemically competent cells

Buffers A (100 mM $MgCl_2$) and B (100 mM $CaCl_2$, 15% v/v glycerol) were freshly prepared, sterile filtered, and cooled on ice before use. 2 mL of an overnight pre-culture was used to inoculate 400 mL LB medium which was incubated at 37 °C, 120 rpm. Upon reaching a cell density of OD = 0.4 – 0.6, the culture was centrifuged for 10 min at 4 °C and 1230 *g*. The cell pellet was carefully resuspended in 10 mL of cold Buffer A and kept on ice for 20 – 30 min. The suspension was centrifuged again for 10 min at 4 °C and 1230 *g* and the cell pellet resuspended in 2 mL of cold Buffer B. Aliquots of 50 µL were shock-frozen with liquid nitrogen and stored at -78 °C until further use.

(C) Transformation of chemically competent cells

Chemically competent cells were transformed by heat-shock treatment at 42 °C. Competent cells were thawed on ice and ~50 ng DNA were added to the cells. After a 20 min incubation

on ice, the cells were heat-shocked at 42 °C for 60 sec using a heat-block. For cell recovery, 700 µL of SOC medium without any antibiotics were added to the cells which were then incubated for 1 h at 37 °C while gently shaken. Lastly, cells were plated on LB agar plates with antibiotics and grown overnight at 37 °C.

(D) Preparation of *E. coli* glycerol stocks for cultivations

All cultivations were started with glycerol stocks of the respective transformants. These were prepared by heat-shock transformation with the respective plasmids, overnight incubation on LB-agar plates at 37 °C supplemented with the necessary antibiotic, and inoculation of TB-medium with a single cell colony. A sample of this overnight culture was mixed with sterile glycerol (50% v/v final concentration) and stored at -78 °C until further usage.

Table S3: Overview of *E. coli* strains used in this work.

Strain	Genotype	Application	Ref.
E. coli DH5α	F^- φ80*lac*ZΔM15 Δ(*lac*ZYA-*arg*F)U169 *rec*A1 *end*A1 *hsd*R17(r_K^-, m_K^+) *pho*A *sup*E44 λ^- *thi*-1 *gyr*A96 *rel*A1	Plasmid amplification	[22]
E. coli BL21(DE3)	F^- *omp*T *hsd*S_B (r_B^-, m_B^-) *gal dcm* *λ*(DE3)	Protein expression	Novagen
E. coli BL21 Tuner(DE3)	F^- ompT *hsd*S_B (r_B^- m_B^-) *gal dcm lacY1*(DE3)	Protein expression	Novagen
E. coli BL21 Gold(DE3)	F^- *omp*T *hsd*S(r_B^- m_B^-) *dcm gal* Tet^r λ(DE3) endA Hte	Protein expression	Novagen
E. coli Δmtn(DE3)	F^-, Δ(araD-araB)567, pfs-773(del) ΔlacZ4787(::rrnB-3), λ^-, *rph*-1, Δ(rhaD-rhaB)568, hsdR514, *λ*(DE3)	Protein expression	[23]

Table S4: Overview of plasmids used in this work.

Plasmid	Properties	Ref.
pET21a(+)	*lacI*, Amp^r, T7 promotor, T7 transcription start, T7 terminator, f1 ori, pBR322 ori	Novagen
pET21a(+)::*Sg*PsmC	pET21a(+), *Sg*PsmC (Cter-His_6)	Synthesized by GenScript (Piscataway, USA)
pET21a(+)::*Tk*MAT	pET21a(+), *Tk*MAT (Cter-His_6)	Synthesized by GenScript (Piscataway, USA)
pET28a(+)	*lacI*, Kan^r, T7 promotor, T7 transcription start, T7 terminator, f1 ori, pBR322 ori	Novagen
pET28a(+)::*Ct*HMT	pET28a(+), *Ct*HMT (Nter-His_6)	Kindly provided by Prof. Seebeck (University of Basel)

(E) Non-optimised heterologous expression of *Sg*PsmC

A glycerol stock of *E. coli* BL21(DE3)//pET21a(+)::*Sg*PsmC was used to inoculate TB/Amp medium which was incubated overnight at 37 °C, 130 rpm to prepare the pre-culture. The next day, expression cultures were prepared by inoculation of 500 mL TB/Amp medium in non-baffled 2 L *Fernbach*-flasks at an OD of 0.05. Cultures were grown until reaching an OD of

~0.6 and protein expression was induced by addition of 100 µM IPTG (final concentration, 1000X stock solution). Cultures were incubated at 25 °C, 130 rpm for 16 – 20 h. The cultures were harvested by centrifugation (10,000 *g*, 30 min, 4 °C) and the pellet stored at -20 °C until further use.

(F) Heterologous expression of *Tk*MAT and *Ct*HMT

Expression of *Tk*MAT and *Ct*HMT proceeded following procedure **(E)** with the following adjustments. For *Tk*MAT, the strain *E. coli* BL21 Gold(DE3)//pET21a(+)::*Tk*MAT and the antibiotic ampicillin were used. Protein expression was induced by the addition of 200 µM IPTG (final concentration).

For *Ct*HMT, the strain *E. coli* Δmtn(DE3)//pET28a(+)::*Ct*HMT and the antibiotic kanamycin were used. All media were supplemented with 1% w/v glucose before use. Protein expression was induced by the addition of 100 µM IPTG (final concentration).

(G) Cell disruption

For the preparation of cell-free extracts (CFE) for enzyme reactions, $(NH_4)_2SO_4$ precipitations, or subsequent enzyme purification, harvested cells were disrupted by sonication. Cell pellets were resuspended in their respective resuspension buffers to a final concentration of 0.2 g cells (wet cell weight) per mL buffer. Suspensions were sonicated three times for 10 min each (5 cycles at 10% interval, 35% amplitude, K76 sonotrode; Bandelin Electronic, Berlin). After each 10 min sonication, cell disruption was halted for 3 min to prevent over-heating of the sample. At all times, the cell suspensions were kept on ice. To remove cell debris, lysed cells were centrifuged for 45 min at 4 °C and 20,000 *g*. Lysates were sterile-filtered and used immediately.

(H) Enzyme purification *via* immobilised metal-ion affinity chromatography (IMAC)

All buffers used for the purification of respective enzymes are listed below. CFEs were prepared following procedure **(G)** using respective resuspension buffers. Whenever possible, samples were kept on ice. A prepacked 5 mL Ni-NTA agarose column (Cytiva, Washington, USA) was washed with dH_2O and equilibrated with equilibration buffer before applying previously prepared CFE (prepared from up to 5 g of wet cell mass) onto the column using a peristaltic pump over the course of 30 min. Afterwards, the respective enzymes were eluted using a step- or linear-gradient of varying imidazole concentrations. Elution fractions containing the target protein were combined and concentrated using a 10 kDa cut-off concentrator (Vivaspin 20, Sartorius, Germany) at 4,000 *g*, 4 °C. Buffer was exchanged for the respective storage buffer using a PD10 column (Cytiva). The final enzyme solution was supplemented with 20% v/v glycerol (final concentration), aliquoted, shock-frozen with liquid nitrogen and stored at -20 °C until further use. Purification was assisted by an FPLC system (ÄKTA pure, Cytiva).

Table S5: Overview of buffers used for protein purification.

*Sg*PsmC: Buffers

Buffer	***Composition***
Base buffer	50 mM Tris, 500 mM NaCl, pH 7.5
Elution buffer	50 mM Tris, 500 mM NaCl, 1000 mM imidazole, pH 7.5
Resuspension buffer	Base buffer + 2% v/v elution buffer
Storage buffer	100 mM Tris, 100 mM NaCl, pH 8.0

*Tk*MAT: Buffers

Buffer	***Composition***
Base buffer	40 mM Tris, 100 mM NaCl, pH 8.0
Elution buffer	40 mM Tris, 100 mM NaCl, 1000 mM imidazole, pH 8.0
Resuspension buffer	Base buffer
Storage buffer	Base buffer

*Ct*HMT: Buffers

Buffer	***Composition***
Base buffer	50 mM potassium phosphate, 500 mM NaCl, pH 8.0
Elution buffer	50 mM potassium phosphate, 250 mM imidazole, pH 8.0
Resuspension buffer	Base buffer + 20 mM imidazole
Storage buffer	50 mM potassium phosphate, 20 mM NaCl, pH 7.6

Table S6: Overview of IMAC purification protocols.

*Sg*PsmC: Purification program

Step	***Buffer***	***[Imidazole]***	***Column Volumes***
1	Base buffer + 12% elution buffer	30 mM	12 CV
2	Base buffer + 11.8% elution buffer	118 mM	5 CV
3	Base buffer + 25.6% elution buffer	256 mM	10 CV
4	Elution buffer	1000 mM	5 CV

*Tk*MAT: Purification program

Step	***Buffer***	***[Imidazole]***	***Column Volumes***
1	Base buffer	0 mM	4 CV
2	Base buffer ⇒ base buffer + 25% elution buffer (linear gradient)	0 mM ⇒ 250 mM	10 CV
3	Base buffer + 25% elution buffer	250 mM	5 CV
4	Elution buffer	1000 mM	5 CV

*Ct*HMT: Purification program

Step	***Buffer***	***[Imidazole]***	***Column Volumes***
1	Base buffer + 12% elution buffer	30 mM	5 CV
2	Elution buffer	250 mM	5 CV

(I) Precipitation profiles of *Sg*PsmC and *Tk*MAT

CFEs were prepared following procedure **(G)** using the same resuspension buffer for both enzymes (100 mM Tris, 100 mM NaCl, pH 8.0) and a cell density of 0.33 g cells per mL buffer. A saturated stock solution of $(NH_4)_2SO_4$ was prepared (702.54 g/L) and cooled on ice to 0 °C. Precipitation proceeded in steps of increasing precipitant concentration (10% saturation per

step). All steps were performed on ice. While stirring, the precipitant concentration of the CFE was increased by the drop-wise addition of the saturated $(NH_4)_2SO_4$ solution. The CFE was stirred for 5 min, centrifuged for 10 min at 20,000 *g* and 4 °C, and the supernatant was removed for the next precipitation step. The pellet obtained after each step was resuspended in a small volume of resuspension buffer. The final volume of the 'pellet fraction' was determined, and the sample stored at -20 °C until further usage. The pellet fractions were used for activity determination, and for assessment of protein concentration *via* Bradford assay[24] in order to record the final precipitation profiles. For SDS-PAGE analysis, SDS-PAGE samples of all pellet fractions were prepared by adjusting the $(NH_4)_2SO_4$ content to the same concentration in order to avoid distortion during electrophoretic separation and by adjusting all samples to the same final dilution for direct comparison.

For *Sg*PsmC, activity was determined using indoline (**8**) as a substrate. Analytical scale reactions were performed in reaction buffer (100 mM Tris, 20 mM $MgCl_2$, 200 mM KCl, pH 8.0) at 35 °C and 700 rpm and consisted of 1 mM indoline (from 100X stock in DMSO), 2 mM SAM (from 100X stock in DMSO), and 10% v/v of the respective pellet fraction. Samples were taken at multiple time points and extracted three times with MTBE (supplemented with 1,3,5-trimethoxybenzene as an internal standard). Quantification of product formation was performed by gas chromatography-mass spectrometry (GC-MS).

For *Tk*MAT, SAM formation was measured for activity determination using ATP and L-methionine as substrates. Analytical scale reactions were performed in reaction buffer (100 mM Tris, 20 mM $MgCl_2$, 200 mM KCl, pH 8.0) at 35 °C and 700 rpm and consisted of 2 mM of ATP and 2 mM of L-methionine (each from 100 mM Stock in dH_2O), and 5% v/v of the respective pellet fraction. Samples were taken at multiple time points and quenched by the addition of 2% w/v TCA (final concentration). Quenched samples were centrifuged for 10 min at 20,000 *g* and the supernatant used for reverse-phase high-pressure liquid chromatography (RP-HPLC) analysis.

(J) DoE-guided expression optimisation

<u>Design of Experiment (DoE).</u> For expression optimisation of *Sg*PsmC, a DoE-guided approach was chosen. Optimisation proceeded using a three-factor 5-level central-composite-design with three replicates for each experimental condition, except for the star-point which was replicated five times, leading to a total of 47 experimental runs. The three factors were inducer concentration (IPTG, 0.05 mM to 0.5 mM), cultivation temperature (20 °C to 37 °C), and bacterial culture optical density at induction (OD_{ind}, 0.05 to 5). *E. coli* BL21 Tuner(DE3) was used for expression optimisation, allowing a finely tuned response to varying IPTG concentrations.[25] As the primary response, *Sg*PsmC activity against 5-methyl indoline (**7**) was chosen. Secondary responses of the samples obtained from the same experimental runs were total *Sg*PsmC protein content and soluble *Sg*PsmC protein content. Protein content was assessed by SDS-PAGE and dot-blot (see methods **(L)** and **(M)**) followed by densitometric analysis. The experimental design was facilitated by the software Design Expert (V12.0.7.0; Stat-Ease) which was also used for data analysis.

Runs were blocked by temperature for practical reasons as the microbioreactor (BioLector; m2p-labs) allowed only one cultivation temperature per experiment and in order to increase the throughput of the overall experimental workflow.

<u>Cultivation procedure.</u> Small-scale cultivations were performed on cultivation plates (48-well, clear-bottom FlowerPlates; m2p-labs, Aachen, Germany) allowing for continuous online monitoring of cell-growth using an accordingly equipped microbioreactor. An overnight

preculture was used to inoculate TB/Amp medium to an OD of 0.05. The inoculated medium was distributed on the cultivation plates (1.2 mL per well). Which well corresponded to which experimental run was chosen at random. As controls, empty vector cells and non-induced cells were used on every cultivation plate during every microbioreactor experiment.

Protein expression was induced manually by the addition of varying IPTG concentrations upon cultures reaching their target OD_{ind}. As the bioreactor does not measure OD but rather back-light scatter (which is proportional to bacterial cell density), 'OD-markers' were placed on each cultivation plate, additionally. The 'OD-markers' consisted of dilutions of the *E. coli* BL21 Tuner(DE3) preculture with the ODs at which the actual microcultures were to be induced. This made it easy to determine when the respective cultures reached their respective target OD_{ind} for expression induction.

Cultures were grown until all had reached the stationary phase. The OD at harvest of all cultures was determined in a 96-well plate using a plate reader. Cells were harvested in the cultivation FlowerPlate by centrifugation at 1,000 *g* and 4 °C for 30 min. The supernatant was discarded, the plates sealed and stored at -20 °C until further use. Before use, cells were thawed at room temperature and washed with 0.9% w/v NaCl.

Primary response. *Sg*PsmC activity was determined for each cultivation run using whole cells. Reactions were performed in reaction buffer (100 mM Tris, 20 mM $MgCl_2$, 200 mM KCl, pH 8.0), and consisted of 1 mM 5-methyl indoline (**7**) (from 100X stock in DMSO), 2 mM SAM (from 100X stock in DMSO) and whole cells at a final 'concentration' of OD = 1. Reaction samples were taken after 5 min and quenched with 2% w/v TCA (final concentration). The sample was centrifuged at 20,000 *g* for 15 min at 4 °C and the supernatant analysed by RP-HPLC. In order to minimize the number of samples to analyse, only one single time point was used to assess enzyme activity. To assure this assessment of enzyme activity was reliable, conversion of all reactions was kept below 5%. Furthermore, SAM formation over time was verified to behave linearly over the course of 20 min when using BL21(DE3)//pET21a(+)::*Sg*PsmC cells (expression using non-optimised protocol) at densities of up to OD = 2 (see **Fig. S3**).

Secondary responses. Total and soluble *Sg*PsmC content of respective cultivations was determined by SDS-PAGE-based densitometry using ImageJ.[20,21] A negative control (empty vector cells) and a reference control (0.25 µg/µL purified *Sg*PsmC diluted in CFE from empty vector cells) were run on every gel. SDS-PAGE images were first colour-inverted and then the band intensity was measured. Intensity was normalised to the reference control.

Calculation of space-time-yields. Space-time-yields (STY) of each individual experimental run were calculated from:

$$\mathrm{STY} = \frac{\mathrm{Activity}\left[\frac{\mathrm{mAu\ SAM}}{\mathrm{min}}\right] \cdot \mathrm{OD}_{\mathrm{Stationary\ Phase}}[\mathrm{OD}]}{\mathrm{Time\ until\ stationary\ phase\ [h]}}.$$

To determine the time needed to reach the stationary phase, the *biogrowth*[18] R-package was used.

Scaled-up expression. Three cultivations conditions yielding the highest, lowest, and a mediocre primary response, respectively, were scaled-up from 1.2 mL to 500 mL culture volume. Cells were harvested 16 h after induction and *Sg*PsmC activity measured as described above.

Table S7: Overview of DoE conditions tested in upscaled cultivations.

Condition	IPTG	OD at induction	Cultivation Temperature
'Best'	104 µM	4.406	22.0 °C
'Mediocre'	50 µM	2.525	28.5 °C
'Worst'	275 µM	2.525	37.0 °C
'Standard'	100 µM	0.6	Growth at 37.0 °C<br>Expression at 25 °C

(K) Bradford assay

A calibration curve using a bovine serum albumin dilution series was prepared in triplicate. 180 µL of the Bradford[24] reagent were mixed with 20 µL of protein solution and incubated for 5 min at room temperature, before measuring absorbance at 595 nm using a plate reader (infinite M1000 pro, Tecan) The Bradford reagent contained 100 mg/L Coomassie G-250, 5% v/v EtOH, and 10% v/v phosphoric acid.

(L) Sodium dodecyl sulphate-polyacrylamide gel electrophoresis (SDS-PAGE)[26]

Analysis of protein samples was done by SDS-PAGE. The composition of used solutions is noted below (**Table S8**). Samples were diluted with 5X SDS sample buffer and heated for 10 min at 95 °C. Gels were run at 180 V for ~50 min, washed with dH_2O and stained in Coomassie solution overnight while shaking. Destaining was done with dH_2O overnight while shaking. For documentation, gels were photographed on a white light table with a Canon EOS 1000D digital camera (ISO 400, 1/250 s^{-1} shutter speed, *f*/8.0 aperture, manual white balance; Canon Deutschland, Germany).

Table S8: Overview of solutions used for SDS-PAGE.

10X Anode buffer, pH 8.9		
Tris	1	M
HCl	0.225	M
10X Cathode buffer, pH 8.25		
Tris	1	M
Tricine	1	M
SDS	1	% w/v
3X Gel buffer, pH 8.45		
Tris	3	M
HCl	1	M
SDS	0.3	% w/v
Coomassie solution		
EtOH	30	% v/v
Anhydrous acetic acid	0.5	% v/v
Coomassie brilliant blue G.250	0.5	% w/v
Preparation of stacking gel (4% acrylamide/bisacrylamide)		
30% Acrylamide/Bisacrylamide solution*	0.4	mL
3X Gel buffer	0.75	mL
dH_2O	1.85	mL
TEMED	2.5	µL
APS solution (10% w/v in dH_2O)	25	µL
5X SDS sample buffer, pH 6.8		
Tris-HCl	0.25	M
SDS	8	% w/v
β-mercaptoethanol	10	% v/v
Dithiothreitol	0.3	M
Glycerol	30	% v/v
Bromophenol blue	0.02	% w/v

(M) Determination of *Sg*PsmC protein content using dot-blot

As an alternative method for determining the *Sg*PsmC content of different cultivations, dot-blot analysis was considered. Samples were prepared by dilution to OD = 4 and addition of dye-free 5X SDS-PAGE sample buffer. Samples were heated at 98 °C for 10 min and then applied onto a nitrocellulose membrane (pore size 0.2 µm, 2 µL sample per dot). After letting the membrane dry for at least 5 min, the following steps were applied at room temperature: three washing steps (TBS-T buffer, 5 min, while shaking), one blocking step (TBS-T buffer with 2.5% w/v skim milk powder, 1 h), three washing steps (TBT-T buffer, 5 min, shaking), antibody incubation (antibody solution, 1 h), three washing steps (TBS-T buffer, 5 min, while shaking). For chemiluminescence-based detection, the membrane was wetted with the detection solution. The resulting chemiluminescence signal was recorded using an INTAS Advanced Fluorescence imaging system (Intas Science Imaging Instruments).

Table S9: Overview of solutions used for dot-blots.

Solution	*Component / Stock solution*	*Concentration*
TBS-T buffer (pH 8.0)	Tris	25 mM
	NaCl	140 mM
	KCl	3 mM
Blocking solution (in TBS-T)	Tween-20	0.2 % v/v
	Skim milk powder (blotting grade)	2.5% w/v
Antibody solution (in TBS-T)	Anti-His-Tag antibody (0.5 mg/mL in dH_2O) [THE™ His-Tag mouse antibody conjugated to horseradish peroxidase (GenScript)]	1:2000
Detection solution	1.4 mM Luminol (in 100 mM Tris, pH 8.6)	1.3 mM
	6.7 mM *p*-hydroxycoumaric acid (PCA) in DMSO	0.6 mM
	30% v/v H_2O_2	0.01% v/v
5X SDS-sample buffer, dye-free (pH 6.8)	Tris	0.25 M
	SDS	8% w/v
	β-mercaptoethanol	10% v/v
	dithiothreitol (DTT)	0.3 M
	glycerol	30% v/v

(N) Biochemical characterisation: General procedure for analytical-scale enzyme reactions

Enzyme reactions and subsequent analysis were generally performed as follows. Deviations are explicitly noted. Reactions were carried out in a heating-block at 700 rpm in 1.5 mL or 2 mL plastic reaction tubes. Reaction samples were taken at specified time points and quenched by the addition of 10% v/v TCA (2% v/v final concentration). Reaction samples were centrifuged at 20,000 *g*, 4 °C for 15 min, and the supernatant was used for subsequent analysis by RP-HPLC for the quantification of the formed SAH or SAM. Reactions were performed in triplicate. In order to minimize the number of reaction samples, often only one single time point was used to assess enzyme activity. To assure this assessment of enzyme activity was reliable, conversion of all reactions was kept below 5% and the linear behaviour of conversion against reaction time was verified beforehand.

For *Sg*PsmC, the standard reaction conditions were as follows: 35 °C, 700 rpm; buffer: 100 mM Tris, 20 mM $MgCl_2$, 200 mM KCl, pH 8.0; 1 mM 5-methyl indoline (**7**) (from 100X DMSO-stock), 2 mM SAM (from 100X DMSO-stock), 1 µM purified *Sg*PsmC. Reaction samples were generally taken after 10 min reaction time. Relative activity was calculated by normalisation of the SAH peak area to the SAH peak area of the sample with the highest concentration of formed SAH.

For *Tk*MAT, the standard reaction conditions were as follows: 35 °C or 50 °C, 700 rpm; buffer: 100 mM Tris, 20 mM $MgCl_2$, 200 mM KCl, pH 8.0; 2 mM ATP (from 100X dH_2O-stock), 2 mM L-methionine (from 100X dH_2O-stock), 4.36 µM purified *Tk*MAT. Relative activity was calculated by normalisation of the SAM peak area to the SAM peak area of the sample with the highest concentration of formed SAM.

(O) Biochemical characterisation of *Sg*PsmC: Procedures for enzyme profiling

pH Profile. Reactions were performed as described under general procedure **(N)** with the following additions. As buffer, a modified Britton-Robinson universal buffer (40 mM boric acid, 40 mM phosphoric acid, 40 mM acetic acid) was used,[27] enabling decoupling of activity effects

ascribed to buffering components from effects stemming from changes in pH. The pH was adjusted with NaOH between pH 2.5 and pH 12. Prior to the reaction, the buffer in which *Sg*PsmC was stored in was exchanged against 3 mM potassium phosphate, pH 7 buffer by ultrafiltration (repeated three times, each 10 min at 10,000 *g*, 4 °C; Vivaspin 500, 10 kDa cut-off; Sartorius). Reactions were performed in a 96 well-plate in a total volume of 200 µL.

Temperature profile. Reactions were carried out in PCR-tubes in a PCR-cycler with a temperature-gradient functionality (XT96, VWR, Radnor, USA). Temperatures between 20 °C and 60 °C were tested. Controls only with SAM were run at 20 °C and 60 °C. Reaction samples were taken after 10 min.

Melting temperature. An aliquot of *Sg*PsmC was pre-incubated for 15 min at temperatures between 30 °C and 70 °C using a PCR-cycler with a temperature-gradient functionality (XT96, VWR). Then, a standard reaction was performed at 30 °C. Reactions were performed in preheated buffer in 96-well plates. Relative activities were fitted to the following logistic function:

$$y = \frac{A_{sym}}{1 + \exp\frac{x_{mid} - x}{s_{cal}}}$$

leading to the following estimated parameters and their standard error, where y is the relative activity in [%], and x_{mid} corresponds to the melting temperature T_M in [°C]: A_{sym} = 98.38 ± 4.98%, x_{mid} = 43.34 ± 0.63 °C, s_{cal} = -2.74 ± 0.52 °C.

Time stability. *Sg*PsmC was incubated under standard reaction conditions without any substrates present. At fixed time points, an aliquot was removed and used to perform a standard reaction. Relative activities were fitted to the following exponential decay function:

$$y = a \cdot \exp(-b \cdot t) + c$$

where y is the relative activity in [%], leading to the following estimated parameters and their standard error: a = 90.47 ± 3.08%, b = 0.22 ± 0.02 h^{-1}, c = 1.28 ± 2.11%. Half-life was determined by:

$$t_{1/2} = \frac{\ln 2}{b}$$

Solvent tolerance. The SAM stock was prepared in reaction buffer instead of DMSO in order to keep the additional DMSO as low as possible. Different buffer/solvent-mixtures were prepared, in which *Sg*PsmC was incubated for 0 h or 1 h prior to starting the reaction by addition of substrates. Reactions were performed in preheated reaction medium in 96-well plates.

(P) Kinetic analysis of *Sg*PsmC

For single-substrate Michaelis-Menten kinetics, a commercial assay kit (MTase-Glo™, Promega, Fitchburg, USA) was used according to the manufacturer's instructions. First, the optimal enzyme concentration for the assay was determined. Enzyme concentration was tested in the range of 0.01 µg to 7 µg enzyme per reaction (20 µL reaction volume). 10 µL of the respective enzyme solution was placed in a 96-well plate (F-bottom, white, non-transparent) and the reaction was started by addition of 2X substrate solution (50 µM SAM, 50 µM methyl acceptor, in 1X reaction buffer). Reactions were performed at 40 °C and stopped after 10 min by addition of 5 µL TFA (0.5% v/v stock solution). 5 µL of the supplied 5X

MTase-Glo™ Regent solution were added and the mixture incubated for 30 min at room temperature. Then, 25 µL of the supplied detection solution were added and the resulting luminescence measured after a further 30 min incubation at room temperature.

For kinetic analysis, 10 µL of enzyme solution (0.01 µg *Sg*PsmC for assaying the natural substrate ***(S,S)*-9**, 2 µg *Sg*PsmC when assaying indoline (**8**)) were placed in a 96-well plate (F-bottom, white, non-transparent). The reaction was started by addition of 10 µL of 2X substrate solution (50 µM SAM, 1 – 200 µM methyl acceptor, in 1X reaction buffer) and performed at 40 °C. Reactions were stopped after 5 – 10 min (three time points) by the addition of 5 µL TFA (0.5% v/v stock solution). 5 µL of the supplied 6X MTase-Glo™ Regent were added to the quenched reaction and incubated for 30 min at room temperature. Then, 25 µL of the supplied detection solution were added and the resulting luminescence measured after further 30 min incubation at room temperature. Enzyme activity was determined by linear regression over the three reaction time points. Enzyme activity was in turn used for non-linear regression to fit the standard Michaelis-Menten kinetic model to the obtained data:

$$v_0 = \frac{v_{max}[S]}{K_M[S]}$$

where v_0 is the initial reaction rate, v_{max} the maximum reaction rate, [S] the substrate concentration, and K_M the Michaelis-Menten constant.

5X Reaction buffer	100 mM Tris pH, 8.0, 100 mM NaCl, 1 mM DTT, 1 mg/mL BSA

(Q) Biochemical characterisation of *Tk*MAT: Temperature and pH profile

pH Profile. The effect of the pH value of the reaction medium on *Tk*MAT activity was assessed with a set of standard reactions performed at varying pH (pH 7.0 – 9.0, 0.5 pH step increment). The reaction was performed at 35 °C, and stopped after 25 min. Reactions were performed in 100 mM Tris, 20 mM $MgCl_2$, 200 mM KCl.

Temperature profile. For the determination of the temperature profile of *Tk*MAT, a set of standard reactions were performed at varying temperatures (30 – 90 °C, 10 °C step increment). Samples were taken after 5 min and prepared for RP-HPLC analysis. Relative enzyme activity was calculated independently for each set of reactions stopped at different times. Formation of 5'-methyl-thio-5'-desoxyadenosine (MTA) was also interpreted as product formation in addition to SAM.[28]

(R) Oligomerisation state of *Sg*PsmC: Size-exclusion chromatography and peak deconvolution

Size exclusion chromatography was carried out on a HiLoad 16/600 pg 200 column (column volume 120.6 mL, Cytiva) using an FPLC system (ÄKTA pure, Cytiva). The column was first washed with 4 column volumes (CV) of dH_2O at a flow rate of 1 mL/min and then equilibrated with 2 CV buffer (10 mM Tris, 100 mM NaCl at pH 7.5) at a flow rate of 1.6 mL/min. IMAC-purified protein was thawed on ice and concentrated up to 1 mL using a 10 kDa cut-off concentrator (Vivaspin 500, Sartorius) at 4,000 *g* and 4 °C. The protein was applied *via* a 1 mL sample loop. For elution, buffer at a flow rate of 1 mL/min was used and the run was stopped after 1.5 CV. The calibration of the FPLC system was carried out using a gel filtration marker kit with proteins of known molecular weights between 12 kDa and 200 kDa (Sigma Aldrich, St. Louis, USA). Peak deconvolution was performed using the 'Peak Analyzer' tool from the Origin 2024b software package (OriginLab), with the following parameters.

Table S10: Settings used for SEC-peak deconvolution using Origin 2024b.

Baseline Mode:	constant, minimum	
Baseline Treatment:	Defaults	
Find Peaks:	*Direction:*	Both
	Method:	2nd derivative
	Smooth derivative – Method:	Quadratic-Savitzky-Golay
	Smooth derivative – Polynomial order:	2
	Smooth derivative – Points of window:	500
Peak Filtering:	*Method:*	By number
	Number of peaks:	4

(S) Analytical scale reactions of *Sg*PsmC with pyrroloindolines

Reactions were performed as described under procedure **(N)** with the following changes. After quenching with TCA, half the sample was analysed with RP-HPLC to determine conversion *via* depletion of the pyrroloindoline and/or the formation of SAH. The other half of the sample was extracted three times with *n*-heptane. In the case of ***rac*-9**, reactions were extracted with EtOAc. Solvent was removed using a vacuum concentrator (SpeedVac, Eppendorf, Hamburg, Germany) and the sample redissolved in a solvent mixture matching the starting conditions of the respective NP-HPLC program. Analysis with chiral NP-HPLC yielded the *ee* value of the substrate and/or the *N*-methylated product.

(T) Determination and calculation of E-values

E-values were calculated by determining pairs of ee_S and ee_P of reaction samples using either chiral GC (for the substrates 2-Me-indoline and 3-Me-indoline) or chiral normal-phase high-pressure liquid chromatography (NP-HPLC; for the pyrroloindoline substrates) as described by Rakels *et al.*[29] Equation 1 can be rearranged to Equation 2 which was used for non-linear regression to determine the respective E-value.

In cases in which *Sg*PsmC exhibited very high enantioselectivity, not all data pairs of ee_P and ee_S could be used for fitting. Due to the high enantioselectivity of *Sg*PsmC and the limited sensitivity of the analytical methods, data pairs with one apparent *ee* value of 100% were determined which cannot be used for fitting. Setting these *ee* values to 98% yields E-values >100 using Equation 1. As precision is lowest when measuring very high *ee* values, E-values greater than 100 are not reported in the main text.[30]

The experimental data and the fitted function were visualised in Rakels plots.[29] If the used substrate consisted of a racemate, the extent of conversion ξ can be calculated using Equation 3,[31] allowing visualisation of ee_P *vs* conversion or ee_S *vs* conversion in Chen plots.[32]

In cases where either ee_S or ee_P could not be determined, conversion was measured by achiral RP-HPLC to calculate the E-value using Equation 4 or Equation 5, by fitting conversion(ee_S, E) or conversion(ee_P, E), respectively, to the recorded data set.

$$\mathrm{E} = \ln\left[\frac{1-\mathrm{ee_S}}{1+\frac{\mathrm{ee_S}}{\mathrm{ee_P}}}\right] \Big/ \ln\left[\frac{1+\mathrm{ee_S}}{1+\frac{\mathrm{ee_S}}{\mathrm{ee_P}}}\right] \qquad \text{Equation 1}$$

$$\mathrm{ee_p} = \frac{\mathrm{ee_S}}{\left[\frac{(1+\mathrm{ee_P})^{E}}{1-\mathrm{ee_S}}\right]^{\frac{1}{E-1}} - 1} \qquad \text{Equation 2}$$

$$\xi = \frac{\mathrm{ee_S}}{\mathrm{ee_S} + \mathrm{ee_P}} \qquad \text{Equation 3}$$

$$\mathrm{E} = \frac{\ln[(1-\mathrm{conv})\cdot(1-\mathrm{ee_S})]}{\ln[(1-\mathrm{conv})\cdot(1+\mathrm{ee_S})]} \qquad \text{Equation 4}$$

$$\mathrm{E} = \frac{\ln[1-\mathrm{conv}\cdot(1+\mathrm{ee_P})]}{\ln[1-\mathrm{conv}\cdot(1-\mathrm{ee_P})]} \qquad \text{Equation 5}$$

(U) Calculation of Chen plots for given E-values

For a given E-value, Chen plots were calculated by solving Equations 4 and 5 numerically by searching for the root of $f(\mathrm{ee_S})$ and of $f(\mathrm{ee_P})$. Missing values were calculated using Equation 3.

(V) Unselective chemical methylation of pyrroloindolines and racemic indolines

To quickly obtain methylated references of the racemic pyrroloindolines for chiral HPLC, substrates (1 mM) were incubated with either CH_3I or MeOTs (each 10 mM) in reaction buffer (100 mM Tris, 20 mM $MgCl_2$, 200 mM KCl, pH 8.0) for 6 h at 35 °C. Samples were extracted using *n*-heptane and prepared for analysis by chiral HPLC. Product formation was further verified by achiral LC-MS and by the shift of the UV/Vis spectrum of *N*-methylated products towards higher wavelengths.

In cases where conversion was low, the following alternative protocol was used. In an argon-flooded 2 mL glass screw-cap vial, 186 µL of dried THF was placed, to which 10 µL educt (1 equiv; from a 100 mM DMSO-stock) was added, followed by the addition of Et_3N (6 equiv, 2 µL). The solution was shaken for 30 min at 35 °C. Then, MeI was added (32 equiv, 2 µL) to the solution and shaken for 16 h to 24 h at 35 °C. The reaction was quenched using a saturated NH_4Cl solution and extracted with EtOAc. Solvent was removed using a vacuum concentrator (SpeedVac) and the sample redissolved in a solvent mixture matching the starting conditions of the respective NP-HPLC program.

For the racemic indolines 2-methyl indoline and 3-methyl indoline, the substrates (0.5 mM) were incubated with MeOTs (2 mM) in reaction buffer for 16 h at 35 °C. Samples were extracted using EtOAc and prepared for analysis by chiral GC.

(W) Calculation of electronic circular dichroism (ECD) spectra and determination of absolute configuration

The Conformer Rotamer Ensemble Sampling Tool (CREST version 3.0.1)[33] was used for conformational sampling. Structural refinement and energetic sorting of the conformer ensemble was performed using the command-line energetic sorting tool (CENSO version 2.1.2) using r^{2}SCAN-3c.[34] To verify that each structure was a minimum on the potential energy surface, a vibrational analysis was done. Thermal and entropic corrections were obtained from unscaled vibrational frequencies for a standard state of 1 mol L^{-1} and 298.15 K, as

implemented in ORCA (version 5.0.4).[35] The ensemble was manually searched for duplicates and these were then removed. Boltzmann-weights of each conformer were calculated from electronic energies obtained from DFT-calculations using the range-separated hybrid density functional ωB97M-V, quadruple-ζ basis set def2-QZVPP and C-PCM solvation model for hexane.[36–38] For each conformer, ECD-spectra were obtained from TD-DFT calculations using the range separated hybrid functional ωB97X-D4, triple-ζ basis set def2-TZVPP, C-PCM solvation model for hexane[38] and employing the Tamm-Dancoff approximation.[38–40] The ECD-spectra of the conformers were generated by convolution of Gaussian shaped lines with a line width of 2000 cm^{-1} and were summed based on their Boltzmann-weight to the final ECD-spectrum and red-shifted so that their strongest signal matched the maximum absorption peak of experimental UV spectra. ECD spectra were calculated for all (*S,S*)-configured pyrroloindoline substrates converted by *Sg*PsmC and for the (*S,S*)-configured *N*-methylated product **14-Me**.

Racemic pyrroloindoline substrates were separated as described below using chiral NP-HPLC coupled additionally to a CD-2095 detector (Jasco, Tokyo, Japan). The HPLC-CD trace was recorded at 242 nm which is close to the second absorbance peak of all substrates and for which the (*S,S*)-configured scaffold of all substrates showed a CD signal in the predicted ECD spectra. By comparison of the polarity of the peaks in the HPLC-CD trace with the predicted ECD spectra and with UV-chromatograms of previously recorded *Sg*PsmC reactions (using racemic substrates), the absolute configuration of the preferred substrate enantiomer of *Sg*PsmC could be deduced. Experimental ECD spectra were recorded in the same manner in stop-flow mode. For further details, see **Fig. S21** through **S28**.

Analytical methods

(A) HPLC Program: SAH/SAM

Separation of SAM and SAH was performed by chiral RP-HPLC on a Jasco chromatography system with the following configuration and conditions.

Table S11: Configuration of RP-HPLC.

Pump	PU-2080 Plus
Ternary gradient unit	LG-2080-02S
3-line degasser	16-2080-53
Autosampler	AS-2057 Plus
Column thermostat	CP-2060
Multiwavelength detector	MD-2010

Table S12: Separation method for quantification of SAH.

Column:	Hyperclone ODS (C18-column) (120 mm x 4 mm, 5 µm; Phenomenex, Torrance, USA)		
Flow rate:	1 mL/min		
Detection:	UV 260 nm		
Injection volume:	10 µL		
Column temperature:	25 °C		
Mobile phase:	Eluent A: acetonitrile		
	Eluent B: 5 mM NaH_2PO_4, 5 mM heptane sulfonic acid, pH 3.75		
Program:	0 min ⇒ 5 min:	5% A	
	5 min ⇒ 17 min:	5% A	⇒ 17% A
	17 min ⇒ 20 min:	5% A	

(B) HPLC-MS Program: Pyrroloindolines (achiral separation)

Separation of pyrroloindoline substrates and products for determination of reaction conversion was performed by achiral RP-HPLC on a Dionex UltiMate 3000 system (Thermo Fisher Scientific) coupled to a ISQ EM mass spectrometer (ThermoScientific) with the following configuration and conditions.

Table S13: Configuration of LC-MS.

Pump	UltiMate 3000 Pump
Autosampler	UltiMate 3000 Autosampler
Column thermostat	UltiMate 3000 Column Compartment
Multiwavelength detector	UltiMate 3000 Diode Array Detector

Table S14: Separation method for pyrroloindolines.

Column:	Hyperclone ODS (C18-column) (120 mm x 4 mm, 5 µm; Phenomenex)
Flow rate:	1.2 mL/min
Detection:	UV 205 nm and 254 nm
Injection volume:	4 µL
Column temperature:	45 °C
Mobile phase:	Eluent C: acetonitrile + 0.1% v/v formic acid<br>Eluent D: dH_2O + 0.1% v/v formic acid
Program:	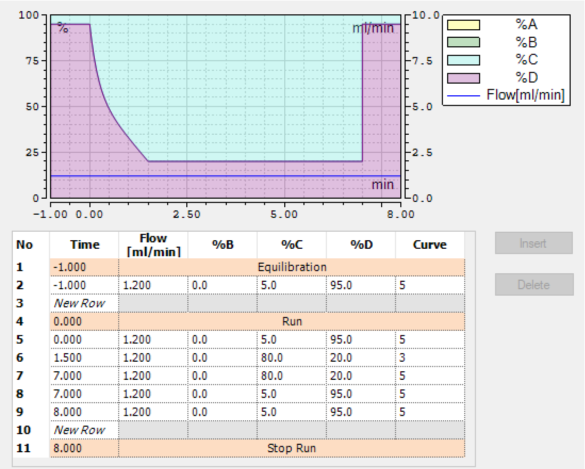

No	Time	Flow [ml/min]	%B	%C	%D	Curve
1	-1.000	Equilibration				
2	-1.000	1.200	0.0	5.0	95.0	5
3	*New Row*					
4	0.000	Run				
5	0.000	1.200	0.0	5.0	95.0	5
6	1.500	1.200	0.0	80.0	20.0	3
7	7.000	1.200	0.0	80.0	20.0	5
8	7.000	1.200	0.0	5.0	95.0	5
9	8.000	1.200	0.0	5.0	95.0	5
10	*New Row*					
11	8.000	Stop Run				

Table S15: Retention times of pyrroloindolines and *N*-methylated products.

Compound	***R_t (N-H)***	***R_t (N-Me)***
***rac*-9**	2.05 min	2.20 min
***rac*-10**	2.45 min	2.75 min
***rac*-11**	2.42 min	*n.d.*
***rac*-12**	2.92 min	2.95 min
***rac*-13**	3.14 min	4.28 min
***rac*-14**	3.89 min	~5.33 min
***rac*-15**	1.97 min	2.13 min
***rac*-16**	2.62 min	2.91 min

(C) HPLC Program: Pyrroloindolines (chiral separation)

Separation of pyrroloindoline substrate and product enantiomers was performed by chiral NP-HPLC on a Dionex UltiMate 3000 system (Thermo Fisher Scientific) with the following configuration conditions.

Table S16: Configuration of chiral HPLC.

Pump	UltiMate 3000 Pump
Autosampler	UltiMate 3000 Autosampler
Column thermostat	UltiMate 3000 Column Compartment
Multiwavelength detector	UltiMate 3000 Diode Array Detector

Table S17: Method parameters common for all separated pyrroloindolines.

Column:	Chiralcel OD-H (250 mm x 4.6 mm, 5 µm; Daicel, Japan) *or*<br>Lux Amylose-1 (250 mm x 4.6 mm, 5 µm; Phenomenex)
Detection:	UV 205 nm and 297 nm
Injection volume:	10 µL
Column temperature:	25 °C

Table S18: Specific separation conditions and retentions times for pyrroloindolines and *N*-methylated products.

Compound	***Column***	***Flow***	***n-Heptane / iPrOH***	***R_t***
***rac*-9**	Chiralcel OD-H	0.5 mL/min	80:20	*N*-H: ~36 min, ~65 min<br>*N*-Me: ~32 min, ~46 min
***rac*-10**	Chiralcel OD-H	0.5 mL/min	95:5	*N*-H: ~37 min, ~57 min<br>*N*-Me: ~23 min, ~28 min
***rac*-11**	Lux Amylose-1	1.0 mL/min	90:10	*N*-H: ~25 min, ~53 min<br>*N*-Me: *n.d.*
***rac*-12**	Chiralcel OD-H	0.5 mL/min	95:5	*N*-H: ~46 min, ~56 min<br>*N*-Me: ~21 min, ~25 min
***rac*-13**	Chiralcel OD-H	0.5 mL/min	90:10	*N*-H: ~29 min, ~46 min<br>*N*-Me: ~20 min
***rac*-14**	Chiralcel OD-H	0.5 mL/min	90:10	*N*-H: ~20 min, ~40 min<br>*N*-Me: ~15 min
***rac*-15**	*could not be separated*			
***rac*-16**	Chiralcel OD-H	0.5 mL/min	50:50	*N*-H: ~29 min, ~33 min<br>*N*-Me: ~15 min, ~18 min

(D) GC-MS Program: Indoline

GC-MS was used as an alternative method to determine *Sg*PsmC activity using indoline as a methyl acceptor. A Trace 1310 GC System (Thermo Fisher Scientific) was used, coupled with an ISQ QD Single Quadrupole MS System (Thermo Fisher Scientific), using the following conditions.

Table S19: Configuration of GC-MS and used temperature gradient.

Column:	OPTIMA 5 MS (30 m x 0.25 mm; film thickness: 0.25 µm;<br>Macharey-Nagel, Düren, Germany)
Carrier:	He
Gas flow:	1 mL/min
Injection volume:	1 µL
Ionisation:	EI, 70 eV
Program:	60 °C for 1 min – 15 °C/min to 185 °C – 120 °C/min to 280 °C –<br>280 °C for 5 min
Temperature zones:	Injector: 250 °C<br>Transfer line: 280 °C<br>Ion source: 230 °C

(E) GC Program: 2-Me-indoline and 3-Me-indoline (chiral separation)

Chiral GC was used for chiral separation of racemic substrates 2-Me-indoline and 3-Me-indoline and respective *N*-methylated products. A Trace GC (Thermo Finnigan) and a Trace GC Ultra (Thermo Scientific) were used together with a TRIPLUS AS autosampler (Thermo Fisher). Columns were purchased from Macherey-Nagel (Düren, Germany).

Table S20: Configuration of chiral GC and used temperature gradient.

Carrier:	H_2	
Carrier pressure:	60 kPa	
Injection volume:	1 µL	
Injector:	210 °C	
Split flow:	25 mL/min	
Detector:	FID; Base temperature: 220 °C	
Program:	60 °C for 5 min – 5 °C/min to 200 °C – 200 °C for 5 min	
Column:	For 2-Me-indoline:	FS-HYDRODEX β-TBDAc
	For 3-Me-indoline:	FS- HYDRODEX β-3P

Table S21: Specific separation conditions and retentions times for 2-Me-indoline and 3-Me-indoline and the respective *N*-methylated products.

Compound	***Column***	***R_t***
2-Me-indoline	FS-HYDRODEX β-TBDAc	*N*-H: 22.02 min, 22.35 min<br>*N*-Me: 18.05 min, 18.33 min
3-Me-indoline	FS-HYDRODEX β-3P	*N*-H: 23.03 min, 23.29 min<br>*N*-Me: 20.31 min, 20.48 min

(F) Nuclear magnetic resonance (NMR) spectroscopy

NMR spectra were recorded on an Avance/DRX 600 NMR spectrometer (Bruker, Billerica, USA) at 151 MHz (^{13}C) and at 600 MHz (^{1}H) at 25 °C using deuterated solvents.

Gene and protein sequences

Extinction coefficients and protein molecular weight used for determination of protein concentration were predicted using ProtParam.[41] Genes of *Tk*MAT and *Sg*PsmC were obtained from GenScript (Piscataway, USA), the former incorporated in the pET21a(+) vector. The latter was initially obtained as a pUC57 construct and subcloned into pET21a(+) by standard restriction-ligation cloning. The pET28a(+)::*Ct*HMT plasmid was kindly provided by Prof. Seebeck (University of Basel). *Tk*MAT had a *C*-terminal His_6-tag, *Ct*HMT had a *C*-terminal His_6-tag and TEV-site, *Sg*PsmC had an *N*-terminal T7-tag and a *C*-terminal His_6-tag.

_Sg_PsmC – _N_-Methyl transferase

- Organism of origin: *Streptomyces griseofuscus*
- UniProt: W8Q892
- GenBank: KF201694.1:2060..2845, AHL44341.1
- ε_{280} (Cys-bridges) = 48025 M^{-1} cm^{-1}
- ε_{280} (Cys reduced) = 47900 M^{-1} cm^{-1}
- MW = 30163.79 Da

DNA Sequence:
ATGGCAGACGACGCGCACGCCCCGATGTACGGGGCCATCGCTGAGATCTACGACCGACTGGATGAC
TGGATCGTCGCCACCTGGCACGAGCAGCCGGTCCCCGAACGGGTGGCCTTCCTGCGCAAGCGGTGG
GAGCAGCGCGACGGCGAGGTGCGGGACGTGCTGGACCTGTGCTGCGGCACCGGCAGCGTGCTGCAT
GAGCTCAAAGGGGCCGGCTATGAGGTCACCGGGCTCGACCAGTCCCCGCAGATGCTCGGCCTCGCC
CGGCAGCGCCTCGGTGACGGAACCGCACTGATCCAGGCGCAACTGCCCGACATCCCGGTGCCCGAC
GCCGCCATGGACGCGGTGTGTTCCACCGGCGCGGCGCTGAGCTACACCCCCGGTGAGGCAGCGCTG
GCCCAGATCCTCCTGGCCGTGCACCGCGTCCTGCGCCCCGGCGGCGTCTTCGTGTTCGACGTCCTG
TCCCGCCACATGATCACCGAACACGCGGGCCGGCACGTATGGGCCGCCGATCAGGGCGACTTCGCC
TTCATCTGGGAGTTCACCAACCCCAGCGAGAAGTACAGCGAAGCCTTCTACACGCAGTTCCTCCGG
GAGGGCGGACCGGATTCCGCCAGGTACGTACGCACCAGCGAGAAGCATCGCCTGTACGTCCTCGAC
CACTCCCCGGTGCGGCGCGCCGCCGAGCGGGCCGGCTTCACCACCGCAGAGGTGCTCGACAACTAC
ACCGACACCCCCGCCCACGAGAGCACGCTCTACGACACGTGGGTCCTGACCCGCGCCCCTAGGCAC
CACCATCACCATCACTGA

Protein Sequence:
MADDAHAPMYGAIAEIYDRLDDWIVATWHEQPVPERVAFLRKRWEQRDGEVRDVLDLCCGTGSVLH
ELKGAGYEVTGLDQSPQMLGLARQRLGDGTALIQAQLPDIPVPDAAMDAVCSTGAALSYTPGEAAL
AQILLAVHRVLRPGGVFVFDVLSRHMITEHAGRHVWAADQGDFAFIWEFTNPSEKYSEAFYTQFLR
EGGPDSARYVRTSEKHRLYVLDHSPVRRAAERAGFTTAEVLDNYTDTPAHESTLYDTWVLTRAPRH
HHHHH

Plasmid Map:

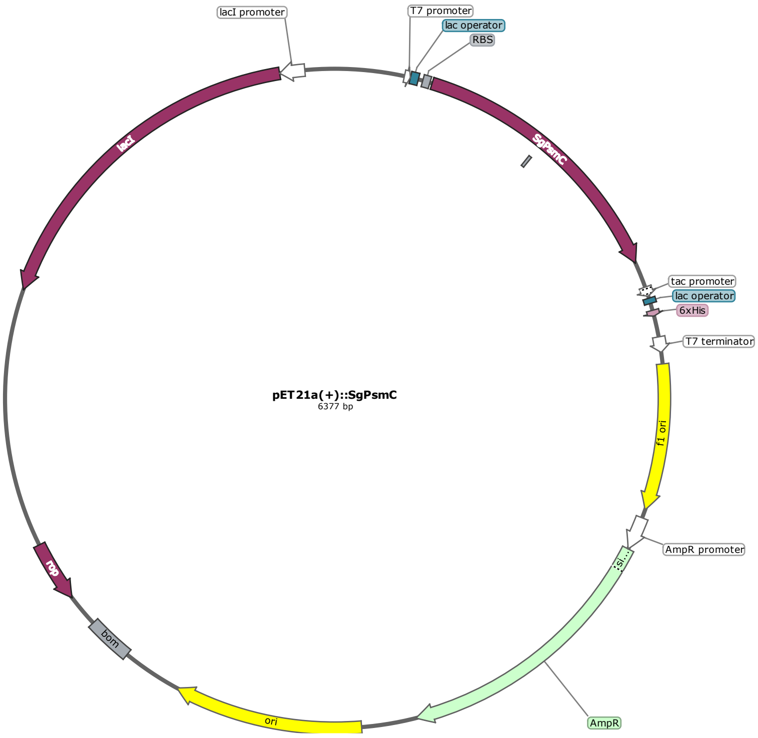

***Tk*MAT – Methionine adenosyl transferase**

- Organism of origin: *Thermococcus kodakarensis*
- UniProt: Q5JF22
- GenBank: NC_006624.1:465352..466569, WP_011249500.1
- ε_{280} (Cys-bridges) = 30830 M^{-1} cm^{-1}
- ε_{280} (Cys reduced) = 30830 M^{-1} cm^{-1}
- MW = 45680.16 Da

Gene Sequence:

ATGGCTGGCAAAGTTCGTAATATTGTTGTTGAAGAACTGGTTCGTACACCTGTTGAAATGCAAAAA
GTTGAACTGGTTGAACGTAAAGGGATCGGGCATCCGGATAGCATTGCAGATGGGATTGCAGAAGCA
GTTAGCCGTGCTCTGTCCCGTGAATATGTGAAACGTTATGGTATCATACTGCATCATAATACAGAT
CAAGTTGAAGTTGTTGGTGGCCGTGCATATCCGCAATTTGGTGGTGGGGAAGTTATCAAACCGATC
TATATCCTGCTGTCCGGCCGTGCAGTTGAAATGGTTGATCGTGAATTTTTTCCGGTTCACGAAATT
GCTTTAAAAGCAGCTAAAGATTATCTGCGTAAAGCAGTTCGTCATCTGGATCTGGAACATCATGTT
ATCATTGATTCCCGGATCGGCCAAGGCAGCGTAGATCTGGTTGGCGTTTTTAATAAAGCTAAGAAA
AATCCTATCCCGCTAGCAAATGATACATCCTTCGGGGTTGGTTATGCACCGCTGAGCGAAACAGAA
AAAATCGTTTTAGAAACAGAAAAATATCTGAATAGCGATGAATTTAAAAAAAAATATCCGGCAGTT
GGGGAAGATATCAAAGTTATGGGGCTGCGTAAAGGCGATGAAATTGATCTGACAATCGCAGCTGCA
ATCGTTGATAGCGAAGTTGATAATCCGGATGACTATATGGCAGTGAAAGAAGCAATCTATGAAGCA
GCAAAAGGAATCGTTGAATCCCATACTGAACGTCCGACAAATATCTATGTGAATACAGCAGACGAC
CCGAAAGAAGGTATCTATTATATTACAGTTACTGGCACTAGCGCAGAAGCAGGTGATGATGGGTCC
GTTGGCCGTGGTAATCGTGTTAATGGTCTGATCACACCGAATCGTCACATGAGCATGGAAGCTGCT
GCTGGGAAAAATCCGGTGAGCCATGTTGGGAAAATCTATAATATCCTGTCGATGCTGATTGCTAAT
GATATTGCAGAACAAGTTGAAGGTGTTGAAGAAGTTTATGTTCGCATCCTGAGCCAAATAGGCAAA
CCGATTGATGAACCCCTGGTTGCAAGCGTGCAAATTATCCCGAAAAAAGGTTATTCGATCGATGTT
CTGCAAAAACCGGCTTATGAAATTGCAGATGAATGGTTAGCAAATATTACTAAAATTCAAAAAATG
ATCCTGGAAGATAAAGTAAATGTTTTTCTCGAGCACCACCACCACCACCACTGA

Protein Sequence:

MAGKVRNIVVEELVRTPVEMQKVELVERKGIGHPDSIADGIAEAVSRALSREYVKRYGIILHHNTD
QVEVVGGRAYPQFGGGEVIKPIYILLSGRAVEMVDREFFPVHEIALKAAKDYLRKAVRHLDLEHHV
IIDSRIGQGSVDLVGVFNKAKKNPIPLANDTSFGVGYAPLSETEKIVLETEKYLNSDEFKKKYPAV
GEDIKVMGLRKGDEIDLTIAAAIVDSEVDNPDDYMAVKEAIYEAAKGIVESHTERPTNIYVNTADD
PKEGIYYITVTGTSAEAGDDGSVGRGNRVNGLITPNRHMSMEAAAGKNPVSHVGKIYNILSMLIAN
DIAEQVEGVEEVYVRILSQIGKPIDEPLVASVQIIPKKGYSIDVLQKPAYEIADEWLANITKIQKM
ILEDKVNVFLEHHHHHH

Plasmid Map:

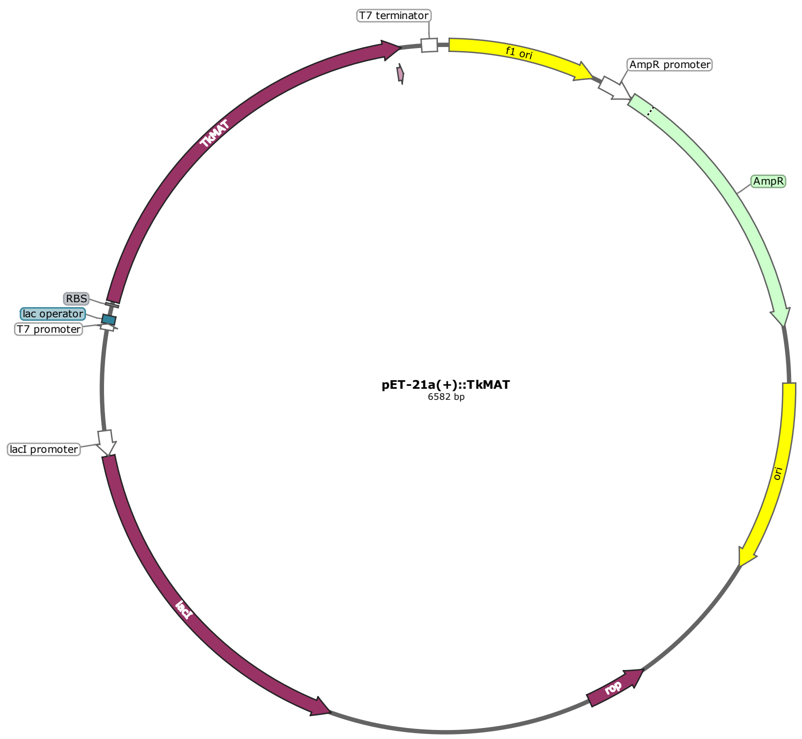

*Ct*HMT – Halide methyl transferase

- Organism of origin: *Chloracidobacterium thermophilum*
- UniProt: G2LF24
- GenBank: CP002514.1, AEP12557.1
- ε_{280} (Cys-bridges) = 65680 M^{-1} cm^{-1}
- ε_{280} (Cys reduced) = 65430 M^{-1} cm^{-1}
- MW = 24617.03 Da

Gene Sequence:

ATGGGCCATCATCATCATCACCATGCAGAAAATCTGTATTTTCAGGGTAGCGGCCTGGGTATGGAT
GCAGATACCGCAAGTTTTTGGGAAGAAAAATATCGTGCAGATCTGACCGCCTGGGATCGTGGTGGT
GTGAGTCCGGCACTGGAACATTGGCTGGCAGAAGGCGCCCTGAAACCGGGTCGCATTCTGATTCCG
GGCTGTGGTTATGGTCATGAAGTGCTGGCCCTGGCACGTCGTGGCTTTGAAGTGTGGGGTCTGGAT
ATTGCACTGACCCCGGTTCGTCGCCTGCAGGAAAAACTGGCACAGGCCGGTCTGACCGCCCATGTT
GTTGAAGGTGACGTGCGTACCTGGCAGCCGGAACAGCCGTTTGATGCAGTGTATGAACAGACCGCC
CTGTGCGCACTGAGTCCGGAAGATTGGCCGCGCTATGAAGCCCAGCTGTGCCGCTGGCTGCGCCCT
GGTGGTCGTCTGTTTGCCCTGTGGATGCAGACCGATCGTCCGGGTGGCCCGCCGTATCATTGCGGC
CTGGAAGCCATGCGTGTGCTGTTTGCACTGGAACGTTGGCGTTGGGTTGAACCGCCGCAGCGTACC
GTGCCGCATCCGACAGGCTTTTTCGAATATGCCGCAATTCTGGAACGCCTGGTT

Protein Sequence:

MGHHHHHHAENLYFQGSGLGMDADTASFWEEKYRADLTAWDRGGVSPALEHWLAEGALKPGRILIP
GCGYGHEVLALARRGFEVWGLDIALTPVRRLQEKLAQAGLTAHVVEGDVRTWQPEQPFDAVYEQTC
LCALSPEDWPRYEAQLCRWLRPGGRLFALWMQTDRPGGPPYHCGLEAMRVLFALERWRWVEPPQRT
VPHPTGFFEYAAILERLV

Plasmid Map:

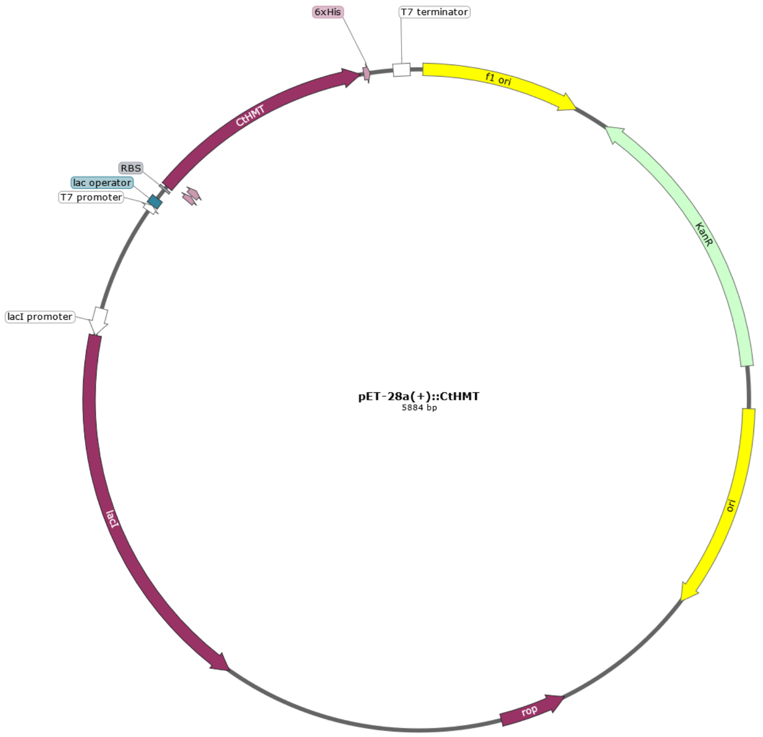

Results

DoE-guided expression optimisation of *Sg*PsmC

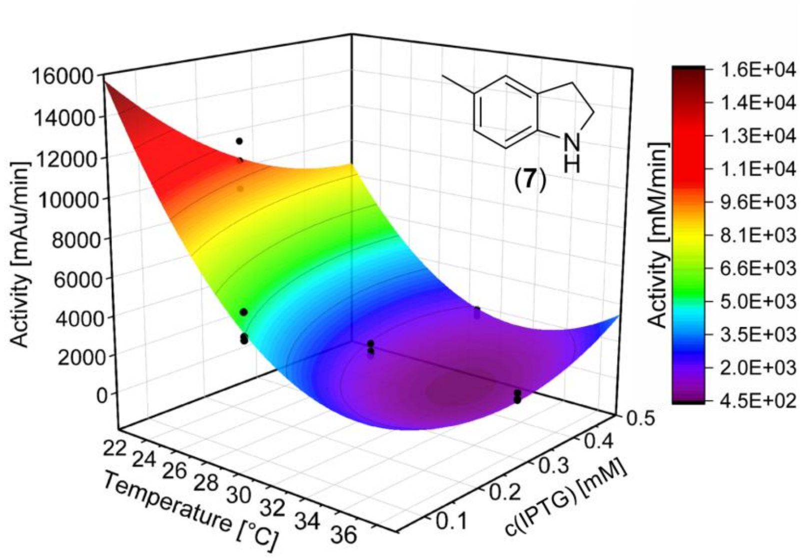

Figure S1a. Surface response plot showing the effect of the factors cultivation temperature and inducer (IPTG) concentration on enzyme activity. Whole cells were used for activity determinations (normalised to cell density) against the model substrate 5-methyl indoline (**7**). Highest activity was measured at low cultivation temperatures and low IPTG concentrations. OD_{600} at induction (here at 2.525) had the lowest effect of the three factors tested. Relative enzyme activities spanned around one order of magnitude in the tested set of cultivation conditions. This is also the case when taking the space-time-yield (defined here as the product of methylation activity and cell density over cultivation time until reaching stationary phase) into account (see **Fig. S4**).

Table S22: ANOVA scores for all responses analysed during the DoE-guided expression optimisation of *Sg*PsmC.

Parameter	***SD***	***Mean***	***C.V. %***	***R^2***	***Adjusted R^2***	***Predicted R^2***	***Adjusted precision***
*Sg*PsmC activity [mAu/min]	711.33	4353.62	16.34	0.9712	0.9640	0.9518	32.5182
Total POI/reference [pixel/pixel]	0.0273	0.1248	21.90	0.6891	0.6114	0.5013	10.1814
Soluble POI/reference [pixel/pixel]	0.0251	0.1295	19.40	0.7149	0.6416	0.5372	11.4992

Table S23: Polynomials describing the surface plot of the respective responses. A: Temperature [°C], B: IPTG concentration [mM], C: OD_{ind}.

Activity [mAu/min]	=	Total POI/Reference	=	Soluble POI/Reference	=
+72217.1 mAu·min⁻¹		+0.036418 mAu·min⁻¹		+0.017154 mAu·min⁻¹	
-3952.4 °C·mAu·min⁻¹	A	+0.013171 °C·mAu·min⁻¹	A	+0.012771 °C·mAu·min⁻¹	A
-57530.7 mM⁻¹·mAu·min⁻¹	B	-0.410924 mM⁻¹·mAu·min⁻¹	B	-0.170457 mM⁻¹·mAu·min⁻¹	B
+1584.8 mAu·min⁻¹	C	+0.033860 mAu·min⁻¹	C	+0.028539 mAu·min⁻¹	C
+1020.2 mM⁻¹·°C⁻¹·mAu·min⁻¹	A * B	+0.000391 mM⁻¹·°C⁻¹·mAu·min⁻¹	A * B	-0.006664 mM⁻¹·°C⁻¹·mAu·min⁻¹	A * B
-57.9 °C⁻¹·mAu·min⁻¹	A * C	-0.000283 °C⁻¹·mAu·min⁻¹	A * C	-0.000102 °C⁻¹·mAu·min⁻¹	A * C
+981.0 mM⁻¹·mAu·min⁻¹	B * C	+0.030199 mM⁻¹·mAu·min⁻¹	B * C	+0.030050 mM⁻¹·mAu·min⁻¹	B * C
+57.6 C⁻²·mAu·min⁻¹	A²	-0.000301 C⁻²·mAu·min⁻¹	A²	-0.000273 C⁻²·mAu·min⁻¹	A²
+34439.2 mM⁻²·mAu·min⁻¹	B²	+0.398713 mM⁻²·mAu·min⁻¹	B²	+0.350263 mM⁻²·mAu·min⁻¹	B²
+16.1 mAu·min⁻¹	C²	-0.005420 mAu·min⁻¹	C²	-0.005028 mAu·min⁻¹	C²

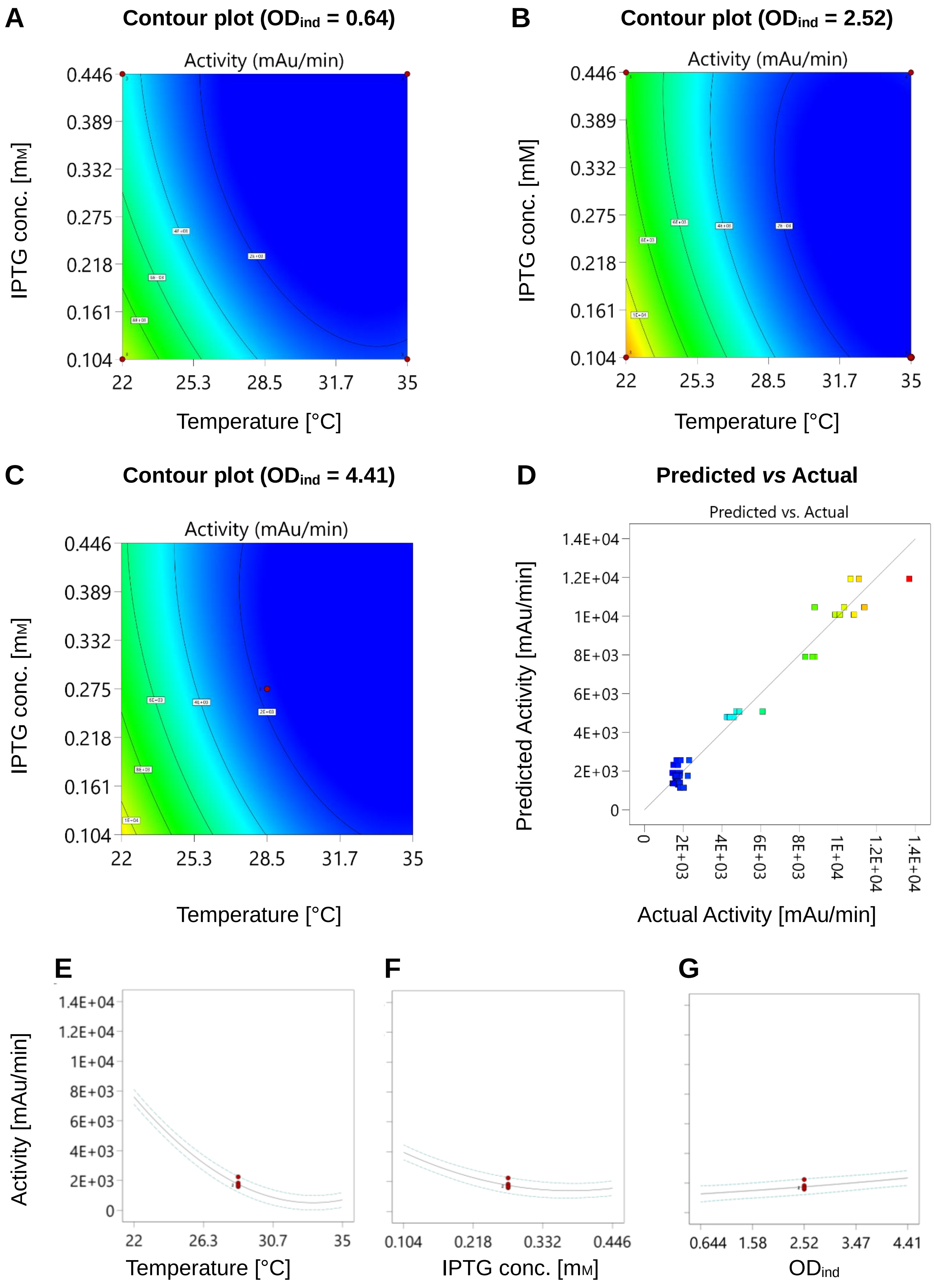
A
Contour plot (ODind = 0.64)
Activity (mAu/min)
IPTG conc. [mM]
Temperature [°C]
B
Contour plot (ODind = 2.52)
Activity (mAu/min)
IPTG conc. [mM]
Temperature [°C]
C
Contour plot (ODind = 4.41)
Activity (mAu/min)
IPTG conc. [mM]
Temperature [°C]
D
Predicted vs Actual
Predicted vs. Actual
Predicted Activity [mAu/min]
Actual Activity [mAu/min]
E
F
G
Activity [mAu/min]
Temperature [°C]
IPTG conc. [mM]
ODind

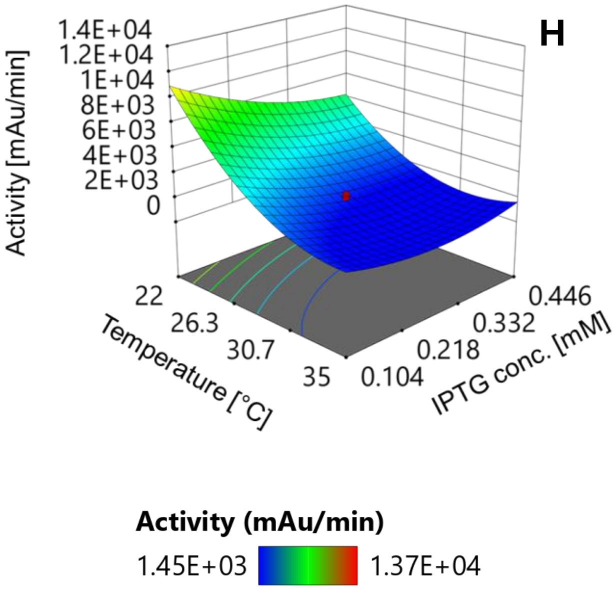

Figure S1b: Graphical overview of DoE-guided expression optimisation results using the *primary readout of whole cell methyltransferase activity* as assessed by RP-HPLC. (**A-C**) Contour plots of activity *vs* IPTG concentration and temperature at fixed OD_{ind}. Highest activity was observed for combinations of low cultivation temperatures and low inducer concentrations. (**D**) Predicted *vs* actual activity. High R^2 of 0.95 indicates the applicability of the chosen primary readout. (**E-F**) ANOVA plots for each factor. Cultivation temperature had the strongest impact on activity, followed by inducer concentration. While the effect of OD_{inc} was minimal, this allowed a simple cultivation protocol. Dashed lines indicate 95% CI. (**H**) Final surface response (with OD_{ind} = 2.525). Highest activity was observed for low cultivation temperatures and low inducer concentrations. The primary response spanned around one order of magnitude.

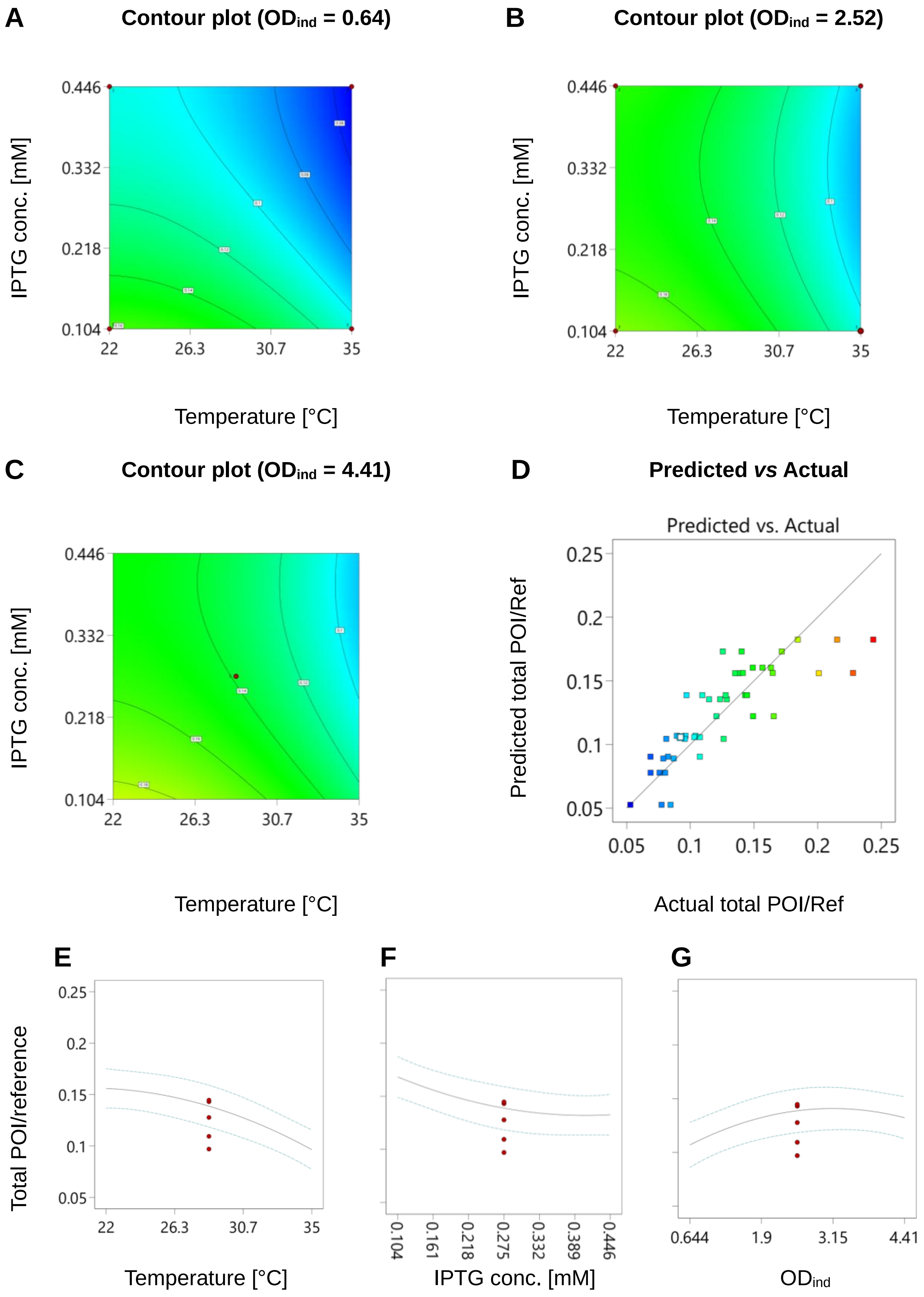
A
Contour plot (ODind = 0.64)
B
Contour plot (ODind = 2.52)
IPTG conc. [mM]
0.446
0.332
0.218
0.104
22
26.3
30.7
35
Temperature [°C]
C
Contour plot (ODind = 4.41)
D
Predicted vs Actual
Predicted vs. Actual
Predicted total POI/Ref
0.25
0.2
0.15
0.1
0.05
Actual total POI/Ref
E
F
G
Total POI/reference
IPTG conc. [mM]
0.104
0.161
0.218
0.275
0.332
0.389
0.446
ODind
0.644
1.9
3.15
4.41

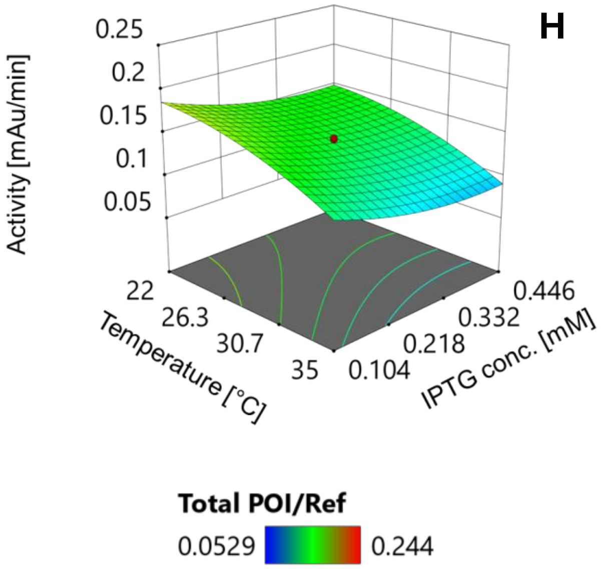

Figure S1c: Graphical overview of DoE-guided expression optimisation results using the *secondary readout of total protein of interest (POI)* as assessed densitometrically by SDS-PAGE (Coomassie stain). The readout was always normalised to a control with a defined concentration of purified *Sg*PsmC (POI/ref). Qualitatively, similar results as with the primary response can be observed (*i.e.*, highest POI/ref was observed for low cultivation temperatures and low inducer concentrations), however, the differences between the individual experimental runs are not as pronounced. Furthermore, the secondary readout suggests similarly strong effects on POI/Ref by all three factors. Compared to the primary response, high POI/Ref were not adequately predicted by the fitted model, leading to a lower R^2 of 0.50. (**A-C**) Contour plots of POI/ref *vs* IPTG concentration and temperature at fixed OD_{ind}. (**D**) Predicted *vs* actual activity. R^2 of 0.50 indicates limited applicability of the chosen secondary readout. (**E-F**) ANOVA plots for each factor. Dashed lines indicate 95% CI. (**H**) Final surface response (with OD_{ind} = 2.525).

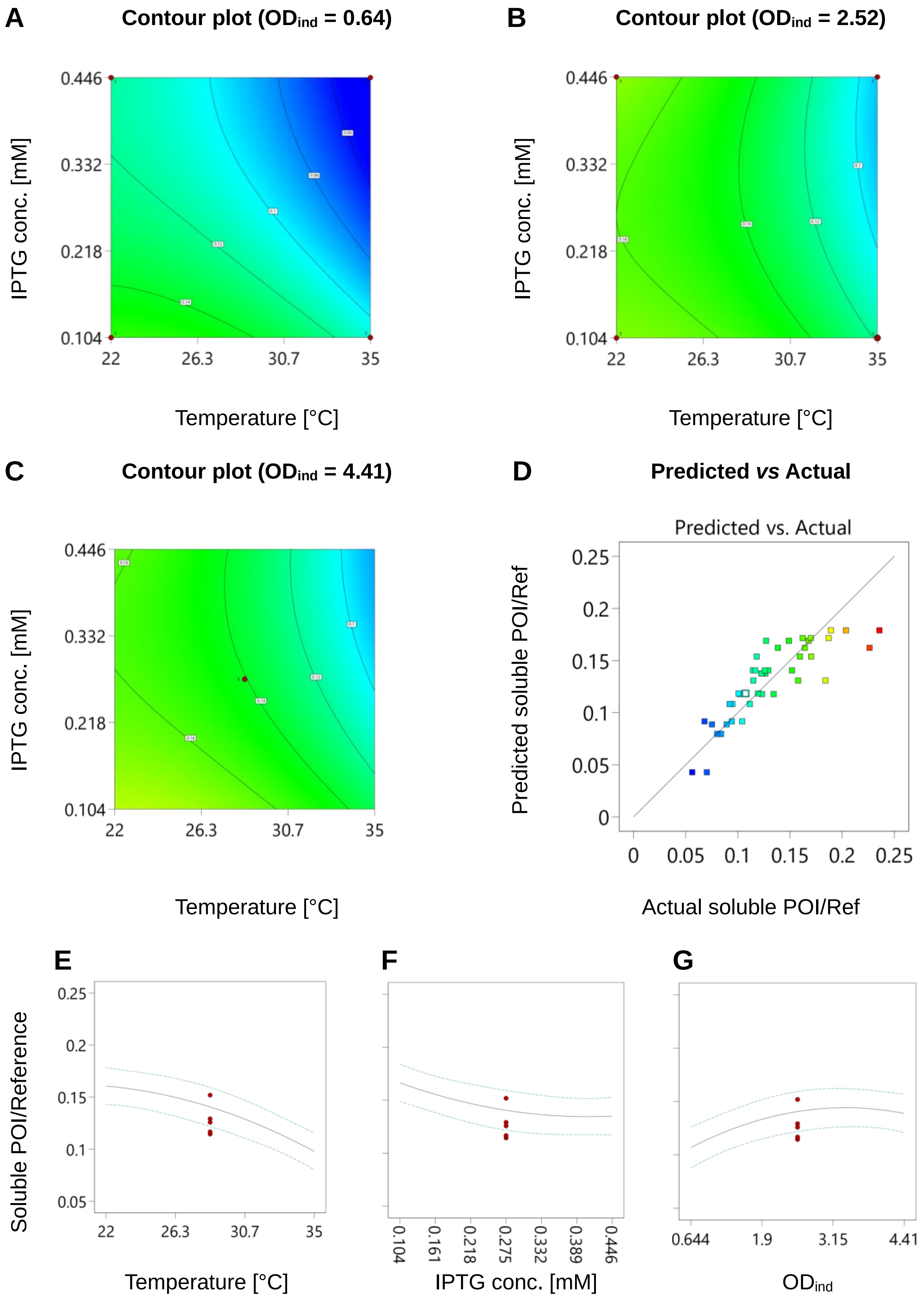

A
Contour plot (ODind = 0.64)
B
Contour plot (ODind = 2.52)
IPTG conc. [mM]
0.446
0.332
0.218
0.104
22
26.3
30.7
35
Temperature [°C]
C
Contour plot (ODind = 4.41)
D
Predicted vs Actual
Predicted vs. Actual
Predicted soluble POI/Ref
Actual soluble POI/Ref
0
0.05
0.1
0.15
0.2
0.25
E
F
G
Soluble POI/Reference
Temperature [°C]
IPTG conc. [mM]
0.104
0.161
0.218
0.275
0.332
0.389
0.446
0.644
1.9
3.15
4.41
ODind

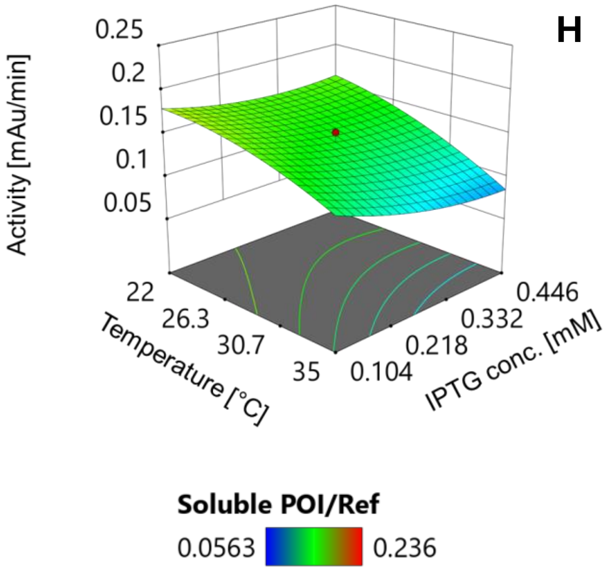

Figure S1d: Graphical overview of DoE-guided expression optimisation results using the *secondary readout of soluble protein of interest (POI)* as assessed densitometrically by SDS-PAGE (Coomassie stain). The readout was always normalised to a control with a defined concentration of purified *Sg*PsmC (POI/ref). The results coincide with those of the total POI secondary readout. Qualitatively, similar results as with the primary response can be observed (*i.e.*, highest POI/ref was observed for low cultivation temperatures and low inducer concentrations), however, the differences between the individual experimental runs are not as pronounced. Furthermore, the secondary readout suggests similarly strong effects on POI/Ref by all three factors. Compared to the primary response, high POI/Ref were not adequately predicted by the fitted model, leading to a lower R^2 of 0.53. (**A-C**) Contour plots of POI/ref *vs* IPTG concentration and temperature at fixed OD_{ind}. (**D**) Predicted *vs* actual activity. R^2 of 0.53 indicates limited applicability of the chosen secondary readout. (**E-F**) ANOVA plots for each factor. Dashed lines indicate 95% CI. (**H**) Final surface response (with OD_{ind} = 2.525).

Std	Run	F1: Temperature [°C]	F2: IPTG conc. [µM]	F3: OD at induction [OD]	M1: OD at harvest [OD]	M2: Time until stationary phase [h]	R1: Activity [mAu / min]	R1: Activity [mM SAH / min]	R2: Total POI / Ref. [pixel/pixel]	R3: Soluble POI / Ref. [pixel/pixel]	M3: Space-Time-Yield [mAu / min] X [OD] / [h]
1	22	22.04	0.104	0.644	47.2	22.9	9861	0.00158	0.16338	0.11808	20289
2	15	22.04	0.104	0.644	46.9	23.9	10827	0.00174	0.14908	0.15946	21303
3	36	22.04	0.104	0.644	47.6	22.5	10099	0.00162	0.15670	0.17031	21348
4	25	34.96	0.104	0.644	48.0	20.2	1495	0.00024	0.10403	0.09472	3557
5	13	34.96	0.104	0.644	47.1	20.2	1709	0.00027	0.08938	0.09212	3988
6	30	34.96	0.104	0.644	48.1	20.1	1743	0.00028	0.09629	0.11151	4171
7	24	22.04	0.446	0.644	45.6	31.1	4619	0.00074	0.09576	0.12315	6768
8	12	22.04	0.446	0.644	44.8	30.9	4266	0.00069	0.12617	0.13435	6187
9	5	22.04	0.446	0.644	45.0	30.4	4417	0.00071	0.08114	0.10185	6527
10	31	34.96	0.446	0.644	45.6	20.2	1602	0.00026	0.07745	0.07025	3622
11	18	34.96	0.446	0.644	46.6	20.0	1717	0.00028	0.05290	0.05627	3999
12	41	34.96	0.446	0.644	47.1	20.1	1729	0.00028	0.08452	0.11432	4051
13	16	22.04	0.104	4.406	46.8	21.4	10651	0.00171	0.17185	0.16787	23268
14	2	22.04	0.104	4.406	47.1	22.0	13685	0.00220	0.12555	0.12713	29254
15	29	22.04	0.104	4.406	45.7	21.0	11085	0.00178	0.14032	0.14892	24119
16	23	34.96	0.104	4.406	45.9	20.2	1740	0.00028	0.10753	0.11931	3960
17	8	34.96	0.104	4.406	41.4	20.2	1785	0.00029	0.09234	0.10740	3660
18	3	34.96	0.104	4.406	43.2	20.1	1683	0.00027	0.10316	0.10063	3616
19	10	22.04	0.446	4.406	42.2	29.1	8318	0.00134	0.13801	0.17002	12066
20	47	22.04	0.446	4.406	42.1	29.0	8787	0.00141	0.20094	0.18702	12732
21	28	22.04	0.446	4.406	42.5	28.0	8684	0.00139	0.13526	0.16212	13160
22	37	34.96	0.446	4.406	44.7	20.2	1668	0.00027	0.10746	0.10418	3697
23	34	34.96	0.446	4.406	44.9	20.0	1666	0.00027	0.08243	0.09422	3735
24	17	34.96	0.446	4.406	46.0	20.1	1691	0.00027	0.06873	0.06807	3868
25	21	20.00	0.275	2.525	48.4	28.7	10318	0.00166	0.22785	0.22630	17410
26	42	20.00	0.275	2.525	46.4	29.7	11365	0.00183	0.14127	0.13845	17724
27	38	20.00	0.275	2.525	47.5	30.8	8799	0.00141	0.16472	0.16423	13563
28	9	37.00	0.275	2.525	48.6	20.2	1820	0.00029	0.06882	0.08343	4379
29	27	37.00	0.275	2.525	48.8	20.2	1459	0.00023	0.08024	0.08393	3526
30	4	37.00	0.275	2.525	46.2	20.3	1489	0.00024	0.07583	0.08019	3391
31	39	28.50	0.050	2.525	38.9	13.3	4739	0.00076	0.21534	0.18923	13913
32	44	28.50	0.050	2.525	43.7	14.0	4915	0.00079	0.18444	0.20363	15346
33	7	28.50	0.050	2.525	40.1	13.3	6104	0.00098	0.24375	0.23572	18344
34	33	28.50	0.500	2.525	35.3	20.4	1847	0.00030	0.12345	0.12643	3207
35	1	28.50	0.500	2.525	38.7	20.4	1686	0.00027	0.11477	0.12348	3204
36	46	28.50	0.500	2.525	37.9	20.2	1452	0.00023	0.12872	0.12262	2725
37	6	28.50	0.275	0.050	41.0	14.6	1463	0.00024	0.03672	0.03987	4104
38	43	28.50	0.275	0.050	40.2	17.5	1847	0.00030	0.07881	0.08929	4249
39	40	28.50	0.275	0.050	39.8	17.5	2034	0.00033	0.08702	0.07520	4626
40	11	28.50	0.275	5.000	33.4	20.2	1663	0.00027	0.16555	0.18401	2748
41	20	28.50	0.275	5.000	34.2	20.0	2313	0.00037	0.12051	0.11486	3946
42	45	28.50	0.275	5.000	35.4	20.4	1875	0.00030	0.14932	0.15778	3260
43	14	28.50	0.275	2.525	36.7	20.1	2239	0.00036	0.14308	0.15192	4091
44	26	28.50	0.275	2.525	36.9	19.6	1585	0.00025	0.09703	0.11449	2979
45	35	28.50	0.275	2.525	38.8	19.5	1700	0.00027	0.12779	0.12918	3377
46	19	28.50	0.275	2.525	37.8	20.4	1829	0.00029	0.14463	0.12586	3395
47	32	28.50	0.275	2.525	35.6	20.0	1662	0.00027	0.10945	0.11673	2953

Table S24: Overview of all factors and corresponding levels and combinations hereof tested in the DoE-guided optimisation of *Sg*PsmC expression. Factors are marked with an 'F'. Responses used for final surface response analyses are marked with an 'R'. Additional measurements and calculated parameters are denoted with an 'M'. Std: Ordered ID of each experimental run. Run: Randomised ID of each experimental run.

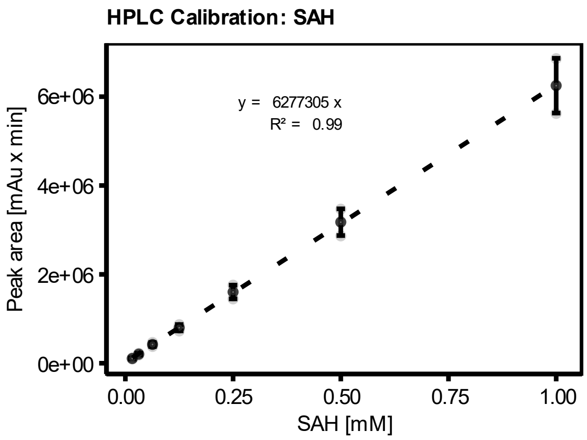

Figure S2: HPLC calibration of SAH. Darker points indicate average values. Performed with *n* = 3, error denotes SD.

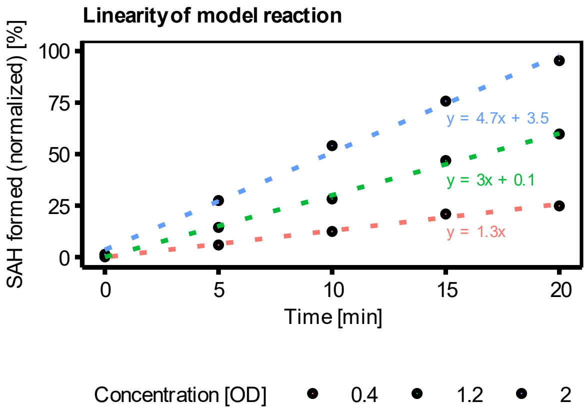

Figure S3: SAH formed (normalised to maximum) over time during the *Sg*PsmC catalysed *N*-methylation of the model substrate 5-Me-indoline (**7**). *Sg*PsmC was used in the form of whole cells with the indicated final OD in the reactions. SAH formation behaves linearly for up to an OD of 2 and up to 20 min reaction time. Performed with *n* = 1.

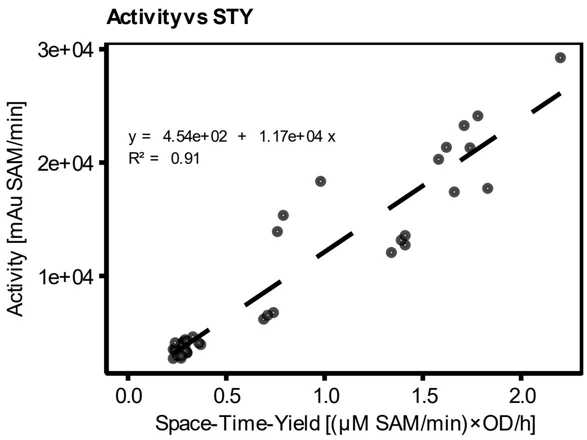

Figure S4: Activity data from cultivation conditions tested during expression optimisation of *Sg*PsmC plotted against space-time-yield (STY). STY is calculated as $\mathrm{STY} = \frac{\mathrm{Activity}\left[\mathrm{mAu}\frac{\mathrm{SAM}}{\mathrm{min}}\right] \cdot \mathrm{OD}_{\mathrm{Stationary\ Phase}}[\mathrm{OD}]}{\mathrm{Time\ until\ stationary\ phase\ [h]}}$. In principle, conditions yielding the highest activity (OD_{600}-normalised) also scored the highest when STY was considered.

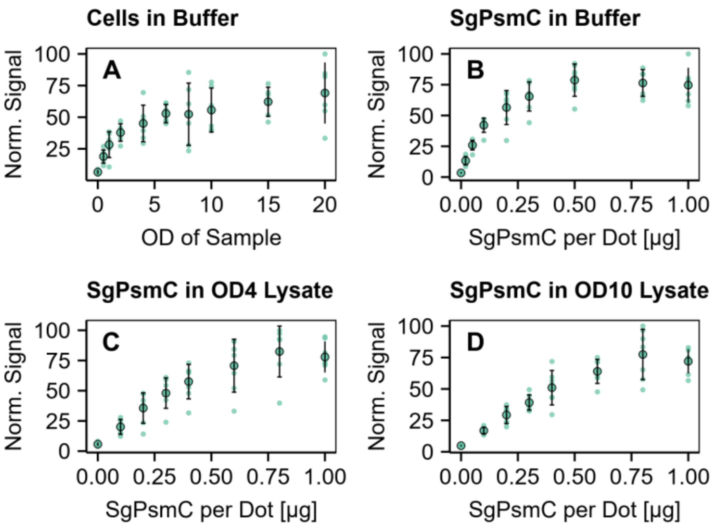

Figure S5: Dot blot calibrations using *Sg*PsmC expressing cells of different optical density (OD_{600}) resuspended in buffer (**A**), and different amounts of purified *Sg*PsmC in buffer (**B**), empty vector lysates with OD_{600} = 4 (**C**), and empty vector lysates with OD_{600} = 10 (**D**). Data normalised to maximum of each individual data set. *n* = 6; average values shown in darker colour; error bars denote SD.

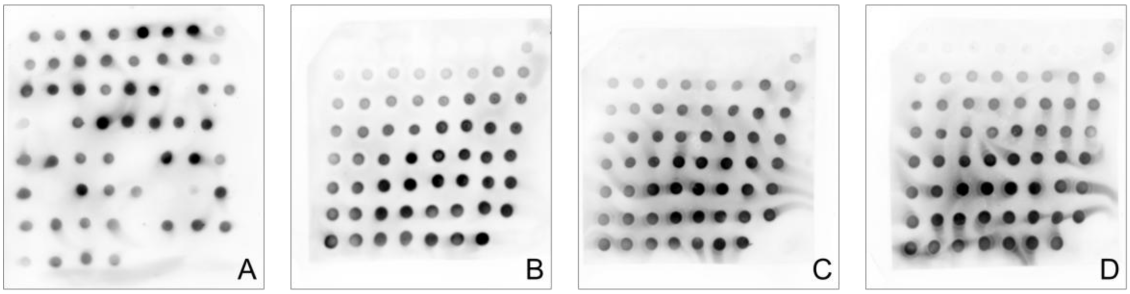

Figure S6: Corresponding dot-blots to **Fig. S5**. Irrespective of 'smearing dots', data spread too strongly (see **Fig. S5**) to be taken into consideration for the final workflow for protein expression optimisation.

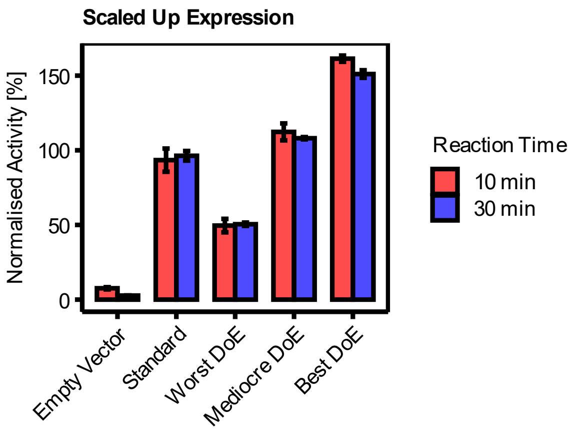

Figure S7: Normalised activity of whole cells from scaled-up (500 mL culture) expression of *Sg*PsmC using selected conditions from the DoE optimisation. 'Standard' indicates non-optimised expression, 'Best', 'Worst', and 'Mediocre' indicate the conditions yielding the highest, lowest, and a mid-ranged primary response during the optimisation procedure, respectively. The 'Best' condition outperformed the non-optimised expression by ~60%. While the conditions at the 500 mL scale (up-scaled) and at the 1.2 mL (DoE optimisation in FlowerPlates) show the same trend, relative differences between the conditions at 500 mL and 1.2 mL scale differ strongly. This may be because additional parameters – apart from the DoE factors – could not be replicated in the scale-up, such as the form of the FlowerPlate wells and O_2 aeration in the BioLector. *n* = 3; error bars denote SD.

Enzyme purification

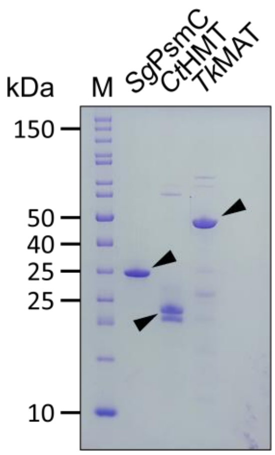

Figure S8: SDS-PAGE of all IMAC-purified proteins used in this work. Black arrows indicate bands belonging to *Sg*PsmC (predicted MW = 30.1 kDa), *Ct*HMT (predicted MW = 24.6 kDa), and *Tk*MAT (predicted MW = 45.7 kDa), respectively. After purification, *Ct*HMT always showed two close bands of similar apparent molecular weight. M: protein ladder. Coomassie stain.

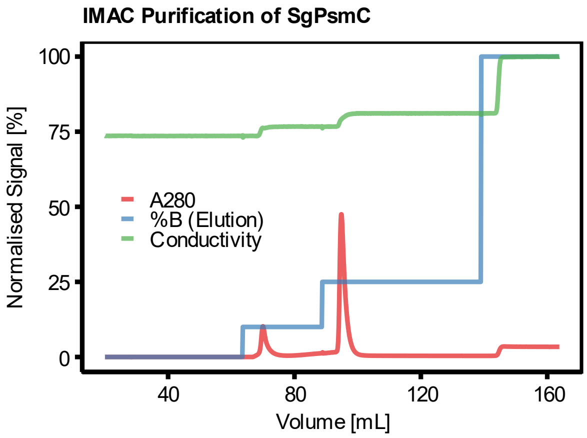

Figure S9: Chromatogram of IMAC purification of *Sg*PsmC. Red: Absorption at 280 nm (A280); blue: concentration (% v/v) of elution buffer containing 1 M imidazole, final purging step performed with 100% elution buffer; green: conductivity. All data normalised. A280 additionally scaled down for visual clarity.

Additional enzymatic profiling of *Sg*PsmC and oligomeric state

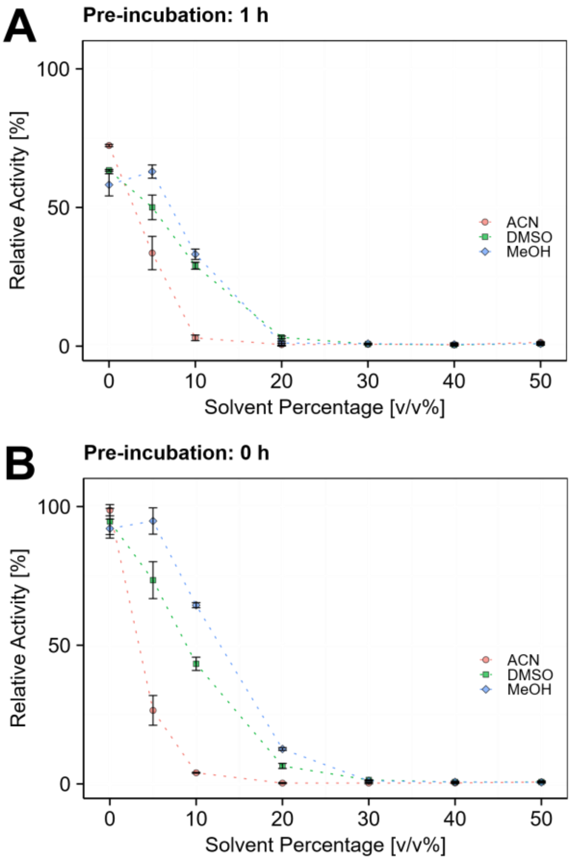

Figure S10: Solvent tolerance, determined by performing reactions in different solvent concentrations either without a prior 1 h enzyme incubation (**A**) or after a 1 h enzyme incubation (**B**). Solvent tolerance was highest for MeOH, followed by DMSO and CH_3CN. While CH_3CN was detrimental to *Sg*PsmC activity, a slight activation effect was observed at 5% *v/v* MeOH. At the same solvent concentration, *Sg*PsmC still showed close to 75% relative activity in DMSO. Applying *Sg*PsmC at higher solvent concentrations than 10% *v/v* (~60% and ~45% relative activity in MeOH and DMSO, respectively) appeared unfeasible. A 1 h incubation prior to the reaction revealed ~63% and ~55% relative activity for MeOH and DMSO at 5% *v/v*, respectively, with activity loss for 5% v/v MeOH being similar to reactions performed in aqueous buffer.

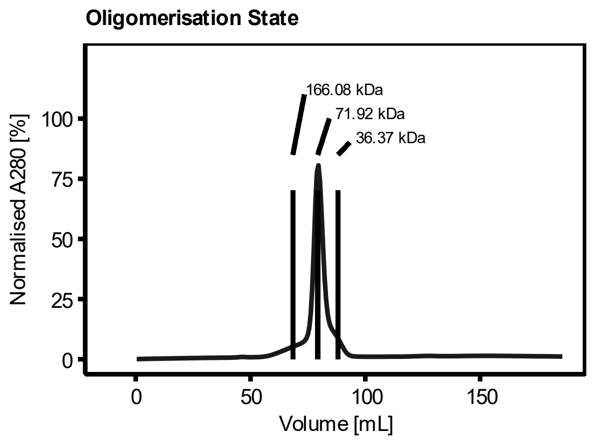

Figure S11: SEC chromatogram with additional hidden peaks identified by peak deconvolution (method **(R)**) and marked with dashed lines. Based on apparent masses, peaks may correspond to a monomeric, dimeric, and tetrameric to hexameric oligomerisation state of *Sg*PsmC (MW = 30.16 kDa).

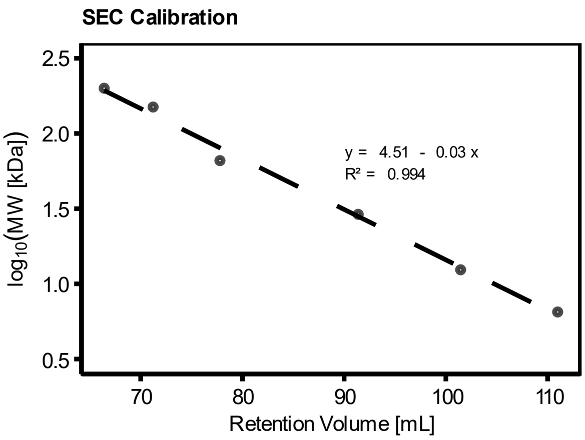

Figure S12: SEC calibration using a commercial protein standard. Molecular weights of proteins were 200 kDa, 66 kDa, 12.4 kDa, 150 kDa, 29 kDa, and 6.5 kDa.

Single-substrate Michaelis-Menten kinetics

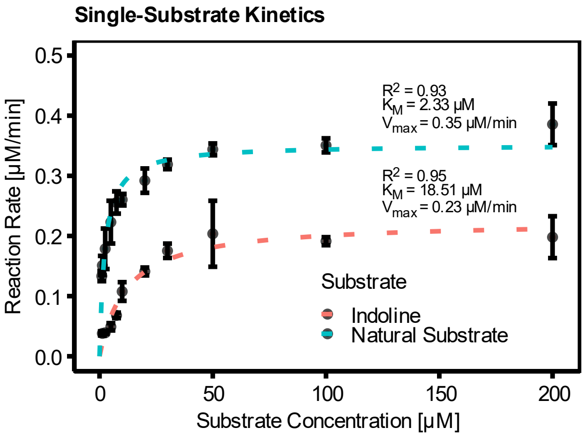

Figure S13: Single-substrate Michaelis-Menten kinetics of *Sg*PsmC using the natural substrate ***(S,S)*-9** (blue) and the minimal model substrate indoline (**8**) (red). Measurements performed with *n* = 3 replicates using the commercially available MTase-Glo^{TM} Assay (Promega); error denotes SD.

Indoline substrate scope

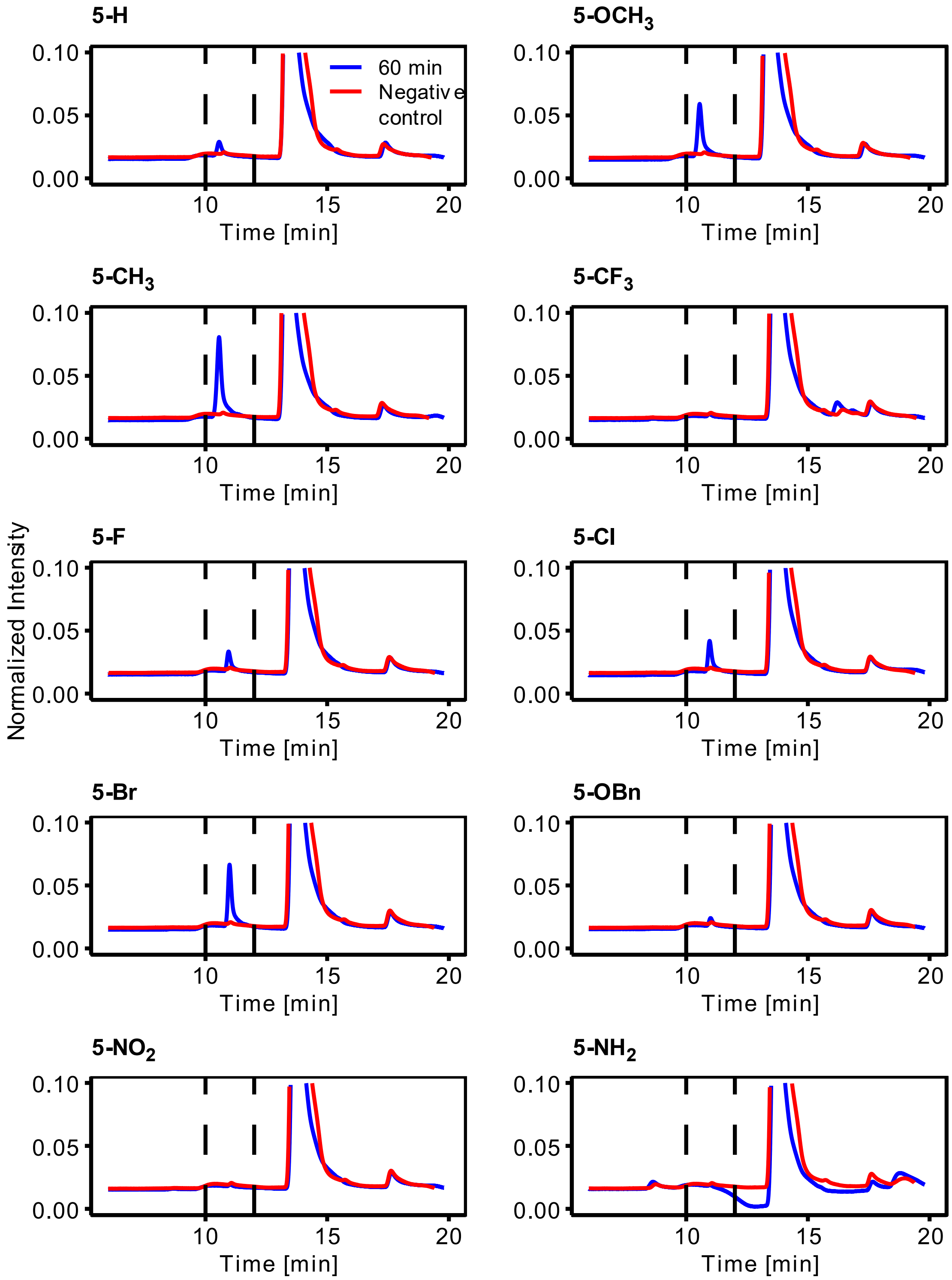

Figure S14a: HPLC-UV traces of the *Sg*PsmC catalysed methylation of various indolines. The highlighted peak corresponds to the by-product SAH. Blue: Samples after 60 min reaction time. Red: Negative controls without *Sg*PsmC. Additional traces are shown in **Fig. S14b**.

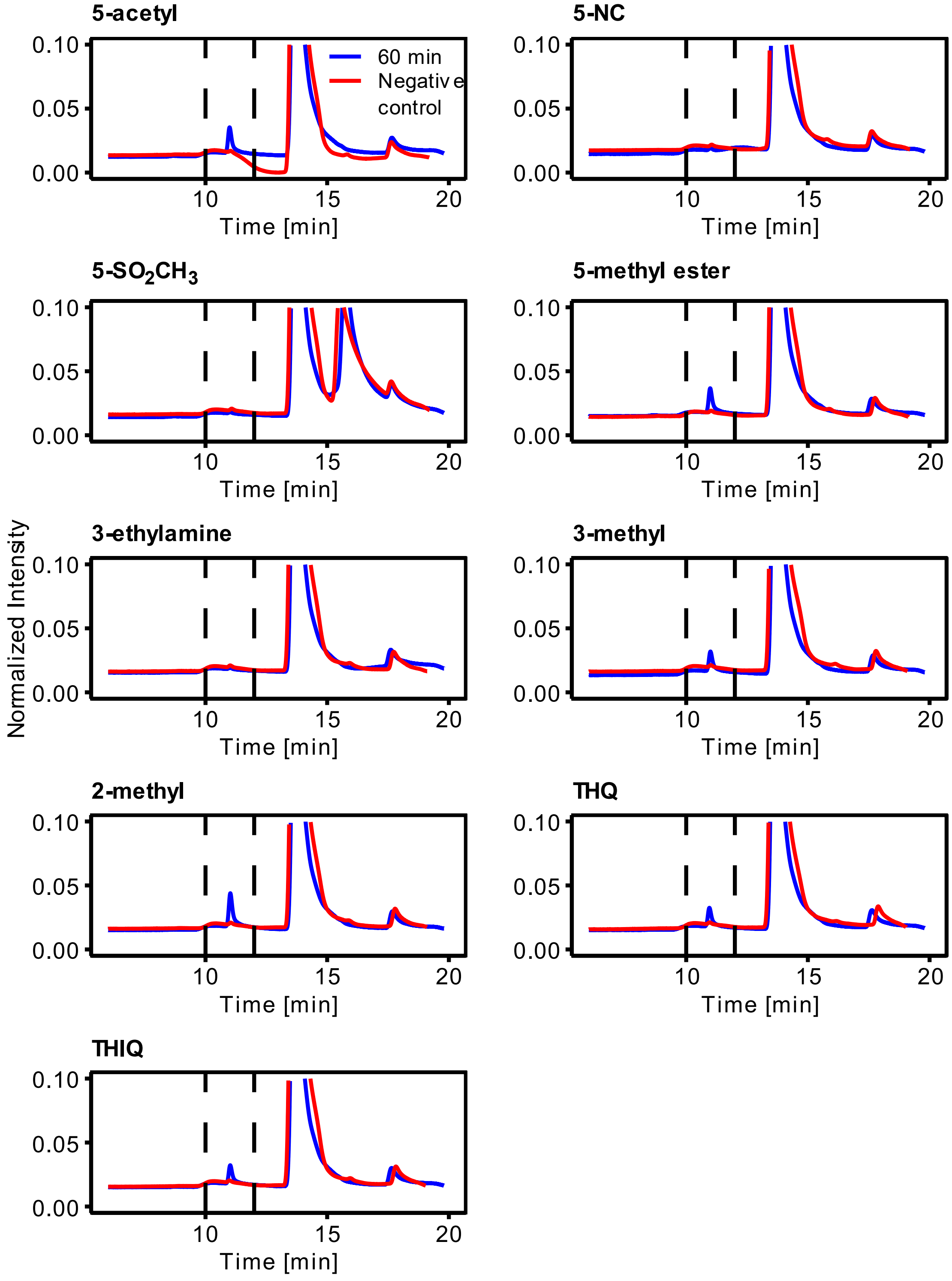

Figure S14b: HPLC-UV traces of the *Sg*PsmC catalysed methylation of various indolines. The highlighted peak corresponds to the by-product SAH. Blue: Samples after 60 min reaction time. Red: Negative controls without *Sg*PsmC. Additional traces are shown in **Fig. S14a**.

Chiral GC analysis of 2-methyl indoline and 3-methyl indoline substrates.

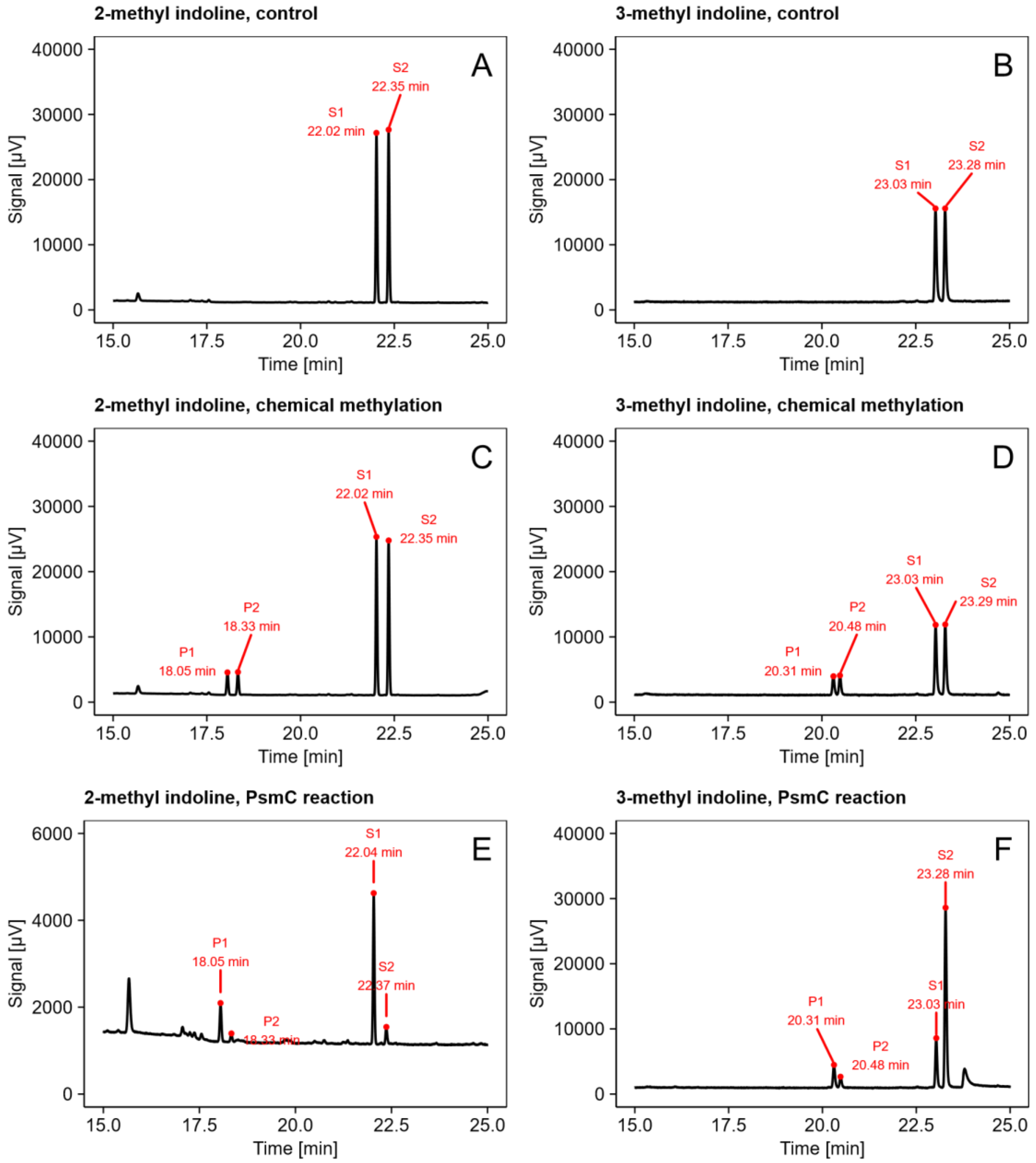

Figure S15: Chiral GC analysis of *Sg*PsmC catalysed reactions of the small racemic indolines 2-methyl indoline (left) and 3-methyl indoline (right). (**A**, **B**): Chiral GC traces of reactions controls without enzyme. (**C**, **D**): Chiral GC traces after unselective chemical methylation. (**E**, **F**): Chiral GC traces of *Sg*PsmC reactions. Substrate and product enantiomers are abbreviated as S1, S2, and P1, P2, respectively, in order of elution.

Analysis of the relative enantioselectivity of *Sg*PsmC

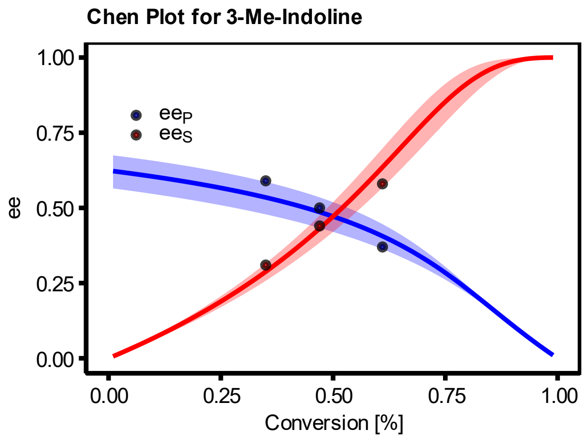

Figure S16a: Chen plot for the *N*-methylation of the substrate 3-Me-indoline showing ee_P and ee_S *vs* conversion. Dots indicate experimental data. The curves describe E = 4.4 ± 0.4 (SE), as determined by non-linear regression. The ribbons indicate the 95% CI. Individual E-values for 3-Me-indoline were 3.7, 4.5, and 5.2.

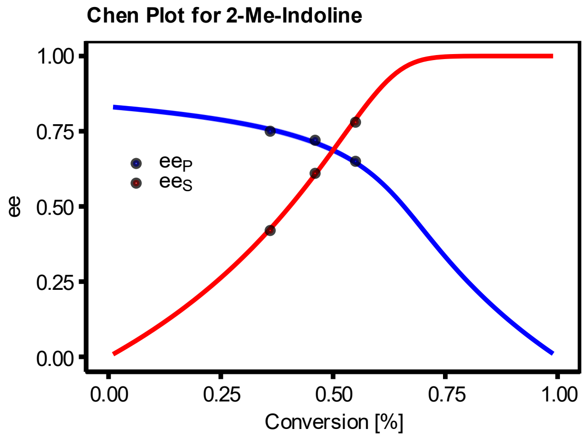

Figure S16b: Chen plot for the *N*-methylation of the substrate 2-Me-indoline showing ee_P and ee_S *vs* conversion. Dots indicate experimental data. The curves describe E = 10.9 ± 0.3 (SE), as determined by non-linear regression. The ribbons indicate the 95% CI. Individual E-values for 2-Me-indoline were 10.3, 11.0, and 11.3.

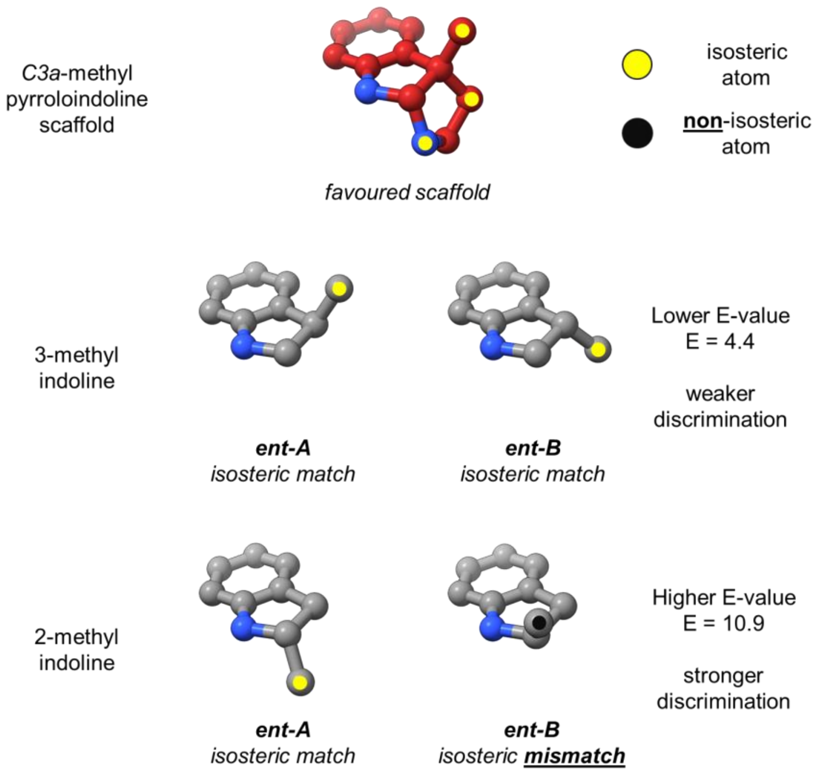

Figure S17: Possible explanation for the different E-values of *Sg*PsmC against 2-methyl indoline and 3-methyl indoline using a simple, isosteric model. The preferred *3a*-methylated pyrroloindoline scaffold of *Sg*PsmC with (*S,S*)-configuration is shown in red (top row; acetyl group at *N1* not shown for the sake of visual clarity). The methyl carbon attached at the stereogenic centre of 3-methyl indoline (middle row) has (irrespectively of the absolute configuration of the stereogenic centre) an isosteric atom (marked in yellow) in the *Sg*PsmC favoured 'full' *3a*-methylated pyrroloindoline scaffold, which may explain the weaker discrimination of the two substrate enantiomers as reflected by the lower E-value of 4.4. In the case of 2-methyl indoline (bottom row), only the methyl carbon of the enantiomer **ent-A** has an isosteric match (marked in yellow) in the pyrroloindoline scaffold, while enantiomer **ent-B** does not (mismatch marked in black), leading to a stronger discrimination by *Sg*PsmC, as reflected by the higher E-value of 10.9.

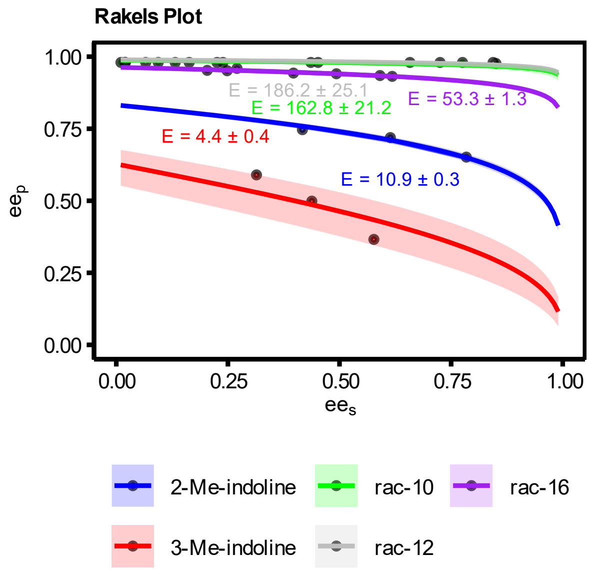

Figure S18: Rakels Plots (ee_P *vs* ee_S) of the *N*-methylation of the substrates 2-Me-indoline (blue), 3-Me-indoline (red), ***rac*-10** (green), ***rac*-12** (grey) and ***rac*-16** (violet) for determination of E-values. For these compounds, E-values could be determined by fitting experimental data to $ee_P(ee_S, E)$ (see Method **(T)**). Dots indicate experimental data. The curves describe the determined E-values, as determined by non-linear regression. Error is SE. Ribbons indicate the 95% CI.

Table S25: E-value determination for the kinetic resolution of ***rac*-13** and ***rac*-14**. Only substrate separation and thus determination only of ee_S was possible for these substrates. Therefore, conversion was measured in parallel by achiral HPLC. The function conversion(ee_S, E) was then fitted to this data set. Due to the error-prone determination of conversion values, the final E-values yielded by this method should be regarded only as a qualitative measure of selectivity.

Compound	ee_S	**Conversion of pyrroloindoline**	**E-value**	**Final E-value**
***rac*-13**	0.92	0.49	268.16	267.15 ± 0.24
	0.98	0.50	267.93	
***rac*-14**	0.237	0.218	15.40	12.30 ± 3.10
	0.237	0.258	6.58	
	0.292	0.293	7.66	
	0.393	0.321	15.82	

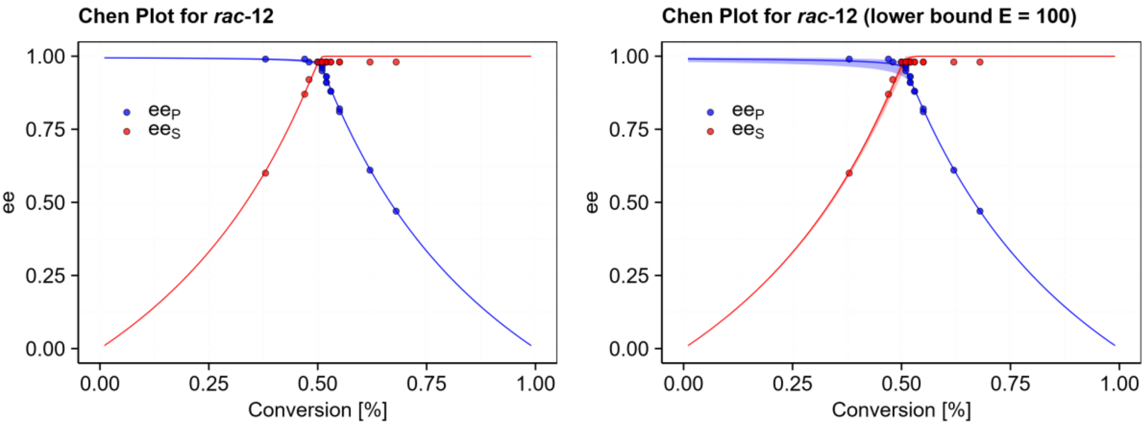

Figure S19: Left: Chen plot for the *N*-methylation of the substrate ***rac*-12** showing ee_p and ee_s *vs* conversion, corresponding to an E-value of 186 ± 25 (SE). Right: As the precision with which *ee* values can be determined using HPLC and GC is too low to determine such high E-values with high certainty, E = 100 (being below the experimentally determined values but nearly as effective as an E-value of E = ∞[42]) has been included as the lower limit of the ribbon for clarity. Dots indicate experimental data. The curves describe E = 186 ± 25 (SE), as determined by non-linear regression. The ribbons indicated the 95% CI.

Chromatographic analysis of pyrroloindolines

Pyrroloindoline substrates and respective chemical and enzymatic methylation reactions hereof were subjected to different chromatographic analyses. Where applicable, these included:

- **A configurational analysis** to determine the absolute enantio-preference of *Sg*PsmC. This was based on chiral HPLC-UV-CD, experimental UV and CD spectra, as well as calculated ECD spectra using racemic substrate references and enzymatic reaction samples with enantio-enriched substrates and products.
- **A chiral HPLC analysis** based on chiral HPLC-UV of racemic substrate references, analytical scale chemical methylation of racemic substrate references, and enzymatic methylation reactions.
- **An achiral HPLC-UV-MS** analysis of racemic substrate references and enzymatic methylation reactions after 0 h and 21 h.

An overview of how these three analyses are connected is given in **Fig. S20**. In the following, all three analyses are grouped by pyrroloindoline substrate, where applicable (groups **Fig. S21** to **S28**).

Configurational analysis

The following general figure description applies to figures concerning configurational analysis (groups **Fig. S21** through **S28**, usually sub-figures **a**, where applicable). Figure specific additions can be found in their respective descriptions.

<u>**(A):**</u> Structure of **(*S*,*S*)-configured pyrroloindoline** substrate for ECD calculation.

<u>**(B):**</u> Overlay of experimental UV spectrum (solid blue line) and experimental ECD spectrum (solid green line) of respective **racemic pyrroloindoline substrate** (enantiomers chromatographically separated) and calculated ECD spectrum (dashed red line) of its **(*S,S*)-enantiomer**. The ECD spectrum was shifted so that the highest signals of both spectra matched (solid red line). The shifted, negative ECD peak at around 240 nm is marked with a dashed line. Due to limited sensitivity, the polarity of the weakest intensity peaks at ~310 nm in the experimental ECD spectrum could not always be given faithfully for all compounds. Therefore, configurational assignment relied primarily on the characteristic peak at ~240 nm. All (*S,S*)-compounds showed very similar ECD spectra with two negative signals at around 200 nm and 240 nm. A further, less pronounced positive signal was predicted at 270 nm. Only the prenylated **(*S,S*)-14** showed deviation from this pattern. Signals were at the same wavelengths but the peaks at around 200 nm and at 270 nm showed opposite polarity.

<u>**(C):**</u> Racemic pyrroloindoline substrates were analysed by HPLC-UV-CD (top two traces, 'rac. controls'). By comparing the polarity of the two substrate enantiomers' HPLC-CD peaks with the calculated ECD spectrum (**B**), absolute configurations can be assigned to both substrate enantiomer peaks. Samples from an analytical scale kinetic resolution of the racemic pyrroloindoline substrate (bottom trace, '*Sg*PsmC reaction') was analysed by chiral HPLC-UV separately. By comparison of the reaction HPLC-UV trace with the control HPLC-UV trace, the stereo-preference of *Sg*PsmC for a given substrate can be deduced. For all tested substrates, *Sg*PsmC showed a preference for the (*S,S*)-configured scaffold, except for the prenylated ***rac*-14**. The preferred substrate enantiomer with a peak of smaller area is marked with a dashed line. All signals normalised to maximum. HPLC-CD baseline is not equal to zero because multiple samples were sequentially measured and recorded in the very same HPLC-CD chromatogram. Slight shifts in retention time are due to some samples not being

measured using the same 'batch' of eluent. Chromatographic separation was very sensitive to eluent composition.

Chiral HPLC analysis

This following general figure description applies to figures concerning chiral HPLC analysis (groups **Fig. S21** through **S28**, usually sub-figures **b**, where applicable). Figure specific additions can be found in their respective descriptions. Where applicable, chiral HPLC-UV traces are shown for the respective racemic pyrroloindoline substrate, the unselective chemical methylation (to obtain *N*-methylated reference peaks), the *Sg*PsmC catalysed *N*-methylation of the substrate, and an *Sg*PsmC catalysed reaction yielding both *N*-methylated product enantiomers. Traces were recorded at 205 nm, if not mentioned otherwise.

Corresponding UV spectra for the substrate and product enantiomers were recorded during chromatographic separation. In comparison to the pyrroloindoline substrates, the *N*-methylated products showed a characteristic red-shift in absorption.

Substrate and product enantiomers are abbreviated with S1, S2, and P1, P2, respectively, in order of elution. If no chiral separation was possible, the index is omitted. Specifications in parenthesis indicate from which HPLC samples the spectra were extracted.

Achiral HPLC-UV-MS analysis

This following general figure description applies to figures concerning achiral HPLC-UV-MS analysis (groups **Fig. S21** through **S28**, usually sub-figures **c**, where applicable). Figure specific additions can be found in their respective descriptions. Where applicable, chiral HPLC-UV traces are shown for the *Sg*PsmC catalysed *N*-methylation of the respective racemic substrate after 0 h and 21 h (the latter is only shown if the substrate was converted). Traces were recorded at 205 nm, if not mentioned otherwise.

Corresponding UV and MS spectra for the substrate and product were recorded during chromatographic separation. In comparison to the pyrroloindoline substrates, the *N*-methylated products showed a characteristic red-shift in their UV spectra and a mass shift of +14 m/z.

Substrate and product are abbreviated with S and P. The specification in parenthesis indicates from which peaks the UV and MS spectra were extracted.

General reaction conditions: 4.3 µM *Sg*PsmC, 2 mM SAM, 1 mM pyrroloindoline; 35 °C, 700 rpm, 0 h and 21 h reaction time.

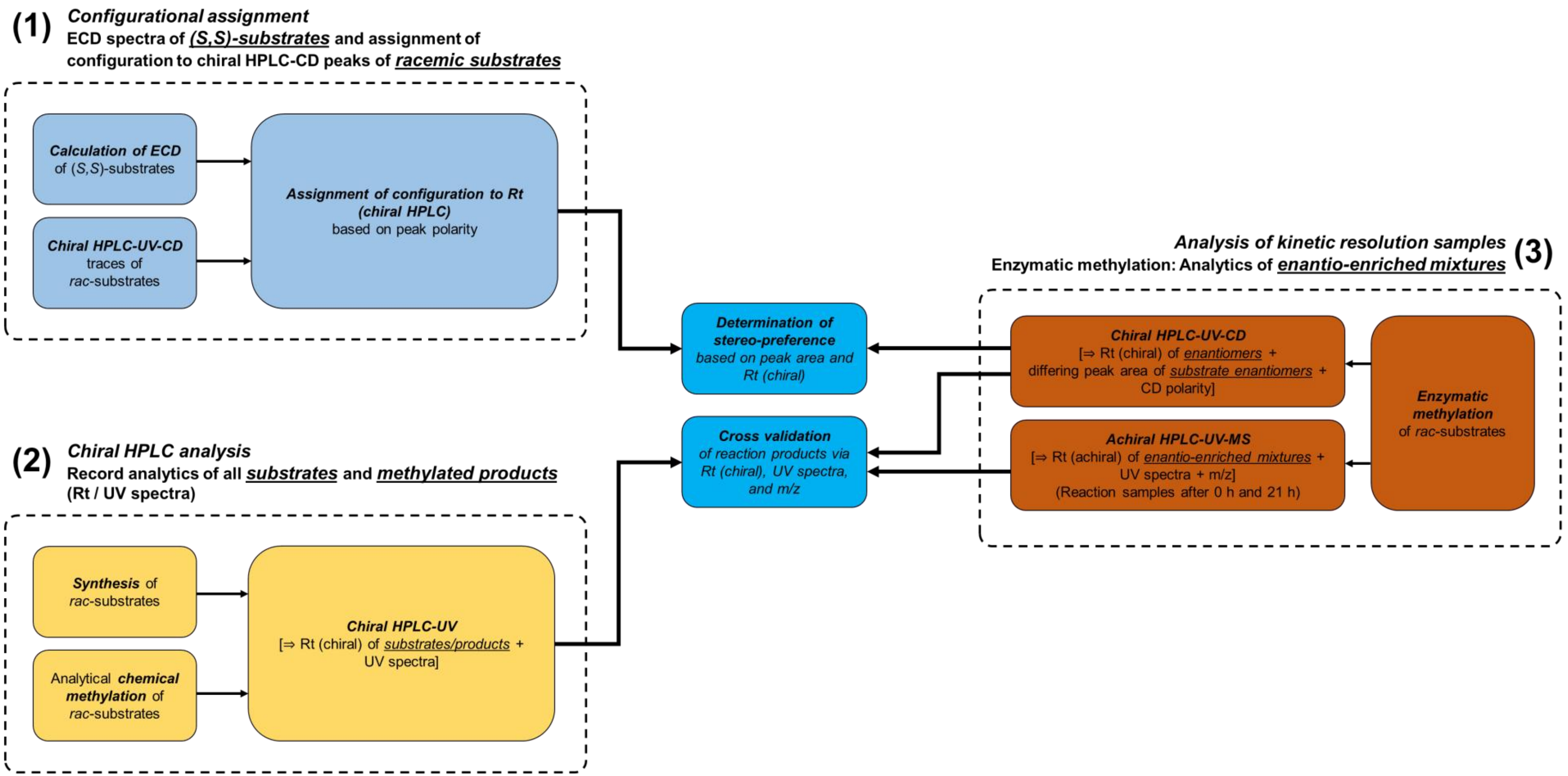

Figure S20: Schematic overview of performed chromatographic analyses. (**1**) **Configurational assignment** was based on calculated ECD spectra of (*S*,*S*)-configured pyrroloindoline substrates, experimental ECD spectra and chiral HPLC-UV-CD traces of racemic pyrroloindoline substrates. This allowed configurational assignment to individual enantiomer's retention times (*Rt*) by comparison of peak polarity. (**2**) **Chiral HPLC analysis** of racemic pyrroloindoline substrates and of analytical scale chemical methylation to obtain chiral retention times and experimental UV-spectra of substrates and products. (**3**) **Analysis of kinetic resolution samples.** Chiral HPLC-UV analysis of enzymatic reaction samples (kinetic resolution of racemic starting material) containing enantio-enriched substrates and products. Comparison hereof to chiral HPLC-UV-CD data from (**1**) allowed determination of the stereo-preference of *Sg*PsmC. Additionally, achiral HPLC-UV-MS analysis of racemic starting material and reaction samples (after 0 h and 21 h reaction time) were analysed and product formation cross-validated based on m/z values, and retention times and UV spectra from (**2**).

Configuration Analysis: *rac*-9

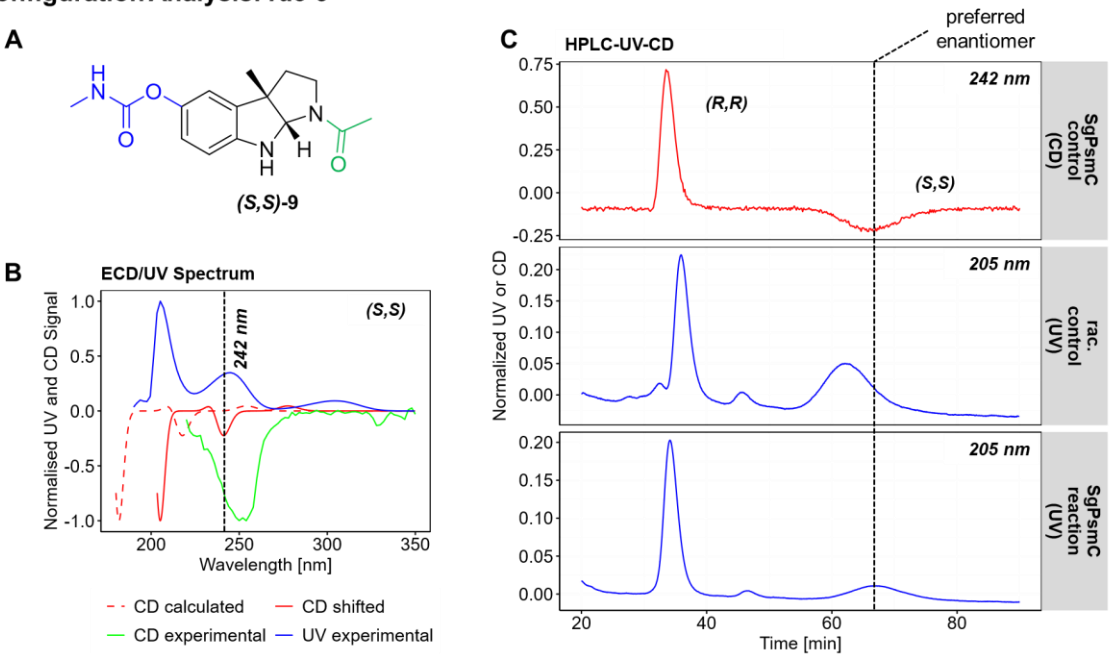

Figure S21a: Results overview of the configurational analysis of the conversion of the racemic substrate ***rac*-9**. Here, the top HPLC-CD trace belongs to the same reaction sample of *Sg*PsmC used for the bottom UV-trace. ***rac*-9** was partially converted by *Sg*PsmC and the remaining substrates were separated *via* chiral HPLC. By comparing the polarity of the two enantiomers' HPLC-CD peaks with the calculated ECD spectrum, the (*R,R*)-configuration can be assigned to the first, and the (*S,S*)-configuration to the second eluted enantiomer. During the kinetic resolution, the second eluted enantiomer HPLC-UV peak decreases, indicating a stereo-preference for **(*S,S*)-9**. Reaction conditions: 4.3 µM *Sg*PsmC, 2 mM SAM, 0.5 mM pyrroloindoline; 35 °C, 700 rpm, 120 min reaction time.

Chiral HPLC Analysis: *rac*-9

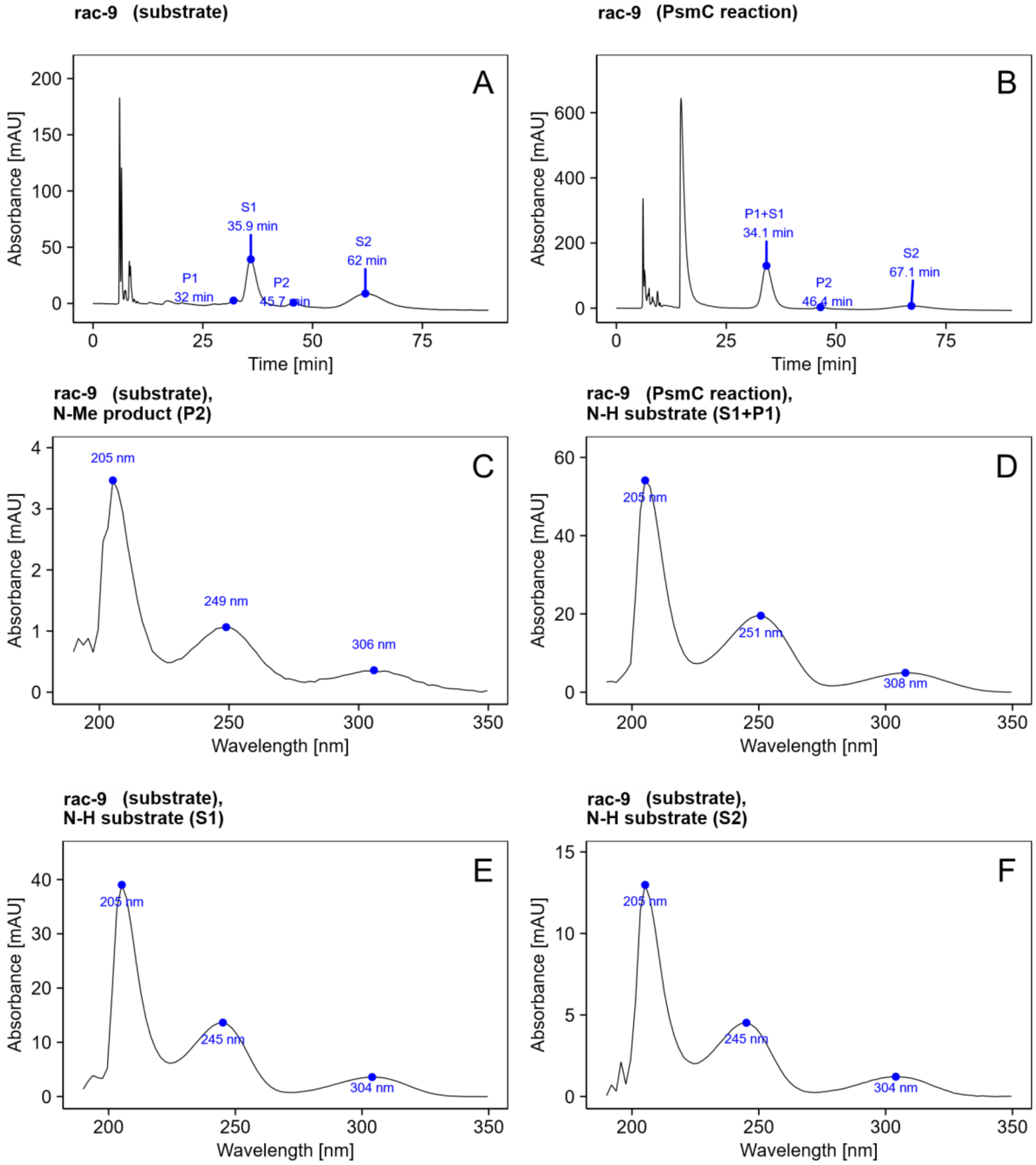

Figure S21b: Chiral HPLC analysis of ***rac*-9**. The substrate sample contained traces of the target *N*-methylated product. (**A**) HPLC-UV trace of ***rac*-9**. (**B**) HPLC-UV trace of the enzyme catalysed *N*-methylation. Reaction conditions: 4.3 µM *Sg*PsmC, 2 mM SAM, 0.5 mM pyrroloindoline; 35 °C, 700 rpm, 120 min reaction time. (**C**) UV spectrum of substrate S1. (**D**) UV spectrum of substrate S2. (**E**) UV spectrum of product P2. (**F**) UV spectrum of product P1. Elution time overlaps with substrate S1.

Achiral HPLC-UV-MS Analysis: *rac*-9

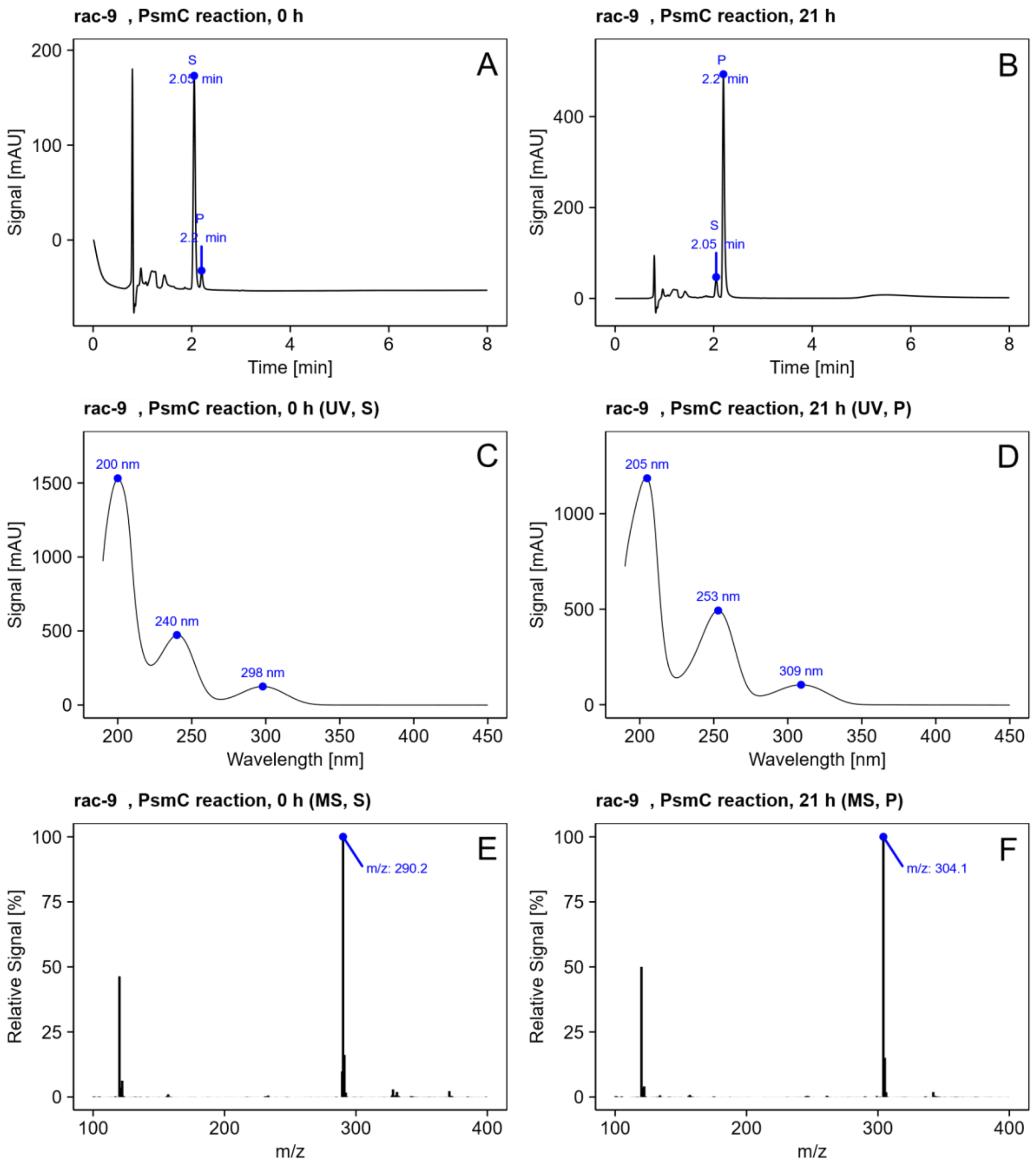

Figure S21c: Achiral LC-MS analysis of ***rac*-9** converted by *Sg*PsmC after 0 h and 21 h reaction time. The racemic substrate showed traces of the target *N*-methylated product. (**A**) HPLC-UV trace after 0 h reaction time. (**B**) HPLC-UV trace after 21 h reaction time. (**C**) UV spectrum of substrate peak 'S' after 0 h reaction time. (**D**) UV spectrum of product peak 'P' after 21 h reaction time. (**E**) MS spectrum of substrate peak 'S' after 0 h reaction time. (**F**) MS spectrum of product peak 'P' after 21 h reaction time.

Configuration Analysis: *rac*-10

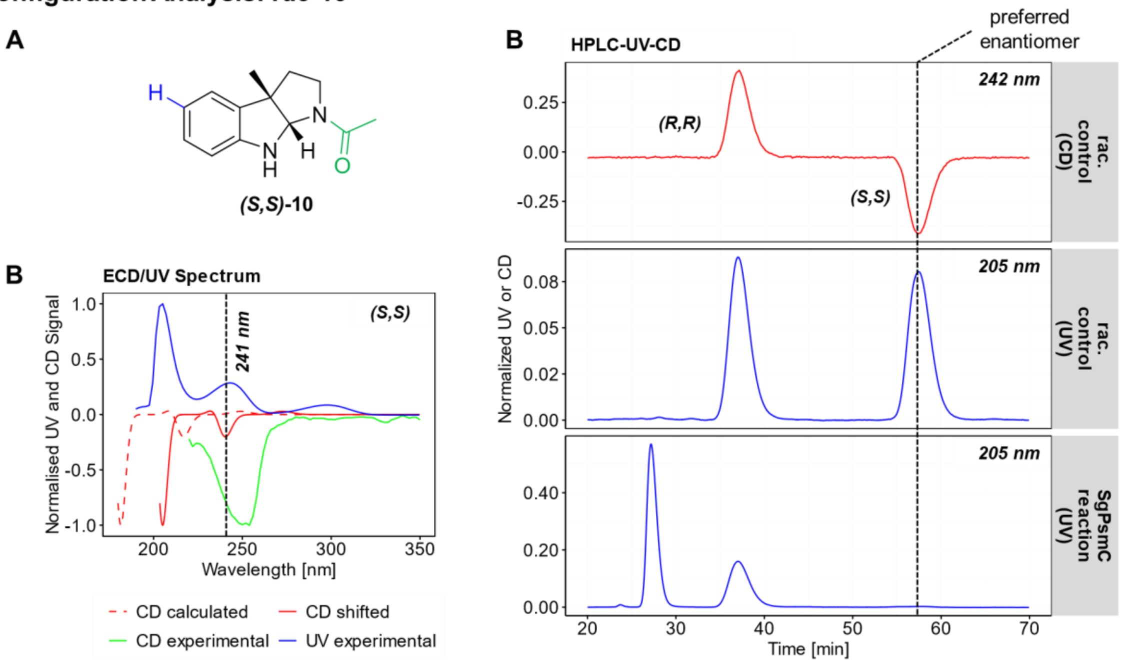

Figure S22a: Results overview of the configurational analysis of the conversion of the racemic substrate ***rac*-10**. By comparing the polarity of the two enantiomers' HPLC-CD peaks with the calculated ECD spectrum, the (*R,R*)-configuration can be assigned to the first, and the (*S,S*)-configuration to the second eluted enantiomer. During the kinetic resolution using *Sg*PsmC, the second eluted enantiomer HPLC-UV peak decreases, indicating a stereo-preference for **(*S,S*)-10**. Reaction conditions: 10 µM *Sg*PsmC, 2 mM SAM, 1 mM pyrroloindoline; 35 °C, 700 rpm, 4 h reaction time.

Chiral HPLC Analysis: *rac*-10

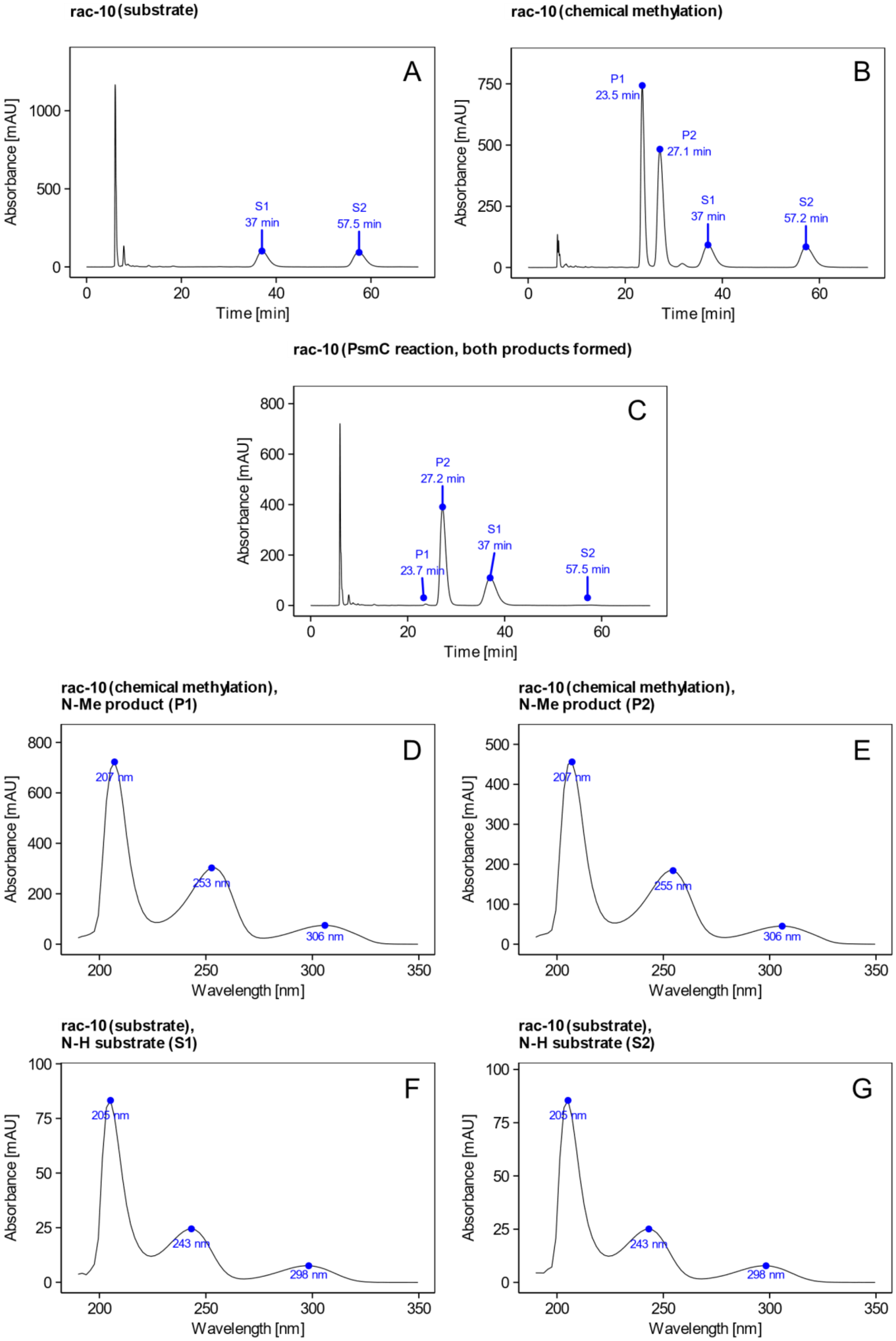

Figure S22b: Chiral HPLC analysis of ***rac*-10**. (**A**) HPLC-UV trace of ***rac*-10**. (**B**) HPLC-UV trace of chemical *N*-methylation. (**C**) HPLC-UV trace of the enzyme catalysed reaction where both product enantiomers could be observed. Reaction conditions: 10 µM *Sg*PsmC, 2 mM SAM, 1 mM pyrroloindoline; 35 °C, 700 rpm, 4 h reaction time. (**D**) UV spectrum of product P1. (**E**) UV spectrum of product P2. (**F**) UV spectrum of substrate S1. (**G**) UV spectrum of substrate S2.

Achiral HPLC-UV-MS Analysis: *rac*-10

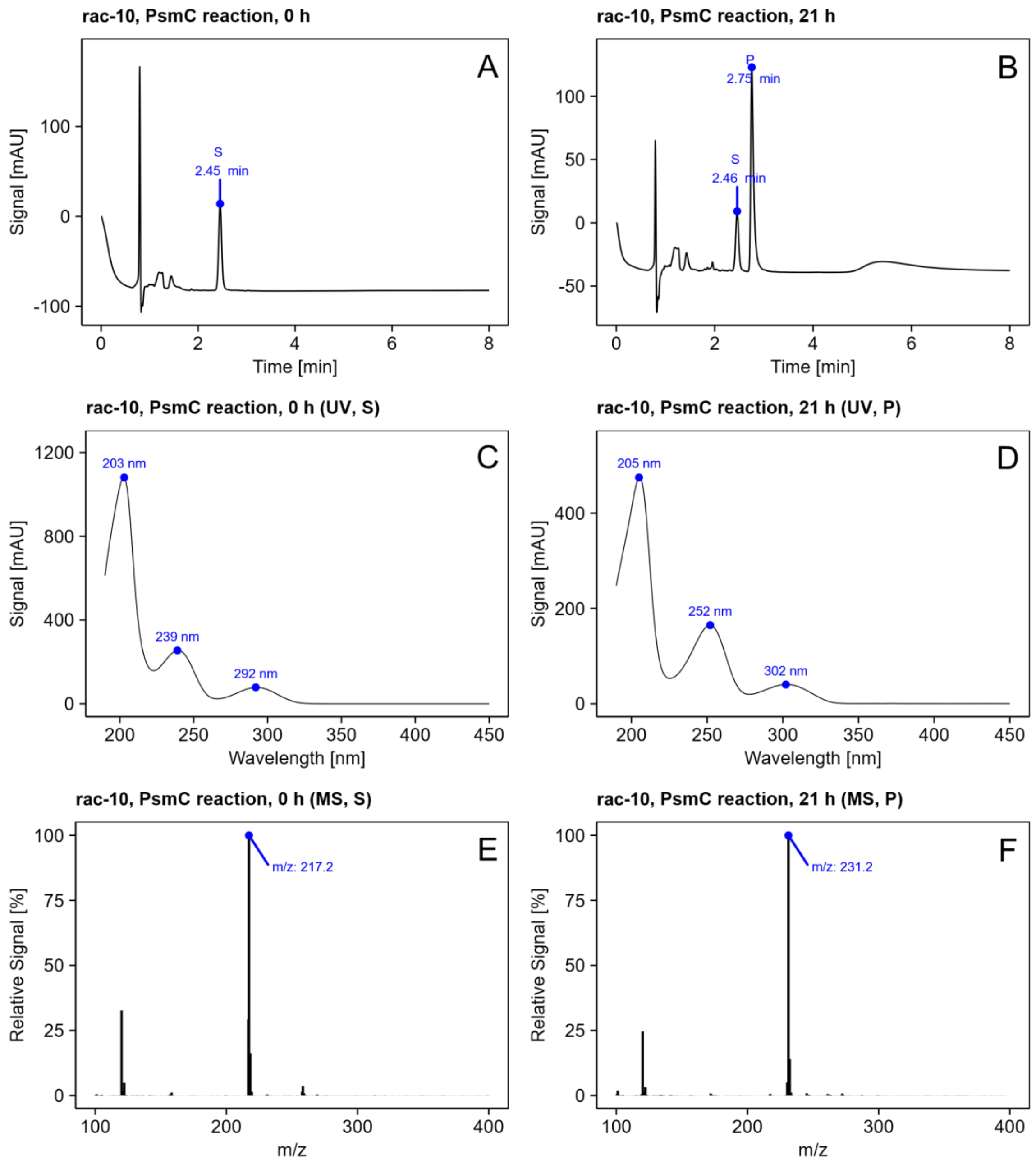

Figure S22c: Achiral LC-MS analysis of ***rac*-10** converted by *Sg*PsmC after 0 h and 21 h reaction time. (**A**) HPLC-UV trace after 0 h reaction time. (**B**) HPLC-UV trace after 21 h reaction time. (**C**) UV spectrum of substrate peak 'S' after 0 h reaction time. (**D**) UV spectrum of product peak 'P' after 21 h reaction time. (**E**) MS spectrum of substrate peak 'S' after 0 h reaction time. (**F**) MS spectrum of product peak 'P' after 21 h reaction time.

Chiral HPLC Analysis: *rac*-11

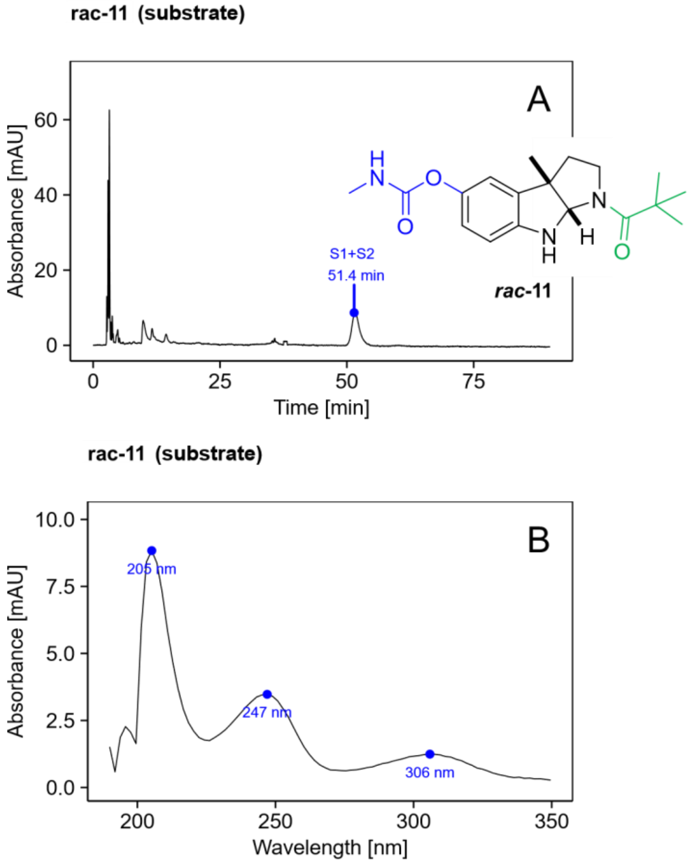

Figure S23a: Chiral HPLC analysis of ***rac*-11**. As the substrate was not converted by *Sg*PsmC, no further chiral HPLC analysis was performed. (**A**) HPLC-UV trace of ***rac*-11**. (**B**) UV spectrum of ***rac*-11**.

Achiral HPLC-UV-MS Analysis: *rac*-11

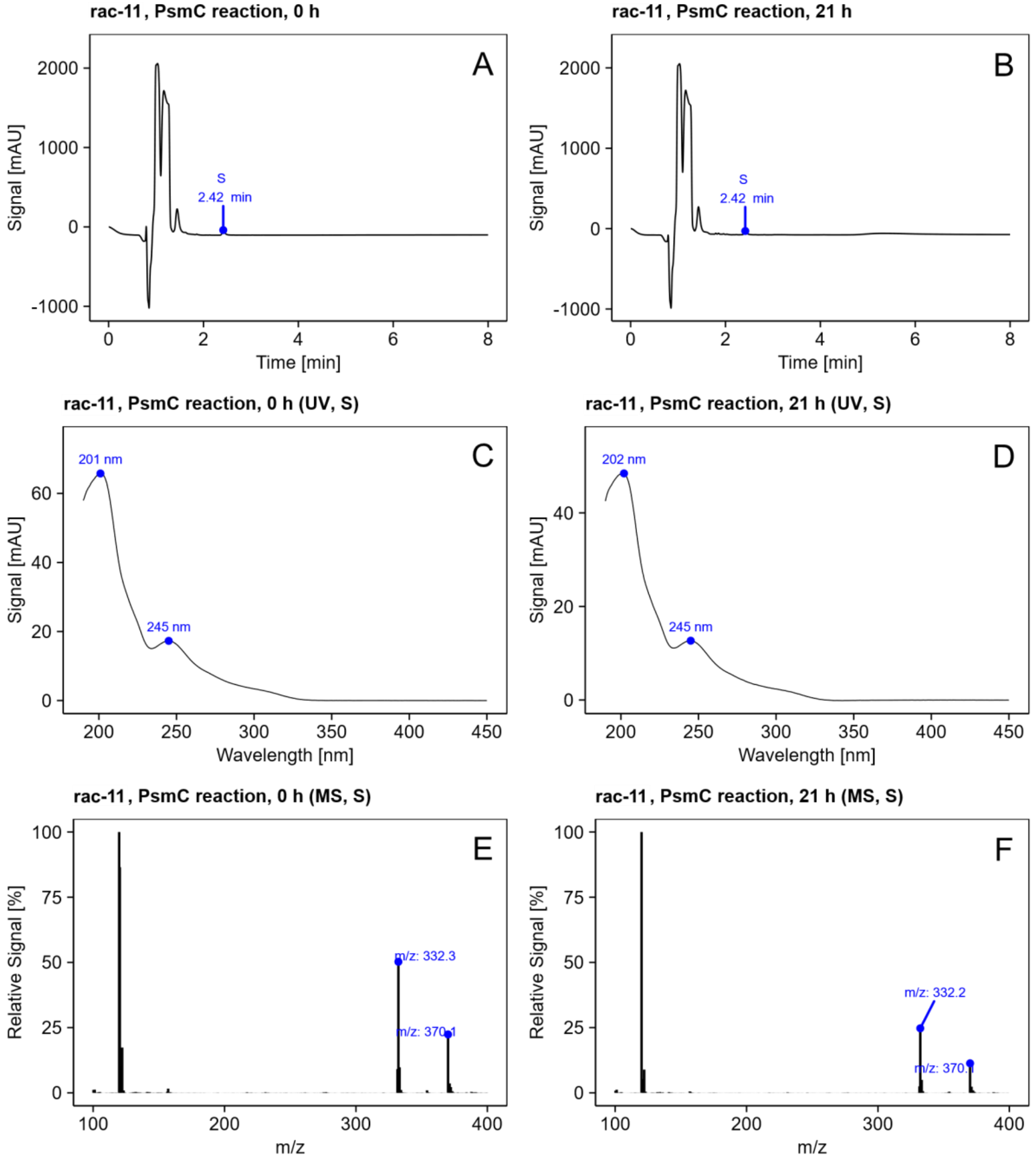

Figure S23b: Achiral LC-MS analysis of reactions samples for the *N*-methylation of ***rac*-11** by *Sg*PsmC after 0 h and 21 h reaction time. The substrate was not converted by *Sg*PsmC, which was also validated by monitoring the formation of SAH using achiral RP-HPLC-UV. The additional MS signal at 370.1 m/z may be an MS adduct. (**A**) HPLC-UV trace after 0 h reaction time. (**B**) HPLC-UV trace after 21 h reaction time. (**C**) UV spectrum of substrate peak 'S' after 0 h reaction time. (**D**) UV spectrum of substrate peak 'S' after 21 h reaction time. (**E**) MS spectrum of substrate peak 'S' after 0 h reaction time. (**F**) MS spectrum of substrate peak 'S' after 21 h reaction time.

Configuration Analysis: *rac*-12

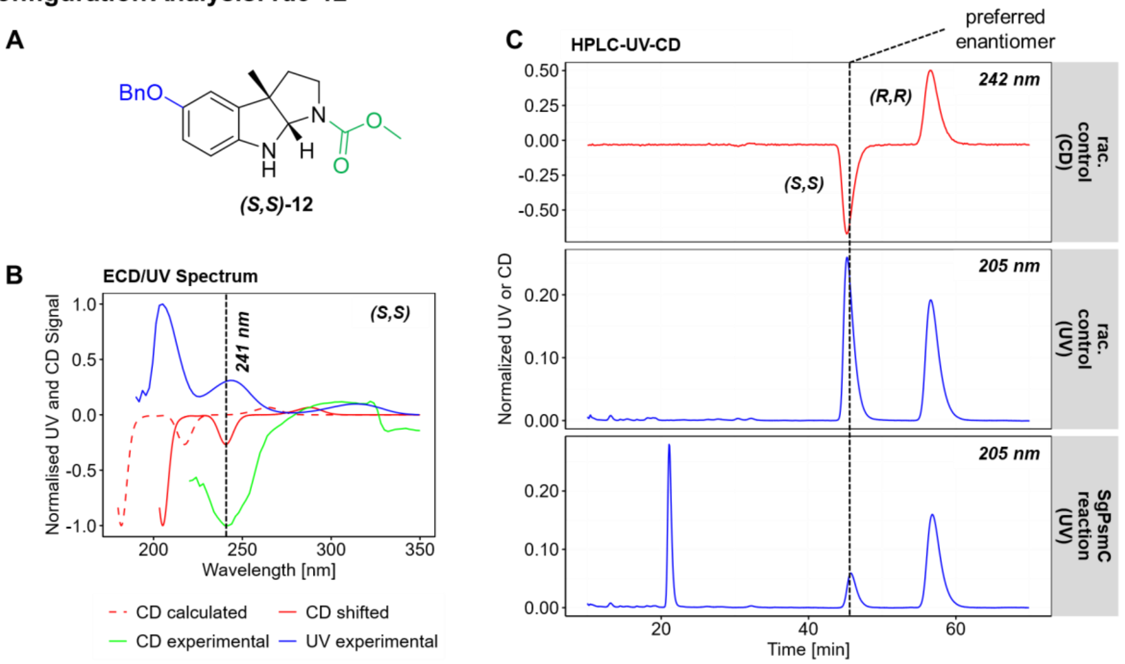

Figure S24a: Results overview of the configurational analysis of the conversion of the racemic substrate ***rac*-12**. By comparing the polarity of the two enantiomers' HPLC-CD peaks with the calculated ECD spectrum, the (*S,S*)-configuration can be assigned to the first, and the (*R,R*)-configuration to the second eluted enantiomer. During the kinetic resolution using *Sg*PsmC, the first eluted enantiomer HPLC-UV peak decreases, indicating a stereo-preference for **(*S,S*)-12**. Reaction conditions: 0.05 U/mL (1% v/v salted-out catalyst) *Sg*PsmC (activity against 5-Me indoline), 2 mM SAM, 1 mM pyrroloindoline; 35 °C, 700 rpm, 3 h reaction time.

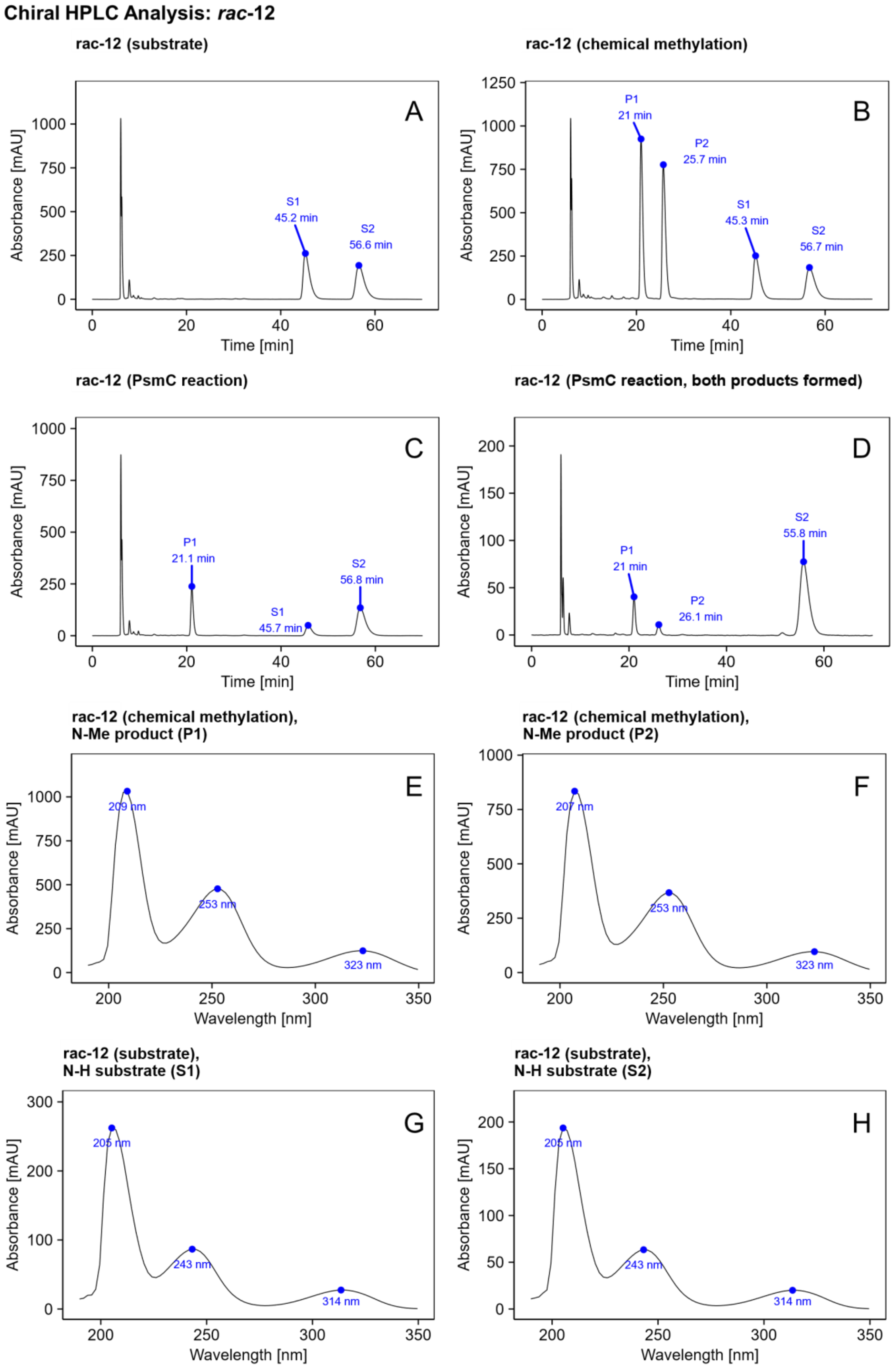

Figure S24b: Chiral HPLC analysis of ***rac*-12**. (**A**) HPLC-UV trace of ***rac*-12**. (**B**) HPLC-UV trace of chemical *N*-methylation. (**C**) HPLC-UV trace of the enzyme catalysed reaction. Reaction conditions: 0.05 U/mL (1% v/v salted-out catalyst) *Sg*PsmC (activity against 5-Me indoline), 2 mM SAM, 1 mM pyrroloindoline; 35 °C, 700 rpm, 3 h reaction time. (**D**) HPLC-UV trace of the enzyme catalysed reaction where both product enantiomers could be observed. Reaction conditions: 0.32 U/mL (1.6% v/v) *Sg*PsmC (activity against 5-Me indoline), 2 mM SAM, 1 mM pyrroloindoline; 35 °C, 700 rpm, 24 h reaction time. (**E**) UV spectrum of product P1. (**F**) UV spectrum of product P2. (**G**) UV spectrum of substrate S1. (**H**) UV spectrum of substrate S2.

Achiral HPLC-UV-MS Analysis: *rac*-12

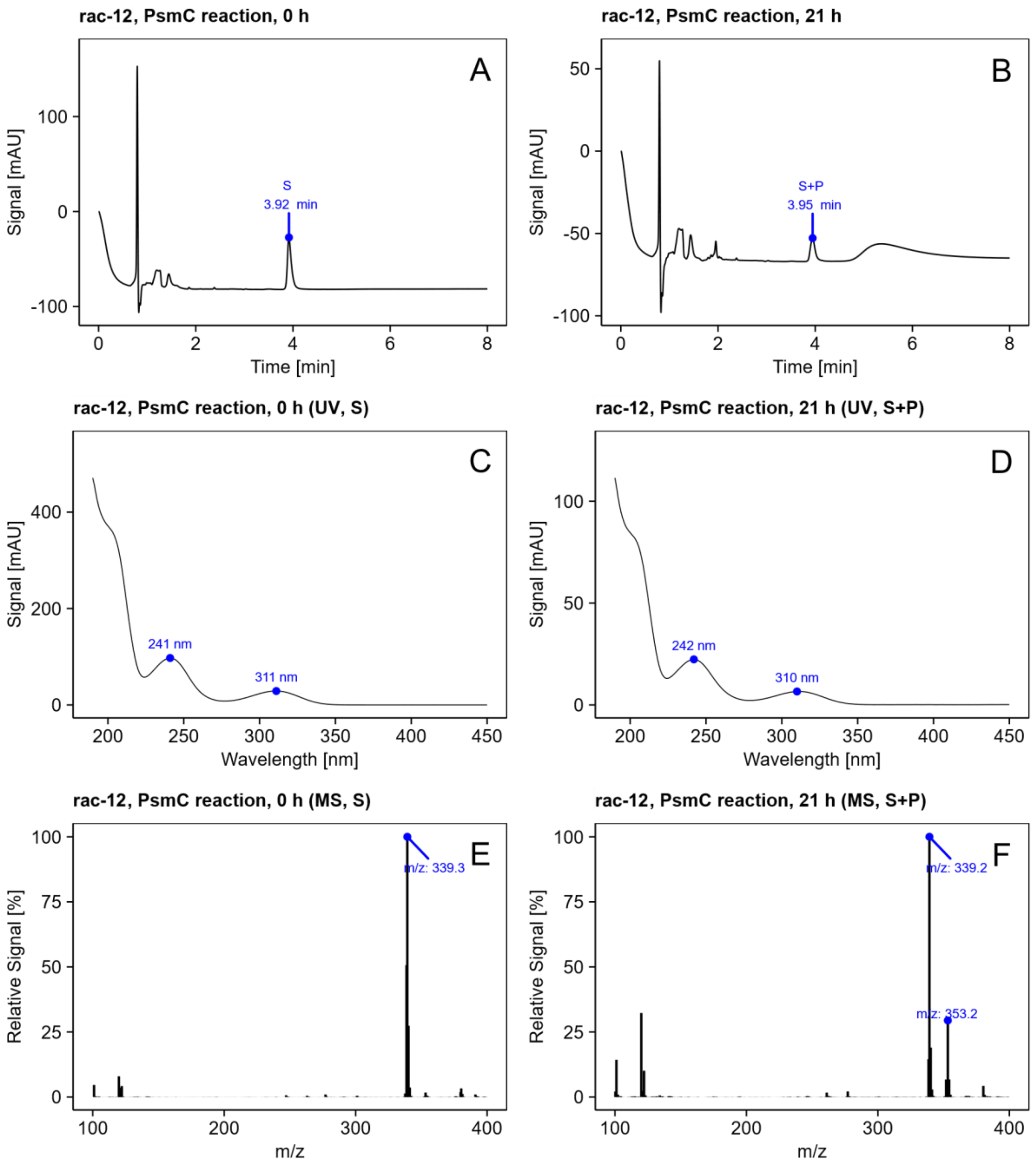

Figure S24c: Achiral LC-MS analysis of ***rac*-12** converted by *Sg*PsmC after 0 h and 21 h reaction time. Separation of substrate and product was not achieved on the achiral reverse phase. Conversion was validated by an MS shift of +14 m/z. (**A**) HPLC-UV trace after 0 h reaction time. (**B**) HPLC-UV trace after 21 h reaction time. (**C**) UV spectrum of substrate peak ‘S’ after 0 h reaction time. (**D**) UV spectrum of non-separated substrate and product peak ‘S+P’ after 21 h reaction time. (**E**) MS spectrum of substrate peak ‘S’ after 0 h reaction time. (**F**) MS spectrum of non-separated substrate and product peak ‘S+P’ after 21 h reaction time.

Configuration Analysis: *rac*-13

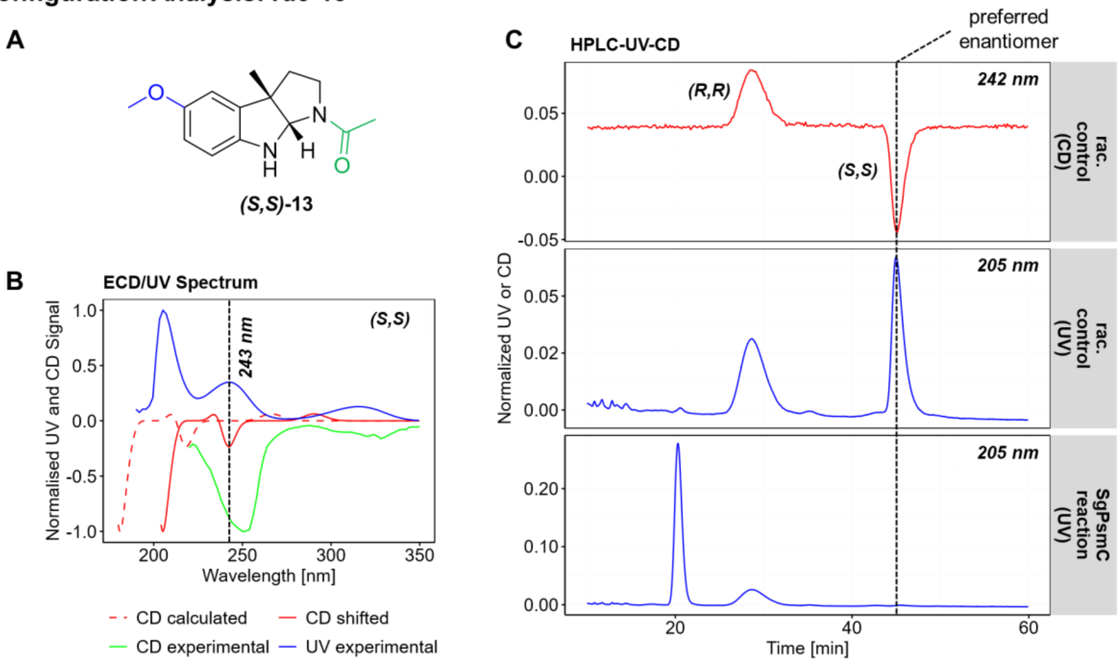

Figure S25a: Results overview of the configurational analysis of the conversion of the racemic substrate ***rac*-13**. By comparing the polarity of the two enantiomers' HPLC-CD peaks with the calculated ECD spectrum, the (*R,R*)-configuration can be assigned to the first, and the (*S,S*)-configuration to the second eluted enantiomer. During the kinetic resolution using *Sg*PsmC, the second eluted enantiomer HPLC-UV peak decreases, indicating a stereo-preference for **(*S,S*)-13**. Reaction conditions: 5 µM *Sg*PsmC, 2 mM SAM, 1 mM pyrroloindoline; 35 °C, 700 rpm, 30 min reaction time.

Chiral HPLC Analysis: *rac*-13

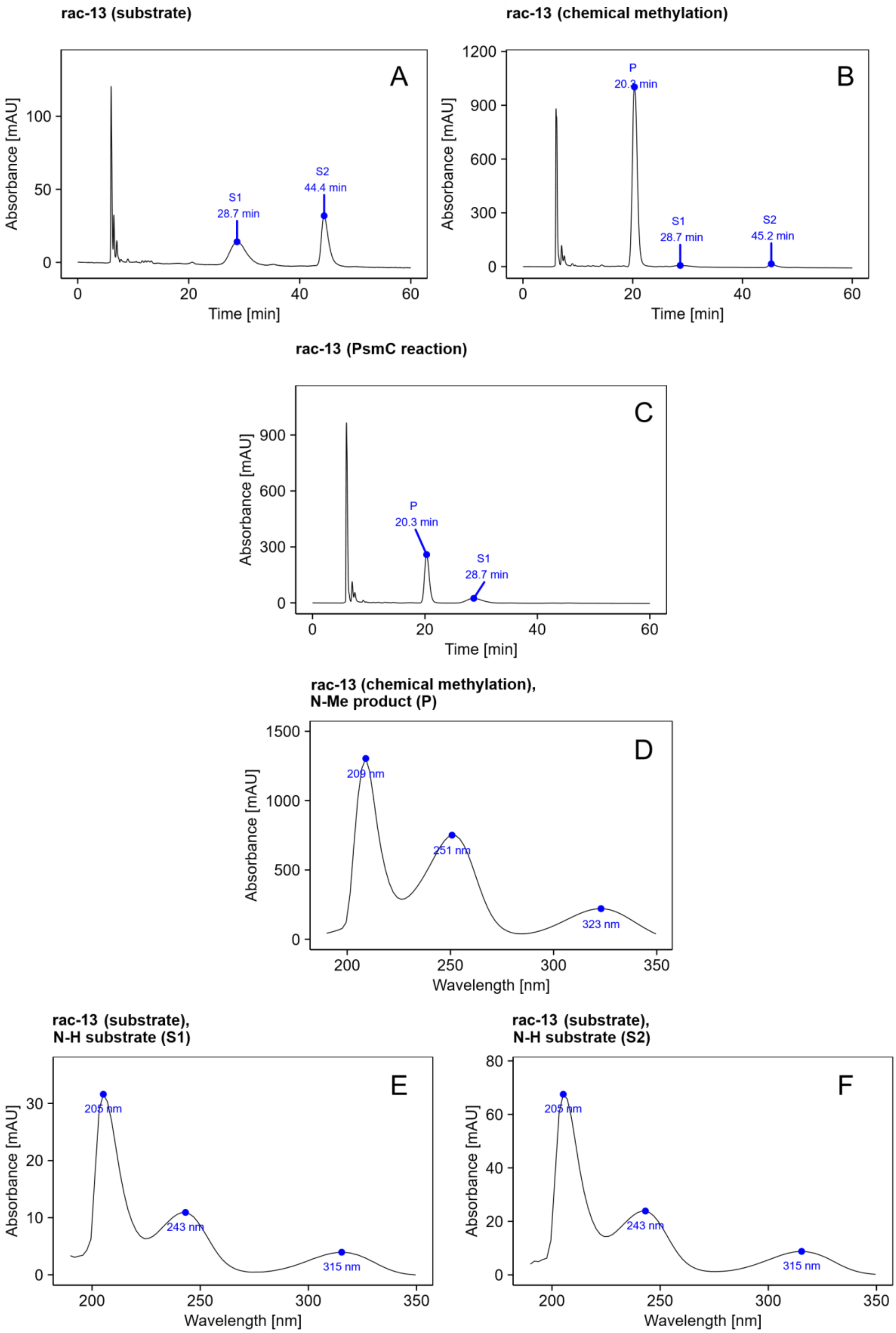

Figure S25b: Chiral HPLC analysis of ***rac*-13**. Chiral separation of *N*-methylated products was not accomplished. E-value determination was, therefore, done using ee_S and conversion values. (**A**) HPLC-UV trace of ***rac*-13**. (**B**) HPLC-UV trace of chemical *N*-methylation. (**C**) HPLC-UV trace of the enzyme catalysed reaction. Reaction conditions: 5 µM *Sg*PsmC, 2 mM SAM, 1 mM pyrroloindoline; 35 °C, 700 rpm, 30 min reaction time. (**D**) UV spectrum of product ***rac*-13-Me**. (**E**) UV spectrum of substrate S1. (**F**) UV spectrum of substrate S2.

Achiral HPLC-UV-MS Analysis: *rac*-13

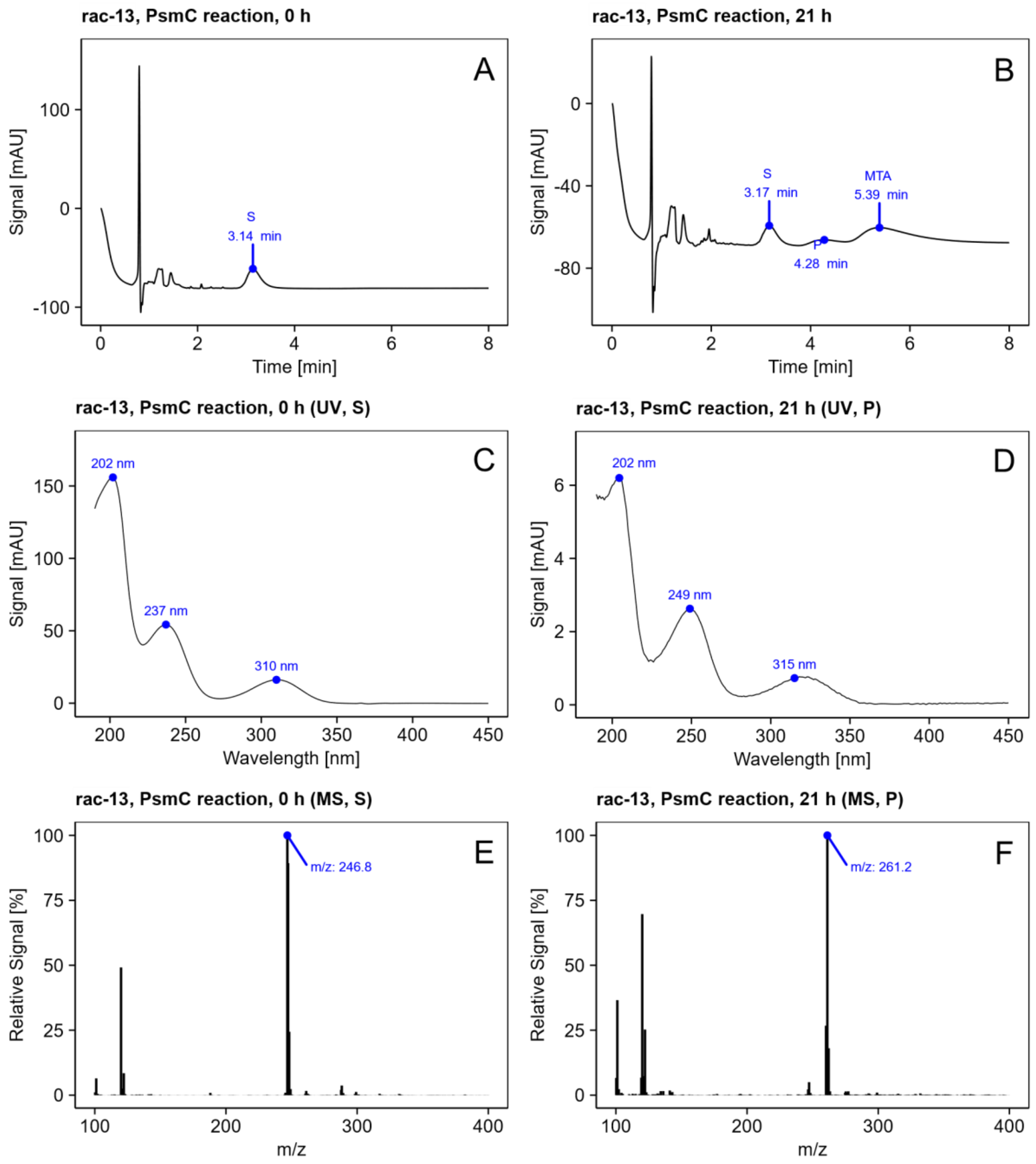

Figure S25c: Achiral LC-MS analysis of ***rac*-13** converted by *Sg*PsmC after 0 h and 21 h reaction time. (**A**) HPLC-UV trace after 0 h reaction time. (**B**) HPLC-UV trace after 21 h reaction time. (**C**) UV spectrum of substrate peak 'S' after 0 h reaction time. (**D**) UV spectrum of product peak 'P' after 21 h reaction time. (**E**) MS spectrum of substrate peak 'S' after 0 h reaction time. (**F**) MS spectrum of product peak 'P' after 21 h reaction time. MTA: presumably 5'-methyl-thio-5'-desoxyadenosine.

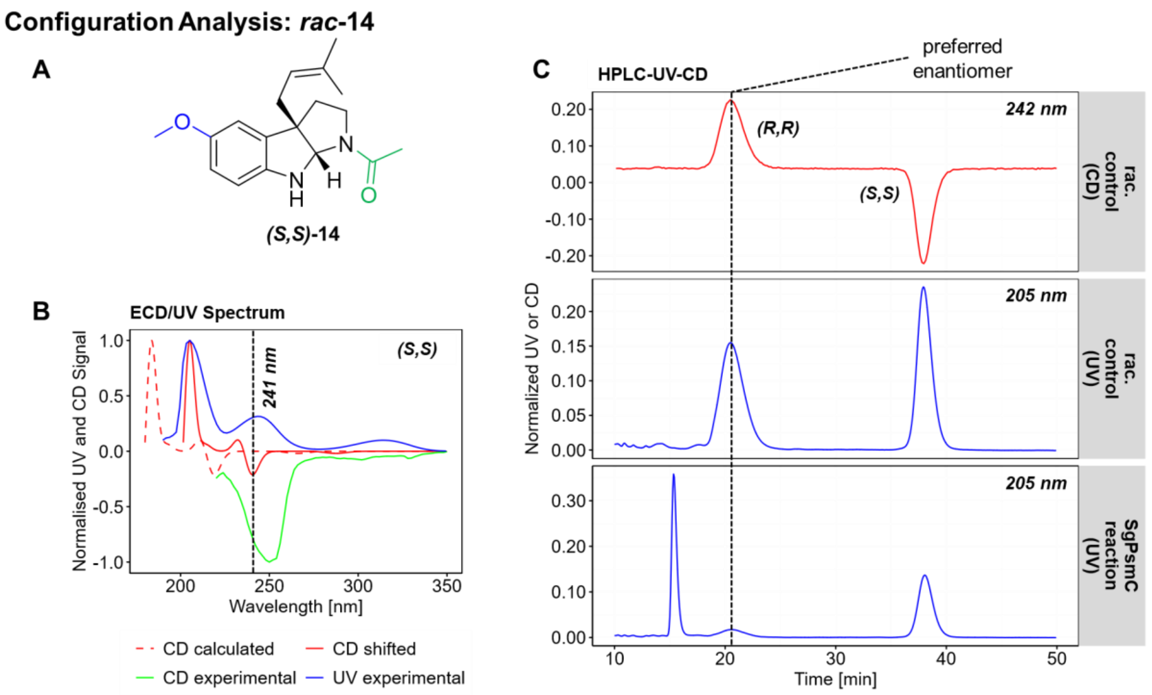

Figure S26a: Results overview of the configurational analysis of the conversion of the racemic substrate ***rac*-14**. By comparing the polarity of the two enantiomers' HPLC-CD peaks with the calculated CD spectrum, the (*R,R*)-configuration can be assigned to the first, and the (*S,S*)-configuration to the second eluted enantiomer. During the kinetic resolution using *Sg*PsmC, the first eluted enantiomer HPLC-UV peak decreases, indicating a stereo-preference for **(*R,R*)-14**. From the tested substrates, this is only case where *Sg*PsmC does not show a preference for the (*S,S*)-configured scaffold. Reaction conditions: 20 µM *Sg*PsmC, 2 mM SAM, 1 mM pyrroloindoline; 35 °C, 700 rpm, 24 h reaction time.

Configuration Analysis: *rac*-14 (including *N*-methylated product)

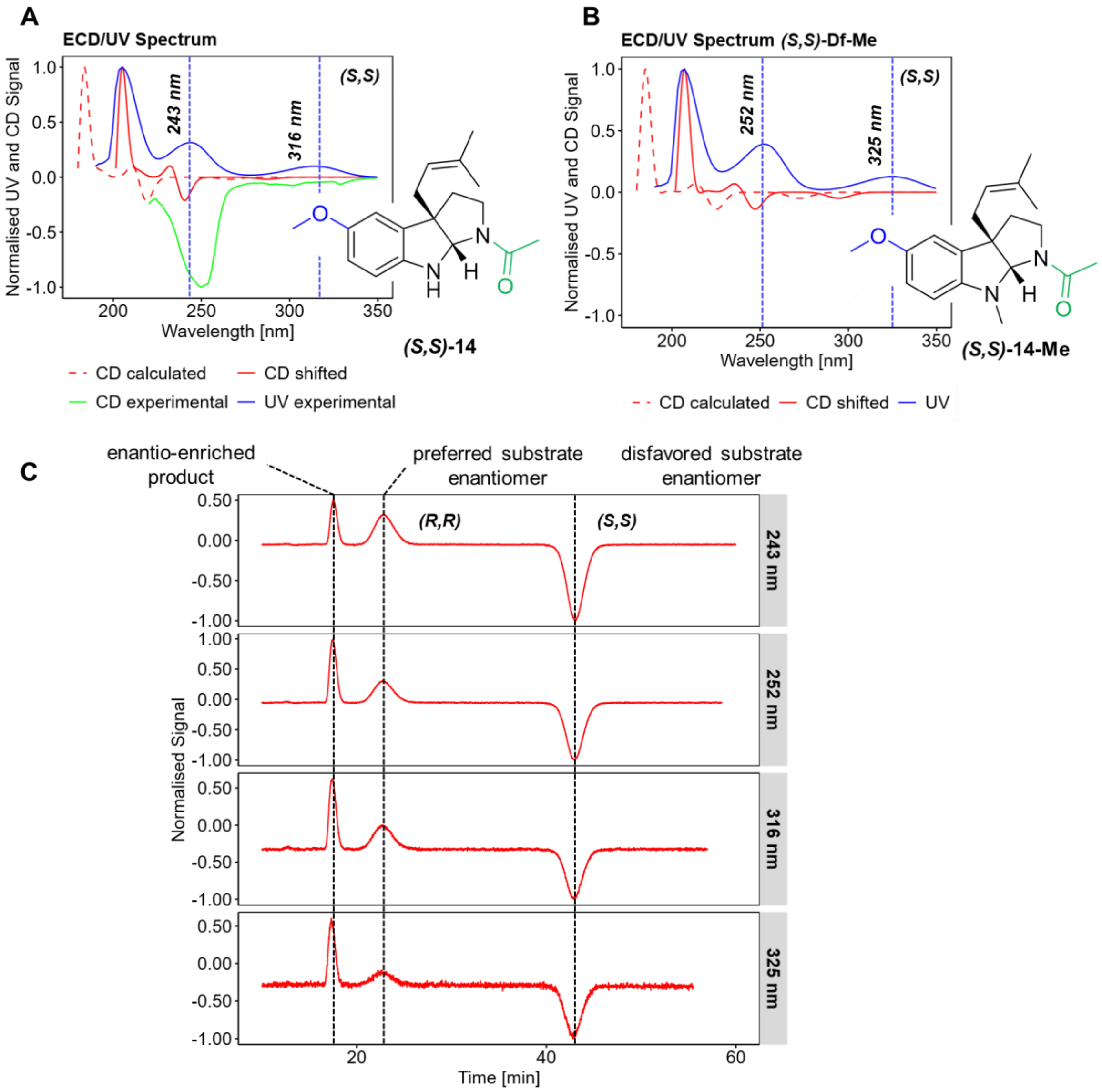

Figure S26b: Results overview of the configurational analysis of the conversion of the racemic substrate ***rac*-14** in order to validate the enantio-preference of *Sg*PsmC. **<u>(A, B):</u>** Experimental UV-spectra (blue), calculated ECD spectra (red, dashed) and shifted calculated ECD spectra (red, solid) for **(*S,S*)-14** and **(*S,S*)-14-Me**. The UV-maxima at which negative CD peaks were predicted and at which the HPLC-CD traces were recorded are marked (blue, dashed). **<u>(C):</u>** HPLC-CD traces for the *Sg*PsmC catalysed *N*-methylation of the *C3a*-prenylated pyrroloindoline ***rac*-14**. The same sample was injected multiple times and the monitored at the UV-maxima of ***rac*-14** (243 nm and 316 nm) and of the *N*-methylated product ***(S,S)*-14-Me** (252 nm and 325 nm). By comparing the polarity of the two substrate enantiomers' and enantio-enriched product's HPLC-CD peaks with the calculated CD spectra, the (*R,R*)-configuration can be assigned to the first, and the (*S,S*)-configuration to the second eluted substrate enantiomer. During the kinetic resolution using *Sg*PsmC, the first eluted substrate enantiomer HPLC-UV peak decreases, indicating a stereo-preference for **(*R,R*)-14**. At the same time, the enantio-enriched product **14-Me** shows a positive HPLC-CD peak, thus implying the same stereo-preference. From the tested substrates, this is the only case where *Sg*PsmC does not show a preference for the (*S,S*)-configured scaffold.

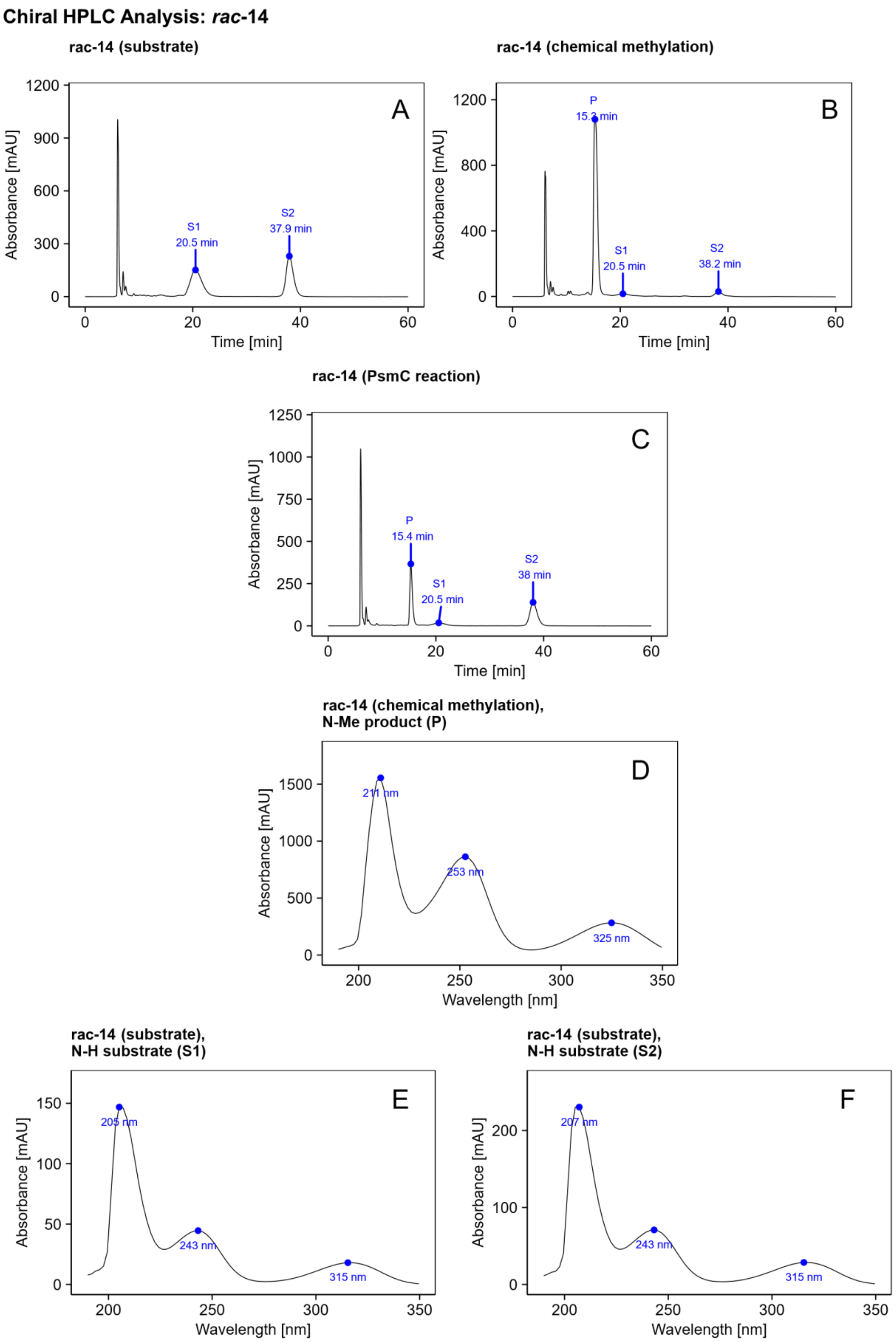

Figure S26c: Chiral HPLC analysis of ***rac*-14**. Chiral separation of *N*-methylated products was not achieved. E-value determination was, therefore, done using ee_S and conversion values. (**A**) HPLC-UV trace of ***rac*-14**. (**B**) HPLC-UV trace of chemical *N*-methylation. (**C**) HPLC-UV trace of the enzyme catalysed reaction. Reaction conditions: 20 µM *Sg*PsmC, 2 mM SAM, 1 mM pyrroloindoline; 35 °C, 700 rpm, 24 h reaction time. (**D**) UV spectrum of product ***rac*-14-Me**. (**E**) UV spectrum of substrate S1. (**F**) UV spectrum of substrate S2.

Achiral HPLC-UV-MS Analysis: ***rac*-14** (0 h reaction time)

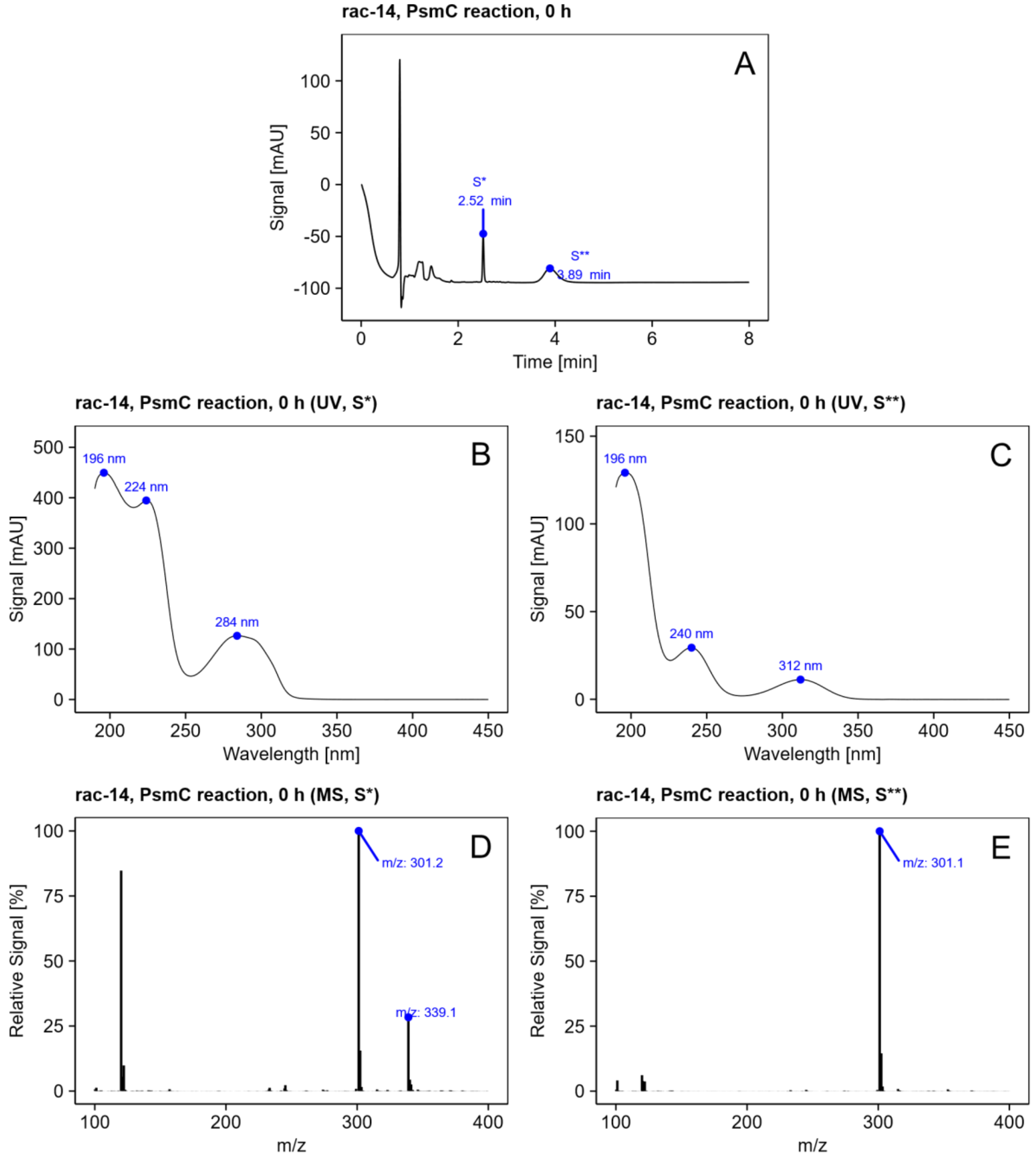

Figure S26d: Achiral LC-MS analysis of ***rac*-14** converted by *Sg*PsmC after 0 h reaction time. See also **Fig. S26e**. Two peaks are visible in the HPLC-UV trace (labelled S* and S** in order of elution). Only the UV spectrum of S** shows the characteristic UV maxima of the pyrroloindoline scaffold. The MS spectra of both S* and S** show the typical substrate signal of 301.1 m/z. The MS spectrum of S* shows an additional signal with an MS shift of +37.9 m/z. During chiral NP-HPLC analysis (see **Fig. S26c**), no similar peak behaviour was observed. (**A**) HPLC-UV trace after 0 h reaction time. (**B**) UV spectrum of substrate peak 'S*' after 0 h reaction time. (**C**) UV spectrum of substrate peak 'S**' after 0 h reaction time. (**D**) MS spectrum of substrate peak 'S*' after 0 h reaction time. (**E**) MS spectrum of substrate peak 'S**' after 0 h reaction time. While 'S**' can be correlated to compound **14**, 'S*' is a chromatographic impurity.

Achiral HPLC-UV-MS Analysis: *rac*-14 (21 h reaction time)

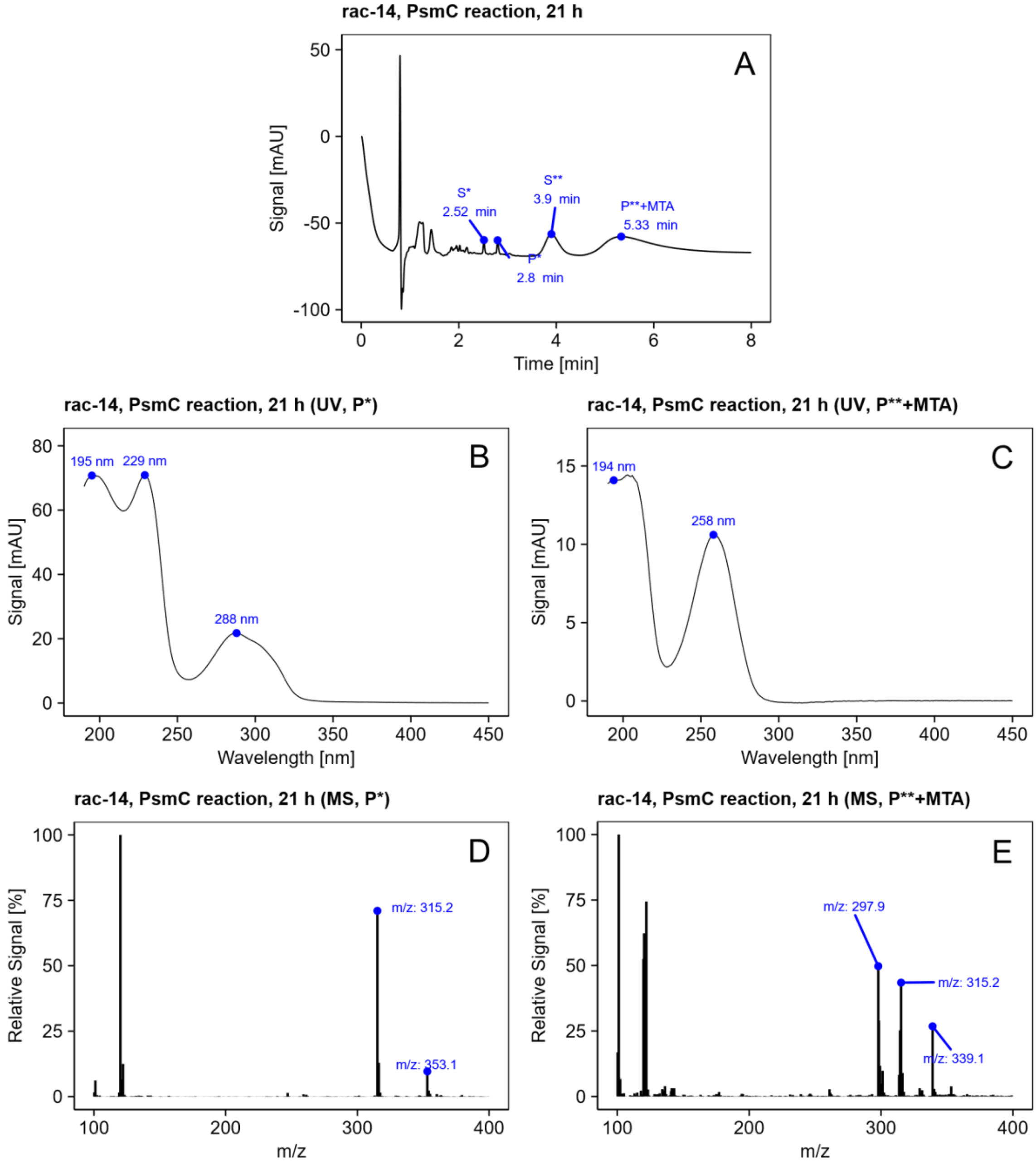

Figure S26e: Achiral LC-MS analysis of ***rac*-14** converted by *Sg*PsmC after 21 h reaction time. See also **Fig. S26d**. Two product HPLC-UV peaks are visible (labelled P* and P** in order of elution). Neither UV spectrum shows the characteristic UV maxima of the pyrroloindoline scaffold. For P** this is likely due to the overlap with presumably MTA. However, P** does show absorbance at 258 nm, close to the 253 nm depicted in **Fig. S26c**. The MS spectra of both P* and P** show the product signal of 315.2 m/z. The MS spectrum of P* shows an additional signal at 353.1 m/z, the MS spectrum of P** at 297.9 m/z (MTA) and 339.1 m/z. Both MS spectra of S* (first eluted peak with m/z signal corresponding to substrate ***rac*-14,** see **Fig. S26d**) and P** show a signal with a mass shift of 37.9 m/z. During chiral NP-HPLC analysis (see **Fig. S26c**), no similar peak behaviour was observed. (**A**) HPLC-UV trace after 21 h reaction time. (**B**) UV spectrum of product peak 'P*' after 21 h reaction time. (**C**) UV spectrum of product peak with overlapping MTA peak 'P**+MTA' after 21 h reaction time. (**D**) MS spectrum of product peak 'P*' after 21 h reaction time. (**E**) MS spectrum of product peak with overlapping MTA peak 'P**+MTA' after 21 h reaction time. While 'P**' can be correlated to compound **14-Me**, 'P*' is a chromatographic impurity.

Chiral HPLC Analysis: *rac*-15

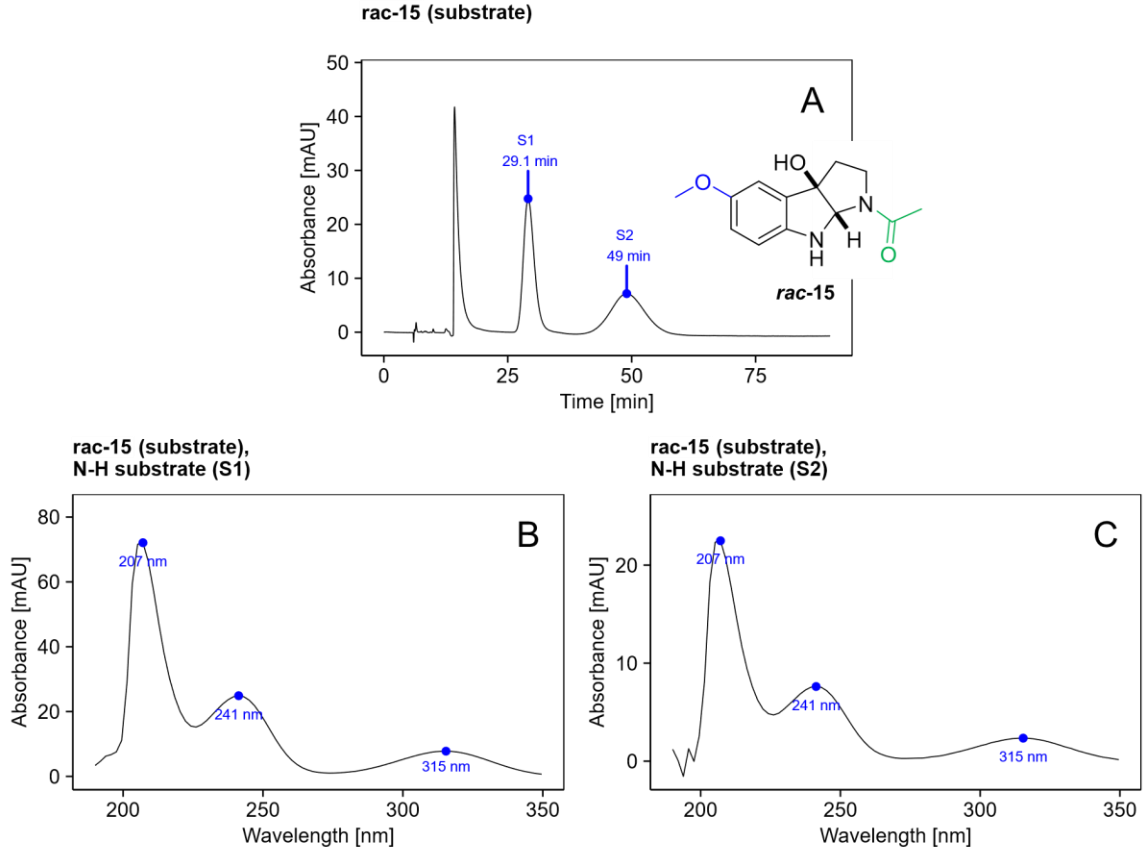

Figure S27a: Chiral HPLC analysis of ***rac*-15**. *N*-methylated products could not be resolved, and no E-value was, therefore, determined. (**A**) HPLC-UV trace of ***rac*-15**. (**B**) UV spectrum of substrate S1. (**C**) UV spectrum of substrate S2.

Achiral HPLC-UV-MS Analysis: *rac*-15

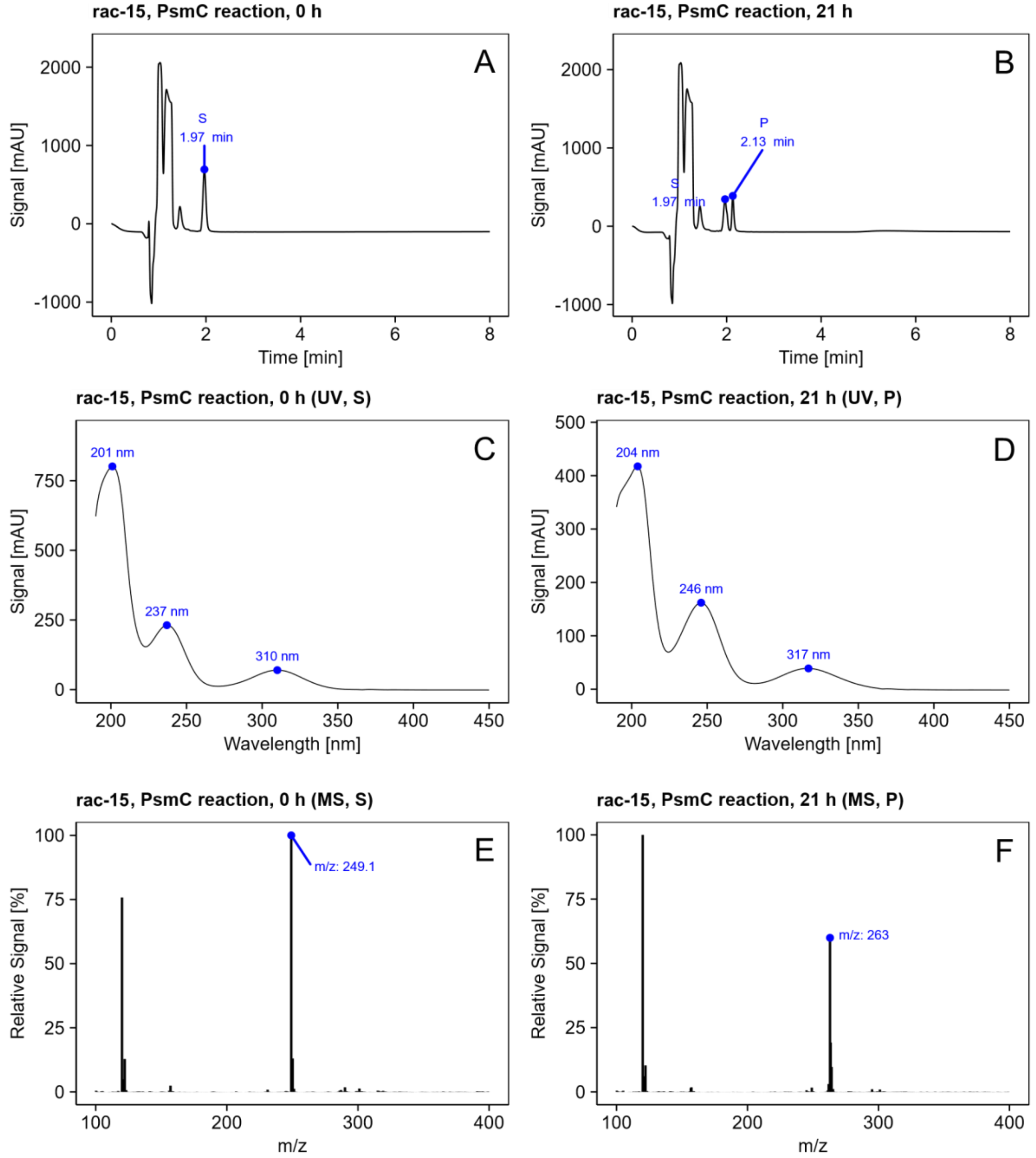

Figure S27b: Achiral LC-MS analysis of ***rac*-15** converted by *Sg*PsmC after 0 h and 21 h reaction time. (**A**) HPLC-UV trace after 0 h reaction time. (**B**) HPLC-UV trace after 21 h reaction time. (**C**) UV spectrum of substrate peak ‘S’ after 0 h reaction time. (**D**) UV spectrum of product peak ‘P’ after 21 h reaction time. (**E**) MS spectrum of substrate peak ‘S’ after 0 h reaction time. (**F**) MS spectrum of product peak ‘P’ after 21 h reaction time.

Configuration Analysis: *rac*-16

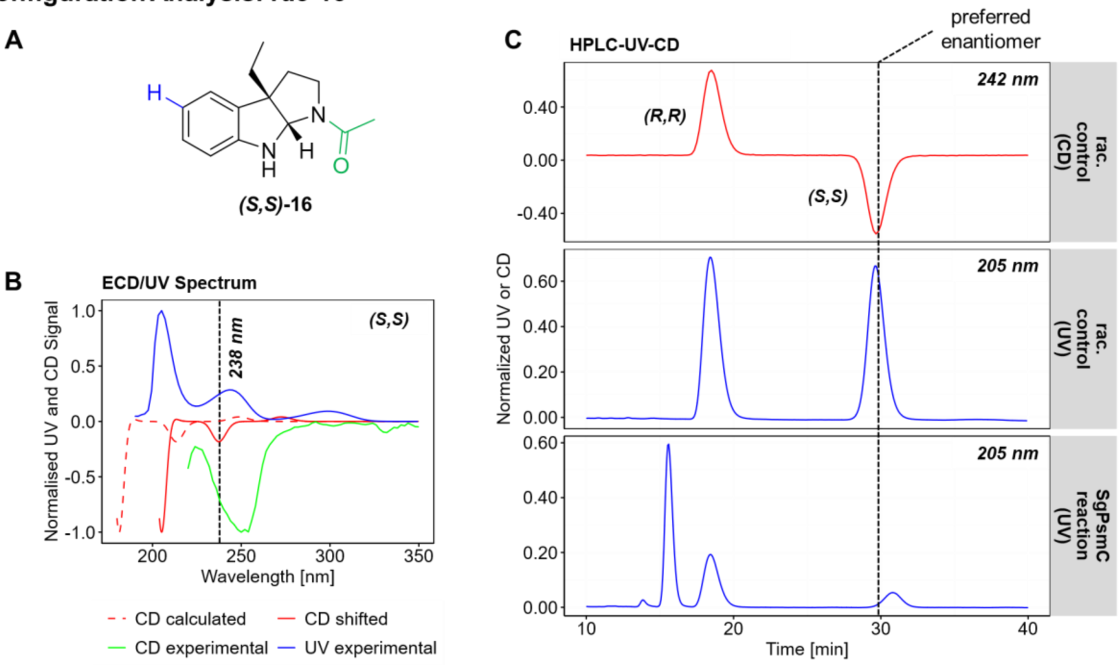

Figure S28a: Results overview of the configurational analysis of the conversion of the racemic substrate ***rac*-16**. By comparing the polarity of the two enantiomers' HPLC-CD peaks with the calculated ECD spectrum, the (*R,R*)-configuration can be assigned to the first, and the (*S,S*)-configuration to the second eluted enantiomer. During the kinetic resolution using *Sg*PsmC, the second eluted enantiomer HPLC-UV peak decreases, indicating a stereo-preference for **(*S,S*)-16**. Reaction conditions: 15 µM *Sg*PsmC, 2 mM SAM, 1 mM pyrroloindoline; 35 °C, 700 rpm, 3 h reaction time.

Chiral HPLC Analysis: *rac*-16

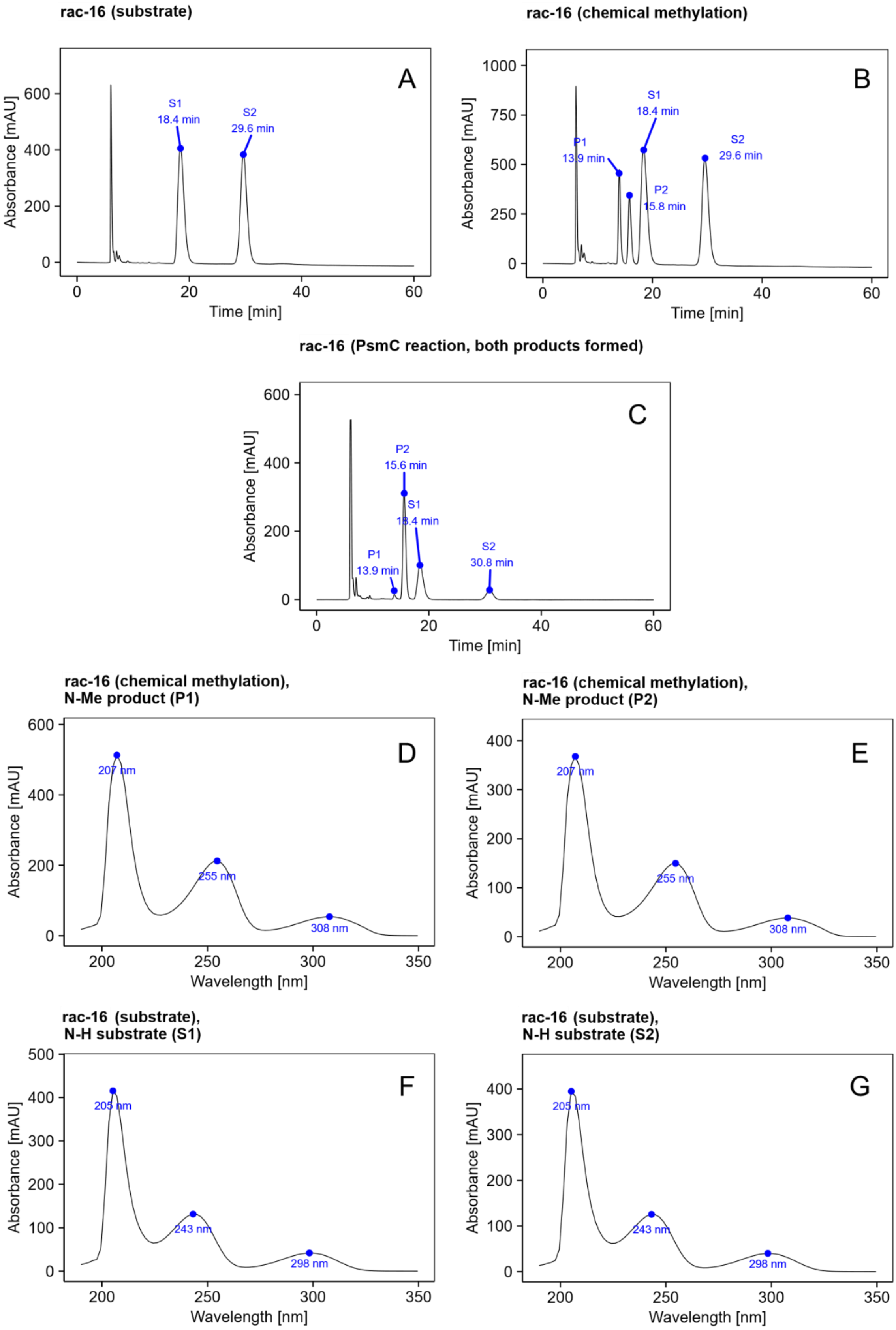

Figure S28b: Chiral HPLC analysis of ***rac*-16**. (**A**) HPLC-UV trace of ***rac*-16**. (**B**) HPLC-UV trace of chemical *N*-methylation. (**C**) HPLC-UV trace of the enzyme catalysed reaction where both product enantiomers were observed. Reaction conditions: 15 µM *Sg*PsmC, 2 mM SAM, 1 mM pyrroloindoline; 35 °C, 700 rpm, 3 h reaction time. (**D**) UV spectrum of product P1. (**E**) UV spectrum of product P2. (**F**) UV spectrum of substrate S1. (**G**) UV spectrum of substrate S1.

Achiral HPLC-UV-MS Analysis: *rac*-16

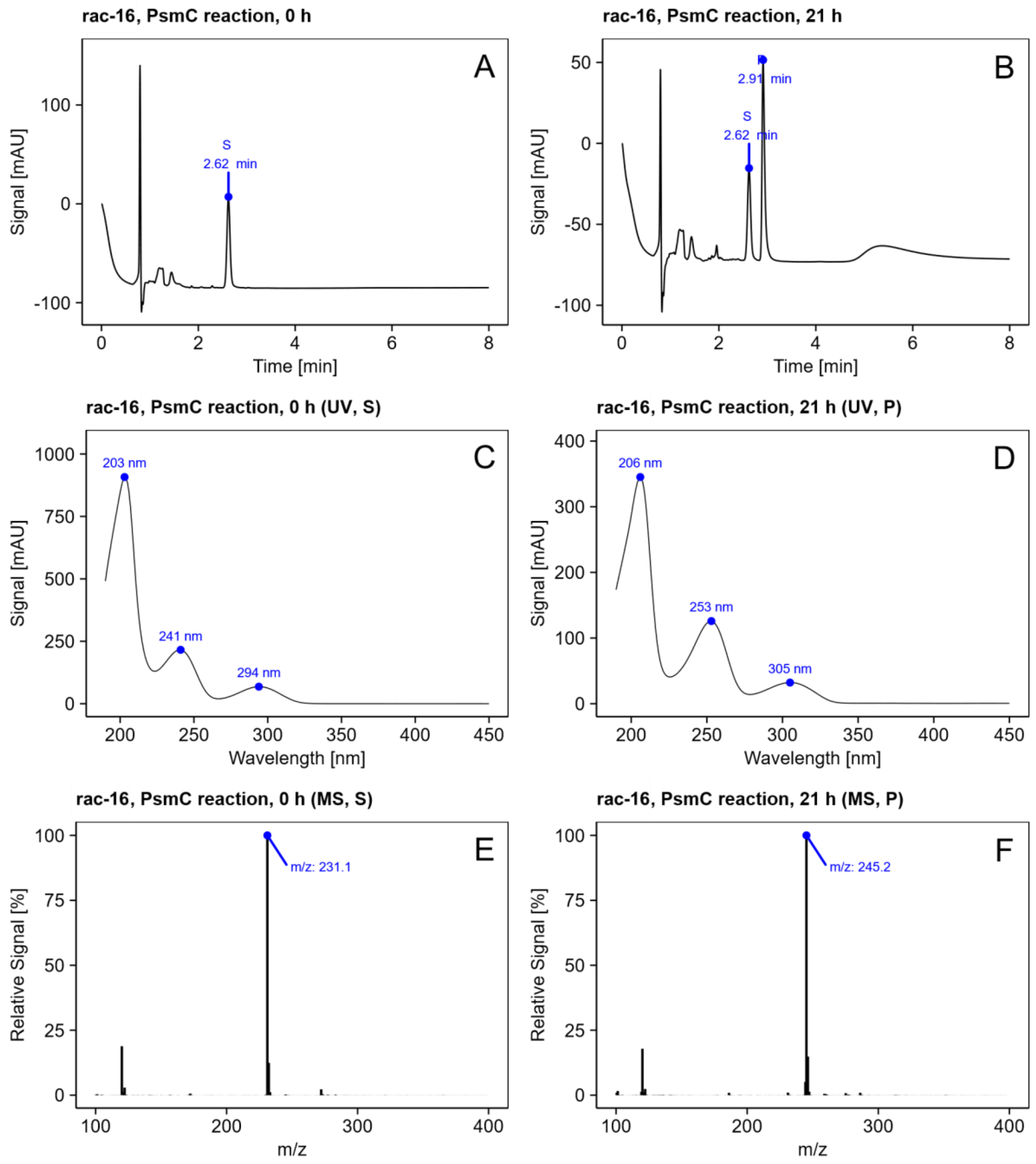

Figure S28c: Achiral LC-MS analysis of ***rac*-16** converted by *Sg*PsmC after 0 h and 21 h reaction time. (**A**) HPLC-UV trace after 0 h reaction time. (**B**) HPLC-UV trace after 21 h reaction time. (**C**) UV spectrum of substrate peak ‘S’ after 0 h reaction time. (**D**) UV spectrum of product peak ‘P’ after 21 h reaction time. (**E**) MS spectrum of substrate peak ‘S’ after 0 h reaction time. (**F**) MS spectrum of product peak ‘P’ after 21 h reaction time.

Precipitation of *Sg*PsmC and optimisation of reaction conditions

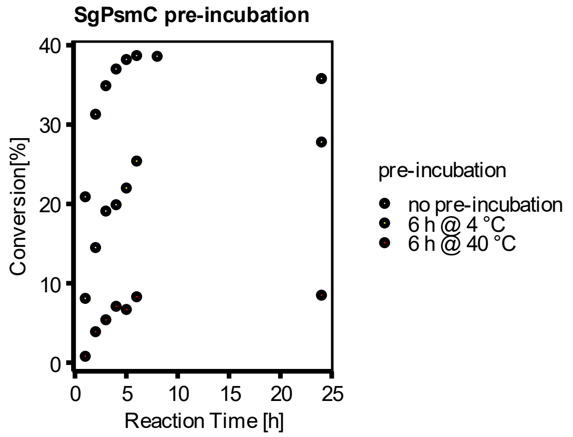

Figure S29: Conversion of ***rac*-12** using 25% v/v of cell-free extracts of *Sg*PsmC. Reactions were performed either directly (blue) or after a pre-incubation of *Sg*PsmC for 6 h at 4 °C or 40 °C (yellow and red, respectively). The results indicate a limited stability of *Sg*PsmC, as was already shown during profiling (see main text). Additionally, for the reaction performed without any pre-incubation, an apparent decrease in conversion could be observed.

Forming the ratio $\frac{\text{peak area at time t}}{\text{peak area at time t}_{-1}}$ for the two substrate enantiomers (S1 and S2) and the one *N*-methylated product (P1) formed for t = 6 h (P1: 1.05, S1: 1.04, S2: 1.09) and for t = 8 h (P1: 0.78, S1: 0.81, S2: 0.80) indicates that the reaction is coming to an apparent halt, as the ratios for all compounds are close to identical. For t = 24 h (P1: 0.35, S1: 0.63, S2: 0.53), the ratio for P1 is far smaller than for S1 and S2. Under the assumption of equal recovery rate for all compounds during extraction from the reaction samples, this may be an indication for product degradation (or faster product degradation than substrate degradation). However, this indication is not decisive, as no degradation products were observed. The apparent decrease in conversion could also be due to low compound recovery, for which recovery studies could be performed.

Conversion determined as conv = ee_S / (ee_S + ee_P). Performed with n = 1.

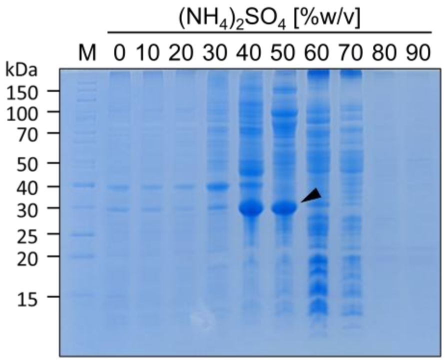

Figure S30a: Corresponding SDS-PAGE to the precipitation profile of *Sg*PsmC. The black arrow indicates a band corresponding to the molecular weight of *Sg*PsmC (predicted MW = 30.1 kDa). M: protein ladder. Coomassie stain.

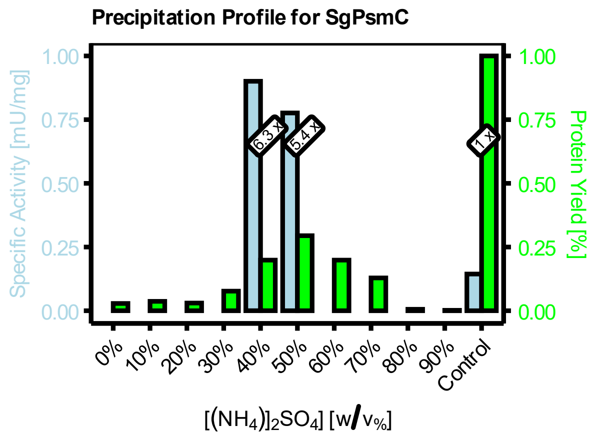

Figure S30b: Precipitation profile of *Sg*PsmC using $(NH_4)_2SO_4$ as the chaotropic agent. Precipitation proceeded in increments of 10% w/v of the chaotropic agent. After each step, precipitated protein was collected, activity determined (light blue bars) and total protein content assessed *via* Bradford assay (green bars). The purification fold (bordered label) was calculated as the ratio of volumetric activities of each fraction relative to non-precipitated CFE control. For precipitation fractions below 40% and above 50% w/v chaotropic agent, conversion against indoline could only be detected over a prolonged reaction time but was too low to reliably determine activity. For application, *Sg*PsmC was precipitated with 50% w/v $(NH_4)_2SO_4$ after removing less soluble proteins by precipitation using 30% w/v $(NH_4)_2SO_4$. Each precipitated protein fraction was measured once (n = 1).

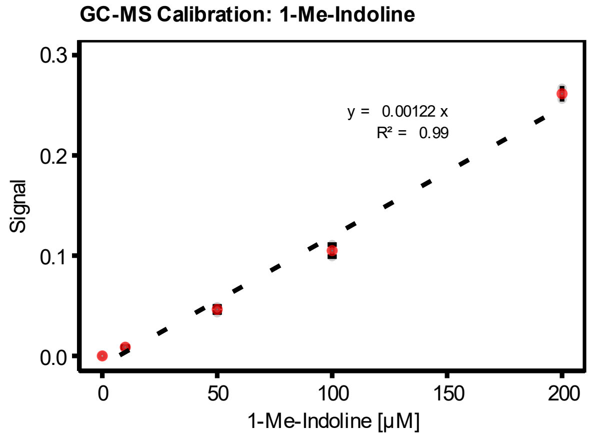

Figure S31: GC-MS calibration of the 'minimal' model product 1-Me-indoline (**8-Me**). The formation of this product was monitored to determine the specific activity of the salted-out fraction of *Sg*PsmC to construct its precipitation profile. Signal is counts of product over counts of internal standard (here, 1,3,5-trimethoxybenzene). n = 3, error denotes SD.

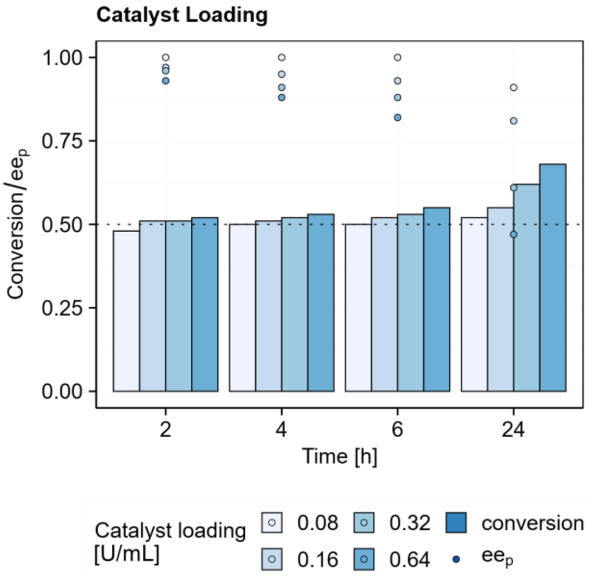

Figure S32: Conversion and *ee$_P$* of analytical scaled reactions monitored over time using ***rac*-12** as the substrate and salted-out *Sg*PsmC as the catalyst formulation. Final catalyst loading is noted in [U/mL] against the model substrate 5-Me-Indoline (**7**). Increasing catalyst loading and prolonging reaction time leads to a decrease in *ee$_P$*. Measurements performed with *n* = 1. Reaction conditions: Salted-out *Sg*PsmC at various concentrations, 1 mM ***rac*-12**, 2 mM SAM; 35 °C, 700 rpm.

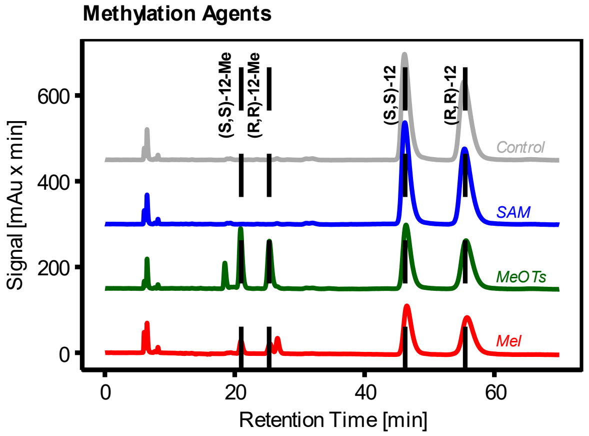

Figure S33: HPLC-UV traces of ***rac*-12** (grey, 1 mM) co-incubated for 2 h at 35 h with 2 equiv (2 mM) of SAM (blue), MeOTs (green), and MeI (red), respectively, in the absence of enzymes. SAM leads to no detectable *N*-methylated products (dashed pair of lines on the left). Both MeOTs and MeI lead to unselective background methylation, being more pronounced for the former methylation agent.

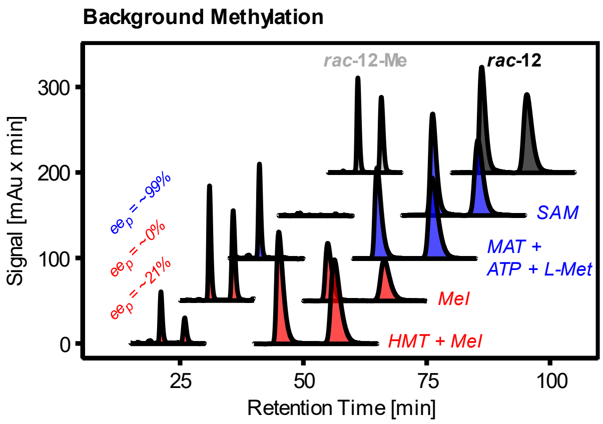

Figure S34a: HPLC-UV traces of various reaction settings and controls to highlight unselective background methylation. The HMT system leads to methylation of both substrate enantiomers (lower red trace). Enantioselectivity is preserved when the MAT system is used instead (lower blue trace). Incubation of the racemic substrate with CH_3I alone leads to non-selective methylation (upper red trace); incubation with SAM leads to no detectable background reaction (upper blue trace). The *Ct*HMT from *C. thermophilum* and the *Tk*MAT from *T. kodakarensis* were used. Racemic references of substrate and *N*-methylated product are shown in black and grey, respectively. Sub-traces were scaled for the sake of clarity. For the original unscaled traces and exact reaction conditions, see **Fig. S34b**.

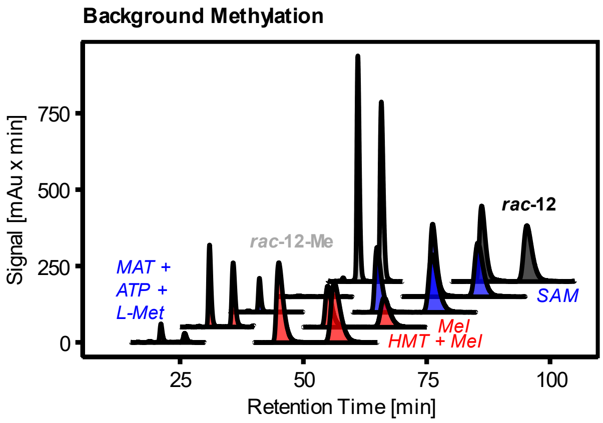

Figure S34b: Unscaled version of **Fig. S34a**. Reaction conditions from bottom to top were as follows: <u>HMT + MeI</u>: 1 µM *Sg*PsmC, 1 µM *Ct*HMT, 1 mM ***rac*-12**, 10 mM MeI, 20 µM SAH. A separate reaction with only 1 mM MeI led to a conversion of only ~9% and an E-value of ~1.6. <u>MeI</u>: Same as (HMT + MeI), but omitting *Ct*HMT. <u>MAT + ATP + L-Met</u>: 1 µM *Sg*PsmC, 1 µM *Tk*MAT, 1 mM ***rac*-12**, 2 mM ATP, 2 mM L-Met. <u>SAM</u>: 1 µM ***rac*-12**, 2 mM SAM (no enzymes).

Discussion on advantages and disadvantages of main SAM (re)generation systems

The two most common systems used to supply SAM *in situ* employ the either a methionine adenosyl transferase (MAT) or a halide methyltransferase (HMT). The MAT-systems generates SAM from ATP and L-methionine in a linear fashion, while the HMT-system regenerates SAM by remethylating SAH using a sacrificial methyl donor, such as CH_3I. Both systems have been used for the generation of SAM and analogues by supplying L-methionine analogues or alkyl halides, respectively, rendering promiscuous methyltransferases into general purpose (alkyl) transferases.[28,43–49] With both systems, a large number of cofactor analogues can be supplied thanks to the large number of MATs[28,50–52] and HMTs[48,53–57] discovered and engineered. Regarding SAM analogues, different sacrificial substrates for the HMT system (of questionable health risk) can be purchased 'off-the-shelf', whereas for the MAT system only L-ethionine is commercially available. This makes prior synthesis of L-methionine analogues necessary for which straightforward chemical and enzymatical protocols have been developed.[58,59] Only the HMT-system is a true recycling system and can run on catalytic amounts of SAH, making it better in terms of atom economy. On the other hand, the catalytic amount of SAH often used and the generally low activity of HMTs against sacrificial substrates other than CH_3I[54,57] can lead to a mismatch between a methyltransferase's K_M value and the concentration of the regenerated cosubstrate.[58] Furthermore, depending on the sacrificial substrate and the nucleophilicity of the methyltransferase's acceptor substrate, background reactions can counter the enzyme's selectivity, as has been reported for biocatalytic Friedel-Crafts alkylation[58] and shown in this work. Compared to the HMT system, the MAT linear supply does not suffer from additional background reactivity and supplies stoichiometric amounts of cofactor 'in batch', thus circumventing the problem of K_M mismatch.[58] However, the HMT system can also be optimised to minimize background alkylation by using higher concentrations of SAH and employing performant enzyme variants, as highlighted in the main text.

However, while both systems consist of only one enzyme in their minimal design, the MAT system is often extended by an additional SAH nucleosidase, making it more complex.[60] Furthermore, being a linear cascade, its atom economy cannot be on par with the recycling system. If one attempts an economic comparison of the two (**Table S26**), the MAT system supplies SAM at 13.09 ct/mmol. The HMT system – assuming catalytic addition of SAM (to circumvent the high costs of even catalytic addition of SAH) and merely two subsequent regeneration cycles – can regenerate SAM at 12.91 ct/mmol. Taking into account that HMTs reaching close to 5000 total turnovers have been discovered[61] and that no additional SAH (or SAM) needs to be supplied when CFEs are used[62] (or when purified enzymes with low K_M retain SAM or SAH at their active site during purification[63]), this cost quickly approaches the price of CH_3I at 3.26 ct/mmol.

This comparison is, of course, not exhaustive as nothing but the respective system's substrate costs (based on largest packaging size obtainable from BLDpharm, Biosynth, and Sigma-Aldrich as of June 2025)[64–68] and the natural cofactor SAM are considered, but it still shows a clear trend. As the price of SAM strongly depends on its form and SAM disulphate tosylate can by now be purchased at 32.21 ct/mmol, this poses the question whether, at least for methylation, SAM generation systems need be used at all. Taking advantage of its low price, SAM disulphate tosylate could be used instead of SAH in the HMT-system in non-catalytic amounts, circumventing a potential K_M mismatch, at least for biocatalytic methylation, or to generate larger concentrations of SAH for subsequent reactions other than methylation.

Table S26: Comparison of substrate costs for providing SAM using stoichiometric addition of SAM, linear *in situ* supply using the MAT system, and recycling using CH_3I and an HMT. Different number of regeneration cycles (RC) are considered for the HMT system. For this system, CH_3I is used as the sacrificial substrate; SAM is used instead of SAH to circumvent the high costs of the latter. Only the cost of the respective substrates is considered. Prices based on largest obtainable packaging size from BLDpharm, Biosynth, and Sigma-Aldrich as of June 2025.[64–68] Prices in Euro-cent (ct).

System	**SAM**	**MAT**	**HMT**	
Type	Stochiometric addition	Linear supply cascade	Catalytic SAM addition with subsequent regeneration cycles (RC)	
Substrate	SAM	ATP + L-Met	SAM + CH_3I	
SAM costs [ct/mmol]	32.21	13.09	3.54<br>17.73<br>12.91	(100 RC)<br>(1 RC)<br>(2 RC)

Precipitation, pH, and temperature profiles of *Tk*MAT

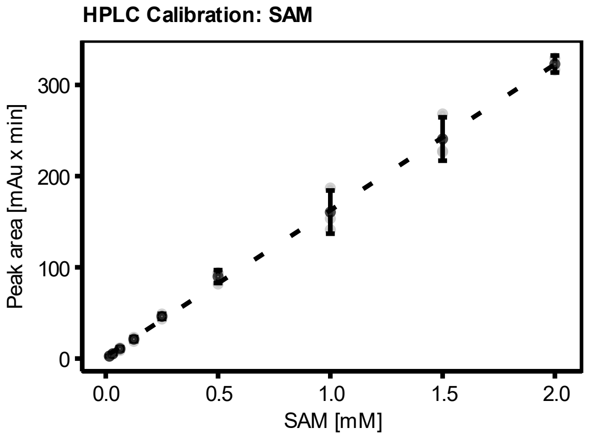

Figure S35a: HPLC calibration of SAM. Darker points indicate average values. Performed with n = 3, error denotes SD.

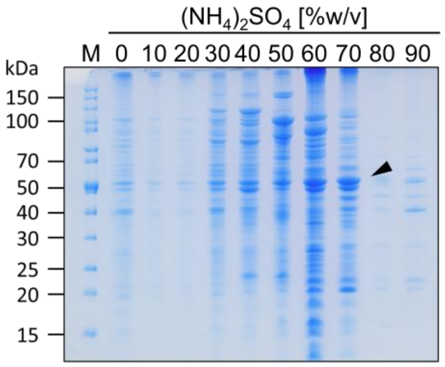

Figure S35b: Corresponding SDS-PAGE to the precipitation profile of *Tk*MAT. The black arrow indicates a band corresponding to the molecular weight of *Tk*MAT (predicted MW = 45.7 kDa). M: protein ladder. Coomassie stain.

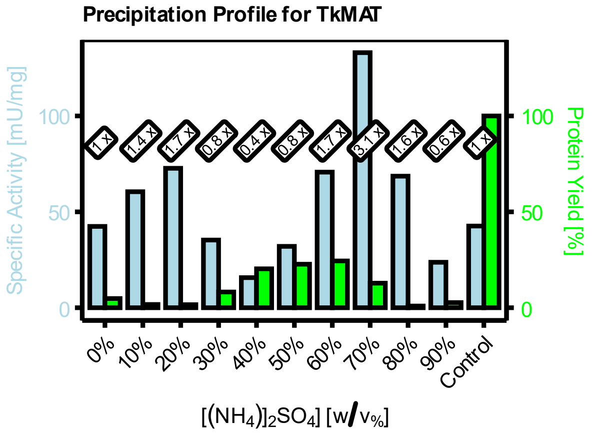

Figure S35c: Precipitation profile of *Tk*MAT using $(NH_4)_2SO_4$ as the chaotropic agent. Precipitation proceeded in increments of 10% w/v of the chaotropic agent. After each step, precipitated protein was collected, activity determined (light blue bars) and total protein content assessed *via* Bradford assay (green bars). The purification fold (bordered label) was calculated as the ratio of volumetric activities of each fraction relative to non-precipitated CFE control. For application, *Tk*MAT was precipitated with 80% w/v $(NH_4)_2SO_4$ after removing less soluble proteins by precipitation using 50% w/v $(NH_4)_2SO_4$. Each precipitated protein fraction was measured once (n = 1).

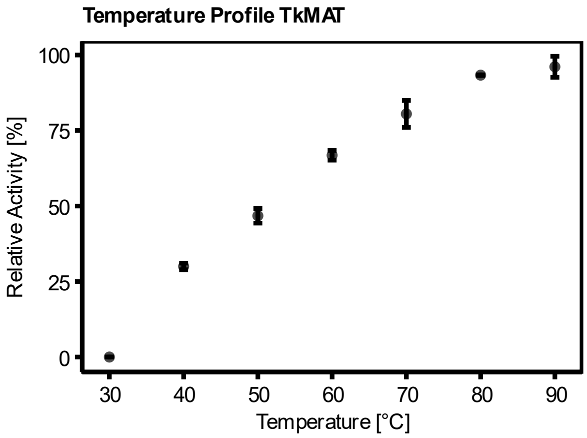

Figure S36: Temperature profile of *Tk*MAT, recorded to verify temperature compatibility with *Sg*PsmC. Measurements indicate a temperature optimum close to 80 °C. Reaction performed at pH = 8.0. Reaction sampling took place after 5 min. Product formation could also be observed at 30 °C after increasing the reaction time. Activity determined with purified enzyme with n = 3 replicates; error bars denote SD. Relative activity normalised to maximum.

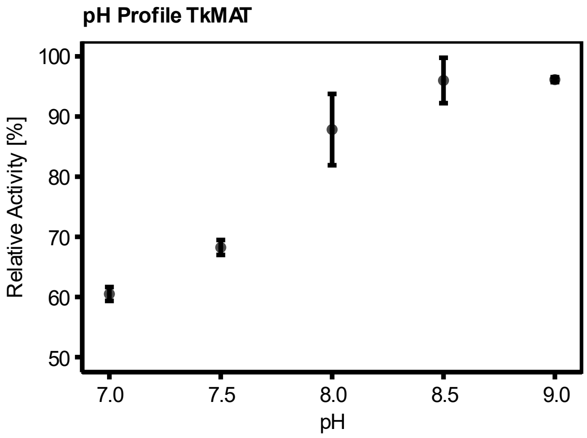

Figure S37: Narrow pH profile of *Tk*MAT, recorded to verify pH compatibility with *Sg*PsmC. Measurements indicate a pH optimum close to pH 8.5. Reaction performed at 35 °C. Reaction sampling took place after 25 min. Activity determined with purified enzyme with n = 3 replicates; error bars denote SD. Relative activity normalised to maximum.

Upscaled kinetic resolution of *rac*-12

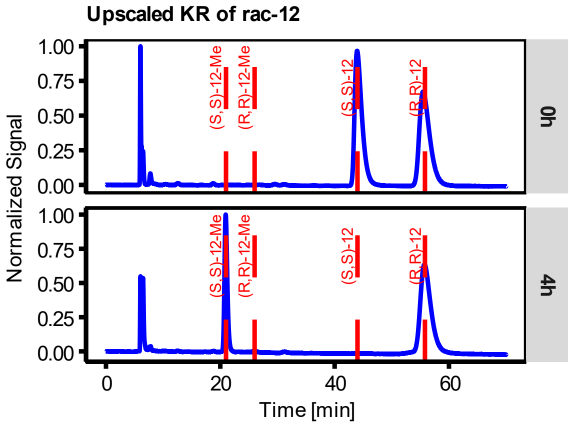

Figure S38a: Chiral HPLC-UV traces of the upscaled kinetic resolution of ***rac*-12** at the beginning of the reaction and after 4 h. The substrate **(*S,S*)-12** was fully depleted and only traces of the **(*R,R*)-12-Me** product were formed. Conversion = ~51%. Isolated yields: **(*S,S*)-12-Me**, ~13% (6.5 mg, 0.02 mmol), ee_P = ~97%; **(*R,R*)-12**: ~9% (4.5 mg, 0.013 mmol), ee_S ≥ 98%.

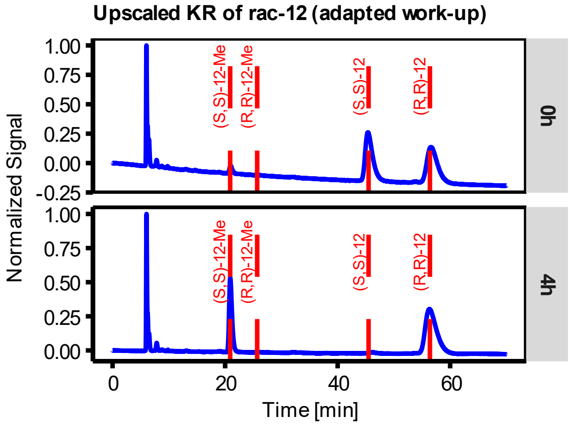

Figure S38b: Chiral HPLC-UV traces of the upscaled kinetic resolution of ***rac*-12** close to the beginning of the reaction and after 4 h. As the top sample was only taken a few minutes after the start of the reaction, slight product formation is visible. The substrate **(*S,S*)-12** was fully depleted and only traces of the **(*R,R*)-12-Me** product were formed. Here, the work-up of the high protein-density reaction mixture (forming a pronounced interphase during extraction) was adapted (see respective chapter under 'Chemical Syntheses'; first attempt). Conversion = ~50%. Isolated yields: **(*S,S*)-12-Me**, ~28% (14.1 mg, 0.043 mmol), ee_P ≥ 98%; **(*R,R*)-12**: ~21% (10.4 mg, 0.03 mmol), ee_S ≥ 98%.

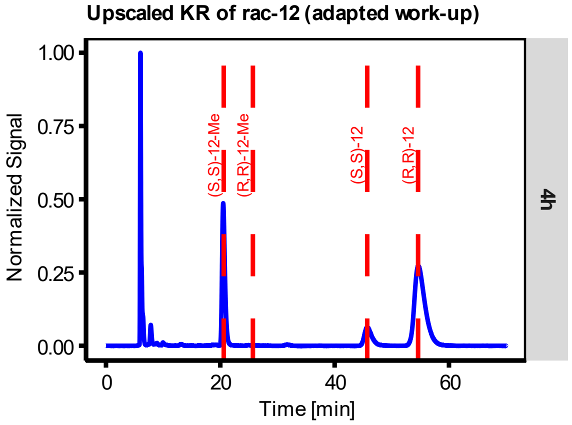

Figure S38b: Chiral HPLC-UV trace of the upscaled kinetic resolution of ***rac*-12** after 4 h. Due to heating malfunction, conversion was only ~42%. The substrate **(*S,S*)-12** showed an ee_S = ~72%. The product **(*R,R*)-12-Me** had an ee_P ≥ 98%. Here, the work-up of the high protein-density reaction mixture (forming a pronounced interphase during extraction) was adapted (see respective chapter under 'Chemical Syntheses'; second attempt). Conversion = ~42%. Isolated yields: **(*S,S*)-12-Me**, ~25% (12.7 mg, 0.038 mmol), ee_P ≥ 98%; enantioenriched **12** (regarding (***R,R***)-enantiomer): 38% (18.8 mg, 0.056 mmol), ee_S = ~73%.

Chemical syntheses

Compound 18

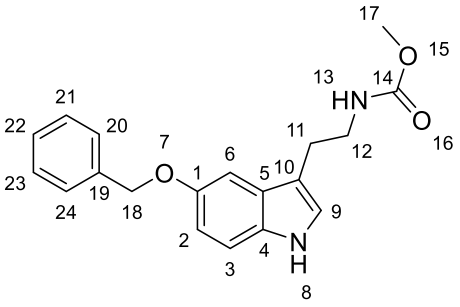

18

5-Benzyloxyindole-3-acetonitrile (500 mg, 1.91 mmol, 1 equiv.) was added to a heat-dried, N_2-purged, round-bottom 50 mL Schlenk-flask. To this, 20 mL of dry MeOH were added. The solution was let to cool down to 0 °C on ice. While stirring, anhydrous $NiCl_2$ (395 mg, 3.05 mmol, 1.6 equiv) was added, followed by portion-wise addition of $NaBH_4$ (505 mg, 13.34 mmol, 7 equiv). The mixture was left stirring for 2 h at 0 °C. Then, methyl chloroformate (360 mg, 3.81 mmol, 2 equiv) was added and the reaction stirred at room temperature for 2.5 h. Methanol was removed under reduced pressure and the remaining mixture extracted thrice with EtOAc. The combined organic phases were washed thrice with saturated $NaHCO_3$ and once with brine before drying over $MgSO_4$. After filtration, solvent was removed under reduced pressure. The remaining oil was purified *via* flash chromatography with silica (50/50 v/v EtOAc/petroleum ether), yielding the oil **18** at 55% yield (342 mg, 1.05 mmol). As based on ^{1}H-NMR, **18** was retrieved together with a by-product at a 9:1 molar ratio. This mixture was used as is for further synthesis towards ***rac*-12**.

^{1}H-NMR (600 MHz, MeOD, ppm): δ=7.47 (13, $^3J_{H,H}$=7.9 Hz, $^4J_{H,H}$=2.7 Hz, 2H; 20-H, 24-H), 7.36 (tt, $^3J_{H,H}$=7.9 Hz, $^4J_{H,H}$=1.7 Hz, 2H; 21-H, 23-H), 7.32 – 7.27 (m, 1H; 22-H), 7.22 (d, $^3J_{H,H}$=8.8 Hz, 1H; 3-H), 7.15 (d, $^4J_{H,H}$=2.4 Hz, 1H; 6-H), 7.02 (s, 1H; 9-H), 6.83 (13, $^3J_{H,H}$=8.7 Hz, $^4J_{H,H}$=2.4 Hz; 1H, 2-H), 5.09 (s, 2H; 18-H), 3.62 (s, 3H; 17-H), 3.35 (t, $^3J_{H,H}$=7.5 Hz, 2H; 12-H), 2.87 (t, $^3J_{H,H}$=7.4 Hz, 2H; 11-H).

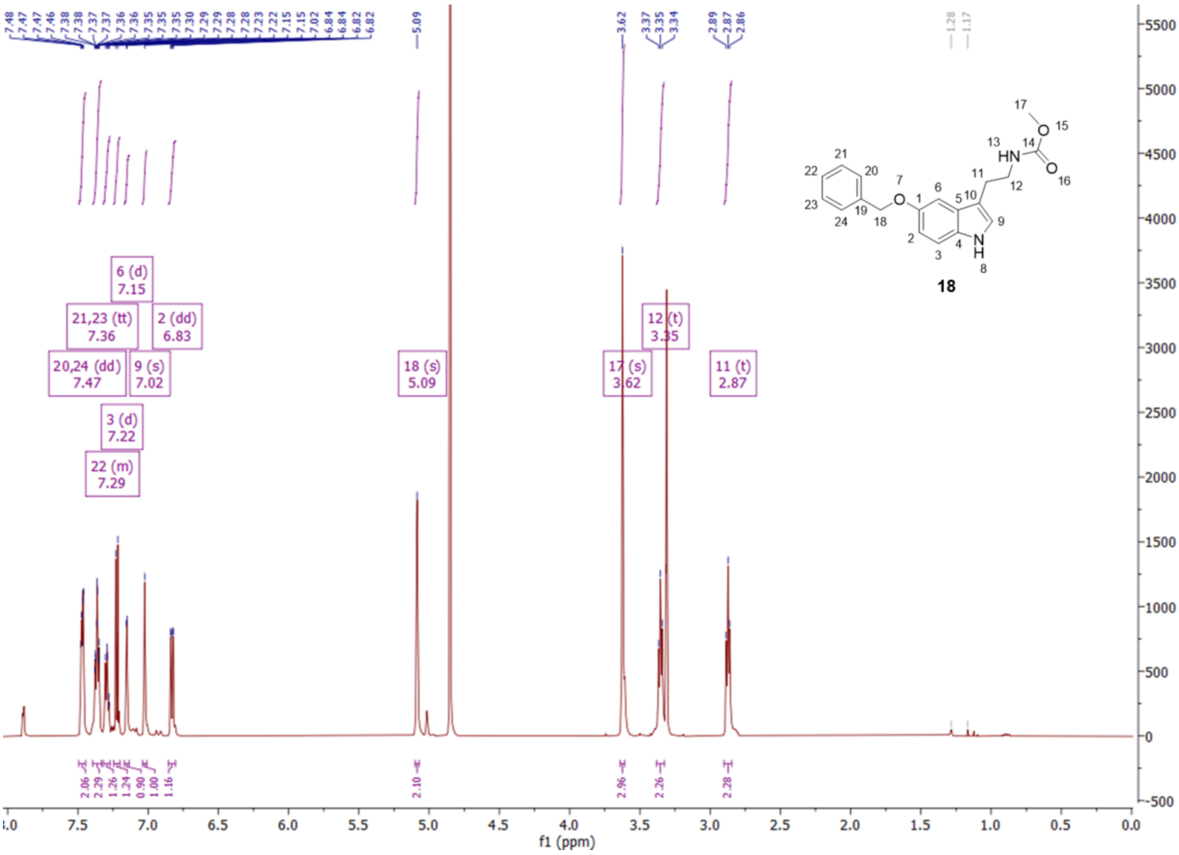

Figure S39: ^{1}H-NMR spectrum of compound **18** in CH_3OD (600 MHz).

Compound *rac*-12

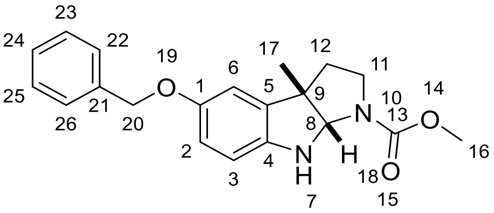

***rac*-12**

18 (651 mg, 2 mmol, 1 equiv) was added to 10 mL dry THF under an inert atmosphere in a dry Schlenk-flask, followed by the addition of *t*BuOK (673 mg, 6 mmol, 3 equiv). The mixture was left stirring at room temperature for 30 min. Then, Et_3B (1 M solution in THF, 5 equiv) was added and the solution was kept stirring at room temperature for an additional 30 min. Then, MeI (498 µL, 8 mmol, 4 equiv) was added at room temperature, yielding a milky mixture. The reaction was heated to 40 °C and left stirring for 48 h. After completion (as monitored by thin-layer chromatography), the reaction was quenched with an aqueous solution of NH_4Cl. The aqueous phase was extracted thrice with EtOAc and the combined organic phases dried over $MgSO_4$, filtrated using celite, and solvent removed under reduced pressure. The crude product was then purified by silica gel column chromatography (70/30 v/v EtOAc/petroleum ether) yielding ***rac*-12** in 78% yield (335 mg, 1.55 mmol). As a side product, the *N*-methylated compound ***rac*-12-Me** could be isolated at 10% yield (67 mg, 0.2 mmol). Two rotamers could be observed for both ***rac*-12** and ***rac*-12-Me**.

^{1}H NMR (600 MHz, $CDCl_3$, ppm): δ = 7.42 (d, $^3J_{H,H}$=7.3 Hz, 2H; 22-H, 26-H), 7.39 – 7.35 (m, 2H; 23-H, 25-H), 7.33 – 7.29 (m, 1H; 24-H), 6.75 (t, $^4J_{H,H}$=2.6 Hz, 1H; 6-H), 6.71 (dt, $^3J_{H,H}$=8.4 Hz, $^4J_{H,H}$=2.5, 1H; 2-H), 6.53 (13, $^3J_{H,H}$=8.3 Hz, $J_{H,H}$=4.4, 1H; 3-H), 5.06 and 5.01 (s, 1H; 18-H), 4.98 (s, 2H; 20-H), 3.76 (s, 1H; 16-H), 3.71 and 3.59 (ddd, $^2J_{H,H}$=10.7 Hz, $^3J_{H,H}$=8.1 Hz, $^3J_{H,H}$=2.1 Hz, 1H; 11''-H), 3.68 (s, 2H; 16-H), 3.13 – 3.03 (m, 1H; 11'-H), 2.24 – 2.16 (m, 1H; 12'-H), 2.07 – 1.97 (m, 1H; 12''-H), 1.39 (s, 3H; 17-H).

^{13}C NMR (151 MHz, $CDCl_3$, ppm): δ = 155.57 (C-13), 154.77 (C-13), 153.05 (C-1), 152.92 (C-1), 142.85 (C-4), 142.51 (C-4), 137.44 (C-21), 137.39 (C-21), 135.09 (C-5), 135.03 (C-5), 128.51 (C-23, C-25) 127.86 (C-24), 127.61 (C-22, C-26), 114.20 (C-2), 114.05 (C-2), 109.96 (C-3), 109.84 (C-3), 82.96 (C-8), 82.49 (C-8), 71.16 (C-20), 54.29 (C-9), 53.21 (C-9), 52.56 (C-16), 52.25 (C-16), 46.21 (C-11), 45.79 (C-11), 37.06 (C-12), 36.91 (C-12), 24.34 (C-17), 24.15 (C-17).

IR (ATR-film): $\tilde{\nu}$ = 2963, 1694, 1490, 1442, 1376, 1281, 1190, 1024, 866 cm^{-1}. HRMS (ESI): m/z calculated for $C_{20}H_{22}N_2O_3$ +H^+: 339.1703 $[M+H]^+$; found: 339.1711.

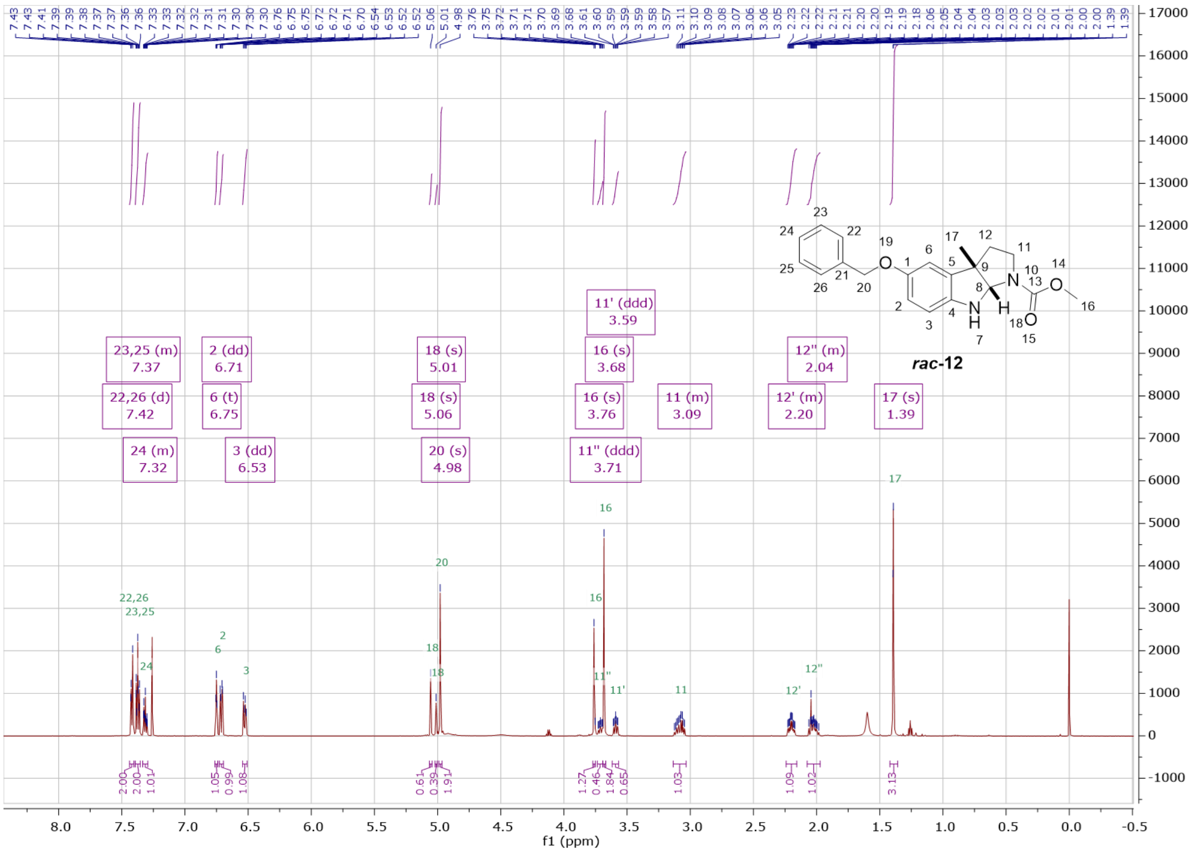

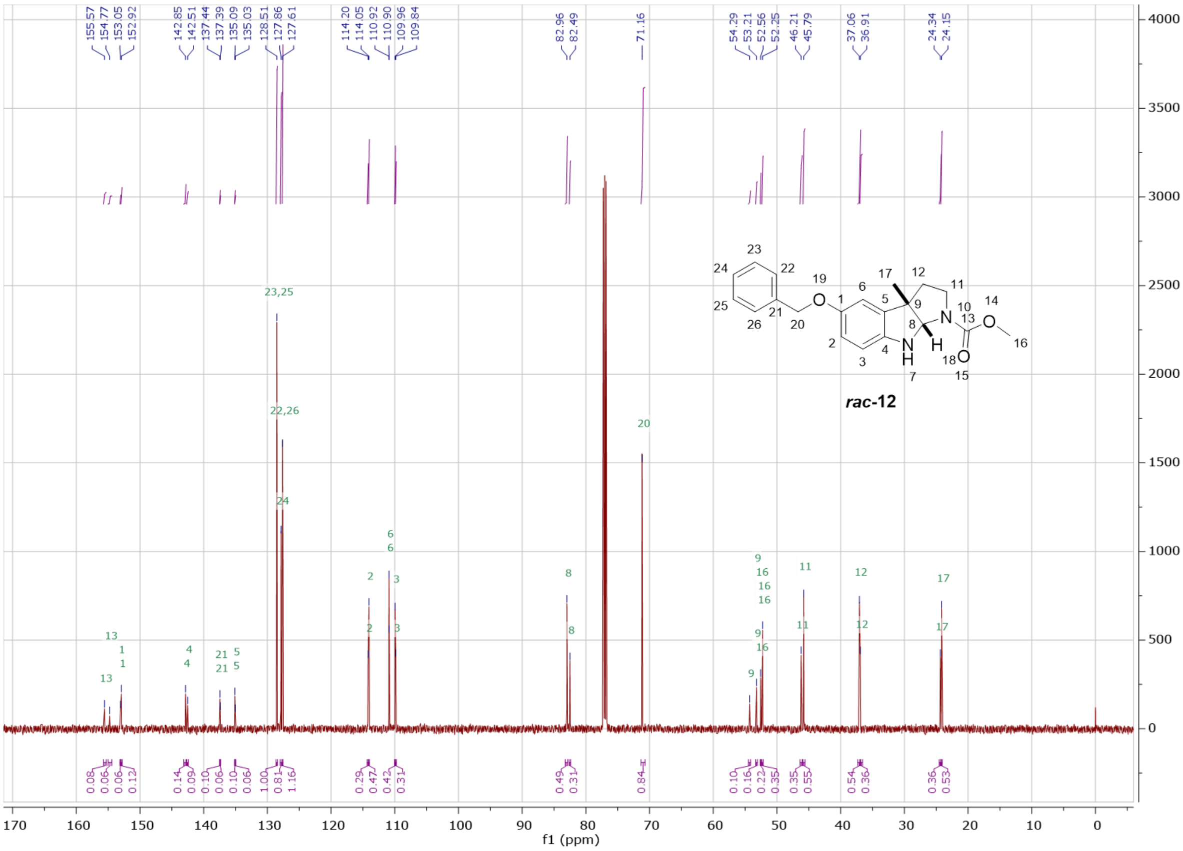

Figure S40: ^{1}H-NMR and ^{13}C-NMR spectra of compound ***rac*-12** in $CDCl_3$ (600 MHz/151 MHz).

Compound *rac*-12-Me

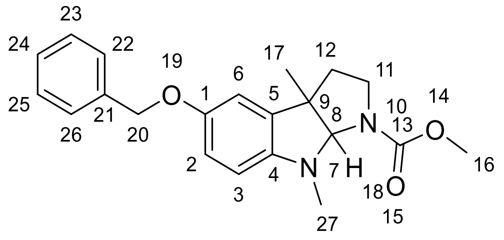

***rac*-12-Me**

***rac*-12-Me** was obtained as a side-product during the synthesis of ***rac*-12** (see above) at 10% yield (67 mg, 0.2 mmol). Two rotamers could be observed.

^{1}H NMR (600 MHz, $CDCl_3$, ppm): δ = 7.45 – 7.41 (m, 2H; 22-H, 26-H), 7.38 (dt, $^3J_{HH}$=8.4 Hz, $^3J_{HH}$=6.8 Hz, 2H; 23-H, 25-H), 7.34 – 7.29 (m, 1H; 24-H), 6.75 (13, $^3J_{HH}$=8.3 Hz, J_{HH}=2.6 Hz, 1H; 6-H), 6.73 (d, J_{HH}=2.5 Hz, 1H; 2-H), 6.32 (t, $^3J_{HH}$=7.5 Hz, 1H; 3-H), 5.15 and 5.03 (s, 1H; 18-H), 4.98 (s, 2H; 20-H), 3.84 and 3.70 (m, 1H; 11-H), 3.77 and 3.72 (s, 3H; 16-H), 3.18 (m, 5, 1H; 11-H), 2.95 (s, 2H; 27-H), 2.85 (s, 1H; 27-H), 2.09 (m, $^3J_{HH}$=5.9 Hz, 1H; 12''-H), 1.91 (m, 1H; 12'-H), 1.40 (s, 3H; 17-H).

^{13}C NMR (C-151 MHz, $CDCl_3$, ppm): δ = 156.49 (C-13), 152.12 (C-1), 145.14 (C-4), 137.56 (C-21), 135.73 (C-5), 128.47 (C-23, 25), 127.80 (C-24), 127.58 (C-22, 26), 113.78 (C-6), 111.00 (C-2), 106.74 (C-3), 89.92 (C-8), 89.17 (C-8), 71.26 (C-20), 52.42 (C-16), 51.74 (C-9), 46.45 (C-11), 46.21 (C-11), 38.83 (C-12), 38.35 (C-12), 34.26 (C-27), 33.57 (C-27), 24.44 (C-17), 24.15 (C-17).

HRMS (ESI): m/z calculated for $C_{21}H_{24}N_2O_3$ +H^+: 353.1860 $[M+H]^+$; found: 353.1866.

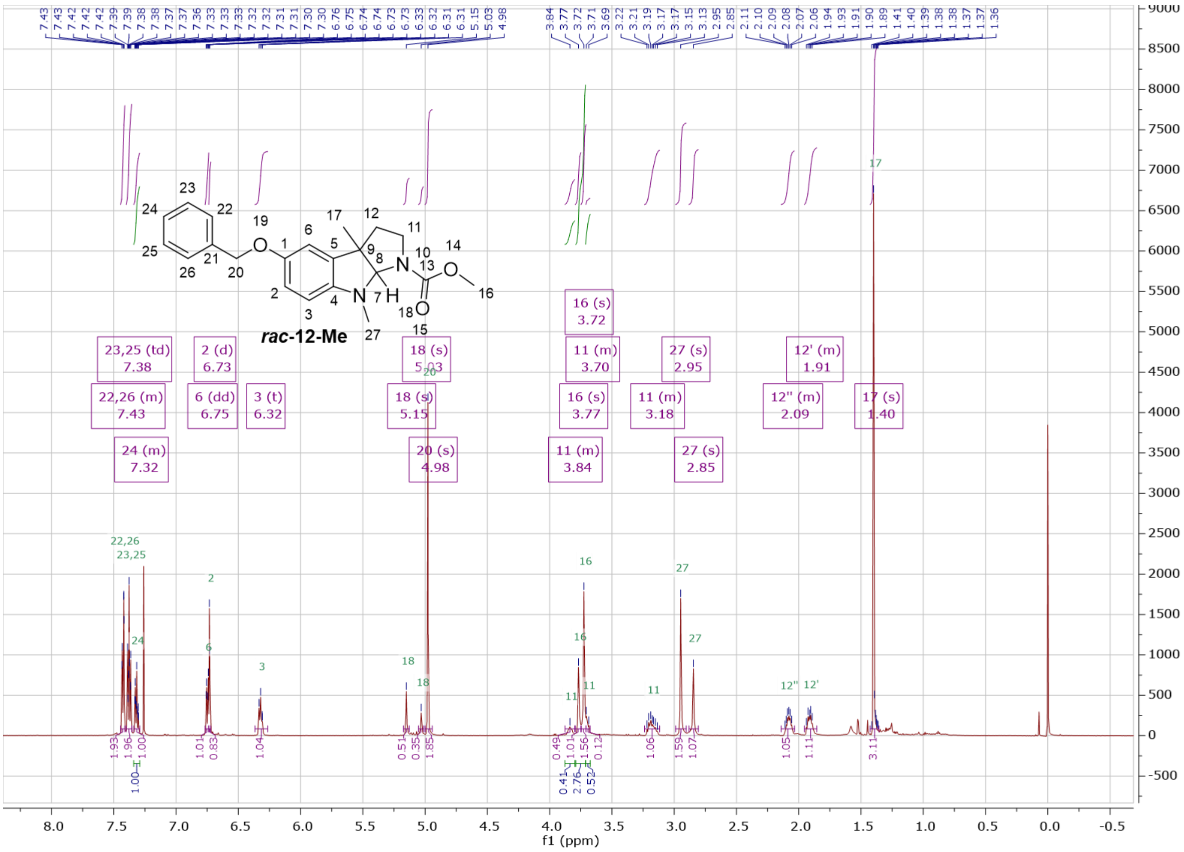

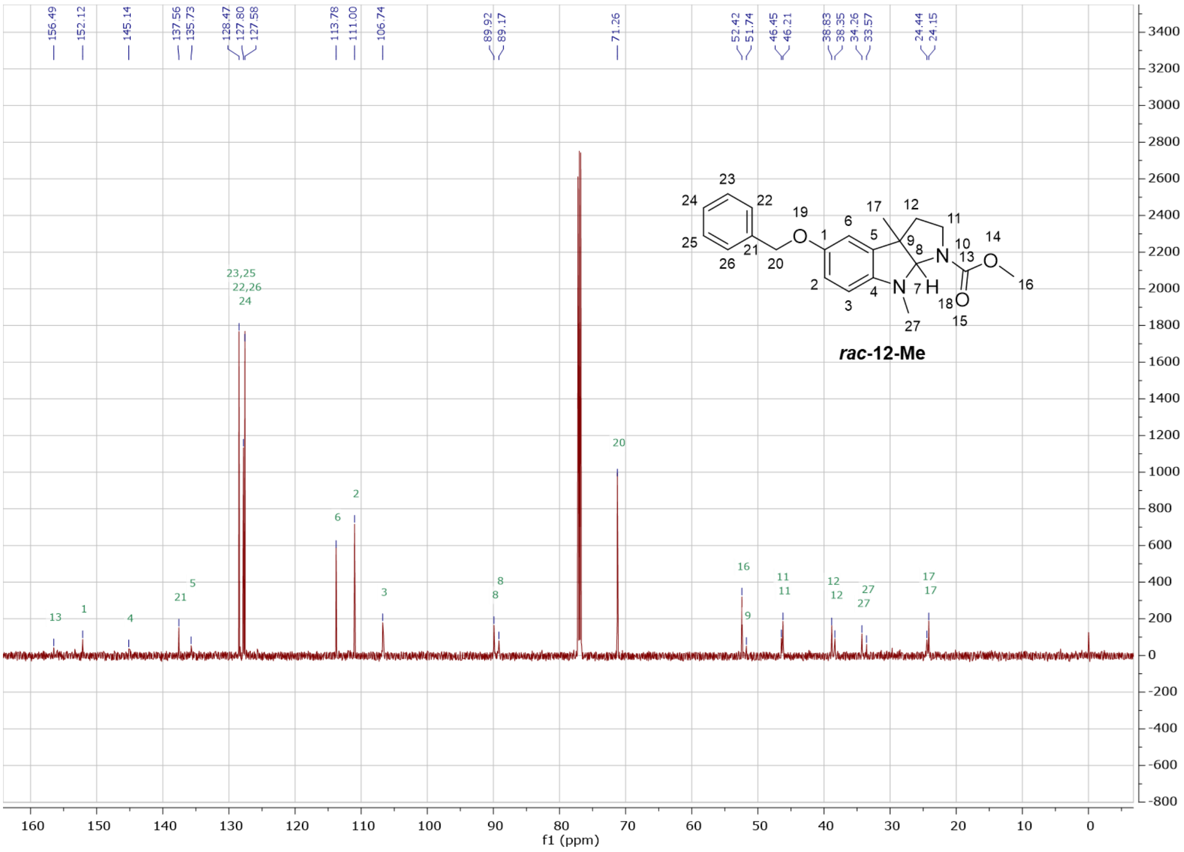

Figure S41: ^{1}H-NMR and ^{13}C-NMR spectra of compound ***rac*-12-Me** in $CDCl_3$ (600 MHz/151 MHz).

Kinetic resolution of *rac*-12 to (*S,S*)-12-Me and (*R,R*)-12

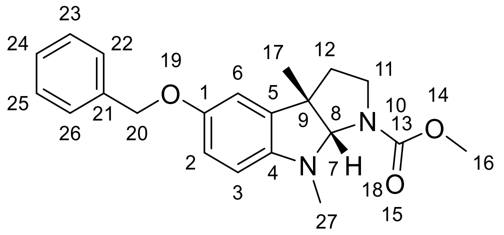

(*S,S*)-12-Me

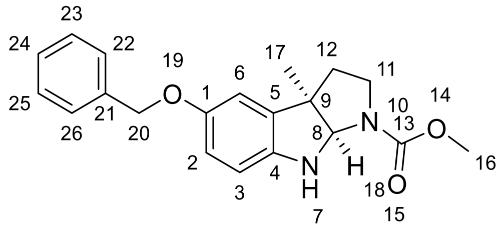

(*R,R*)-12

*Sg*PsmC and *Tk*MAT in precipitated form were prepared as described above. Substrate ***rac*-12** (100 mM stock in DMSO), ATP and L-Met (both 200 mM stocks in dH_2O) were added to reaction buffer (100 mM Tris, 20 mM $MgCl_2$, 200 mM KCl, pH 8.0) in a 250 mL Schott glass-bottle to a final concentration of 1 mM (50 mg, 0.15 mmol, 1 equiv), 2 mM (2 equiv) and 2 mM (2 equiv), respectively. Precipitated catalysts were then added to the mixture at a final activity of 0.08 U/mL (*Sg*PsmC, activity against 5-Me-indoline; 1.6% v/v) and 10.62 U/mL (*Tk*MAT, activity for SAM formation; 1% v/v). The reaction was left shaking at 250 rpm at 35 °C. After 4 h, conversion of substrate reached ~51%, and the reaction was extracted using EtOAc. The combined organic phases were dried over $MgSO_4$ and after filtration, solvent was removed under reduced pressure. Flash chromatography with silica (25/75 v/v EtOAc/petroleum ether) yielded **(*S,S*)-12-Me** at 13% yield (6.5 mg, 0.02 mmol) with ee_P = ~97%. **(*R,R*)-12** could be re-isolated at 9% yield (4.5 mg, 0.013 mmol) with an ee_S ≥ 98%. ^{1}H-NMR corresponded to the chemically produced references (see chemical synthesis above, and ^{1}H-NMR spectra below). Enantiomeric excesses were determined by chiral HPLC (see above).

Isolated yields were low, primarily due to inefficient work-up of the high protein-density reaction mixture forming a pronounced interphase during extraction. The reaction was repeated as described above but the work-up procedure was adapted. Upon reaction completion, the reaction was quenched by the addition of an equal volume of ice-cold MeOH, leading to protein precipitation. This mixture was cooled to 4 °C and then centrifuged for 10 min at 10,000 *g* and 4 °C (thus, below the flash point of MeOH, due to safety reasons) and the supernatant was collected. The pellet was washed with reaction buffer, an equal volume of MeOH added again, the suspension centrifuged, followed by collection of the supernatant. This process was repeated a total of three times. The bulk amount of MeOH was removed under reduced pressure and the educt and product were then extracted using EtOAc. Residual protein in the solution led again to an interphase, which was, however, less pronounced than before. The interphase and organic phases were collected and phase separation was left to proceed on ice. The combined organic phase was dried over $MgSO_4$ and after filtration, solvent was removed under reduced pressure. Purification proceeded as described above. Two additional attempts following this procedure were made. The first attempt led to a conversion of ~50% and ~28% isolated yield of **(*S,S*)-12-Me** (14.1 mg, 0.043 mmol) with ee_P ≥ 98%. **(*R,R*)-12** could be re-isolated at ~21% yield (10.4 mg, 0.03 mmol) with an ee_S ≥ 98%. Due to a heating malfunction of the incubator, the second reaction attempt only led to a conversion of ~42%. The isolated yield for **(*S,S*)-12-Me** was ~25% (12.7 mg, 0.038 mmol) with an ee_P ≥ 98%. Enantio-enriched **(*R,R*)-12** could be re-isolated at 38% yield (18.8 mg, 0.056 mmol) with an ee_S = ~73%.

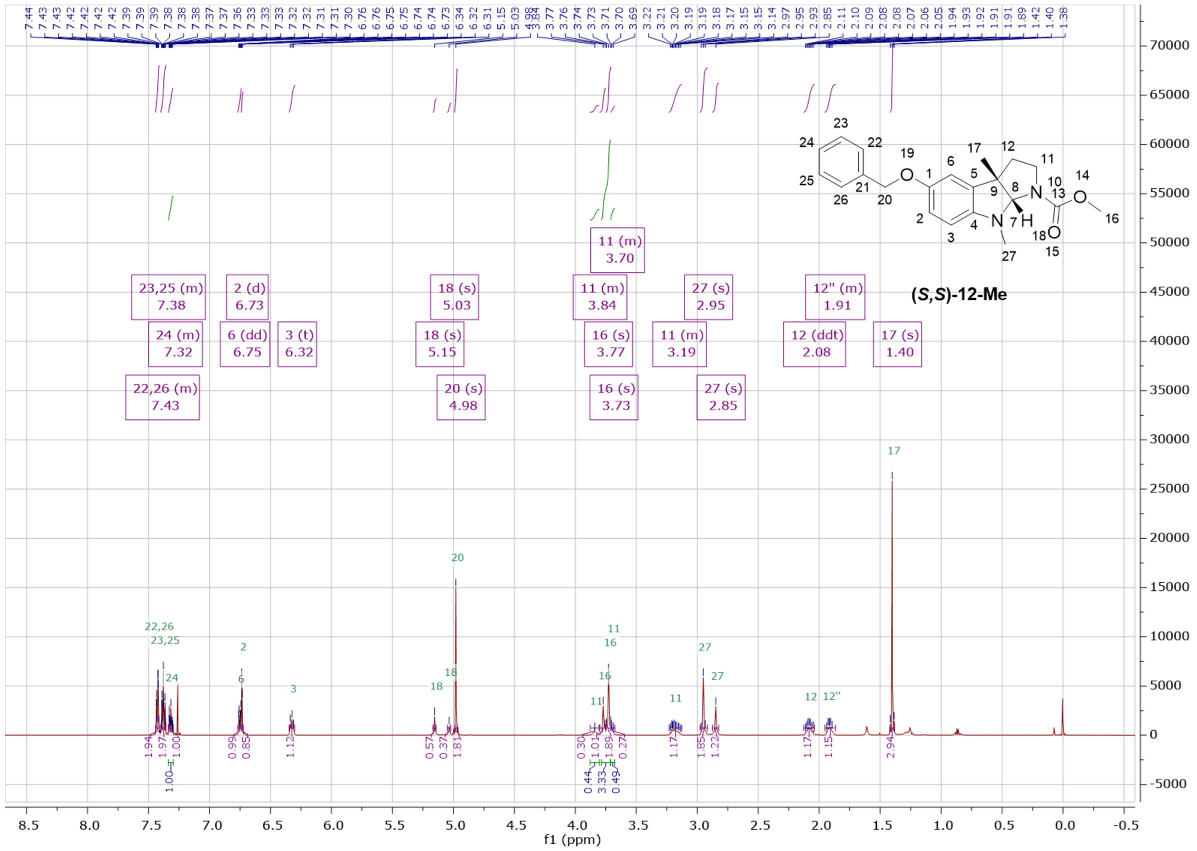

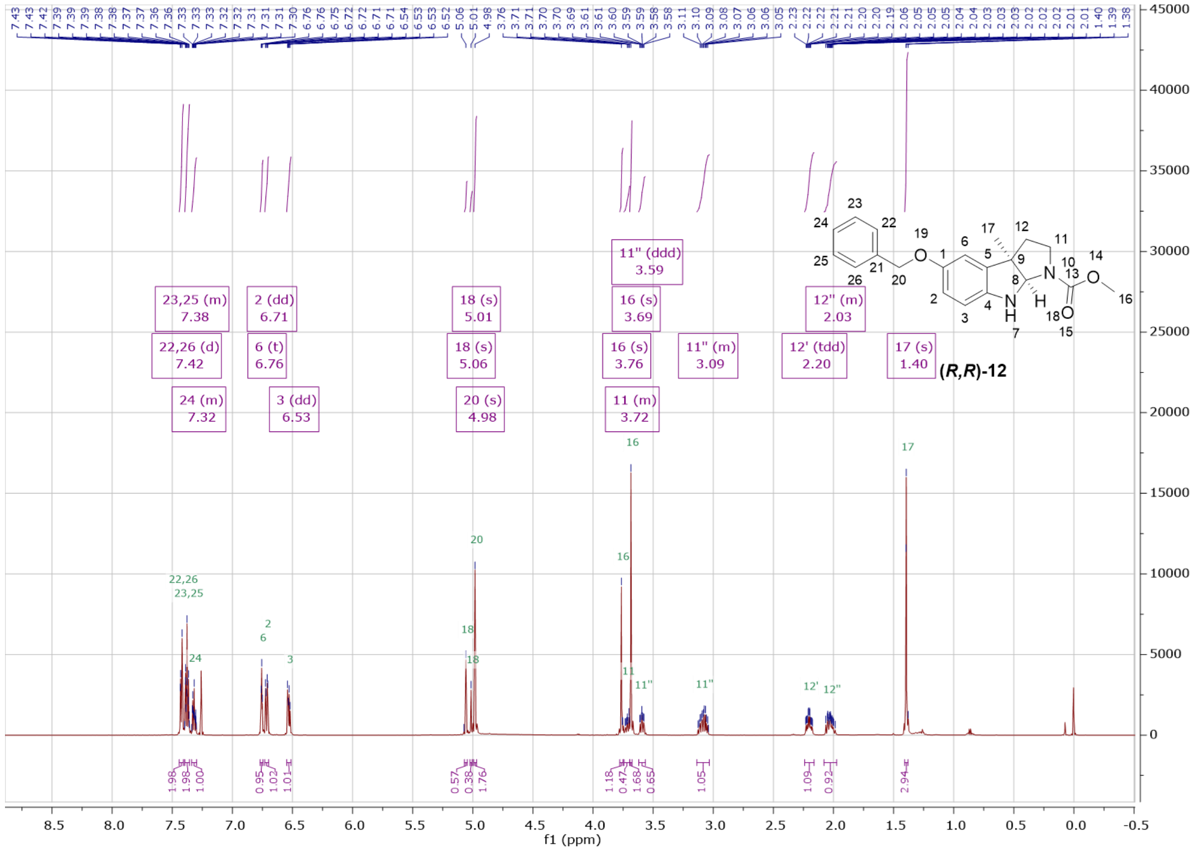

Figure S42: ^{1}H-NMR in $CDCl_3$ (600 MHz) of compound ***(S,S)*-12-Me** (top) and ***(R,R)*-12** (bottom).

Compound *rac*-16

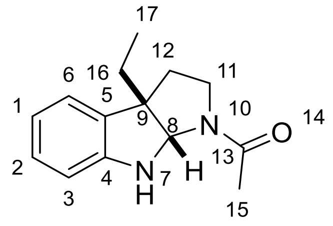

***rac*-16**

Synthesis of the pyrroloindoline ***rac-16*** was adapted from Yi *et al*.[69] *N*-methyl tryptamine (408 mg, 2 mmol, 1 equiv) was added to 10 mL dry THF under an inert atmosphere, followed by the addition of *t*BuOK (672 mg, 6 mmol, 3 equiv). The mixture was left stirring at room temperature for 30 min. Then, Et_3B (1 M solution in THF, 9.98 mL, 10 mmol, 5 equiv) was added, resulting in a clear solution which was kept stirring at room temperature for an additional 30 min. Then, EtI (642 µL, 8 mmol, 4 equiv) was added at room temperature, yielding a milky mixture. The reaction was heated to 40 °C and left stirring for 24 h. The course of the reaction was monitored by thin-layer chromatography (90:10 DCM/MeOH). After completion, the reaction was quenched using 100 mM potassium phosphate buffer (pH 8.0). The aqueous phase was extracted 3 times with EtOAc and the combined organic phases dried over $MgSO_4$, filtrated using celite, and solvent removed under reduced pressure. The crude product was then purified by silica gel column chromatography (90:10:0.1 EtOAc/MeOH/NH_3) yielding a white solid in 77% yield (354 mg, 1.54 mmol). Two rotamers could be observed.

^{1}H NMR (600 MHz, $CDCl_3$, ppm): δ = 7.08 – 7.03 (m, 2H; 2-H, 6-H), 6.75 (td, $^3J_{HH}$=7.4 Hz, 1.0, 1H; 1-H), 6.58 (dt, $^3J_{HH}$=7.7 Hz, $^4J_{HH}$=0.8 Hz, 1H; 3-H), 5.21 (s, 1H; 18-H), 3.59 (ddd, $^3J_{HH}$=9.8 Hz, $^3J_{HH}$=8.1 Hz, $^2J_{HH}$=1.3 Hz, 1H; 11'-H), 3.20 (ddd, $^3J_{HH}$=11.3 Hz, $^3J_{HH}$=10.1 Hz, $^1J_{HH}$=6.3 Hz, 1H; 11''-H), 2.27 (ddd, $^3J_{HH}$=12.5 Hz, $^3J_{HH}$=6.3 Hz, $^2J_{HH}$=1.3 Hz, 1H; 12'-H), 2.12 (ddd, $^3J_{HH}$=12.5 Hz, $^3J_{HH}$=11.2 Hz, $^2J_{HH}$=8.2 Hz, 1H; 12''-H), 2.01 (s, 3H; 15-H), 1.78 – 1.73 (m, 2H; 16'-H), 0.89 (t, $^3J_{HH}$=7.5 Hz, 3H; 17-H).

^{13}C NMR (151 MHz, $CDCl_3$, ppm): δ = 170.10 (C-13), 149.66 (C-4), 131.23 (C-5), 128.33 (C-2), 123.17 (C-6), 118.56 (C-1), 109.21 (C-3), 80.30 (C-8), 56.58 (C-9), 47.32 (C-11), 35.52 (C-12), 31.05 (C-16), 22.67 (C-15), 9.19 (C-17). LC-MS (ESI[+]): t_R = 8.59 min. MS (ESI [+]): m/z = 231 $[M+H]^+$.

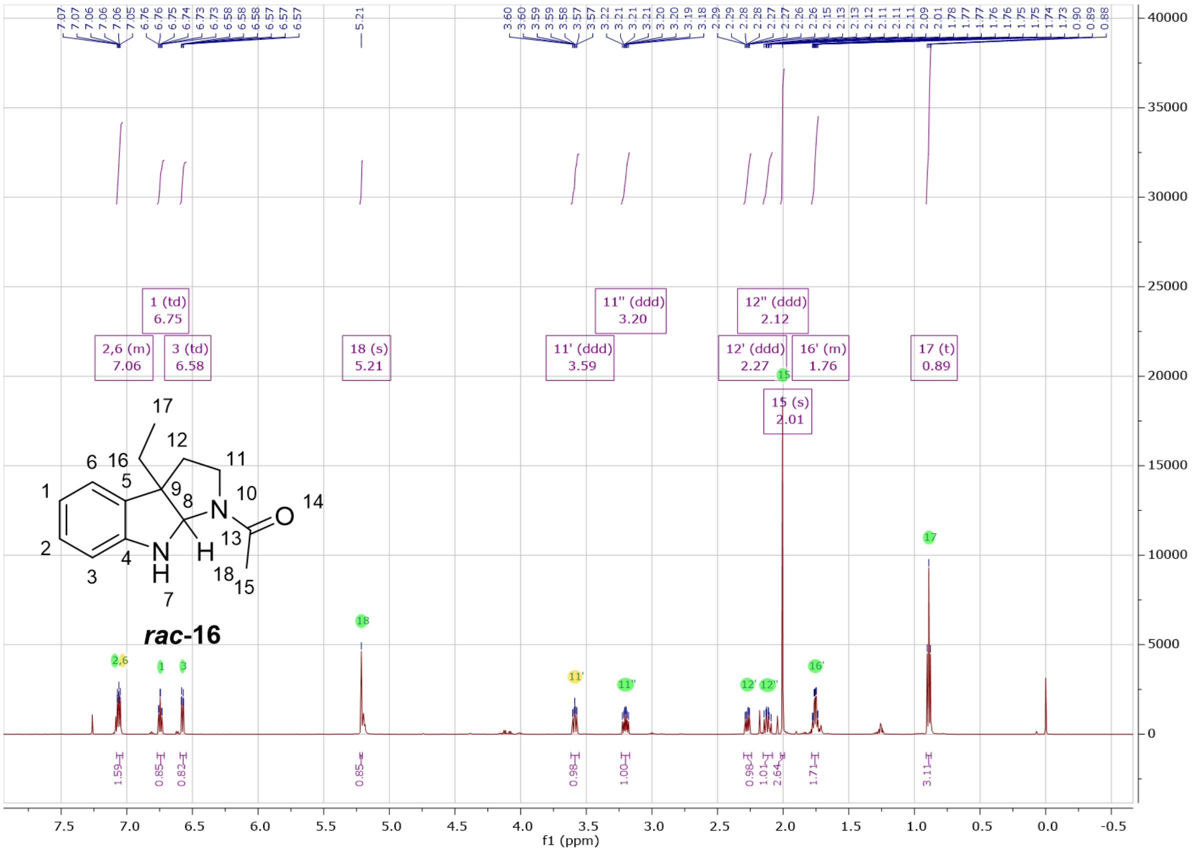

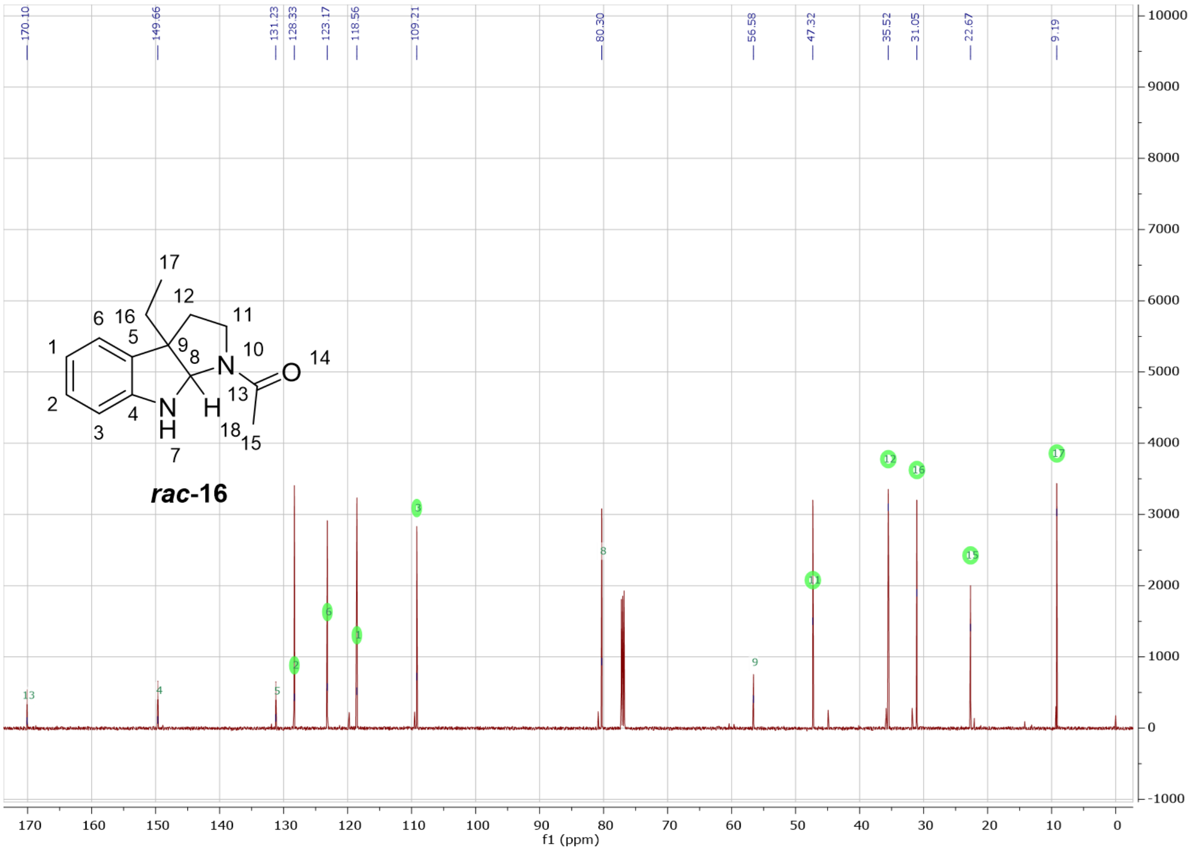

Figure S43: ^{1}H-NMR and ^{13}C-NMR spectra of compound ***rac*-16** in $CDCl_3$ (600 MHz/151 MHz).

Electronic energies, Gibbs free energies, Boltzmann weights, and coordinates of optimised configurations

Table S27: Electronic energy, correction to Gibbs free energy, and respective Boltzmann weights of the conformers of (S,S)-9 at the ωB97M-V/def2-QZVPP/C-PCM(Hexane)//r2SCAN-3c/C-PCM(Hexane) level of theory for the calculation of the ECD-spectrum.

Conformer ID #	Electronic energy [E_h]	Correction to Gibbs free energy [E_h]	Boltzmann weight [%]
1	-973.115128	0.281608	58.79
5	-973.114422	0.281406	34.48
6	-973.110934	0.281620	0.68
10	-973.110512	0.281636	0.43
12	-973.111187	0.281403	1.12
14	-973.111330	0.281189	1.64
15	-973.111177	0.280516	2.85

```
Coordinates of (S,S)-9, Conformer ID# 1
charge: 0, multiplicity: 1
40

      C     -4.02844491870555 -1.50865470397071 -2.18584067462594
      C     -3.84963412819288 -0.46294097037024 -1.10863596209006
      N     -2.88407495030945 -0.69572618477007 -0.18538878991612
      C     -2.61307203701519 0.29981612084982  0.87446623999963
      H     -3.49927862558835 0.41078470468141  1.51055164786868
      N     -2.18088479333881 1.59925107084428  0.37128072826955
      C     -0.80311662889052 1.53071937891126  0.15854548401242
      C     0.00953303428690  2.38013521979397  -0.58553215249561
      C     1.38319032770434  2.12964449256952  -0.60537805903537
      C     1.91267014723527  1.06821732037056  0.11435201266921
      O     3.30355841758534  0.90943222646316  0.11055365798975
      C     3.80273343716414  -0.25936413837602 -0.42812625817343
      N     5.15154986210348  -0.25254906396132 -0.35365982958973
      C     5.92803889263426  -1.40951523064998 -0.77206376966128
      O     3.12249798647575  -1.14524499566698 -0.90812111984972
      C     1.10480791872810  0.22123995088838  0.87214607169978
      C     -0.25596642651589 0.45759072682708  0.87451535982354
      C     -1.36548688959726 -0.28145035485945 1.59775785900060
      C     -1.34484129442927 0.01381911756333  3.09707797847637
      C     -1.40634370392065 -1.78054943523950 1.26852578001445
      C     -1.95314143384852 -1.83258871336507 -0.15570131491627
      O     -4.54486488034809 0.55809198721429  -1.06104065019758
      H     -3.08315583965263 -1.71958242031695 -2.69727373792476
      H     -4.76096178243519 -1.14284604728990 -2.90551317088337
      H     -4.38849707786671 -2.44986771383458 -1.75528501969617
      H     -2.76981046408337 1.99227169273513  -0.35192668464090
      H     -0.40665948984876 3.21162489214362  -1.14660762759031
      H     2.05074597617746  2.75974130855087  -1.18514860784819
      H     5.59778341191788  0.49977138514605  0.14549021909322
      H     5.61685635496546  -1.73024305864151 -1.77025153099516
      H     5.80624321413131  -2.25597692421094 -0.08426985806605
      H     6.98283838658839  -1.12769059852441 -0.80477860973999
      H     1.54451385021262  -0.60723628790295 1.42036809169371
      H     -0.42674976961300 -0.37918501396313 3.54620785367461
      H     -2.20253175729964 -0.45156385069136 3.59552832293486
      H     -1.37657378394440 1.09311661414796  3.27787473065264
      H     -2.09360512409520 -2.28743335693852 1.95732330083119
      H     -0.42237983586190 -2.25075117199561 1.35492278978227
      H     -2.46874984701162 -2.77371727969403 -0.37133279504752
```

```
      H     -1.14683573549782 -1.69806069446742 -0.89212190550297

Coordinates of (S,S)-9, Conformer ID# 5
charge: 0, multiplicity: 1
40

      C     -4.77657057406715 -1.31248032120331 -1.71592489799758
      C     -4.23300902470810 -0.26247459515115 -0.77318877849849
      N     -3.14893033877269 -0.60737169195744 -0.03490049109331
      C     -2.52584393981658 0.37548785951140  0.87956871939560
      H     -3.23905749866710 0.64805924211980  1.66654531635381
      N     -2.01974435401093 1.56961811970841  0.21389432002546
      C     -0.73510247998076 1.28373375903155  -0.25016662847111
      C     0.03174647712474  1.97680742062694  -1.18123388417576
      C     1.33317947245071  1.53350937973653  -1.42551780495973
      C     1.83426832259154  0.42483500093421  -0.75800793708656
      O     3.11325215851826  -0.01737813481916 -1.11446622937363
      C     4.07336283061959  -0.05951101293378 -0.12275958496179
      N     5.23910627606830  -0.50288447768303 -0.64131234500380
      C     6.40194092307811  -0.71091626755798 0.20778912166760
      O     3.89029901460137  0.26697116783668  1.03339004294236
      C     1.06638646436112  -0.28052182549850 0.16855170878905
      C     -0.21921482258255 0.16201570234572  0.41270020953591
      C     -1.25626707098454 -0.36546057894787 1.38588338793307
      C     -0.88542784689885 -0.01389199627061 2.82643598798534
      C     -1.58450382727041 -1.85103099469518 1.18268551492019
      C     -2.41305137858153 -1.87731529023591 -0.09925036785127
      O     -4.74479438591164 0.85791121185134  -0.67133956985689
      H     -5.57462361569092 -0.86582956241947 -2.30914990579100
      H     -5.18018355424189 -2.16341538767326 -1.15565227095193
      H     -3.99432988177182 -1.69518765235862 -2.38020858079983
      H     -2.67759713832109 2.02603816043784  -0.40487096652844
      H     -0.36246659881839 2.84249877825140  -1.70522945414621
      H     1.96476256768973  2.04651365923053  -2.14434591637551
      H     5.25254057992292  -0.80747225134216 -1.60089491698805
      H     6.25762647135209  -1.55075185804022 0.89892480989113
      H     6.60732666454714  0.18742520813390  0.79747411106026
      H     7.26419284124009  -0.91987082550954 -0.42917310636265
      H     1.48130893418029  -1.14591406557693 0.67730810651088
      H     -1.68080794715348 -0.31713196751664 3.51648284140289
      H     -0.72163635162459 1.06351542278373  2.92953301891909
      H     0.03947373377236  -0.52505140422268 3.11291115330354
      H     -2.18398147389081 -2.21128881513222 2.02794745524647
      H     -0.68428368461634 -2.46869296640241 1.11267293498411
      H     -3.09666932453814 -2.73141441048468 -0.13770636178969
      H     -1.76441661919806 -1.90844173890726 -0.98768876180358

Coordinates of (S,S)-9, Conformer ID# 6
charge: 0, multiplicity: 1
40

      C     -4.91154287685662 -0.63772509338061 -1.72374798971621
      C     -4.20807746126032 0.13039258755417  -0.62862181014565
      N     -3.14589333513395 -0.47616404305769 -0.04090404316952
      C     -2.38115804528284 0.22566388800563  1.01081523882256
      H     -3.03014666422920 0.41685811832012  1.87352581085449
      N     -1.75503056069801 1.46444027315248  0.56107509939855
      C     -0.52937687690780 1.13856373109317  -0.01260958622284
      C     0.28171496129655  1.91327734178317  -0.83830723619213
      C     1.50924661741503  1.38606156797950  -1.24280433135345
      C     1.90352361181303  0.12080593587832  -0.82149709956374
      O     3.11020322057391  -0.41027628095305 -1.28844573612600
```

C	4.26807807145712	-0.24233512636997	-0.55223410050685
N	4.15072020542191	0.46525181423656	0.59456471177908
C	5.33386248028202	0.76376935021358	1.39049170367528
O	5.29666541559081	-0.74046789852817	-0.96227174035759
C	1.09058680786702	-0.66087749994223	0.00242393650797
C	-0.12799247632577	-0.14162190352083	0.39599455609599
C	-1.19451990566946	-0.73848314040463	1.29449244057216
C	-0.74878324076841	-0.73359062040155	2.75664396500992
C	-1.70089213184243	-2.10616271935486	0.81381274205219
C	-2.56652807247547	-1.77771098650058	-0.40008307128755
O	-4.57259388797615	1.25666608599599	-0.27430493560106
H	-4.20823204522949	-0.96183532896548	-2.49828145128656
H	-5.67183353417385	0.00637739522821	-2.16566537493032
H	-5.39352655782077	-1.53445318311615	-1.31818718020474
H	-2.37120821898682	2.11065690832232	0.08493648572844
H	-0.03053173317389	2.89898100756108	-1.17040228567413
H	2.16528907446446	1.95160396942038	-1.89816813794186
H	3.28186375332949	0.93128250186313	0.80488827122019
H	5.87290465702269	-0.15789287610470	1.62773690659921
H	5.01408233672545	1.23860197181094	2.32078372050420
H	6.01945148962720	1.43635545399393	0.86104390696962
H	1.42272134904388	-1.65148980585053	0.30342419455175
H	0.11268776124975	-1.39610826555350	2.89063186207892
H	-1.55801861493947	-1.07976319195857	3.40897255294781
H	-0.45536888430206	0.27440127708387	3.06730954174750
H	-2.31185872719531	-2.56503807279907	1.60078807297774
H	-0.88239677377795	-2.79034581020084	0.57184716269100
H	-3.34642168072563	-2.52614167059903	-0.57165239651846
H	-1.95486950742863	-1.69572766193449	-1.31110437598596

Coordinates of (S,S)-9, Conformer ID# 10
charge: 0, multiplicity: 1
40

C	-3.73020780988001	-1.77249588141613	-2.22725894567912
C	-3.60302953300447	-0.63038561549664	-1.24507513724634
N	-2.73187838350792	-0.80025462115458	-0.21842066573429
C	-2.52454804966108	0.28354302972764	0.76597031305027
H	-3.46038862406392	0.47696155500026	1.30342855141030
N	-2.00437251047290	1.51748944948775	0.18918140374903
C	-0.61833205949268	1.40937958215411	0.12635033351201
C	0.28658377019335	2.18807296704291	-0.59102747903821
C	1.64980138298374	1.93039218971857	-0.43542630565344
C	2.08046449506374	0.91847734261437	0.41549989804936
O	3.45232195609116	0.71057626742506	0.59415304040792
C	4.12050998951324	-0.19880603050656	-0.20251158946359
N	3.37938790613634	-0.82561878465485	-1.14645577502000
C	4.02276430059428	-1.72296631231862	-2.09709612329590
O	5.30270289361414	-0.39089601703179	-0.00436663467056
C	1.17630720078945	0.13747486862365	1.13858529967280
C	-0.17301065833126	0.38902805626299	0.97964104038779
C	-1.36856712913448	-0.25679287375262	1.65412076297300
C	-1.47599966753903	0.17916918544274	3.11526889903320
C	-1.43057195103847	-1.77891771416255	1.46337098971031
C	-1.85481576502863	-1.95665493220833	0.00753125609090
O	-4.25597528102331	0.41216576373468	-1.35928571046342
H	-2.75363285778385	-2.06830568398230	-2.62527282681662
H	-4.37823186046266	-1.45603027133786	-3.04466763729876
H	-4.16943602958732	-2.65158700878513	-1.74223919912648
H	-2.51498164871893	1.87254219773201	-0.60884828760695
H	-0.05196057125724	2.98132364246757	-1.25100557787766
H	2.38855553889286	2.51766245712072	-0.97326282073817

```
      H     2.43793166343971  -0.51144113964728 -1.32407456288431
      H     4.70864913917944  -1.18592292274355 -2.76358035886534
      H     3.24748793703234  -2.20669653963629 -2.69576154903858
      H     4.59261676206618  -2.49004256980175 -1.56523423720190
      H     1.54639741878812  -0.64164178207023 1.80058577864618
      H     -0.61775616392728 -0.19289095110123 3.68428979462092
      H     -2.39197773491211 -0.21450182663176 3.56947645033413
      H     -1.48689229086294 1.27111514031010  3.19206269267348
      H     -2.19165749775948 -2.19865648106293 2.13245754052460
      H     -0.47523200668980 -2.26420557714301 1.68396111675142
      H     -2.38677921068395 -2.89844496464237 -0.16040218523487
      H     -0.98385705955438 -1.92453719357674 -0.66480155264314

Coordinates of (S,S)-9, Conformer ID# 12
charge: 0, multiplicity: 1
40

      C     -5.85867372050539 -0.81842027116604 -0.31637458341498
      C     -4.67491555497175 0.01667940464588  0.11528760050383
      N     -3.45223552273547 -0.56733681172273 0.01870580386736
      C     -2.26306988876238 0.15036744800041  0.45716304686045
      H     -2.43030935081667 0.54670110003774  1.46942960286664
      N     -1.83877506018812 1.21953463972109  -0.45956156196880
      C     -0.44947657976675 1.15799582193079  -0.57978582808919
      C     0.41308542561298  2.10407525009623  -1.12498241945413
      C     1.77608076856133  1.80464601083449  -1.17794590041543
      C     2.24420878430697  0.58478860021273  -0.71258529123256
      O     3.60912630065845  0.31196103903632  -0.86242363235078
      C     4.34486924344053  0.11767432226508  0.28923098732534
      N     5.61881882937602  -0.20039728460280 -0.03665964255931
      C     6.64249092904489  -0.30614636622238 0.99234848839982
      O     3.90030813158396  0.20629493077720  1.41643353596266
      C     1.38475951264996  -0.37322691651064 -0.17511907003387
      C     0.03947273887926  -0.06819750598140 -0.10370908886217
      C     -1.11793429839363 -0.89552480750201 0.39232349596949
      C     -0.88010403955523 -1.59423049184069 1.72698301271664
      C     -1.63990404852191 -1.87823874548806 -0.69072522670518
      C     -3.15361972494242 -1.91845048314469 -0.47690664582254
      O     -4.79669400557404 1.17415956849392  0.52390452126186
      H     -5.84600227930771 -0.95743479598647 -1.40362238907972
      H     -6.77325499414447 -0.29416550927332 -0.03877648286349
      H     -5.84620340228302 -1.81159545553410 0.14439176045653
      H     -2.25912425694413 2.12367785491587  -0.29333184254705
      H     0.04499221962028  3.05741998372738  -1.49252298241079
      H     2.48296935273778  2.52294040282677  -1.58179847490504
      H     5.89251048001047  -0.11340306737467 -1.00245779236177
      H     7.53385150301730  -0.75978445326786 0.55282680790791
      H     6.28624763328989  -0.94366519338734 1.80577113308492
      H     6.90910023413151  0.67234271070750  1.41248363154240
      H     1.77923422265785  -1.31632346278186 0.19175129037328
      H     -0.58431118355281 -0.87260550793796 2.49442744143375
      H     -0.08400041283156 -2.33983088171247 1.63221163311901
      H     -1.78627493907210 -2.10858275014825 2.06742829492133
      H     -1.17596906670898 -2.86520603376316 -0.60554727391365
      H     -1.41621342524705 -1.47483545260914 -1.68409240222689
      H     -3.43835526133042 -2.67880153408410 0.26556410321134
      H     -3.69466529342340 -2.13114530618726 -1.40485766056721

Coordinates of (S,S)-9, Conformer ID# 14
charge: 0, multiplicity: 1
40
```

C	-5.66714956768750	-1.32125529740064	-0.63558664171092
C	-4.68371464721156	-0.22871286779445	-0.28341391302826
N	-3.42202455314370	-0.62471017683311	0.02713947629511
C	-2.42264364835711	0.36065844871226	0.41612151288898
H	-2.83610645437728	1.00550339250922	1.20552669734003
N	-1.91138717425997	1.16574968559361	-0.70333362272926
C	-0.52784851501665	1.27835474378561	-0.55911290585716
C	0.34329471536842	2.13561512067785	-1.22416646664987
C	1.71007241480635	2.02736524404396	-0.95866167990247
C	2.17568568897382	1.08841232619486	-0.05011163349589
O	3.54628782959709	1.08768104310323	0.23476632866781
C	4.25292098089571	-0.06280014315247	-0.05369019704250
N	5.55241169330834	0.11223521331570	0.27359169386956
C	6.50486611545969	-0.98147920896885	0.16123591461968
O	3.77188684909525	-1.06571643022436	-0.54308092428719
C	1.30823467534323	0.23110126230914	0.62691606633897
C	-0.04333362241310	0.33719945444542	0.36230964454490
C	-1.20063024512772	-0.47329027125324	0.88495662442793
C	-1.19180691705991	-0.71377221121336	2.39081519260458
C	-1.38735544077004	-1.79400393587275	0.08961489738967
C	-2.90181136354408	-1.99971234274123	0.03421795824653
O	-4.99329713627358	0.96543283514715	-0.28608033606464
H	-5.66201360417025	-2.12794693494840	0.10470956106744
H	-5.40993093923716	-1.76090271776616	-1.60624921881845
H	-6.66398301630666	-0.88467013662230	-0.69950559511486
H	-2.43659548775171	2.00403296456286	-0.91075735867476
H	-0.02227966723572	2.87224203817179	-1.93354503996523
H	2.42159095829638	2.68049388332148	-1.45454704009049
H	5.81141436982481	0.95605415038502	0.75862659356603
H	7.51502184898951	-0.57621485906698	0.25440790401423
H	6.40598834088942	-1.46328105816889	-0.81529498742719
H	6.35263130772790	-1.74190280797695	0.93807012736254
H	1.69797524389326	-0.49248559562742	1.33670151946280
H	-2.09785503908583	-1.24254738839048	2.70862389033945
H	-1.13894142926377	0.23443170753203	2.93465696009990
H	-0.32834258938488	-1.32375489556879	2.67562690907930
H	-0.86413987891092	-2.63173525169340	0.55947309783935
H	-0.99213359986247	-1.66530904551795	-0.92350744287843
H	-3.26932158687990	-2.55144807724990	0.91225842895858
H	-3.20936690913770	-2.54649185975912	-0.86325199528582

Coordinates of (S,S)-9, Conformer ID# 15
charge: 0, multiplicity: 1
40

C	-5.90730505895467	-0.81066291853831	-0.08444318963087
C	-4.71462282254306	0.11564459707607	-0.01763743930338
N	-3.50044767558183	-0.46913427946832	0.15351742191385
C	-2.30436596409542	0.35312229381523	0.27420015923247
H	-2.47381883207707	1.13243250764239	1.03167186206895
N	-1.85688139950404	0.94649776686993	-0.99513868624865
C	-0.46778782422207	0.82663516386007	-1.06105361285695
C	0.41305491866500	1.45604481549288	-1.93528046678960
C	1.77169474648577	1.14660916766332	-1.84299970532440
C	2.21803781436250	0.22055278052142	-0.91182057628868
O	3.57945338381599	-0.10538621512936	-0.92069703195782
C	4.30804882079612	0.18905781895428	0.21466642345621
N	5.59250785832263	-0.18994353233361	0.03952189218233
C	6.56467274773809	-0.04935479751295	1.11254031127895
O	3.85482030977144	0.71986909247042	1.20947384188784
C	1.34009943875026	-0.42246785296279	-0.03930344752078
C	-0.00093783384389	-0.10052609828353	-0.11683821134739

C	-1.17435498375899	-0.63865146420714	0.66011772053122
C	-0.95734291426172	-0.72911903183845	2.16709772328959
C	-1.70215867368978	-1.97444525578946	0.06987030307586
C	-3.21816437592303	-1.90760079528115	0.25994841655572
O	-4.82171554095274	1.33983644617638	-0.12380726751753
H	-6.81609181220728	-0.20904778365497	-0.06137474511021
H	-5.91440740605691	-1.52534059184250	0.74505477619158
H	-5.88645784417627	-1.38596379152215	-1.01726183387412
H	-2.26521398403405	1.84305754147880	-1.22138675686117
H	0.06216628367966	2.17802678680302	-2.66679408519240
H	2.49240174971268	1.62761798162740	-2.49720087400230
H	5.83826227200558	-0.67877875616586	-0.80564465524309
H	6.34718189695871	-0.72381084679408	1.95001140548326
H	6.56895400235553	0.97726520128421	1.49112775685684
H	7.55517308539256	-0.28362752395715	0.71650468130855
H	1.71731173640739	-1.13567042645186	0.68770843829321
H	-0.65777468343113	0.24150501007257	2.57376679091939
H	-0.17089578910102	-1.45509422680442	2.39768326139949
H	-1.87337866091865	-1.04933385078107	2.67696026609343
H	-1.25395073069585	-2.84361514953698	0.56007394019441
H	-1.46389736243768	-2.01777538269675	-0.99825795197825
H	-3.52117884403825	-2.29343764465896	1.24474389242434
H	-3.75433004871453	-2.47637675559657	-0.50694074758990

Table S28: Electronic energy, correction to Gibbs free energy, and respective Boltzmann weights of the conformers of ***(S,S)*-10** at the ωB97M-V/def2-QZVPP/C-PCM(Hexane)//r2SCAN-3c/C-PCM(Hexane) level of theory for the calculation of the ECD-spectrum.

Conformer ID #	Electronic energy [E_h]	Correction to Gibbs free energy [E_h]	Boltzmann weight [%]
1	-689.818377	0.230551	93.23
2	-689.815111	0.230282	3.9
3	-689.814112	0.229849	2.14
4	-689.812877	0.230085	0.45
5	-689.811706	0.229476	0.25
6	-689.809464	0.229562	0.02

```
Coordinates of (S,S)-10, Conformer ID# 1
charge: 0, multiplicity: 1
32

      C     -3.53370898155387 -0.82748066045900 1.37380424454127
      C     -2.68940115140728 -0.67483406966986 0.12875549677626
      N     -1.67540198970192 0.22411368936709  0.18461557565428
      C     -0.77211286729110 0.40064468610081  -0.97374256161556
      H     -1.34451874780303 0.77227417581215  -1.83197041116784
      N     -0.03129159436413 -0.80158622452297 -1.33649572126613
      C     1.11348506105309  -0.85245802909445 -0.54308343023719
      C     1.96451767394990  -1.92981298574983 -0.31014528563660
      C     3.09648578514592  -1.71220319447958 0.47752413498469
      C     3.37650397759960  -0.45733746288022 1.01696996297606
      C     2.51111765338731  0.61606243732599  0.77460372203427
      C     1.38265259448069  0.41002290549456  0.00238194535717
      C     0.30655881067812  1.38468558181251  -0.43929607608800
      C     0.83161232199876  2.34102989664263  -1.50996998770997
      C     -0.38634226690132 2.09907859639880  0.72998232796271
      C     -1.27468773832840 1.02349184880105  1.35016120136140
      O     -2.90461527337256 -1.33107187094331 -0.89627501566652
      H     -4.23716017595352 -1.64537059840563 1.21725170202365
      H     -4.09349779210991 0.09246483760614  1.57688042000808
      H     -2.91407996265610 -1.03819071557436 2.25203598573644
      H     -0.59265314694829 -1.63533098920523 -1.45231467716049
      H     1.75486978111194  -2.91104591500908 -0.72678959182304
      H     3.76995030177375  -2.54205184662855 0.67435381088178
      H     4.26272316594109  -0.31393761034367 1.62733191951372
      H     2.72343730163091  1.59683158659355  1.19533274440196
      H     0.02632472610427  2.98038301854617  -1.88904481703104
      H     1.26078331919749  1.78239760127740  -2.34798029830000
      H     1.61585522214329  2.98077812188679  -1.09190347678096
      H     -1.00173581700427 2.92146631645496  0.34438447380070
      H     0.33075277072884  2.51265333200795  1.44525997219300
      H     -2.14589346713504 1.44511725553789  1.86161400240871
      H     -0.70978949439421 0.41117628529929  2.06906770786718

Coordinates of (S,S)-10, Conformer ID# 2
charge: 0, multiplicity: 1
32

      C     -4.28084476491672 -0.03802927260684 0.58073664915714
      C     -3.03434221404002 -0.41510825746395 -0.18654334315576
      N     -1.89907051584296 0.25995060917045  0.13076721344654
      C     -0.66403641844120 -0.00483430367334 -0.59439861866836
      H     -0.86231994931104 0.03633374159844  -1.67557526491732
      N     -0.01699031799730 -1.27352094072794 -0.22964464548033
```

```
      C     1.35353700698306  -1.04548194228493 -0.11608201693650
      C     2.37980845110555  -1.98383005595110 -0.03764082626379
      C     3.68600403873834  -1.51720597994537 0.12196589231111
      C     3.96555082105960  -0.15436597153039 0.20743709233674
      C     2.92425976903999  0.77844987040480  0.12961763223909
      C     1.62746781038948  0.32861927218473  -0.03731762053109
      C     0.32704717272706  1.08751653702981  -0.11130486833319
      C     0.34496409040372  2.31026958570948  -1.02285334039532
      C     -0.23078634212114 1.42731662014632  1.29764611200681
      C     -1.74845331851289 1.30528954533890  1.15296802165475
      O     -3.03075241197591 -1.29576690222536 -1.05042573301043
      H     -5.13438058739666 -0.54194277453298 0.12703930647026
      H     -4.44608263024367 1.04444400975712  0.57963900569805
      H     -4.19353348549427 -0.35807548344111 1.62542932848593
      H     -0.32436090255431 -2.08591275874023 -0.74642944973784
      H     2.17267515072012  -3.04834082643895 -0.10375267911573
      H     4.49957879811722  -2.23562713688474 0.17591558040621
      H     4.99014696380233  0.18363969987605  0.32729174189203
      H     3.13726624827666  1.84340201417028  0.18935714537919
      H     -0.64994355992848 2.76584864278765  -1.08894796947103
      H     0.66462620169267  2.03584806899416  -2.03282856081780
      H     1.03647365568707  3.06598635556604  -0.63615374444372
      H     0.08164246593946  2.42088176822348  1.63231576924669
      H     0.13398330899992  0.68880880857950  2.01939441165404
      H     -2.20185464476794 2.24946834000085  0.81616646652288
      H     -2.22903989013773 1.01470911690917  2.09308131237075

Coordinates of (S,S)-10, Conformer ID# 3
charge: 0, multiplicity: 1
32

      C     -3.38437491435919 -1.03912442837344 -1.11137258593432
      C     -2.91193488956184 -0.51047295938375 0.22981338162310
      N     -1.66895304788155 0.04790530202243  0.26993335025417
      C     -0.75878024110172 0.20021517777201  -0.87352889126375
      H     -1.31777525214614 0.51964981529164  -1.76043630639692
      N     0.04594642920675  -0.98868357294101 -1.18937423495681
      C     1.24908421376625  -0.91266899924676 -0.48384578056309
      C     2.18991872735756  -1.91701981002552 -0.27264221399887
      C     3.36105042562139  -1.58346363543180 0.40957735240670
      C     3.58983984150120  -0.28571539932721 0.86521889554574
      C     2.63210595409521  0.71063988190919  0.64713316130601
      C     1.46498529448972  0.38883548756415  -0.02276488306781
      C     0.28437853439675  1.26358968254453  -0.39117511650463
      C     0.65309546942159  2.27221236804095  -1.47926519078910
      C     -0.37788989577539 1.90553668737652  0.84022198397297
      C     -1.18505464675668 0.76835789985664  1.45725632318897
      O     -3.63024317049064 -0.56423062696949 1.22761276544123
      H     -2.67276153257887 -1.72886083248656 -1.57494460714381
      H     -3.54565712829539 -0.21150617111924 -1.81174244451213
      H     -4.33088263238447 -1.55663911246229 -0.95494985873407
      H     -0.43639876973762 -1.87697690583887 -1.18172203970008
      H     2.02016566553966  -2.93053752239993 -0.62561917157018
      H     4.10592953538162  -2.35400583634390 0.58868903276528
      H     4.50799330758439  -0.05003266404351 1.39423752049622
      H     2.80203174828957  1.72261257737112  1.00857960766123
      H     1.07833163658718  1.76442977643425  -2.35124403748889
      H     1.40052051576412  2.97711166144436  -1.10030317946582
      H     -0.22840243909954 2.84091380600504  -1.79640815054341
      H     -1.05114937038784 2.71131122264010  0.52178999133378
      H     0.36224457417519  2.32452160219691  1.52848337347213
      H     -2.03643369878263 1.09841743368697  2.05668536885322
```

```
      H     -0.55396024383863 0.11678809423647  2.07804658431295

Coordinates of (S,S)-10, Conformer ID# 4
charge: 0, multiplicity: 1
32

      C     -3.19048278520232 -1.41800994327957 -1.14568998324653
      C     -3.12789802703035 -0.25387728652266 -0.17827215976518
      N     -1.90473268634456 0.29294584578829  0.06070910423178
      C     -0.65417696916912 -0.06505994805673 -0.58831395980943
      H     -0.79090744584404 -0.12069772849031 -1.67877813958701
      N     -0.01660951925214 -1.29615946675980 -0.07744694351315
      C     1.35924469903583  -1.05661506777424 0.01947394026668
      C     2.38036633650793  -1.98547945666229 0.19630854714376
      C     3.68772992505043  -1.50897481251085 0.31338347970416
      C     3.96954473571591  -0.14511829431166 0.26170045948358
      C     2.93195255560207  0.77789789146981  0.08631339226275
      C     1.63422640932198  0.31633953724975  -0.03988753710175
      C     0.33580396966858  1.06468940728740  -0.18857986470801
      C     0.35684095971082  2.20708550332002  -1.19836720939289
      C     -0.23530924376465 1.51643270544270  1.18180973827185
      C     -1.74971296918858 1.38752354144964  1.03375725215013
      O     -4.13369652592464 0.16229408355059  0.39656967546711
      H     -2.79922668504975 -2.32048919102769 -0.66178860343660
      H     -2.61431555104231 -1.24700090968974 -2.06001660223205
      H     -4.23569118385691 -1.59097337579276 -1.40333245918851
      H     -0.28533263771995 -2.15515296356596 -0.53833619820820
      H     2.17035158027615  -3.05074592726046 0.23909735026977
      H     4.49899873482865  -2.21996273687238 0.44388130789966
      H     4.99450887234280  0.20096393254071  0.35175355156015
      H     3.14734585467422  1.84288218962520  0.04058667368134
      H     -0.63952157010458 2.65009323201966  -1.30903094564014
      H     0.68975830311135  1.85410062240870  -2.17953781105787
      H     1.04022961508831  2.99552869344878  -0.86696839939089
      H     0.07900091749264  2.53298936992463  1.43529656784240
      H     0.12444856032187  0.84016583309107  1.96445762376248
      H     -2.21534171558529 2.30347558837875  0.64521198839735
      H     -2.25294651367036 1.13570913158138  1.97222616388322

Coordinates of (S,S)-10, Conformer ID# 5
charge: 0, multiplicity: 1
32

      C     -3.95847148941875 -0.47597832307720 1.06134569619848
      C     -2.83042260103597 -0.62151540568775 0.06531015212565
      N     -1.76068996016963 0.20667092056815  0.22320403023795
      C     -0.72309968956141 0.27621124415596  -0.82561157725729
      H     -1.18879474844868 0.63906859647208  -1.75529950314122
      N     -0.01384516050504 -0.96431760672730 -1.07043612923689
      C     1.22521353579026  -0.93798820217971 -0.45061478389712
      C     2.13836565256827  -1.97964593503488 -0.29279442116038
      C     3.36856494464122  -1.68915939507929 0.29863412988210
      C     3.68540624206368  -0.39924995544609 0.72332386033378
      C     2.75347445173680  0.63374521000079  0.56763748016106
      C     1.52997495763883  0.35759466052152  -0.01468399502443
      C     0.34546472250506  1.26789548123733  -0.25539885096617
      C     0.66072043071167  2.39449699769944  -1.24080879051277
      C     -0.26172238891557 1.79904786463611  1.07533007324158
      C     -1.74389270584063 1.43195091467814  1.02821769189590
      O     -2.85944746966859 -1.46342821235546 -0.83671933066022
      H     -4.70654624146275 -1.24028927746671 0.85052252085958
      H     -4.42119432211278 0.51436992632576  0.98330801104456
```

```
      H     -3.59723814819157 -0.59298144782476 2.08847034324185
      H     -0.56414699832622 -1.80811984678317 -1.13467438919197
      H     1.90254726557480  -2.98699613756463 -0.62426655785154
      H     4.09287824781469  -2.48889434442001 0.42824656900139
      H     4.65021976147362  -0.19769452570723 1.17813663356558
      H     2.98764139548237  1.63953672428734  0.91093875716763
      H     -0.22563655234804 3.01558629432368  -1.41771401428458
      H     0.99621348295715  1.98634047835800  -2.19988907383879
      H     1.45586973322729  3.03395516246194  -0.84389951021628
      H     -0.11148606651199 2.87682713921060  1.18932492482677
      H     0.21591850857542  1.29641724937628  1.92154447932596
      H     -2.33602268729023 2.22326887110401  0.53984007350857
      H     -2.16299610295329 1.25911487993706  2.02323550062126

Coordinates of (S,S)-10, Conformer ID# 6
charge: 0, multiplicity: 1
32

      C     -3.24168085689464 -1.24165863939831 -1.30335041442799
      C     -3.12970937153401 -0.24839844347319 -0.16618380158565
      N     -1.87818399173638 0.19171415215327  0.15656474407569
      C     -0.67460280274894 -0.05906559247632 -0.61796541633263
      H     -0.90033700501298 -0.02413915087378 -1.69346033401944
      N     0.01032877243796  -1.34248189737229 -0.31867679026055
      C     1.36124617988999  -1.06702237153192 -0.05692713312448
      C     2.37932377140714  -1.98513139330397 0.18129339997747
      C     3.67701184614896  -1.49910979082197 0.35317572412492
      C     3.95447168890226  -0.13532311610748 0.27814184924296
      C     2.91910147334552  0.77897001719664  0.05322270301173
      C     1.62816869070861  0.30739714051466  -0.10702087777669
      C     0.32314944403674  1.05744693219777  -0.20581767089434
      C     0.31149422213381  2.23805053820407  -1.16893241801234
      C     -0.19086306901820 1.44968194225890  1.20779959701205
      C     -1.70498627907113 1.27849577109724  1.13705159175248
      O     -4.11931662085303 0.15830988194769  0.44018345119031
      H     -3.29106496911356 -0.69390067988956 -2.25225210460497
      H     -4.17427386999488 -1.79438993917339 -1.18294701692781
      H     -2.40100732991901 -1.93777226488184 -1.36126213664908
      H     -0.46794663695056 -1.94608494724019 0.33818132775619
      H     2.17394122372147  -3.05139850154052 0.21820996777824
      H     4.48553087051005  -2.20226055434475 0.53361134789349
      H     4.97375065591077  0.21827561266102  0.39964005354040
      H     3.12735010802351  1.84600442845581  0.02139347255207
      H     0.60334484638925  1.92162360807799  -2.17546474512800
      H     1.01066849189657  3.01184377448138  -0.83599844450489
      H     -0.68661469356241 2.68811945137753  -1.22366842385843
      H     0.10661253003677  2.46626602418288  1.48088508388710
      H     0.23022825345865  0.76142991476333  1.94877255830681
      H     -2.21586696442380 2.18348094560311  0.78049074223427
      H     -2.15276860812447 1.00543714725620  2.09731011377109
```

Table S29: Electronic energy, correction to Gibbs free energy, and respective Boltzmann weights of the conformers of (***S,S***)**-12** at the ωB97M-V/def2-QZVPP/C-PCM(Hexane)//r2SCAN-3c/C-PCM(Hexane) level of theory for the calculation of the ECD-spectrum.

Conformer ID #	Electronic energy [E_h]	Correction to Gibbs free energy [E_h]	Boltzmann weight [%]
1	-1110.662961	0.338669	2.88
2	-1110.664819	0.339413	9.36
3	-1110.664359	0.339383	5.93
4	-1110.663324	0.338633	4.39
6	-1110.665150	0.339231	16.11
8	-1110.665435	0.339500	16.39
9	-1110.664293	0.339431	5.26
10	-1110.664365	0.339370	6.06
11	-1110.663325	0.338664	4.25
12	-1110.664048	0.339020	6.28
13	-1110.662946	0.338618	2.99
14	-1110.662911	0.338580	3
16	-1110.664092	0.339139	5.8
17	-1110.662908	0.338664	2.73
18	-1110.662917	0.338700	2.66
19	-1110.661564	0.339110	0.41
21	-1110.661048	0.339012	0.26
22	-1110.664255	0.339399	5.23

```
Coordinates of (S,S)-12, Conformer ID# 1
charge: 0, multiplicity: 1
47

      O     -5.17848845104546 -1.29941111679411 0.57872018445316
      C     -4.66705643813177 -0.21163042294671 0.33621901666394
      O     -5.08391674442003 0.96089211450433  0.88528172389730
      C     -6.20318666778140 0.83623075375172  1.78527127592205
      N     -3.62468041881730 -0.02679961570271 -0.50356853634472
      C     -2.97081159733689 -1.19103257148600 -1.13643367955706
      H     -3.67172873370504 -1.68111087381176 -1.82323773994432
      N     -2.41936474289110 -2.15994001364809 -0.19612776121003
      C     -1.14527403781339 -1.69839469382498 0.17001025963816
      C     -0.34510785560447 -2.09082122218520 1.24026552134149
      C     0.92838678872607  -1.54210930617656 1.35351920691049
      C     1.40478981296648  -0.62053911665132 0.41459192768934
      O     2.67743466975255  -0.15439524710781 0.64047724742606
      C     3.20858170492324  0.79008109668224  -0.30159396383380
      C     4.59652916367401  1.14304407595305  0.14546073293069
      C     5.69426240197758  0.41981479335359  -0.32252432631681
      C     6.97913715338426  0.72731881317711  0.11526415826999
      C     7.17496594735077  1.76142048723286  1.02741386950293
      C     6.08322122901909  2.48597890408197  1.50059403580583
      C     4.79990206872063  2.17704028535639  1.06057715256903
      C     0.59757420992555  -0.22841692763907 -0.66084979298464
      C     -0.67530167724811 -0.77314446826265 -0.75985208526631
      C     -1.73049276746922 -0.55350860817962 -1.82752608475559
      C     -1.34818586239033 -1.24719891937066 -3.13482925062126
      C     -2.11597302948411 0.92197755282982  -2.00837958182927
      C     -2.94728191113947 1.24868850888251  -0.77046117306999
      H     -6.40339179323889 1.84920851964241  2.13625024562678
      H     -5.95098643716692 0.18341529876377  2.62555588858403
      H     -7.07536458961213 0.43349710318978  1.26273364307067
```

```
H -3.05663363312113 -2.44014058856789 0.53911138253001
H -0.69895159746503 -2.80762277018980 1.97602575551922
H 1.57720398124792 -1.82023499586367 2.17863764629185
H 2.57046510367267 1.68634225775412 -0.32781519996496
H 3.22789944584345 0.34249711921833 -1.30650745898843
H 5.53892106720768 -0.38851831988506 -1.03321830872518
H 7.82840639110545 0.16106833788604 -0.25653220447723
H 8.17772841909142 2.00431551089014 1.36751281712695
H 6.23288685752963 3.29409658810859 2.21102341782258
H 3.94601374505590 2.74077329139664 1.42905262931579
H 0.94058474782793 0.48628182770937 -1.40196066371480
H -0.44626064054371 -0.78934796290749 -3.55450465688726
H -2.15559380272312 -1.16152364452565 -3.87073198381190
H -1.14116392940349 -2.30819298850003 -2.96167702600604
H -2.72553457281998 1.03254387658755 -2.91385846017420
H -1.23864175567917 1.56859636403512 -2.10505202935622
H -3.67404073875117 2.04753169656407 -0.94361274472512
H -2.30934048319949 1.53552921667530 0.07794497365679

Coordinates of (S,S)-12, Conformer ID# 2
charge: 0, multiplicity: 1
47

O -4.89760838586575 -0.97443329951332 -0.31474181790785
C -4.36899640975171 0.01922676081844 0.17184832338717
O -4.93261820770113 0.77273624252070 1.15420617179953
C -6.23098505230551 0.31876249449152 1.58601696690184
N -3.16152688906432 0.50084747442815 -0.19651913780125
C -2.35005150460661 -0.22642708183343 -1.19463936437900
H -2.85739883169339 -0.20970868841975 -2.16697101706297
N -2.01463970393571 -1.59531721916815 -0.82005777072790
C -0.87183258684345 -1.53391675726220 -0.00816378391188
C -0.32895023060564 -2.51322361409705 0.81914742006305
C 0.87770969189441 -2.24575876700328 1.46072538161771
C 1.53546242135844 -1.02571699789367 1.28168728613140
O 2.71775882498859 -0.88665695899666 1.97519946210848
C 3.42475523493337 0.34278375229489 1.84046239381294
C 4.05746035706142 0.54374644886044 0.48218529567046
C 4.32862248820513 1.83652482221687 0.03136399006627
C 4.94386308519545 2.04066548690352 -1.20040047925402
C 5.28292183051079 0.95140946937885 -1.99930823000629
C 5.00720343974544 -0.33985966277338 -1.55600209837850
C 4.40097701707179 -0.54423259574064 -0.31985156911967
C 0.98662198248448 -0.04243324783506 0.45024571664932
C -0.21918143303400 -0.31365859579816 -0.17776182648285
C -0.99654585510836 0.54100699281678 -1.16011402242588
C -0.29564033611350 0.59232535007583 -2.51755071563769
C -1.36125473992739 1.92633430537901 -0.60730632603670
C -2.46963742639553 1.64831211853030 0.40492422389006
H -6.53462040993470 1.01788518882269 2.36604462271087
H -6.17061605031092 -0.69733374098420 1.98570923045754
H -6.94258012579141 0.33958654317030 0.75585532146104
H -2.80014725434245 -2.14980049860438 -0.50310677132104
H -0.82669601836379 -3.46756719535164 0.96633619996920
H 1.32701127525282 -2.98432897800227 2.11797708445784
H 4.20472066051549 0.29209695156436 2.60955280780331
H 2.77121156532749 1.19349333492126 2.08754213366290
H 4.05238558487098 2.69066360916800 0.64695488995509
H 5.15011107819389 3.05168015414561 -1.54071855241779
H 5.75513353963962 1.10845152705551 -2.96487913103987
H 5.26400707705549 -1.19386917701610 -2.17660202181586
H 4.17837388252635 -1.55084635053088 0.02234083096129
```

```
      H     1.48848205031383  0.90432985001418  0.27977897706323
      H     0.67195659108780  1.09662231228699  -2.42221253072218
      H     -0.90275160973181 1.13818121061079  -3.24853215074236
      H     -0.11416336003977 -0.41901306534631 -2.89545721771889
      H     -1.74102611459340 2.55657710296872  -1.42109271455147
      H     -0.50017088094156 2.42974841214220  -0.15748665915061
      H     -3.15300187988340 2.49286692362333  0.53173646735484
      H     -2.05782838134786 1.38218765296127  1.38913471065717

Coordinates of (S,S)-12, Conformer ID# 3
charge: 0, multiplicity: 1
47

      O     -4.41020589199697 -0.04274320052553 1.36544065916599
      C     -3.93820534696503 0.59925705082896  0.43338795773520
      O     -4.09758053591773 1.94154849603292  0.28069528741889
      C     -4.88779552879004 2.57118251700382  1.30895996317856
      N     -3.19856165713335 0.06763744525540  -0.56462355258441
      C     -2.83606400854266 -1.36483056996239 -0.53263099131845
      H     -3.73937274787489 -1.97792886389238 -0.63907506924323
      N     -2.08197927065483 -1.77175313026238 0.64673170139575
      C     -0.73307371860181 -1.47643637330053 0.39951941304218
      C     0.32411809941433  -1.38601237179372 1.29256241421046
      C     1.61137056486690  -1.14634919357769 0.79597095021861
      C     1.82539572106698  -1.00837160959040 -0.57576493208600
      O     3.05198317815345  -0.78124667759098 -1.15982205398780
      C     4.19175743595729  -0.70340138073559 -0.30846416256032
      C     4.26037136886132  0.56351472930671  0.51301657664230
      C     5.01330151455790  0.58274573990604  1.68806861970190
      C     5.12748914770345  1.75170917700903  2.43468945815307
      C     4.47865799571355  2.91152361308974  2.01799297674320
      C     3.71981295156901  2.89402488076214  0.85038201364681
      C     3.61338158155742  1.72730278296356  0.09870123380303
      C     0.75135249676787  -1.09673336292525 -1.47275364199729
      C     -0.51503274483706 -1.32398407185286 -0.97489562276026
      C     -1.82991867058470 -1.48948274481940 -1.71323932360953
      C     -1.90006566488792 -2.84169975458555 -2.42214651554080
      C     -2.16775542496477 -0.30119266765661 -2.62489798954790
      C     -2.56400605684673 0.81320637026788  -1.65962056367724
      H     -4.42425193155792 2.43332041388268  2.28977503354149
      H     -5.89993958975302 2.15731393525326  1.32535285715916
      H     -4.91286859838234 3.62892533582413  1.04440734359607
      H     -2.48496527378258 -1.47537095587705 1.52692552330701
      H     0.16863979901315  -1.48832547680948 2.36285088266657
      H     2.42988548152182  -1.04741226723148 1.49950820849203
      H     5.04578182277270  -0.74474848492157 -0.99523787166295
      H     4.24858478507343  -1.58804490161277 0.34362392783766
      H     5.51078704204755  -0.32517730242541 2.02395067938550
      H     5.71605040300674  1.75431013472710  3.34795699019837
      H     4.55953691403776  3.82281558344249  2.60366112963571
      H     3.20596079874441  3.79359916191424  0.52286634157033
      H     3.01635459017390  1.71165194100693  -0.80859418319359
      H     0.93966237957969  -0.98076559619718 -2.53731743290080
      H     -1.14298785138466 -2.89247074756812 -3.21157717923436
      H     -2.88628821678648 -2.99112482326689 -2.87581823154534
      H     -1.71043298795863 -3.65707160238764 -1.71661869783100
      H     -3.01653594928023 -0.56413236390109 -3.26844606255199
      H     -1.32597914701451 -0.02048594322296 -3.26478399145354
      H     -3.26324374388055 1.52985080521846  -2.09995208839531
      H     -1.68344551378121 1.36300632479742  -1.29710798476376

Coordinates of (S,S)-12, Conformer ID# 4
```

```
charge: 0, multiplicity: 1
47

      O     -5.13746257444618 -1.24477196848097 0.82345279614655
      C     -4.66635506895494 -0.15725693202529 0.50831041806956
      O     -5.05890516435026 1.02518242992599  1.05420192408793
      C     -6.10189788293969 0.91137876185668  2.04269698474237
      N     -3.69802377412932 0.01727992689535  -0.41761285983341
      C     -3.07529133278768 -1.15634000101651 -1.06472996284635
      H     -3.81950693595060 -1.67878745624147 -1.67820918707778
      N     -2.43392204313691 -2.08626825781130 -0.14337490667284
      C     -1.14463191836240 -1.59598812099479 0.11252091620592
      C     -0.26141523597238 -1.93434499707973 1.12594627541176
      C     1.01659636680733  -1.35911972347562 1.13115622482229
      C     1.39346967973244  -0.46279087614605 0.12973530119520
      O     2.62035435143575  0.15045546841205  0.05111566209596
      C     3.57634091073338  -0.15236203351409 1.07983457524299
      C     4.82088656603747  0.63047977674832  0.78118811334550
      C     4.95353476136396  1.94289215311412  1.23747412062103
      C     6.09200932153918  2.68277648392776  0.93351070178159
      C     7.10877314302863  2.11409176080994  0.16948155330570
      C     6.98270017393606  0.80536706538383  -0.28990399143210
      C     5.84224767118767  0.06793194801466  0.01482740765576
      C     0.49260721716057  -0.12408752793595 -0.89196217031162
      C     -0.76496836235648 -0.68930387050891 -0.88530358361282
      C     -1.90558383344344 -0.52201571324694 -1.87159325734020
      C     -1.61387961107972 -1.25251476207884 -3.18201900372979
      C     -2.33048850600861 0.94043447355992  -2.06678367797658
      C     -3.06660929539666 1.29393723923940  -0.77698616576194
      H     -5.77351656876739 0.28869875080136  2.87957396083345
      H     -7.00506241336244 0.47943817567155  1.60256951132559
      H     -6.29188090332199 1.93156056404813  2.37820236731172
      H     -3.00788704645558 -2.36303840430639 0.64327595651085
      H     -0.54340553354367 -2.63167009597730 1.90985191328869
      H     1.69982181427561  -1.62431246284068 1.92960589688956
      H     3.78880669120086  -1.23163940533136 1.08419154336226
      H     3.16586042999413  0.12985123160423  2.06086899342980
      H     4.15849395613901  2.38457683237712  1.83376821253540
      H     6.18841852099380  3.70260989184983  1.29526766840675
      H     7.99987741019984  2.68960819089696  -0.06501115895513
      H     7.77450846450819  0.35831468987965  -0.88434652140725
      H     5.74111766943771  -0.95366926256206 -0.34399111177318
      H     0.80469306925596  0.57976074799225  -1.65956255364627
      H     -1.37341431776717 -2.30359609651271 -2.99234057043544
      H     -0.75692759338455 -0.79347722075656 -3.68579831341322
      H     -2.47863547163972 -1.20493681228528 -3.85334936945941
      H     -3.01258905889000 1.01078924090924  -2.92320896767952
      H     -1.47546572725472 1.59672024966556  -2.25383504827944
      H     -3.81903928606654 2.07553027589974  -0.91563658942655
      H     -2.36835272919852 1.61757567164516  0.00828997244666

Coordinates of (S,S)-12, Conformer ID# 6
charge: 0, multiplicity: 1
47

      O     -4.35549880652069 -0.03278012819318 0.69011781175273
      C     -3.47247716783529 0.77488845629389  0.42248222270239
      O     -3.37694809372380 2.00804483980464  0.98907857994121
      C     -4.42056207135629 2.31426499120260  1.93542545307274
      N     -2.47812440280110 0.55506078943621  -0.46563210224095
      C     -2.34559652963599 -0.76691190680611 -1.11169294573050
      H     -3.20588384792802 -0.94819370194815 -1.76716971833730
```

N	-2.17304209302653	-1.87679277395397	-0.18050332508438
C	-0.81387382867276	-1.94414090149099	0.14284756711698
C	-0.19789976197257	-2.59953363953266	1.20477647231813
C	1.19589852386525	-2.55791386481488	1.28735759681456
C	1.94709891659970	-1.88586272258384	0.32614502626810
O	3.33201872951740	-1.85604704561571	0.42206361384311
C	3.82187031433625	-0.83552288456892	1.32877963502855
C	3.46199615421867	0.54601310685039	0.85521476810643
C	4.12557104077357	1.10562237143418	-0.23984996179104
C	3.74099262382564	2.34433087482400	-0.74082454995895
C	2.68752544570064	3.04037122158518	-0.14842403434507
C	2.02522266609478	2.49303891328047	0.94669305528371
C	2.41024752984689	1.24964670188582	1.44303100650329
C	1.32344231533802	-1.23881614145152	-0.74405551876516
C	-0.05565600032016	-1.26935172304003	-0.82039101816839
C	-0.98066334554514	-0.65281116302273	-1.85131161628003
C	-0.94273676137333	-1.42582749598362	-3.16888245632707
C	-0.76328192819073	0.85864176764715	-2.01849156586409
C	-1.36000787340776	1.46439930203635	-0.75154880123190
H	-4.41034959777150	1.60149079987127	2.76470500683284
H	-5.40032333861598	2.29091621975766	1.45016494430382
H	-4.19698220562161	3.31970469895939	2.29396092146607
H	-2.85281993061916	-1.91573113690959	0.56846219854926
H	-0.77998943908118	-3.12830829656205	1.95437587505745
H	1.71022769306028	-3.05273552022445	2.10683762005470
H	4.90651270658722	-0.97977628973424	1.34720882169910
H	3.41971622148529	-1.01446157594497	2.33613619743510
H	4.94142137122678	0.55751659877250	-0.70596511247505
H	4.26483352958623	2.77200552230461	-1.59130658063900
H	2.38845108563224	4.00971209615707	-0.53764831657683
H	1.20587418313402	3.03298092340126	1.41353259020955
H	1.88220821838525	0.81276376969466	2.28772364874538
H	1.92944078386296	-0.70779762772624	-1.47364427120744
H	-1.16003018459416	-2.48559720463744	-3.00065682647827
H	0.05202562504749	-1.35064888179848	-3.62018647853079
H	-1.67686902774368	-1.02387860481666	-3.87617276933111
H	-1.30802682657488	1.21264901296717	-2.90261037090295
H	0.29461743128846	1.11146955181749	-2.13984892594477
H	-1.71665825077687	2.48856282601768	-0.89395866751750
H	-0.63185179570387	1.45886587535878	0.07229530062335

Coordinates of (S,S)-12, Conformer ID# 8
charge: 0, multiplicity: 1
47

O	-4.59807548997613	-0.17707339618227	0.48752166118441
C	-3.72170887977350	0.66061580112763	0.30240299952535
O	-3.72310806668207	1.89637567458898	0.87106476031401
C	-4.85916744008223	2.16536101125260	1.71634647614806
N	-2.64009412682636	0.47486113077415	-0.48570130472568
C	-2.40869460129401	-0.83742551400627	-1.12511094055819
H	-3.20068034201263	-1.03872967543400	-1.85661294174528
N	-2.28473596310912	-1.95112754293214	-0.19020541208625
C	-0.95376250331525	-1.96387442759304	0.25567027478165
C	-0.40292125496699	-2.59772528940303	1.36576651534417
C	0.97051219010003	-2.49550550891909	1.57521758597602
C	1.78411401525992	-1.78423295174125	0.68958210654800
O	3.12196555001426	-1.74396589379095	1.01719149984914
C	3.98470062086862	-0.97971939679944	0.17895213105504
C	3.74125797563797	0.51131841603714	0.24405279649994
C	4.09249440083703	1.31750348393360	-0.83966777239859
C	3.89719517583346	2.69489587399341	-0.78854201070501

C	3.33592924619859	3.27786984352419	0.34490650800511
C	2.97898125109550	2.47590882740575	1.42647430541809
C	3.18398280151633	1.10009207961985	1.37908635896652
C	1.22900578322892	-1.16055778049935	-0.43383104771841
C	-0.13972617689047	-1.25500012430277	-0.62663819755318
C	-0.98848536108270	-0.67120234378950	-1.73915333893792
C	-0.80294918043259	-1.44152743759301	-3.04589076128837
C	-0.81571742613718	0.84709442859978	-1.89155320332576
C	-1.54021499241094	1.42935335247744	-0.68089395084878
H	-5.78885984535965	2.10827069170739	1.14325268874010
H	-4.70456987744621	3.17765338164498	2.09156879985219
H	-4.90051538495478	1.45282972712012	2.54489744801565
H	-3.02087788295176	-1.99856722531991	0.50342342958803
H	-1.02258840006094	-3.15202332356563	2.06499373649301
H	1.43170467850757	-2.96492432929171	2.43910486807369
H	3.92341816969950	-1.33106159351814	-0.86251271126946
H	4.99192777643531	-1.21031788753521	0.54555805393935
H	4.51614067423212	0.86349558627869	-1.73359792901386
H	4.17458432940050	3.31203381794638	-1.63865309971135
H	3.17391613798530	4.35129495561846	0.38354901228949
H	2.53659354996746	2.92343780803793	2.31219620018213
H	2.89631793292565	0.47374176800118	2.21865221170266
H	1.83659539550603	-0.58484188536478	-1.12378131679400
H	-0.99314947103292	-2.50897940292187	-2.89441458547013
H	0.22439166476980	-1.32731582697502	-3.40749321308195
H	-1.48587919888184	-1.06828156137821	-3.81730540973300
H	-1.29804233563120	1.17971156166569	-2.81917655639376
H	0.23790640141469	1.14138995366491	-1.92666981919710
H	-1.92567120363363	2.43738893458457	-0.85810209236435
H	-0.88525031648948	1.45633220925176	0.20218518642858

Coordinates of (S,S)-12, Conformer ID# 9
charge: 0, multiplicity: 1
47

O	-4.69646643484106	-0.94370502169846	1.20267312305252
C	-4.48118868755201	-0.07497224562435	0.36431983774066
O	-5.15482528232455	1.10529793531827	0.30651848926794
C	-6.17308235168863	1.26348662562417	1.31435391742845
N	-3.55080638460033	-0.16731792621239	-0.61103308707686
C	-2.65596330882413	-1.34183476591229	-0.66902035851194
H	-3.24180012515610	-2.24262165322014	-0.88903126440336
N	-1.84359868619627	-1.54351556933677	0.52468357955925
C	-0.70969084176633	-0.72725530981009	0.39883352009604
C	0.20130748672842	-0.33430774909484	1.36802629475478
C	1.31137571697656	0.42835975672162	0.98454138802467
C	1.49567902559089	0.78618215301591	-0.35131624503560
O	2.54365958984945	1.54535395205299	-0.82297711478865
C	3.50155538536705	2.01887775987107	0.11942427800480
C	4.41921742226779	0.94447114217013	0.65610549169770
C	4.66400246038967	-0.22182743433831	-0.06803398761718
C	5.54228708439817	-1.18215712291028	0.42583765930729
C	6.18921801630479	-0.98380972114973	1.64305135741513
C	5.94829287545922	0.18012938281282	2.36896066602928
C	5.06296627659760	1.13602602282746	1.87962569723444
C	0.57269855129521	0.38256377389185	-1.32658298524086
C	-0.52143655082485	-0.36349945900409	-0.93977771657460
C	-1.64186301240862	-0.94534026587261	-1.78071599387442
C	-1.15381685937826	-2.13867918465185	-2.60162377326078
C	-2.38639771786402	0.10776934413604	-2.61387875590205
C	-3.22081676474233	0.87532601648275	-1.59162118149508
H	-6.93544518314471	0.48535811549058	1.21815344074075

```
      H      -6.60736292822587 2.24709130547914  1.13180134203534
      H      -5.73484115005772 1.21919491413751  2.31529898490545
      H      -2.36008969895295 -1.51854342683166 1.39495986262650
      H      0.07053002475004  -0.61126934107699 2.41025078171659
      H      2.02925239951943  0.71909550315929  1.74267554573064
      H      3.00254660710644  2.54656934659835  0.94621912956340
      H      4.08690862967511  2.75947050215920  -0.43870781512871
      H      4.15428950106553  -0.37948000948825 -1.01410525030606
      H      5.72053061109698  -2.09079935340169 -0.14250293849092
      H      6.87328811193514  -1.73506907548547 2.02716872188094
      H      6.44315001065487  0.34000138427653  3.32287863847006
      H      4.86714396946287  2.03815529495446  2.45613956276966
      H      0.73351516375324  0.67553068226016  -2.36117905602935
      H      -0.41124931574220 -1.81143558690495 -3.33675526082745
      H      -1.98720751962770 -2.60945094323811 -3.13503053482735
      H      -0.68410552831680 -2.88581471056693 -1.95376999615206
      H      -3.04145533818451 -0.39445665438114 -3.33658081534175
      H      -1.70038171407126 0.75702022831772  -3.16575468738477
      H      -4.12864999214018 1.30848025309498  -2.02097713044250
      H      -2.63862354361310 1.68112113535839  -1.12181536133997

Coordinates of (S,S)-12, Conformer ID# 10
charge: 0, multiplicity: 1
47

      O      -4.47235490598450 0.11526667582688  1.16745940917996
      C      -3.95056321633001 0.69115653199640  0.21883962478435
      O      -4.08198645039633 2.02299824922202  -0.02453338358762
      C      -4.90537554012160 2.72753216923358  0.92609836876224
      N      -3.17693780517972 0.08822845925662  -0.71055299321003
      C      -2.83909368654301 -1.34412424410835 -0.57450726048683
      H      -3.74673487454039 -1.95054318533308 -0.68203938775659
      N      -2.14251570194034 -1.68715391094862 0.65926402923488
      C      -0.77955235500591 -1.42750725254047 0.45329452861931
      C      0.24021171640742  -1.29828535159127 1.38435709103070
      C      1.55145411352103  -1.10974077469234 0.93047584672343
      C      1.82600572748905  -1.06021866420751 -0.43653067657073
      O      3.08034161668997  -0.88881461913062 -0.97921125208680
      C      4.18522249578233  -0.78762806734838 -0.08534299719294
      C      4.25122947612674  0.52286608845522  0.66498195648137
      C      3.64920301491529  1.67440481122331  0.15896008599773
      C      3.75286284529200  2.87995056499385  0.84712638114446
      C      4.46431924232595  2.94850828768538  2.04237872951512
      C      5.06827703196118  1.80102681433171  2.55059571026695
      C      4.95663631918992  0.59371055759733  1.86729777670410
      C      0.78962140146431  -1.18709204406816 -1.37233125298974
      C      -0.50071196154238 -1.36345262139913 -0.91705653712758
      C      -1.78563678702516 -1.55562670742484 -1.70030897713299
      C      -1.84805495312546 -2.94792270803925 -2.32767589988203
      C      -2.06451703083752 -0.42153686410838 -2.69701179448763
      C      -2.48365781712020 0.75576131328534  -1.82021314701879
      H      -4.48539277620684 2.64839166917491  1.93269906166428
      H      -5.92328468512067 2.32777451766025  0.92589616377747
      H      -4.90371294394163 3.76604777058241  0.59308254666829
      H      -2.57755031895630 -1.33228490290608 1.50169639716563
      H      0.03764902666644  -1.33211992031663 2.45110186523226
      H      2.34109176708035  -0.98028433038887 1.66149640146819
      H      5.06597342119200  -0.88811654198114 -0.73100766163572
      H      4.19212356232551  -1.63400228040902 0.61798039178561
      H      3.08888548656812  1.61938225190702  -0.76992088055008
      H      3.27399552986428  3.76988581064717  0.44811735383540
      H      4.54317652581795  3.89006894221354  2.57829026902834
```

```
      H      5.61952545918181  1.84361424484544  3.48587944697046
      H      5.41859540683634  -0.30374449360062 2.27445032295837
      H      1.02506971546266  -1.14062541575371 -2.43279693229191
      H      -1.05913831296004 -3.05774896224816 -3.07897910181883
      H      -2.81648812025298 -3.11133030539381 -2.81366131171810
      H      -1.70194837353725 -3.72080145286864 -1.56619616287663
      H      -2.88893875346226 -0.71220478706132 -3.35995736280434
      H      -1.19147282745068 -0.19249425756510 -3.31519500592683
      H      -3.15141354971639 1.45331488169215  -2.33388211557250
      H      -1.61066715486308 1.31488405360298  -1.45372766427367

Coordinates of (S,S)-12, Conformer ID# 11
charge: 0, multiplicity: 1
47

      O      -5.14978930357904 -1.22088391173932 0.78801357483354
      C      -4.68209276840687 -0.16431583636120 0.37703584838166
      O      -5.10770707071396 1.06173277225663  0.78432775558887
      C      -6.18044377160341 1.03413337725502  1.74679319885685
      N      -3.68555149570323 -0.07068775148362 -0.53057317070949
      C      -3.02539048135500 -1.29444764700784 -1.03128325598442
      H      -3.74054750875540 -1.88939217694445 -1.61237767807598
      N      -2.40531400095549 -2.11402977302687 0.00276498459529
      C      -1.13212102612599 -1.57888596641710 0.24993827030121
      C      -0.28095713858938 -1.79685195440285 1.32217161276816
      C      0.98839457507722  -1.20285055641509 1.31137696987096
      C      1.38864715374046  -0.40781609929071 0.23616052387560
      O      2.60874752811798  0.21575823928259  0.13688912583357
      C      3.53377471819412  0.03229962228361  1.22053812261607
      C      4.77739459795039  0.80138694649714  0.88467793127678
      C      5.82274853165924  0.18712628439160  0.19405698131428
      C      6.96263309376641  0.90938794772813  -0.14687547045093
      C      7.06429690154167  2.25439892501718  0.20012891310708
      C      6.02362518080639  2.87453060027518  0.88808181409066
      C      4.88557871831364  2.14986518378434  1.22826625693927
      C      0.52041456471282  -0.19126445933989 -0.84519630814448
      C      -0.72904726132529 -0.77379675976759 -0.82295169035041
      C      -1.83608008734011 -0.72766074289864 -1.85931479336988
      C      -1.48922705939243 -1.58466899647152 -3.07634366515639
      C      -2.27196728069454 0.69996584549211  -2.21890100126865
      C      -3.05735947685959 1.17217835036057  -0.99802946029196
      H      -5.87114616234348 0.50690246911088  2.65357066211164
      H      -7.06340111541797 0.54445940419173  1.32658015816192
      H      -6.39420714902142 2.08038617573263  1.96829558876508
      H      -3.00334672696632 -2.31619978428493 0.79419088727601
      H      -0.58144321775450 -2.41457224966873 2.16382537015768
      H      1.64631323874674  -1.37303380446555 2.15567156094801
      H      3.75930213909824  -1.03776403653900 1.33940489574079
      H      3.08799367444868  0.40433031061166  2.15508065783703
      H      5.74089266855023  -0.86281730936290 -0.07708811555648
      H      7.77310123020747  0.42223992651698  -0.68165312157082
      H      7.95499517825355  2.81825654904872  -0.06245466893383
      H      6.10094512836517  3.92290951203538  1.16215828617594
      H      4.07171104297064  2.63160375703307  1.76515983598866
      H      0.85036633165614  0.43508582990401  -1.67028730992882
      H      -0.62028865539460 -1.16710091299839 -3.59541186203492
      H      -2.33002919580803 -1.61981586828228 -3.77816918093805
      H      -1.24356594256759 -2.60678097189107 -2.77055527074993
      H      -2.92412787623312 0.67145270915457  -3.10067992138751
      H      -1.41904951275360 1.34715720376513  -2.44341836569189
      H      -3.81375228242679 1.92362429781229  -1.24178909031264
      H      -2.39109262809008 1.58541532951840  -0.22711638650517
```

```
Coordinates of (S,S)-12, Conformer ID# 12
charge: 0, multiplicity: 1
47

      O     -4.71838326452728 -0.96825937781964 -0.72849767448013
      C     -4.30772154936550 -0.07396734325246 0.00325974284784
      O     -4.99967958862126 0.41253486395729  1.06792548980405
      C     -6.28694343452524 -0.20158990671764 1.27781000267753
      N     -3.11744798421212 0.54870159720776  -0.14519639627646
      C     -2.18030042773114 0.10521861218711  -1.19632763357470
      H     -2.61385573068644 0.29797318682393  -2.18523149593285
      N     -1.76738811419140 -1.28943423173262 -1.08674712706801
      C     -0.68938920303681 -1.34384409025385 -0.19792685179478
      C     -0.13781410532384 -2.45153258262598 0.43939888442607
      C     1.00484798042328  -2.26296551948402 1.21978399191495
      C     1.57381199059256  -0.99891634811965 1.35808937837571
      O     2.70073646746364  -0.83122536361841 2.15110146483329
      C     3.94061612619502  -1.12997854031196 1.45819057101105
      C     4.18767751899753  -0.17015102749845 0.32767720949377
      C     3.88475565260282  -0.52620983377711 -0.98739194854003
      C     4.02602715878612  0.39618829629023  -2.02102799597797
      C     4.47307040132353  1.68557940579647  -1.74634538069847
      C     4.78230044194872  2.04850043041764  -0.43595420861533
      C     4.63908192854838  1.12504645469006  0.59389516499708
      C     1.01057065879445  0.11113352086248  0.72276705440931
      C     -0.12274869034906 -0.07338775070103 -0.04623456456696
      C     -0.89642218026950 0.92675279841112  -0.88275714425974
      C     -0.10646642133754 1.31712270995090  -2.13189583645586
      C     -1.40472665749642 2.13153686937125  -0.07799650018266
      C     -2.55921017949942 1.57200131384423  0.74915429118140
      H     -6.17686102020627 -1.27421374804012 1.46007158297264
      H     -6.93451223853625 -0.04609839816058 0.41039639834900
      H     -6.70128519006504 0.29369753941283  2.15664068583434
      H     -2.52344656432590 -1.95361191628901 -0.97907760577242
      H     -0.58099279417283 -3.43828704284316 0.33824618336450
      H     1.45927618951462  -3.10358231006501 1.73721334330666
      H     3.91272605971181  -2.16499882987311 1.08872950085182
      H     4.71429454621961  -1.04574116634963 2.22734664411229
      H     3.52123928288831  -1.52943414324185 -1.19819825252583
      H     3.78270549618009  0.10922648213331  -3.04031171347586
      H     4.58434669958191  2.40674011730259  -2.55135215361198
      H     5.13805669975219  3.05205646218140  -0.21932426279930
      H     4.87024627163224  1.40950187894968  1.61810906043889
      H     1.47523566163944  1.08701007413343  0.83928240262931
      H     -0.69939732309541 1.97378439541523  -2.77856655310419
      H     0.17916986877682  0.42624012059172  -2.70095026063113
      H     0.81125717242968  1.84251518006097  -1.84623496697843
      H     -1.77138354986748 2.90283019206967  -0.76666668337570
      H     -0.61931139299121 2.57136118688914  0.54365667666855
      H     -3.31198388653867 2.32600527416552  0.99586019288445
      H     -2.20080878303075 1.11792050765933  1.68412729331427

Coordinates of (S,S)-12, Conformer ID# 13
charge: 0, multiplicity: 1
47

      O     -5.23178001837998 -1.03511633866423 0.65445758479719
      C     -4.68245133891023 -0.02452481124931 0.22942056737976
      O     -5.07951332313790 1.23967651746288  0.53630449346127
      C     -6.22455341030997 1.31220384158164  1.40921773284453
      N     -3.61317434906293 -0.02546694147329 -0.59682901889491
```

```
      C     -2.97686853400993 -1.30311621832835 -0.97974319638814
      H     -3.67310723647358 -1.89654069675564 -1.58487986997666
      N     -2.47949805929466 -2.09089073905668 0.14214762929065
      C     -1.20303171997653 -1.59861940226615 0.45632831029037
      C     -0.44426613522934 -1.80098980116326 1.60649763429148
      C     0.84077291001950  -1.26997143171746 1.65632586557741
      C     1.36891408775752  -0.55253969256311 0.57740003784016
      O     2.64756948953177  -0.08112906271763 0.75326102990234
      C     3.23043062411291  0.65873856304796  -0.33035933587968
      C     4.61353525708674  1.06092527626830  0.08933279650898
      C     4.81495397600806  2.23757405621985  0.81265417441575
      C     6.09218866099558  2.59713701651840  1.23125123524213
      C     7.17980813293404  1.78070064909306  0.92881779407441
      C     6.98588150458999  0.60463229931389  0.20851698598822
      C     5.70695540186569  0.24685455017104  -0.20819553764760
      C     0.60328440323576  -0.35133100641620 -0.57813670717314
      C     -0.68134355388036 -0.87576429248299 -0.61469247011711
      C     -1.70039026693992 -0.83626209004797 -1.73795265827854
      C     -1.30349678501190 -1.77279851735526 -2.87886968196846
      C     -2.03794742668984 0.58735028787310  -2.20432201900812
      C     -2.89203863756978 1.16064433851324  -1.07643430829453
      H     -6.01255223647452 0.82298827882653  2.36398907482317
      H     -7.09419678553074 0.83871836465158  0.94501573662172
      H     -6.40477949200412 2.37740653698667  1.55830877811075
      H     -3.14439282973715 -2.21322379697225 0.89589729143448
      H     -0.83866902304638 -2.35825732071967 2.45162845942558
      H     1.45833789787190  -1.40269338947982 2.53958393480825
      H     2.61738043927992  1.54721946414194  -0.54468718417152
      H     3.26679753120118  0.03060614968638  -1.23291061990309
      H     3.96430354728996  2.87281339700926  1.04826487894707
      H     6.24048733636389  3.51596558654991  1.79152900083934
      H     8.17797356741128  2.06240192146887  1.25208386598148
      H     7.83198660794591  -0.03356447658566 -0.03005451258714
      H     5.55314490765936  -0.67213527276789 -0.76891480384732
      H     0.98698150386705  0.20344715361036  -1.42823819230040
      H     -1.13217006619887 -2.78663993576594 -2.50290105939492
      H     -0.37779135088349 -1.42394135399532 -3.34855125399017
      H     -2.08813501424068 -1.80875495452802 -3.64299611169345
      H     -2.62001483008764 0.53944294347015  -3.13309925341910
      H     -1.14004155651621 1.18338362475266  -2.39314547267092
      H     -3.59030617145326 1.92961691691801  -1.41907364617067
      H     -2.26875763597812 1.58680380893639  -0.27708797912091

Coordinates of (S,S)-12, Conformer ID# 14
charge: 0, multiplicity: 1
47

      O     -5.21675108854237 -1.22027491018758 0.60784498552428
      C     -4.70357663543928 -0.14929653214399 0.30236252270655
      O     -5.13580811440178 1.05609203145717  0.76054160484664
      C     -6.27358704052847 0.98831693842098  1.64327605567490
      N     -3.64388344873231 -0.01781075328020 -0.52594441106383
      C     -2.97149212668934 -1.21988885225061 -1.06110026627025
      H     -3.65493766603180 -1.76037666271750 -1.72734164466290
      N     -2.43794839975208 -2.11778699906891 -0.04332331200713
      C     -1.17393389168573 -1.62646941403602 0.31756905793822
      C     -0.39777540973858 -1.93771460861877 1.43128650441536
      C     0.87158079103031  -1.37724237951157 1.53368123507304
      C     1.36630428875726  -0.52261790391792 0.54274867885954
      O     2.62986327529636  -0.03136155526468 0.76591076029748
      C     3.20872202106894  0.78272649558448  -0.25985199002533
      C     4.61330008049906  1.12121510619137  0.14991386457414
```

C	5.52699759992324	0.10023870922587	0.42509578238809
C	6.83273329344492	0.40612924536834	0.78950510345349
C	7.24237397504405	1.73639887532476	0.87586351250540
C	6.33788671389736	2.75698619832722	0.60154229270525
C	5.02633003390841	2.44900114904056	0.24492624183208
C	0.58232556792971	-0.20964700586275	-0.57493833949539
C	-0.68578783942071	-0.76667543689074	-0.66452173206925
C	-1.71808346088961	-0.62574670830008	-1.76733054389406
C	-1.30527250993872	-1.40568680744200	-3.01537922267471
C	-2.10377853589406	0.83225767434662	-2.05720860057365
C	-2.96275409315405	1.23901019374262	-0.86278897241355
H	-7.13322490740901	0.54683137297446	1.13136002883758
H	-6.48424591782530	2.02202435896969	1.91975499140421
H	-6.03732794163898	0.39564449444329	2.53139861372275
H	-3.09059087124250	-2.35046742177957	0.69494582712457
H	-0.76652557048490	-2.60184735817875	2.20799637301856
H	1.50199646176232	-1.59307921433994	2.39111872465627
H	2.61877587891575	1.70056832537967	-0.40012980559180
H	3.20995336026164	0.22497500071803	-1.21064610257356
H	5.20606663256591	-0.93606632921954	0.35750276966137
H	7.53568190839277	-0.39398537262724	1.00396877317303
H	8.26400142282862	1.97447656442949	1.15824252447235
H	6.64921189244080	3.79535416051467	0.67241822337848
H	4.31746261633977	3.24828785967272	0.04198059045860
H	0.93805220364316	0.45759767327192	-1.35340455843571
H	-0.39603006276714	-0.97330831471133	-3.44614628754681
H	-2.09687052586588	-1.37454600507336	-3.77249079371660
H	-1.09854738339528	-2.45131873541793	-2.76544564417920
H	-2.69384569920071	0.87769158381852	-2.98102232221785
H	-1.22642119032103	1.47455391709152	-2.17912561780950
H	-3.68679685406140	2.02215663881621	-1.10510272777761
H	-2.34406283289933	1.58406071371086	-0.02179274770355

Coordinates of (S,S)-12, Conformer ID# 16
charge: 0, multiplicity: 1
47

O	-4.68781634173965	-1.30457803972324	-0.28879368085437
C	-4.30975340041889	-0.18635782213249	0.04383478891596
O	-5.02703427881650	0.65074516370203	0.83974664664103
C	-6.29903241025512	0.12581543434360	1.26969188986036
N	-3.13478131072304	0.36594365906889	-0.33138085057063
C	-2.17058673166106	-0.42201054678122	-1.12495469342729
H	-2.59133150497043	-0.63533631930348	-2.11513700513258
N	-1.72884277605411	-1.65595904685090	-0.48503760222410
C	-0.66602355569646	-1.33574338541833	0.36506799569430
C	-0.10228325554318	-2.09813674270121	1.38404918550247
C	1.02236957031965	-1.59392318810447	2.04072976144684
C	1.56202386888911	-0.35957968963123	1.68595934428023
O	2.67319724157190	0.12903039758286	2.35947204492458
C	3.92862629030551	-0.38819131541297	1.84594214941555
C	4.17004229434149	0.05293477391241	0.42918931384268
C	4.59288799231884	1.35778093182824	0.16342788013300
C	4.72946953543187	1.80253099793908	-1.14711982079296
C	4.44240189850612	0.94473502292932	-2.20833718723981
C	4.02357010855380	-0.35796107281696	-1.95254505966431
C	3.88859926092708	-0.80014283688202	-0.63900684024669
C	0.98613706514518	0.40569744014839	0.66819154555105
C	-0.12978957459975	-0.08985538937543	0.02093038981996
C	-0.91049226060126	0.49086519256919	-1.14178539026005
C	-0.10795738744725	0.39197435304316	-2.43906574808338
C	-1.45841767807084	1.89834414062377	-0.86597683310765

C	-2.61457747616132	1.66912485703719	0.10420673328017
H	-6.73554647367033	0.90658341510007	1.89354062192269
H	-6.16137611829431	-0.79250048089194	1.84727397422259
H	-6.94209972355212	-0.08011244010274	0.40947749484556
H	-2.47159640909863	-2.24928003605118	-0.13774656258613
H	-0.52240286251971	-3.05923742488619	1.66708783528072
H	1.48554925410913	-2.15898749733964	2.84511354424460
H	3.92751699834745	-1.48531009801231	1.91521255187817
H	4.68979592101471	0.01189766967815	2.52252662783165
H	4.80697905704799	2.02863870253645	0.99256243019195
H	5.06300171980718	2.81779343671938	-1.34359518048765
H	4.54880668463199	1.29162069107620	-3.23246785048645
H	3.79720881098092	-1.02934171121580	-2.77624110222278
H	3.54662993626526	-1.81275474424046	-0.43728491929853
H	1.42865011859569	1.36275318395931	0.40348501956941
H	-0.70402999621628	0.73306131915614	-3.29306046563222
H	0.20699522844139	-0.64120706046538	-2.61907812660179
H	0.79295631959226	1.01126396488164	-2.37098568066630
H	-1.82975251170626	2.33597474167331	-1.80093717313664
H	-0.69336806218220	2.56372736989949	-0.45520519677462
H	-3.38786508772042	2.43952082056857	0.03598779526867
H	-2.26256798742537	1.61950920836277	1.14462540493272

Coordinates of (S,S)-12, Conformer ID# 17
charge: 0, multiplicity: 1
47

O	-4.54261540061940	-1.68565093364760	0.22409121462837
C	-4.39107671073734	-0.46969738056178	0.17707122746681
O	-5.18792386071038	0.41951775599006	0.82716085688641
C	-6.25700986132272	-0.17812334902249	1.58782009030204
N	-3.41631138848301	0.15197147330000	-0.52313135135490
C	-2.39180579272764	-0.64580069495845	-1.22621174093777
H	-2.85957359824003	-1.21938267372548	-2.03566610507392
N	-1.61592321247906	-1.52753438239779	-0.36209293103643
C	-0.57183851248610	-0.77269905099353	0.18270244247830
C	0.24073818777562	-1.06463415243821	1.27318399949980
C	1.27858932400464	-0.18240560864013	1.57982166280962
C	1.48646427490836	0.96595723036596	0.82117925218789
O	2.49877113109308	1.83993484854109	1.18819563332942
C	3.62376277776692	1.85379222830825	0.27356853302689
C	4.33630196800600	0.53061716938190	0.25589153415539
C	4.09701283702836	-0.39300597445838	-0.76270209408837
C	4.70541600287588	-1.64541519469689	-0.73929176324489
C	5.55978802347744	-1.98405821574283	0.30610736307287
C	5.80397006563164	-1.06749177191834	1.32789575125967
C	5.19364730104313	0.18166710521773	1.30236354124142
C	0.66095512620780	1.25994324035194	-0.26969134279665
C	-0.36481329131530	0.38504119696609	-0.57648661960135
C	-1.37960728502171	0.43584429783331	-1.70304457309533
C	-0.72953419031406	0.09997272866866	-3.04507883409657
C	-2.19791231787517	1.73509045126007	-1.72715657772078
C	-3.15768795760639	1.59775217503660	-0.54801977928279
H	-6.78629043653377	0.65864749166997	2.04497683347154
H	-5.85655714687326	-0.84372282645780	2.35750543296588
H	-6.92708564791616	-0.74259250542658	0.93339720077107
H	-2.16477050728399	-2.11429880780482	0.25348064183371
H	0.08205533631983	-1.95771443845835	1.87101419423741
H	1.94125112269642	-0.37960200216768	2.41718063924805
H	4.27372460337918	2.65023221613253	0.64823063961826
H	3.28083224823339	2.12106647845421	-0.73580584919913
H	3.42174691290041	-0.13003155935789	-1.57388511695039

H	4.51012174641078	-2.35779441158525	-1.53590941018708
H	6.03645586299144	-2.96013731060063	0.32551127033996
H	6.47331350272023	-1.32771876927384	2.14322850659120
H	5.37774873114523	0.89571444541331	2.10206080561194
H	0.82314130475020	2.16915869747496	-0.84461596113189
H	0.00297828034686	0.86935720074929	-3.31107838971272
H	-1.48217106194054	0.04780723090659	-3.83979372906286
H	-0.20850711105512	-0.86148486411571	-2.99074617068124
H	-2.75998843658229	1.79573283141626	-2.66739053877282
H	-1.56355874630331	2.62292510748652	-1.64845916201766
H	-4.08700607099879	2.15731736635499	-0.68715918936172
H	-2.69008812628733	1.92607591117018	0.39136796237335

Coordinates of (S,S)-12, Conformer ID# 18
charge: 0, multiplicity: 1
47

O	-4.52089812990255	-1.72655266476436	-0.10703043901617
C	-4.37110911148808	-0.55784507504277	0.23257151042839
O	-5.12637373507791	0.05926601031493	1.17979727186263
C	-6.14772847793681	-0.77198264213777	1.76684502678366
N	-3.43975998748571	0.27438916580574	-0.28420251038296
C	-2.45785633583755	-0.23640615732973	-1.26172761483817
H	-2.97186761582603	-0.52708888052679	-2.18599213828613
N	-1.63482019942490	-1.33556310343566	-0.77134290837766
C	-0.55934321945876	-0.77853956384553	-0.07193522597325
C	0.31407331626116	-1.39216770985410	0.81998296582256
C	1.37042370951153	-0.63869018837792	1.33546906311228
C	1.53758377260777	0.69535345356159	0.97495767590773
O	2.57284244516923	1.42277380013211	1.54326535216469
C	3.65478487667421	1.71823907104465	0.62414200079660
C	4.35823970989503	0.46692158129397	0.17899540876799
C	5.25493397884690	-0.17565908119173	1.03634931648228
C	5.85875108477183	-1.37015439445923	0.65949342240624
C	5.56911071602956	-1.93714765553691	-0.58087355750623
C	4.67548769947600	-1.30465346328733	-1.44044004889568
C	4.07329487646783	-0.10788579646193	-1.06041557391540
C	0.65114015314371	1.31085781497503	0.08443457156386
C	-0.39405302209351	0.56499130946754	-0.42867608718435
C	-1.47344810534172	0.95882943115917	-1.41927509513784
C	-0.90465756461826	1.08644923330473	-2.83228743024293
C	-2.28961016054850	2.18189688002192	-0.97576487373567
C	-3.17949631652448	1.65433106761933	0.14671223305793
H	-6.64655919968221	-0.13832071316424	2.50096483571596
H	-5.70180999194759	-1.64359177886105	2.25393584648436
H	-6.85767178889424	-1.10631033880397	1.00517673679264
H	-2.14759321723329	-2.09867116432951	-0.34816165497434
H	0.18745265485021	-2.43230084106529	1.10672837009090
H	2.07990911878888	-1.08364891873872	2.02674034952135
H	4.32613670728658	2.37158215474644	1.18951238887324
H	3.26658492505809	2.27198124395088	-0.24228188275012
H	5.47477002030199	0.26500715417682	2.00627011938617
H	6.55872705123153	-1.85976374563798	1.33093414065226
H	6.04097900583867	-2.87015786437749	-0.87609585352045
H	4.44454172470638	-1.74468356714339	-2.40649961289036
H	3.36699465630959	0.38256904837493	-1.72662300985589
H	0.78367760256994	2.35888733610912	-0.17565060087448
H	-0.38226678127004	0.16878598396110	-3.12179591698882
H	-0.18850417012097	1.91380996772781	-2.87596118605189
H	-1.70341381335671	1.28026593133784	-3.55696637757484
H	-2.90522693281252	2.53369732812272	-1.81295996184310
H	-1.65024332634424	3.00741968831729	-0.64927880836928

```
      H     -4.11390911200921 2.21345703341342  0.24853126760552
      H     -2.65724949056082 1.66852361943430  1.11418849490678

Coordinates of (S,S)-12, Conformer ID# 19
charge: 0, multiplicity: 1
47

      O     -5.11947869310658 -0.75733397165304 0.02216398540570
      C     -4.57419799515941 0.33140930763028  -0.10072677111353
      O     -5.23289490660180 1.52127686275979  -0.08090335401030
      C     -6.65517233679234 1.41376131576370  0.12624833144461
      N     -3.24352963268808 0.50870136633995  -0.27194352581889
      C     -2.36547688673725 -0.64133079101574 -0.39994078608124
      H     -2.79346492206934 -1.35102190974873 -1.12345023196989
      N     -2.05752422294472 -1.31253991546060 0.87496612160253
      C     -0.68663506216274 -1.61666716519086 0.86734828560506
      C     0.02175725986066  -2.44976114987604 1.72930593701920
      C     1.40239040328469  -2.54874173577647 1.57504480927498
      C     2.07126404561649  -1.82579618684853 0.58408847611224
      O     3.43465441916316  -2.01764105201416 0.53370012935077
      C     4.16512436068153  -1.32455477326026 -0.47406950363342
      C     4.26898642466737  0.16592378474782  -0.24342469237192
      C     4.17980296638527  0.70291359814744  1.04058272925180
      C     4.30566045270927  2.07516106814022  1.23672681365208
      C     4.52903310687636  2.92281151167028  0.15433787265777
      C     4.62082803262351  2.39050594127526  -1.12939212848010
      C     4.48536922895654  1.01926259991517  -1.32648697178030
      C     1.35886208599237  -0.98537148318697 -0.27979707859936
      C     -0.01659625804202 -0.90171200910697 -0.12758437892667
      C     -1.00996217358101 -0.04353834373725 -0.86524270845766
      C     -0.85817725305349 -0.03720078714415 -2.38272096192751
      C     -1.07633921110121 1.39696814359482  -0.28727236903166
      C     -2.54819130476605 1.79701521036179  -0.40339348152785
      H     -7.11979054686307 0.83966331547214  -0.68026784602086
      H     -7.02443022965664 2.43995883590334  0.12515634074251
      H     -6.86958458868523 0.93141975186122  1.08404081264619
      H     -2.68760482570248 -2.06544332368809 1.11802389046244
      H     -0.48264468072499 -3.01669639925871 2.50678956852518
      H     1.98294470910084  -3.19839889719317 2.22325827789803
      H     3.74107766426577  -1.52952435139204 -1.46896637158143
      H     5.16433624058733  -1.77512415051832 -0.44251951838792
      H     3.99757748949798  0.04262189992522  1.88361849753480
      H     4.22773131430773  2.48424078938450  2.24025155749373
      H     4.62643645924122  3.99349363708694  0.30973241666473
      H     4.78951413169813  3.04481321887892  -1.98023816058327
      H     4.54398445331709  0.60824430797515  -2.33264776924945
      H     1.85759796805820  -0.40038828641504 -1.04499195386910
      H     -1.65939153910070 0.54332468767372  -2.85446579192116
      H     -0.89094411944942 -1.05622651093950 -2.78073470872226
      H     0.09760650073568  0.41444181729538  -2.66893076263719
      H     -0.41012525557837 2.08110265408778  -0.82096982009063
      H     -0.77775581657255 1.37690605547079  0.76614312739359
      H     -2.77626705640291 2.26173118019362  -1.37298393825037
      H     -2.85675020008481 2.49116033186941  0.38422760430598

Coordinates of (S,S)-12, Conformer ID# 21
charge: 0, multiplicity: 1
47

      O     -5.06878324505223 -0.61711024042302 1.27105488381970
      C     -4.89018803268759 0.07625833627851  0.27856053131956
      O     -5.81510991101814 0.93427166283898  -0.22955963140822
```

C	-7.04751231960845	0.99448019849334	0.51570810736934
N	-3.75162534621733	0.09025851943635	-0.45283086126421
C	-2.66264740856333	-0.81207122648090	-0.12259534399956
H	-3.05879842122530	-1.82847170180425	0.02012159950378
N	-1.86059609430712	-0.39217576927281	1.03925452120878
C	-0.51128880144158	-0.61261360085895	0.72115680784177
C	0.59001591844254	-0.61464602643525	1.56472019608719
C	1.86524096232611	-0.80290478555872	1.01709242026447
C	2.02477098452733	-0.98135195975931	-0.35698281391755
O	3.23203105664911	-1.18931833909062	-0.98657334969708
C	4.40339368337649	-1.25374194843114	-0.17822625543197
C	4.84004873647730	0.08125017040408	0.37997843019456
C	5.64499192443820	0.12144582815884	1.51951705069760
C	6.09679068894989	1.33798493522015	2.02240116353242
C	5.73695153615277	2.52857931305391	1.39566084282236
C	4.92727447904714	2.49282110572081	0.26312490402828
C	4.48324622186435	1.27551848853186	-0.24534271438268
C	0.90924903191129	-0.97171813214027	-1.20681735961904
C	-0.34446362130249	-0.79442887276299	-0.65914636048405
C	-1.69055866928863	-0.70390519466897	-1.32839866269456
C	-1.95525868966695	-1.76189928139050	-2.39421389234527
C	-1.98544373270705	0.72874568512563	-1.85200112183423
C	-3.48701048364188	0.92361540801842	-1.63416290151398
H	-7.53553786096390	0.01603675401510	0.53292870760458
H	-7.66724035817188	1.72053716188304	-0.01172044376887
H	-6.86116037555226	1.32433515962817	1.54159378108715
H	-2.18301713033100	-0.75249665800184	1.92746655101436
H	0.47917653210557	-0.47135287649889	2.63588971218392
H	2.72234170798265	-0.79086783344341	1.68032879813612
H	5.17628121647331	-1.64242073197791	-0.85224132966896
H	4.27347513466682	-1.98578626533351	0.63314086668730
H	5.91711370007359	-0.80601570021574	2.01992392993159
H	6.72249758104836	1.35673539565755	2.91041889423054
H	6.08138187491090	3.47970579860690	1.79148100327493
H	4.63769636295630	3.41816234346334	-0.22715451859796
H	3.84630449318170	1.24761361582184	-1.12472730028953
H	1.05499517332905	-1.11899171880369	-2.27375025541329
H	-1.81685515835429	-2.76766059244843	-1.98551424250833
H	-1.26839481450490	-1.63541991480963	-3.23745570791759
H	-2.97897640799074	-1.68325498258878	-2.77793827253069
H	-1.69347198831507	0.84695016328680	-2.89956142080026
H	-1.42539763078223	1.45439902408995	-1.25283286811105
H	-4.07901026079813	0.57588744605964	-2.49245377008156
H	-3.75025223839831	1.96856183940627	-1.44388230455984

Coordinates of (S,S)-12, Conformer ID# 22
charge: 0, multiplicity: 1
47

O	-4.56161792526866	-1.72436263538221	-0.60679119681728
C	-4.41410627368771	-0.67612440628095	0.01222967904577
O	-5.09606320474327	-0.36427667014963	1.14708562749854
C	-6.03483467705085	-1.37033609400378	1.57688569020708
N	-3.55957830757271	0.30824189296642	-0.34348771663671
C	-2.66232666476502	0.12334205207456	-1.50311849226265
H	-3.25545687067399	0.06058560668989	-2.42363443482925
N	-1.76557483457935	-1.02123949235965	-1.39793145384205
C	-0.65008091564194	-0.60868244255038	-0.65431614278019
C	0.32347803926665	-1.37646971000403	-0.03277732187918
C	1.39775567770313	-0.73146927189447	0.59222972754691
C	1.48518610463750	0.66101133105522	0.58852480974527
O	2.49230350086343	1.38569759036345	1.18645563606740

C	3.50840844405814	0.66864135040721	1.88178426351399
C	4.46882767126712	-0.07002219564649	0.97823333976048
C	5.19153478068508	-1.15372248696022	1.47967365201105
C	6.11481665506268	-1.81849929024346	0.67804194538994
C	6.31541631719398	-1.41187372172839	-0.63881124158643
C	5.59027119621080	-0.33601075449073	-1.14502567282169
C	4.67383003373234	0.33483966423055	-0.34034431520381
C	0.49878539666619	1.43103805305375	-0.04395259608849
C	-0.55980775524707	0.78824418630742	-0.65187107799524
C	-1.73278474859499	1.36861095786006	-1.41900283377080
C	-1.29542058435563	1.88542524342819	-2.78929944419735
C	-2.54253281780567	2.39105736582046	-0.60871969915277
C	-3.30804686624003	1.54597951675655	0.40638671290665
H	-5.52052404080337	-2.31211619608187	1.78763546499380
H	-6.79800733592787	-1.53795625517096	0.81179669187810
H	-6.48706863920067	-0.97131154169538	2.48554964887130
H	-2.21871817616973	-1.88989268730806	-1.14327734156897
H	0.26807602759770	-2.46136747610976	-0.02837180042599
H	2.16508415301628	-1.33555671790525	1.06227523678603
H	3.06259078187807	-0.02060017950678	2.61476683114281
H	4.04893949107618	1.44117542308020	2.44189658968892
H	5.02756049780426	-1.48281771402918	2.50411158881076
H	6.67096448777786	-2.66118197588154	1.07929343976035
H	7.02913883785350	-1.93470626995788	-1.26901938868868
H	5.73716436473610	-0.01772901828141	-2.17340392909027
H	4.10321973547109	1.16957823246588	-0.73703921302035
H	0.58403557026006	2.51487917649218	-0.03127931630156
H	-0.77509449341446	1.10135231833539	-3.34880024858447
H	-0.60956233003149	2.73076337156801	-2.67128671339892
H	-2.16106830497034	2.21872102422949	-3.37265373006263
H	-3.24332496137148	2.91134164746561	-1.27339792786301
H	-1.90211774243673	3.13886828499152	-0.13184393509603
H	-4.24757089153216	2.00753346828993	0.72342574540333
H	-2.70042840273294	1.34300744569053	1.30000486293632

Table S30: Electronic energy, correction to Gibbs free energy, and respective Boltzmann weights of the conformers of ***(S,S)*-13** at the ωB97M-V/def2-QZVPP/C-PCM(Hexane)//r2SCAN-3c/C-PCM(Hexane) level of theory for the calculation of the ECD-spectrum.

Conformer ID #	Electronic energy [E_h]	Correction to Gibbs free energy [E_h]	Boltzmann weight [%]
1	-804.354277	0.259795	55.74
3	-804.350991	0.259425	2.54
4	-804.350591	0.259316	1.87
5	-804.353855	0.259690	39.85

```
Coordinates of (S,S)-13, Conformer ID# 1
charge: 0, multiplicity: 1
36

      C     -3.77115038295671 -1.26787542448402 1.73491080374653
      C     -3.18975979939053 -0.89822174249845 0.38862907752511
      N     -2.25648392054438 0.08523701782052  0.37537571134867
      C     -1.60641562872729 0.48109094011830  -0.89517654152701
      H     -2.35986404090237 0.89046365066154  -1.57880713419806
      N     -0.85780568684752 -0.58913202148031 -1.54296952911153
      C     0.41447034087278  -0.62066566104134 -0.95371842311293
      C     1.37749302538775  -1.61750349889316 -0.99527908783522
      C     2.61789122584368  -1.38734646460228 -0.38516230922187
      C     2.88024187376635  -0.17295873594500 0.25086584254784
      O     4.06209529074905  0.13904378352030  0.87568119081154
      C     5.08843434382303  -0.84825280592797 0.86222878109940
      C     1.90005711494416  0.83096136946039  0.28953238576853
      C     0.67965226844075  0.59344510747069  -0.30705570852540
      C     -0.52800672930724 1.50269654674888  -0.43626615147028
      C     -0.27739153939944 2.61327738753387  -1.45600001484327
      C     -1.04922191373823 2.01644889150112  0.91289766446367
      C     -1.71622760823022 0.79575784787282  1.54207764575040
      O     -3.54274516313040 -1.45866892254831 -0.65509882537676
      H     -4.42242260013559 -2.13236091407248 1.60499589828529
      H     -4.35659032415168 -0.43698736009542 2.14456568091669
      H     -2.98362031900003 -1.50562320564034 2.45800778117127
      H     -1.36891163150444 -1.45799772822567 -1.63504145515677
      H     1.18512189375319  -2.56606963098876 -1.48871097626206
      H     3.36473276940818  -2.17221189932574 -0.41582679258755
      H     5.39642768233695  -1.09214201841313 -0.16371933348711
      H     4.76834105563007  -1.76633629621246 1.37376795154943
      H     5.93227218335461  -0.41082792681202 1.39880318385454
      H     2.12275486593963  1.76844678387023  0.79324634150442
      H     0.03371190309144  2.19031281796534  -2.41660686184761
      H     0.51890711697849  3.27687470346139  -1.10308978160629
      H     -1.18396885896613 3.20890257286227  -1.61132102150168
      H     -1.79062828407183 2.80690218767461  0.74205407893621
      H     -0.24931337689032 2.42454801025626  1.53779690573343
      H     -2.51012471357019 1.06930999085826  2.24445147085596
      H     -0.97950243285556 0.17366264755009  2.07231155180249

Coordinates of (S,S)-13, Conformer ID# 3
charge: 0, multiplicity: 1
36

      C     -4.94904477199300 -0.27714780889713 0.80526452414596
      C     -3.74134617293399 -0.51146432080879 -0.07349587596773
      N     -2.60497322833971 0.14854770639369  0.26980999584690
      C     -1.40544597063222 0.01693743569182  -0.54581881837704
      H     -1.66502596044066 0.18529377926040  -1.60141253817104
```

```
      N     -0.70363208788464 -1.26516189685078 -0.37303284692677
      C     0.67177733720542  -0.98975513857308 -0.32806067567174
      C     1.73802915507277  -1.86991990400886 -0.43521623435282
      C     3.04312971257581  -1.37601512718029 -0.30482487870283
      C     3.26626495070464  -0.02020399779729 -0.06260531195171
      O     4.50633385888404  0.55185364136659  0.07647184330471
      C     5.63720685544997  -0.30695752108440 -0.03191631745976
      C     2.18309296182660  0.86595879523589  0.04742069325968
      C     0.90257509198668  0.37375483917039  -0.09174294389842
      C     -0.42410108972025 1.07932759305301  0.01619394361478
      C     -0.50865528443845 2.41078177190312  -0.72246291960655
      C     -0.89894098511699 1.20562203927187  1.48907587996410
      C     -2.41869117140480 1.04584786800382  1.41925298033560
      O     -3.77013513147750 -1.26814074184719 -1.04724711772793
      H     -4.79592308161953 -0.73626726201969 1.78877269529790
      H     -5.81732829465233 -0.73647135498872 0.33257512352408
      H     -5.13501054243008 0.79011828812556  0.96459663038010
      H     -1.00549948823659 -1.99676944244055 -1.00251888878693
      H     1.57759429869191  -2.92796313650310 -0.62279250422796
      H     3.87138454199688  -2.06854218164829 -0.40025557394439
      H     5.62497008793043  -1.08524496403321 0.74339135053788
      H     6.51198831702883  0.33040614847000  0.10902910162491
      H     5.68741715888272  -0.78037413360345 -1.02202542358328
      H     2.37784046490231  1.91973883671810  0.22869428175525
      H     -0.24366694672267 2.28635428582399  -1.77692663757951
      H     0.17743549126700  3.13955457250104  -0.27856796972440
      H     -1.52196852867468 2.82546948323621  -0.66923940991689
      H     -0.59553572679593 2.15650057499093  1.93681052168173
      H     -0.46649421719832 0.39131231458159  2.07998151028987
      H     -2.92082272002437 2.00959808337604  1.24756102963018
      H     -2.82828888366930 0.60898087511074  2.33619078138405

Coordinates of (S,S)-13, Conformer ID# 4
charge: 0, multiplicity: 1
36

      C     -4.90448301104187 -0.15014655616465 0.84573950990050
      C     -3.72821285019206 -0.38806637311603 -0.07373249593407
      N     -2.54508957283296 0.16468958906829  0.30061102468415
      C     -1.36930506788409 0.02514546595031  -0.54767764968772
      H     -1.63444812483370 0.29363773949478  -1.58090993773123
      N     -0.74700781912066 -1.30801821724934 -0.49367013509985
      C     0.64351905300750  -1.11671222901669 -0.45171402987152
      C     1.65360303696633  -2.05654061926892 -0.64429912786444
      C     2.97700637671484  -1.65295931326624 -0.49548842754860
      C     3.29638620485913  -0.33574118497298 -0.14982019146674
      O     4.63710317895318  -0.06284026890995 -0.03598023045382
      C     5.00388198853217  1.26809871527158  0.31095182108573
      C     2.27989611744994  0.60729673674343  0.04845973356533
      C     0.96318117780094  0.19954584234411  -0.11465335912480
      C     -0.31366621584043 0.97635052492475  0.07617280875791
      C     -0.32635286143514 2.36504885898994  -0.55441421595977
      C     -0.75748840440152 1.01522320774368  1.56383662205397
      C     -2.28517240647738 0.95453679497065  1.51273911968508
      O     -3.82177573010770 -1.05572899813694 -1.10660283200224
      H     -4.76565129087955 -0.69512263956807 1.78658822916597
      H     -5.80770151661113 -0.51465450907952 0.35622532666396
      H     -5.01912681077104 0.91055014702174  1.09243594794761
      H     -1.09734615951824 -1.96078868623061 -1.18220988938400
      H     1.42289742173166  -3.08432720699907 -0.91047819203450
      H     3.78816899526893  -2.35822149651588 -0.64979303734314
      H     6.09457247904927  1.27806073184348  0.35149025772895
```

```
      H     4.60115982216569  1.55226340159341  1.29306985148040
      H     4.66180999866601  1.98864516415978  -0.44480309898397
      H     2.50153243101688  1.63630150397967  0.31112355756436
      H     -0.08482238448448 2.30870119507193  -1.62032897300367
      H     0.40918423965345  3.01445583318230  -0.06802687955978
      H     -1.31110116431843 2.83514837536592  -0.44964195401446
      H     -0.38944225182193 1.90765249993268  2.07869712607459
      H     -0.36763820093489 0.13182063065601  2.08044030137415
      H     -2.72881988513291 1.95773253161659  1.42782028347327
      H     -2.70667079319583 0.47183280856987  2.40090313586240

Coordinates of (S,S)-13, Conformer ID# 5
charge: 0, multiplicity: 1
36

      C     -3.88312053117407 -1.06958282679395 1.67473488551422
      C     -3.27779878869379 -0.69645260837060 0.33994708420991
      N     -2.25413051411723 0.19270519141852  0.36051352737247
      C     -1.57651196731762 0.58148316257237  -0.89778073019010
      H     -2.29190312457486 1.08908829270107  -1.55598475558188
      N     -0.93520012229224 -0.52555371616614 -1.59740752589740
      C     0.33320770733920  -0.69483355265025 -1.02224094812888
      C     1.20400832380372  -1.77743104659420 -1.12248891654695
      C     2.45547014955813  -1.68154484704262 -0.52197763103523
      C     2.84160145451577  -0.52501770336541 0.16437509566958
      O     4.10003406998473  -0.55544961924924 0.71250503355540
      C     4.53741164686610  0.60865673485454  1.40488013379910
      C     1.96405900057810  0.56198658382946  0.26332497128026
      C     0.71368492050161  0.45354351537531  -0.32972040405021
      C     -0.40413331004540 1.47672894122108  -0.40539418242572
      C     -0.05796644477434 2.60472197407814  -1.37712151745011
      C     -0.86785641677484 1.97601489449405  0.97026341545172
      C     -1.64227368671377 0.79596587633476  1.55176765804532
      O     -3.69008911015409 -1.17217467547305 -0.72368451772606
      H     -4.61013844879213 -1.86591125496855 1.51448686743118
      H     -4.39023684449594 -0.20764896714881 2.12302160313919
      H     -3.11718532063579 -1.40705340501332 2.38109914577896
      H     -1.52401744526453 -1.34080521006366 -1.71329549558059
      H     0.92075804567306  -2.68104440218538 -1.65500004287883
      H     3.15684111803030  -2.50887370386902 -0.57567154100359
      H     4.56075126225158  1.48468046152239  0.74193425758067
      H     5.54946963255626  0.39022851126655  1.75037702197972
      H     3.89706317108737  0.82510954393653  2.27135422133020
      H     2.23915668545465  1.46901198765303  0.79205385344440
      H     -0.90517221089435 3.28965085347966  -1.49559076098297
      H     0.20535393567195  2.19861098096996  -2.35905936391667
      H     0.79966129809800  3.17469732525362  -1.00438627228541
      H     -1.53349442644275 2.83869576797014  0.84155076882294
      H     -0.03030970638451 2.28049137197080  1.60512085727025
      H     -2.40236885451886 1.11048110128668  2.27411503499067
      H     -0.96357514790946 0.08420446676551  2.04557916901442
```

Table S31: Electronic energy, correction to Gibbs free energy, and respective Boltzmann weights of the conformers of ***(S,S)*-14** at the ωB97M-V/def2-QZVPP/C-PCM(Hexane)//r2SCAN-3c/C-PCM(Hexane) level of theory for the calculation of the ECD-spectrum.

Conformer ID #	Electronic energy [E_h]	Correction to Gibbs free energy [E_h]	Boltzmann weight [%]
1	-960.371594	0.343049	24.63
3	-960.371567	0.343095	22.79
4	-960.370644	0.342885	10.7
5	-960.370427	0.342895	8.42
7	-960.369412	0.342370	5.01
11	-960.369063	0.342342	3.57
12	-960.369525	0.342844	3.42
13	-960.370313	0.343167	5.6
17	-960.366935	0.342024	0.52
21	-960.369924	0.343081	4.06
22	-960.369128	0.342832	2.27
23	-960.369504	0.343017	2.78
25	-960.369639	0.342907	3.61
27	-960.367838	0.342582	0.76
28	-960.367510	0.342504	0.58
29	-960.367104	0.342064	0.6
30	-960.366496	0.342412	0.22
31	-960.365167	0.341903	0.09
32	-960.365939	0.342466	0.11
33	-960.365403	0.341918	0.12
36	-960.365790	0.342105	0.14

```
Coordinates of (S,S)-14, Conformer ID# 1
charge: 0, multiplicity: 1
46

      C     -4.76609445770818 -0.81160533057148 1.33022894121457
      C     -3.96147249429888 -0.28730245487125 0.16196808134829
      N     -2.73156521007774 0.21016317867322  0.44156527117221
      C     -1.86477191231378 0.71663089136925  -0.64639417388654
      H     -2.34297137869775 1.58189139357417  -1.12186446078257
      N     -1.50559396735466 -0.28077780575792 -1.64699104829291
      C     -0.39738974339705 -0.98100612065007 -1.14669040089196
      C     0.13354739678851  -2.19635599446247 -1.57124263324463
      C     1.29243842020433  -2.66520167179797 -0.95919883855612
      C     1.91282369775742  -1.93924897044073 0.06267976859237
      O     3.04899211543192  -2.50670032063193 0.58782343389363
      C     3.72049089031044  -1.78393020313018 1.61301430693839
      C     1.37492624653991  -0.71977531599301 0.48996446519513
      C     0.22378704512006  -0.25434231430255 -0.13069154093310
      C     -0.53431950327256 1.03907607298447  0.08811522985652
      C     0.19489668390412  2.24333988791089  -0.55333198070152
      C     1.48387295215140  2.59014340909005  0.12708389161110
      C     2.72290055569321  2.28369309912512  -0.28008605310017
      C     3.92327367725957  2.68869642731590  0.53060395337323
      C     3.04993826963772  1.51880434679162  -1.53029094643606
      C     -0.95439633620940 1.26826640210221  1.54492880215511
      C     -2.08746777847879 0.26624549875180  1.76066796273063
      O     -4.39573057663611 -0.30546523881389 -0.99513646853925
      H     -5.66961182817431 -1.28381799323630 0.94418220874071
      H     -5.04994182525683 0.00734790593900  2.00095922097199
```

```
      H     -4.19336371484926 -1.53696644204425 1.91774950403054
      H     -2.28925751136791 -0.81280212975561 -2.00421824156663
      H     -0.34050888520963 -2.77352298613995 -2.36016583836934
      H     1.73164716833759  -3.61078291694177 -1.26309768742741
      H     4.59296168222660  -2.38245310461144 1.88152142997165
      H     3.08250006806555  -1.65373205917910 2.49822925248756
      H     4.05120053405039  -0.79741525493993 1.25798825827766
      H     1.84045046082402  -0.13353952113738 1.27396252778805
      H     0.34632448082447  2.02189527094963  -1.61493819081449
      H     -0.48605596127380 3.10490006737625  -0.50088248904773
      H     1.38539728524840  3.13559838552234  1.06729649246954
      H     3.64219821998696  3.24676283448463  1.42814866370071
      H     4.60485742397390  3.30956762859086  -0.06538286122612
      H     4.49872056913882  1.80422426884057  0.83678204578838
      H     3.61357535927225  0.61135177771496  -1.27552155689824
      H     3.69583347662119  2.11691301884411  -2.18649239341122
      H     2.16845971661908  1.20845560464993  -2.09357653716793
      H     -1.32612181641308 2.29412490569092  1.66456414062726
      H     -0.12876137704618 1.11863449768957  2.24629900410066
      H     -2.79076160649237 0.59239014727882  2.53373618021991
      H     -1.69040651145956 -0.72025277185113 2.04317130403813

Coordinates of (S,S)-14, Conformer ID# 3
charge: 0, multiplicity: 1
46

      C     -4.77186551833528 -0.79537429164598 1.28584433562392
      C     -3.94589986423350 -0.31714937268905 0.11274184194543
      N     -2.74650338724442 0.24567098089055  0.39938661196102
      C     -1.85816903160324 0.71025284597234  -0.69027441730951
      H     -2.34490702234265 1.52814974243653  -1.23569190486988
      N     -1.43420260657124 -0.33516427175676 -1.61260582972391
      C     -0.32760080259683 -0.97326354447363 -1.03319315925918
      C     0.24928146905731  -2.19184444808797 -1.35553166186016
      C     1.40349677425366  -2.59699586848977 -0.67089585205128
      C     1.95675349178531  -1.78857270117106 0.32265721404928
      O     3.08197054473168  -2.10303732008733 1.04587411524516
      C     3.72137242604030  -3.34078846839145 0.75206576292560
      C     1.36445500473293  -0.55882101942429 0.64687607614498
      C     0.23600351424620  -0.16100878186999 -0.03916118636940
      C     -0.56502823185385 1.12055736647703  0.06515984539837
      C     0.15593161204027  2.29819641984667  -0.63266169745616
      C     1.41363484914972  2.71861754377303  0.06428053565507
      C     2.67077564400527  2.40149067178655  -0.27291777913427
      C     3.83374658201957  2.87362839303027  0.55557199255215
      C     3.05484470699517  1.55244114809532  -1.45057203322441
      C     -1.04237484833593 1.43365455504130  1.48779252170418
      C     -2.15312634551702 0.41332157438636  1.73314786947348
      O     -4.33745593588566 -0.42870422015455 -1.05455599275524
      H     -5.64146836057119 -1.32929263844836 0.90239762237146
      H     -5.11203219500329 0.05417763084630  1.88886576119137
      H     -4.19322628155099 -1.45516376646364 1.94091976063464
      H     -2.18870257202948 -0.90779697628878 -1.96954518573074
      H     -0.17817058053458 -2.83239408385920 -2.12175831680728
      H     1.84852260840344  -3.55214776831848 -0.92472118710828
      H     4.58233755743994  -3.39997722940080 1.42038037477484
      H     4.06669822393484  -3.37625014479711 -0.29034532205486
      H     3.05392313712334  -4.19243221462750 0.94252853691352
      H     1.81146991464355  0.05919632715205  1.42000366106242
      H     0.34697697449390  2.01066051083597  -1.67187912619811
      H     -0.54670099767518 3.14323793960584  -0.65881529207506
      H     1.27254640163058  3.32680725137481  0.95934166315328
```

```
      H     4.53946658902894  3.45115061876349  -0.05561976055432
      H     4.39436475530629  2.01697678480531  0.95339550474572
      H     3.51181997407513  3.49658757614028  1.39474757080849
      H     3.73865707663377  2.10183063749342  -2.11076317275232
      H     2.20074753166249  1.20990573167636  -2.03709126992324
      H     3.59613308289993  0.66202374330085  -1.10453047711802
      H     -1.44748494399455 2.45311349850346  1.52439795050995
      H     -0.23811082639498 1.35702941634670  2.22458487812482
      H     -2.89375378233405 0.76907303559308  2.45668219570975
      H     -1.73788631172561 -0.53809281372816 2.09768642165672

Coordinates of (S,S)-14, Conformer ID# 4
charge: 0, multiplicity: 1
46

      C     -4.87497942627954 -0.91549390365980 0.47189462104773
      C     -3.83285420492088 -0.16638126162086 -0.32834070786160
      N     -2.73340758361085 0.25581473404250  0.34363449210039
      C     -1.64315392120476 0.95461654407205  -0.37519623988807
      H     -2.02924972368605 1.89234164741575  -0.79338254299949
      N     -1.00849386393228 0.15482816472241  -1.41552444331276
      C     0.00315944467925  -0.60029954952762 -0.81094710102302
      C     0.70380885934351  -1.68814623647497 -1.31068845718099
      C     1.73867044349252  -2.23885481574965 -0.54271881008020
      C     2.05849426825097  -1.70142370945047 0.70503518529807
      O     3.05371860318182  -2.17429613961358 1.52528297292655
      C     3.80782543269610  -3.28886469353786 1.05998970733252
      C     1.34403620810461  -0.60214853722538 1.20376736581175
      C     0.32858927232992  -0.06536879071791 0.44180953439329
      C     -0.55308783116220 1.13357501556735  0.72041879311144
      C     0.23702209227298  2.45900793295002  0.60772675434706
      C     0.88227060414612  2.66346319375539  -0.73009401103033
      C     2.16672575928886  2.47339952471616  -1.05604677146708
      C     2.63430108294194  2.66599394422020  -2.47274733870243
      C     3.24509354866461  2.03852173183145  -0.10499826398565
      C     -1.32417747811725 1.02495650423582  2.04381193560101
      C     -2.41807378755020 0.00068822013788  1.75481232933462
      O     -3.96809849953164 0.05665957062130  -1.53650720316663
      H     -4.43446122511120 -1.76563089988001 1.00373266483844
      H     -5.64623847306392 -1.27119626518223 -0.21146017379020
      H     -5.33421255247791 -0.25943437102969 1.21994704468301
      H     -1.65288595968097 -0.30683977196803 -2.04446602595495
      H     0.46495340065520  -2.11444393607698 -2.28096992418108
      H     2.27996003918913  -3.09109658734298 -0.93712032550409
      H     4.53796231941082  -3.50468264473942 1.84211828369934
      H     4.33475295002506  -3.05415236312706 0.12492527293978
      H     3.16886124525463  -4.16923864616097 0.90571529437094
      H     1.61070592213397  -0.19533941037710 2.17629054865679
      H     -0.46692623607310 3.28038355816688  0.80370380320229
      H     0.97540035385864  2.47273497013121  1.41747968527404
      H     0.20298927622850  2.95704836917338  -1.53094336871772
      H     3.06342362730361  1.73359980422434  -2.86338792975293
      H     3.42840068712763  3.42250032375994  -2.52463978675750
      H     1.81817701964539  2.97180763770959  -3.13292538038541
      H     4.10442885481543  2.71868062237916  -0.16714957316736
      H     3.60956170063441  1.04048467973192  -0.38292598857756
      H     2.91522812665017  1.98954109005350  0.93384167653407
      H     -1.77223399791396 1.99607206500127  2.28892188485738
      H     -0.67828006383805 0.72434044486654  2.87399866938250
      H     -3.29871315163209 0.13866055723010  2.39023826406312
      H     -2.04408316253892 -1.02496831725353 1.89351358368089
```

```
Coordinates of (S,S)-14, Conformer ID# 5
charge: 0, multiplicity: 1
46

      C     -4.84995453473144 -0.92640178704396 0.51646512531483
      C     -3.82491478506700 -0.13674024463912 -0.26666105254804
      N     -2.68815057175661 0.20765851341418  0.38789478345460
      C     -1.61295173483354 0.93893060032542  -0.32068075967468
      H     -1.99469687736068 1.91357668026622  -0.64873747791969
      N     -1.04050825245064 0.20899743522478  -1.44577349170875
      C     -0.01946591602735 -0.60527112916161 -0.94032337196793
      C     0.64111706427562  -1.66541057150197 -1.55771935539195
      C     1.69365349317557  -2.27629963160363 -0.88296524394941
      C     2.08801980863261  -1.84125835139889 0.38692451293713
      O     3.14241296774080  -2.52211254974632 0.94561175835699
      C     3.60383415764069  -2.07509305764197 2.21538087688287
      C     1.41976179080073  -0.77848876871430 1.00564510564838
      C     0.37024836372449  -0.17627095521189 0.32706984993750
      C     -0.47434961391163 1.01120789724596  0.73776660432025
      C     0.33527452934321  2.32947404878910  0.69818656531035
      C     0.93533148735241  2.62732088009908  -0.64295042773639
      C     2.20427168485173  2.44680489788904  -1.02936728160033
      C     2.62433301512067  2.73937611876136  -2.44375478754193
      C     3.30949741985988  1.92955334471516  -0.15300908386230
      C     -1.19269552939081 0.81108856961756  2.08054140624618
      C     -2.32001232740213 -0.16601495696958 1.75903678959969
      O     -4.00532183143397 0.18528214090878  -1.44611170530109
      H     -5.26080620366920 -0.32418969700744 1.33463168438974
      H     -4.40797507402839 -1.82616502346116 0.95779451681243
      H     -5.65804045766617 -1.20914671556855 -0.15835971332589
      H     -1.72255202522807 -0.19852862510222 -2.07270236969568
      H     0.35066256009877  -2.01188530632761 -2.54559357772753
      H     2.22902205737250  -3.10638511293433 -1.33437804839713
      H     4.44779302075412  -2.71789084453744 2.47227181556440
      H     2.82529531428274  -2.17449457762263 2.98463757173790
      H     3.94185265910726  -1.03002168661045 2.17347360322347
      H     1.70946073680086  -0.41907128230818 1.98796061986520
      H     -0.34584365929449 3.14384731880103  0.98384200237700
      H     1.10275972827204  2.26930899100827  1.47845738446434
      H     0.23221703066941  2.98829684748831  -1.39415628614037
      H     3.02595624166872  1.83390310770005  -2.91793339342532
      H     3.42666910468700  3.48855209636751  -2.46819559747649
      H     1.78965106376887  3.10295263462667  -3.04913440842668
      H     3.65168934019548  0.95244838328158  -0.51976944467952
      H     3.01333080265836  1.80461849118065  0.88984278013912
      H     4.17436640822966  2.60439844542294  -0.19259691028666
      H     -1.60857694347974 1.76748298711272  2.42159042080551
      H     -0.51990082129558 0.43268132902534  2.85599307267963
      H     -3.16996142660988 -0.06257333568796 2.44118332599808
      H     -1.96370326544690 -1.20647754847050 1.79947161271813

Coordinates of (S,S)-14, Conformer ID# 7
charge: 0, multiplicity: 1
46

      C     -4.74921418157181 -0.41179359605581 0.26787702039378
      C     -3.54102824604967 0.17110865059537  -0.43090330944985
      N     -2.42468719508425 0.34486087841992  0.31928191263964
      C     -1.18129644729103 0.85222944234025  -0.30380827237969
      H     -1.35700382491249 1.85909292110223  -0.70125285643984
      N     -0.64430443354083 -0.01755471673922 -1.34324168666520
      C     0.19569795891755  -0.95185162484847 -0.72865914094692
```

C	0.72160038691264	-2.12934034968383	-1.24050802929196
C	1.61644407051911	-2.86992435706428	-0.45676341248939
C	1.97653777801043	-2.42798938015077	0.81716620102730
O	2.84313698275669	-3.08685774005329	1.65410931624549
C	3.41436211195883	-4.30096231020584	1.17732729665615
C	1.43941144930989	-1.23671242130420	1.32886456781677
C	0.55386101244360	-0.51736469363335	0.55403648970358
C	-0.14548992658610	0.79196366210920	0.85726189442444
C	0.83167711825981	1.98816566806029	0.87380411254444
C	1.63893023373290	2.11978008408941	-0.38110194597951
C	1.54183590381261	3.07050040681293	-1.31823625196399
C	2.40863644646785	3.02658743303684	-2.54686521807187
C	0.58596824969071	4.23001237513388	-1.28252295311494
C	-1.00277180274594	0.73285742750762	2.13234678906295
C	-2.23749415990683	-0.06105165554635	1.71768427520072
O	-3.56040385400581	0.47873040934504	-1.62777828990541
H	-5.11595806353244	0.26924062427766	1.04402220210020
H	-4.50791131549083	-1.36464481601487	0.75129285092089
H	-5.53504159664272	-0.56619822195566	-0.47168126734281
H	-1.32250078139223	-0.33772107002129	-2.02220192251441
H	0.45217453268206	-2.48178168013931	-2.23216445342215
H	2.01980276458957	-3.79119733780777	-0.86096300350397
H	2.64150731433015	-5.05444872435454	0.97225520678394
H	4.06601008918345	-4.65972337774338	1.97608578373598
H	4.00966978038396	-4.13440600602336	0.26911713519797
H	1.72948036249962	-0.91015356246144	2.32460499655022
H	0.25423163245817	2.89573609422369	1.07970564498315
H	1.50669025655801	1.83984490128807	1.72979572794628
H	2.36048745981001	1.31940926435472	-0.54896346941863
H	1.78915775262166	2.96325516234143	-3.45135695756032
H	3.08771972028896	2.16990109090817	-2.53433355924641
H	3.00518086820137	3.94379657534138	-2.63885770987421
H	1.13288823422980	5.17809926801407	-1.36618873586064
H	-0.02418524116905	4.26770784785130	-0.37829803515473
H	-0.09081040734357	4.18432159219203	-2.14604922310436
H	-1.29223083242886	1.74921217536742	2.42748684080519
H	-0.46761367475642	0.27085616007356	2.96706283939475
H	-3.11517591056639	0.18394671388510	2.32395689225516
H	-2.05404857561215	-1.14386918686453	1.79074370731221

Coordinates of (S,S)-14, Conformer ID# 11
charge: 0, multiplicity: 1
46

C	-4.75197404825410	-0.36498448303210	0.37768248463094
C	-3.55835333216759	0.22658339795915	-0.33859326485889
N	-2.40397993976513	0.31763986922584	0.36780718095340
C	-1.17426102440051	0.82765638168968	-0.27958310219044
H	-1.33455157426478	1.86302274821128	-0.60368234811302
N	-0.71356251657804	0.01027956902453	-1.39625087590601
C	0.12518116792882	-0.98216214215250	-0.87701235497167
C	0.59657472084099	-2.14038756672934	-1.49262036741356
C	1.50029841973650	-2.94094683297537	-0.80118619685769
C	1.93768818780981	-2.59616621674246	0.48237722703442
O	2.83020823850770	-3.46736945320209	1.05726483670008
C	3.31116910324415	-3.14279283275315	2.35683484682880
C	1.46220980883606	-1.43226840301196	1.09876884991538
C	0.55294778675964	-0.64460102465377	0.40598211283700
C	-0.08967566096119	0.66158217431798	0.82576594236025
C	0.92418412445227	1.82605290271975	0.87353519134145
C	1.67714439652217	2.01681712217080	-0.40704535168357
C	1.58715515170279	3.04231161597875	-1.26265465145070

C	2.39288731716812	3.05279209382720	-2.53293503622149
C	0.69680494181028	4.24111098348752	-1.09101026343435
C	-0.89082976916316	0.54463129753717	2.13317008108963
C	-2.16774421156570	-0.18410063828980	1.72688583465428
O	-3.62151377217067	0.61263892170995	-1.51076018671281
H	-4.52017328418024	-1.35433029855385	0.78672121433301
H	-5.57492494010368	-0.44718063510667	-0.33247994813962
H	-5.06103526500416	0.27396717867521	1.21253164604067
H	-1.43633488808835	-0.25295502270848	-2.05364818103260
H	0.27207857272634	-2.41956417069012	-2.49113362143801
H	1.88335203872615	-3.85259853686305	-1.25005523688169
H	4.00503091516821	-3.94036269252068	2.62846862022686
H	2.49379532915465	-3.10746843632418	3.09060339111353
H	3.84334312283947	-2.18137752507273	2.35915202591846
H	1.78467744920491	-1.14285698526913	2.09390393513317
H	0.38570449242241	2.73434738773003	1.16432479241394
H	1.63227338740735	1.60326554894927	1.68592639108188
H	2.34702206037285	1.19786549699802	-0.67189110804545
H	1.73013709381884	3.09463323997278	-3.40745852750565
H	3.02332074999137	2.16400049050976	-2.62133556451634
H	3.03446444356973	3.94247382268925	-2.58269960027248
H	0.13013103268028	4.23724899807850	-0.15817425208369
H	-0.01889186148967	4.29779982075144	-1.92187406810561
H	1.28914515134210	5.16456647917930	-1.12770024589244
H	-1.13376805638666	1.54728996153854	2.50676438565131
H	-0.33425888418814	0.01327690794193	2.91092366362638
H	-3.00913192351236	0.04679670161076	2.38777029660046
H	-2.01649425249984	-1.27431721583296	1.72176940324246

Coordinates of (S,S)-14, Conformer ID# 12
charge: 0, multiplicity: 1
46

C	-4.27826319806205	0.14749485559560	1.42236867746530
C	-3.40772695490727	0.30219136164027	0.19527741020438
N	-2.08113370288324	0.48356839823151	0.40671212805418
C	-1.15296423021893	0.60693869657898	-0.74060577892452
H	-1.41163721876567	1.50061280557018	-1.31875766391030
N	-1.11131632211166	-0.56544256144222	-1.60646266890122
C	-0.21583292349521	-1.47623785289849	-1.02870788086626
C	-0.04937352828024	-2.82884071892356	-1.28407934539020
C	0.97690529928465	-3.52143095992830	-0.62734449952036
C	1.81923124764703	-2.85931905036888	0.26699644552405
O	2.84672786346057	-3.45546842874944	0.95519839928088
C	3.06542235884816	-4.84424345164773	0.72799523721436
C	1.64311477312668	-1.49040531951393	0.52224389594882
C	0.62589465860413	-0.81822438896195	-0.12219291484365
C	0.24257697297918	0.64670770723358	-0.05683089998026
C	1.28895985798508	1.50016905829520	-0.81655525897886
C	0.92803909307256	2.94859975976077	-0.96669982759336
C	1.36368058254543	3.97621380875305	-0.22504328615211
C	0.90862547066395	5.38190550764333	-0.50613762234837
C	2.31766526454010	3.85267591025858	0.92959434690679
C	-0.04761866443826	1.13570742254728	1.36615864264494
C	-1.39627577766554	0.50507061471941	1.70593460335497
O	-3.87453002861922	0.26719907213912	-0.94896699533330
H	-4.30470464614137	1.08027739136281	1.99702020748935
H	-3.89971348459599	-0.63816575015350	2.08486992821800
H	-5.28969422872754	-0.10043314625457	1.10002624176850
H	-2.02086973799060	-0.90037068544088	-1.89817652883586
H	-0.69761404549795	-3.35471094097115	-1.97936895190001
H	1.09863969734209	-4.57971490782095	-0.82702188169731

H	3.91489080064947	-5.12049988216148	1.35520549165117
H	3.30946168350134	-5.04447634485862	-0.32425160557045
H	2.18961840661247	-5.44073782368154	1.01803843467282
H	2.30639291594064	-0.99392229550104	1.22637653477023
H	2.24688371438718	1.37455049291782	-0.30030899549652
H	1.41360257492579	1.05298480703108	-1.81155145875801
H	0.23083476278565	3.17808478671213	-1.77317522912613
H	1.76622756482247	6.02920100695805	-0.73182417585442
H	0.41504701628144	5.81398297508928	0.37435453815903
H	0.21274409539578	5.42389478869011	-1.34833766969148
H	3.20239053630924	4.48030754667931	0.76197395206091
H	2.65120551666678	2.82915654992235	1.10978068521681
H	1.84554254227171	4.22165132351241	1.84985307995171
H	-0.13079868004329	2.22878484022527	1.36300321477484
H	0.73815506330163	0.84764355710245	2.07105886597602
H	-1.95714703570373	1.09136996804235	2.44100736647658
H	-1.26865592580343	-0.51567050393406	2.09678281188831

Coordinates of (S,S)-14, Conformer ID# 13
charge: 0, multiplicity: 1
46

C	-4.36117621160326	0.13622565731515	0.38542515046614
C	-3.13896761910977	0.45435122970689	-0.44689702388394
N	-1.95571643898655	0.52861065271069	0.20926106529200
C	-0.70570905749070	0.80677635471931	-0.53440422949075
H	-0.76546515949479	1.80340339836930	-0.98474183948509
N	-0.36956662866213	-0.19564399868188	-1.53808924073896
C	0.29295584513514	-1.23843176151504	-0.87399676624421
C	0.54765799818501	-2.53244996835838	-1.30147778172322
C	1.30121753404083	-3.38178117189245	-0.47981784095821
C	1.79062056348738	-2.93030852089731	0.74680689207696
O	2.53303102067558	-3.69055419635853	1.61587490067867
C	2.82768742604764	-5.02819709956830	1.22590953139389
C	1.52895278184329	-1.61876633007441	1.17300231930956
C	0.77921625587568	-0.79236828611384	0.36254241331798
C	0.38836158007037	0.65905260294438	0.55595936727230
C	1.61840737435896	1.56989116043997	0.31696482597191
C	1.32530627603711	3.04119158308575	0.34507591106742
C	1.13925939922392	3.84565321831351	-0.71088802207432
C	0.85379185406929	5.31063804260617	-0.52583977521951
C	1.19321098246709	3.40050594099583	-2.14548305218534
C	-0.35458264705786	0.92108856230029	1.87125402264119
C	-1.73183358298834	0.30057608817264	1.64297690708987
O	-3.20659207691144	0.64024803681664	-1.66749948770809
H	-4.21035952966643	-0.76314789122763	0.99182945835479
H	-5.20807607578417	-0.01110006431848	-0.28499899709492
H	-4.58689702568583	0.96243605335716	1.06926240243974
H	-1.12996319083578	-0.42842703258966	-2.16443676947104
H	0.17297507558262	-2.89496593575386	-2.25462557676990
H	1.49127587054175	-4.39444051697260	-0.81619040101135
H	1.91077969046316	-5.61954001804604	1.09708508628733
H	3.42094842906678	-5.45321237175306	2.03753316381204
H	3.41143299399249	-5.05495758582032	0.29562082126659
H	1.91604462922218	-1.28728802519999	2.13341603893263
H	2.35274702576940	1.32745313644211	1.09682848394138
H	2.07141215805784	1.27068421433188	-0.63425196032325
H	1.25833813319246	3.49527965773088	1.33420900756114
H	-0.11129012378347	5.57677481663750	-0.97654094312628
H	1.61318682704465	5.92044711091163	-1.03261992462133
H	0.83238473475387	5.59219456372738	0.53055996813912
H	0.24860730671599	3.64186241619498	-2.65057272777405

```
      H     1.37179326328296  2.33028819004918  -2.26490364763224
      H     1.97979121230579  3.94682226708649  -2.68228792638730
      H     -0.45541640860424 2.00057023500549  2.03109834456630
      H     0.16246002036915  0.48806969544070  2.73283650772834
      H     -2.50952692676120 0.77781807131940  2.24803411814187
      H     -1.72284555845244 -0.77596218158949 1.87058722617413

Coordinates of (S,S)-14, Conformer ID# 17
charge: 0, multiplicity: 1
46

      C     -4.95072100533885 0.31205933626674  0.37790792484500
      C     -3.66157859131308 0.67273994298082  -0.32809593418715
      N     -2.50629183300517 0.42737512515446  0.34573818887405
      C     -1.21191371063095 0.63238913056836  -0.30927547362347
      H     -1.19405279939521 1.62681666870904  -0.76626936750689
      N     -0.92701926335986 -0.40189181103218 -1.31025900066872
      C     0.15269624571618  -1.17734252081189 -0.87145697324845
      C     0.76099566836552  -2.25120248266107 -1.52131288244900
      C     1.79232972930844  -2.92392671752281 -0.87590761074967
      C     2.20678253601879  -2.54939338236157 0.40768730164784
      O     3.22348795336635  -3.30054411465160 0.94296335795620
      C     3.68260840647334  -2.94277625908301 2.24195989202023
      C     1.59648132033957  -1.47012285676383 1.05613536023141
      C     0.57856631176317  -0.79264673340528 0.39632636666419
      C     -0.16365955706363 0.44722816626823  0.83759321557386
      C     0.78506202479661  1.66250451054805  0.96518977432249
      C     1.57804441754089  1.91671439033052  -0.28060419227524
      C     1.43191316803963  2.92477351048734  -1.15094314467802
      C     2.29624748198871  3.00865067403957  -2.37976486756758
      C     0.43333961091822  4.04163969507019  -1.02525663867842
      C     -1.00579147864631 0.24286329111637  2.11675594390695
      C     -2.34597922124591 -0.28909223692922 1.61232718638794
      O     -3.65099011015336 1.17762445143609  -1.45268398498204
      H     -5.78362997068709 0.54742991297083  -0.28479037951171
      H     -5.06165611740865 0.87629498107954  1.31033194420189
      H     -4.97556848378794 -0.75390964961899 0.62948926454871
      H     -0.93369033731449 -0.09984781937250 -2.27372131390219
      H     0.43964558320651  -2.56381712093985 -2.51092928284382
      H     2.28868939626218  -3.76226654621115 -1.35561397969365
      H     4.08758743661939  -1.92139781621425 2.25719985998982
      H     4.47761124671340  -3.65000033783443 2.48508384343305
      H     2.88045613751776  -3.02692608456712 2.98830031837919
      H     1.91401235053637  -1.14477585986230 2.04183988599913
      H     0.18429070582936  2.53370549801226  1.24791894711993
      H     1.46848065916920  1.46275124528183  1.80386641519805
      H     2.33390903297460  1.16378591900347  -0.50675813711721
      H     3.00134643008078  2.17515286806682  -2.43881403979475
      H     2.86505219796491  3.94768962716534  -2.39262559898352
      H     1.67847231838092  3.00599576434887  -3.28749329907136
      H     -0.17840718242828 3.98432393790074  -0.12321412093026
      H     -0.24118138207649 4.04405282608877  -1.89168993021442
      H     0.94689165771512  5.01182005707240  -1.02956813870909
      H     -1.15848963342912 1.20754184661473  2.61593631662931
      H     -0.52065668735386 -0.43805046869587 2.82244598006566
      H     -3.16947506289877 -0.07687414735978 2.29913977364860
      H     -2.30359760006888 -1.37723841068266 1.44437122974316

Coordinates of (S,S)-14, Conformer ID# 21
charge: 0, multiplicity: 1
46
```

C	-4.39578814794920	0.08627182515225	0.78491640195544
C	-3.26520172982989	0.41231833839383	-0.16542414154895
N	-2.01605970928281	0.44467322479376	0.35917857275900
C	-0.84968454529778	0.72822349975073	-0.50757361166047
H	-0.94224369354558	1.73812308544672	-0.92114271672935
N	-0.64140342708234	-0.24970003820528	-1.56916624914854
C	0.07923542349435	-1.31473794149741	-1.00775689796790
C	0.27289294363575	-2.60177289341198	-1.50356753683777
C	1.10132461040723	-3.46858100899991	-0.79726377278092
C	1.73535588965000	-3.06273230345453	0.38215129774762
O	2.52566637889777	-4.01006269614600	0.98452381354556
C	3.20339621497615	-3.63059417458965	2.17720101600995
C	1.53940896777050	-1.76770768900427	0.87824502998148
C	0.70171220384993	-0.91365024559402	0.17396858323376
C	0.35409086973163	0.53484789685579	0.45281309201699
C	1.56299092078767	1.44140722390708	0.11014525633425
C	1.29377268201214	2.91400122431821	0.21157014584846
C	1.00675812065235	3.75077740002236	-0.79561097218434
C	0.76123386982809	5.21242740431455	-0.54020092442497
C	0.90268298749990	3.34728989511465	-2.23967363058729
C	-0.23920958872762	0.76404590300090	1.84823459224855
C	-1.64196467061582	0.16686305686534	1.75191512206112
O	-3.46131461901247	0.64056421196095	-1.36462015692948
H	-4.53074306646646	0.89363461674213	1.51369913008784
H	-4.19727818800586	-0.83424689130064	1.34395312270267
H	-5.31276406067509	-0.02644234597380	0.20628982251707
H	-1.47236613839806	-0.46144883158446	-2.10727939530203
H	-0.20907639575582	-2.93135875061263	-2.41973474199713
H	1.26901390543662	-4.48118315533692	-1.15184471891942
H	3.77791503323400	-4.50521098804067	2.48750624787268
H	2.49543684111613	-3.36058292647272	2.97313364658003
H	3.88840379872151	-2.79004806140658	1.99926375146060
H	2.01757478118839	-1.42992994159687	1.79205145223056
H	2.37399828796994	1.16895365193993	0.79904718258925
H	1.90743261444633	1.16671631422078	-0.89250996407923
H	1.33781930253242	3.33955818162396	1.21459174004136
H	1.47171066044493	5.82914474410358	-1.10608059094001
H	0.85383502413109	5.46289918203470	0.52016896870323
H	-0.24188981060324	5.50162619506103	-0.87983450219998
H	-0.08765244088175	3.61076768775556	-2.63390236338643
H	1.05545308276970	2.27963795210178	-2.40777899371196
H	1.63350411917960	3.90275769562393	-2.84182988344089
H	-0.30633795990036	1.83894041077548	2.05108716903841
H	0.36115885373704	0.29868534847692	2.63594577488179
H	-2.34250545739216	0.63249069519070	2.45264970002754
H	-1.62413473867888	-0.91671598231924	1.94280513230184

Coordinates of (S,S)-14, Conformer ID# 22
charge: 0, multiplicity: 1
46

C	-4.26551230432105	0.19831343960597	1.50098467883474
C	-3.42440882241907	0.36300878899135	0.25483449619409
N	-2.08478984099986	0.47207975953095	0.43212510829656
C	-1.18578793464746	0.60059520167706	-0.73736825675774
H	-1.42036996688775	1.52754404543409	-1.27180002264720
N	-1.22361053615590	-0.53730559525645	-1.64901353123097
C	-0.34773598699287	-1.50453261149840	-1.13501783264006
C	-0.24595341394350	-2.85609644482484	-1.45607665321182
C	0.76649649350030	-3.60549892618715	-0.86468091045329
C	1.67023190375349	-3.02162852156847	0.02969870811914
O	2.62176793739419	-3.86681508155341	0.54505683473980

```
      C     3.56759391379860  -3.30817136527233 1.44999119493793
      C     1.56500308054008  -1.66263609200685 0.35139212878643
      C     0.54531150259643  -0.92530137408029 -0.23465812406247
      C     0.22985906649803  0.55063474335675  -0.09605291884168
      C     1.29009049978292  1.39134758778665  -0.85134386616726
      C     0.98952662394912  2.85909611183750  -0.93000347390512
      C     1.49024865256322  3.83431662315757  -0.15932640588930
      C     1.09068277438304  5.26913213059499  -0.36822643435268
      C     2.46844697904456  3.61997571645004  0.96131627750761
      C     0.00288716505655  0.99075871946834  1.35454052298756
      C     -1.36254891038357 0.40623598645653  1.70900860814047
      O     -3.92506102286723 0.39845841079450  -0.87483139617535
      H     -5.29695725460418 0.01374038576961  1.20032524059800
      H     -4.22879492951787 1.10557294651933  2.11457736232159
      H     -3.90747796310638 -0.63278897569121 2.11790364345730
      H     -2.15684140142298 -0.82476529816027 -1.91598431077395
      H     -0.93741603491822 -3.32378906473686 -2.15134217530805
      H     0.87099642195365  -4.66300667776095 -1.08820039442293
      H     4.23168341863527  -4.12657712125222 1.73374317848150
      H     3.07679345329478  -2.91068991167907 2.34933334111856
      H     4.15569907615119  -2.51106930961159 0.97433763636931
      H     2.25098353949519  -1.18748047413500 1.04532661424233
      H     2.25683692657992  1.20362898151241  -0.37123749482089
      H     1.36557205549156  0.98225651219085  -1.86743484619328
      H     0.28131841323146  3.15270512875441  -1.70544895188581
      H     0.37527661836978  5.37721674764552  -1.18784808480226
      H     1.96980951998291  5.88794820357949  -0.59095923466009
      H     0.64022924331138  5.68387914660640  0.54317689468613
      H     3.37649931234308  4.21347406702184  0.79398677615720
      H     2.75872060570142  2.57563552681956  1.08986801433201
      H     2.03885636089356  3.97090833667160  1.90905701437299
      H     -0.03026115328033 2.08557675252279  1.40027625726094
      H     0.79448329660450  0.63813215782058  2.02295164894060
      H     -1.87445130771925 0.98417959702507  2.48530072520626
      H     -1.27034607071276 -0.63536891032639 2.05199241311312

Coordinates of (S,S)-14, Conformer ID# 23
charge: 0, multiplicity: 1
46

      C     -4.55251818785101 -0.29689103034064 1.52614142357538
      C     -3.79566096919818 -0.05783261102653 0.23871393121037
      N     -2.49901703610292 0.32117472525778  0.35408212336024
      C     -1.67300500125665 0.53906831673322  -0.85548599369604
      H     -2.08643086246781 1.37308200637501  -1.43582947538622
      N     -1.51692960627244 -0.64076471896363 -1.69753026355948
      C     -0.46443176887245 -1.39225444086974 -1.15333657061762
      C     -0.13061988225211 -2.72458127195876 -1.38353219628411
      C     1.00244644335904  -3.24290613371142 -0.76388605656317
      C     1.79258484182547  -2.44993212056613 0.07488513933798
      O     2.87980094013190  -3.07697766478263 0.63223046271932
      C     3.71867073030457  -2.29568638510418 1.47585565972583
      C     1.45378032425917  -1.11105087831585 0.30566454985176
      C     0.32464420932780  -0.59914110192874 -0.32024060864932
      C     -0.25730206754699 0.80072154275162  -0.26947516170571
      C     0.54451267890049  1.80308852336572  -1.13184963885643
      C     1.95055691758689  2.00610961074446  -0.65384083556600
      C     2.44074888582662  3.04528327959731  0.03494396947374
      C     3.88586123434932  3.07675303948402  0.45367413962704
      C     1.64937942360201  4.24992423301713  0.46092925344409
      C     -0.52719946954979 1.29835362307845  1.15569542518026
      C     -1.74180373285207 0.48764636964392  1.60217331851723
```

```
      O     -4.32478434507219 -0.19723793137124 -0.86964138075562
      H     -5.53312327235172 -0.70493438523389 1.28061573538345
      H     -4.68396156038377 0.64194064436838  2.07605563977105
      H     -4.01773875002785 -0.99225804283530 2.18176760802673
      H     -2.38490102067784 -1.11620774139543 -1.91092677513923
      H     -0.73853537292497 -3.35434721112037 -2.02702371011687
      H     1.28858276298185  -4.27925801173138 -0.91684053052753
      H     4.52220737129326  -2.95993296719282 1.79908421208575
      H     3.17373322771690  -1.92942633600695 2.35701658399071
      H     4.14831532655959  -1.44129803593733 0.93418632489230
      H     2.04521972508221  -0.47846814415776 0.95853259832667
      H     0.56375656385033  1.41523250676601  -2.15954301583092
      H     -0.01282942873280 2.74646870913923  -1.15537025372867
      H     2.63926709522661  1.19540094522731  -0.89169896337384
      H     4.42052831783487  2.17668384148280  0.13757225668295
      H     3.97243260013290  3.16768395235464  1.54470640498552
      H     4.39620943974619  3.95027328157561  0.02717250837117
      H     1.60697973336697  4.30703115526037  1.55688140680333
      H     0.62528780880168  4.25339306081154  0.08366053859414
      H     2.14409618000477  5.16891326741670  0.12139557978888
      H     -0.77469730771194 2.36694235807517  1.13030453018718
      H     0.33469822885320  1.16398704437587  1.81500213944603
      H     -2.33491001367893 1.00820190672458  2.36100268586995
      H     -1.43623135514019 -0.48910077907614 2.00640528112774

Coordinates of (S,S)-14, Conformer ID# 25
charge: 0, multiplicity: 1
46

      C     -4.55312428452506 -0.27717443018786 1.46824377449112
      C     -3.77276036808481 -0.10175515544573 0.18461691870720
      N     -2.49781872059268 0.34390463695686  0.30160269349826
      C     -1.64832443265216 0.50581390667274  -0.90070761071110
      H     -2.07824920264341 1.27685859499891  -1.55180126859706
      N     -1.42187308375818 -0.72429350388777 -1.64848332890061
      C     -0.36074956801078 -1.39526740862522 -1.02343426975048
      C     0.02532598845717  -2.72174385791552 -1.13766709400780
      C     1.16134139830164  -3.15942842740068 -0.44363736723086
      C     1.88843060502333  -2.27370232163447 0.35245207733983
      O     3.00487654276064  -2.61413829511409 1.07566324922738
      C     3.44458115750148  -3.96647123611854 0.99943743702275
      C     1.49088682161515  -0.93218628806890 0.46413827432047
      C     0.37592243740815  -0.50595964854033 -0.22832823652450
      C     -0.26122778394858 0.86879580771451  -0.30169533879474
      C     0.52425038231767  1.83350027817939  -1.21970298846712
      C     1.89877647440375  2.14831857752368  -0.71268468750079
      C     2.31597509714963  3.27637520838034  -0.12282333282675
      C     3.73957128315254  3.41874409140772  0.34359523555918
      C     1.45882924365015  4.48287867567729  0.13928442721105
      C     -0.58914874407059 1.46243713458702  1.07332508058764
      C     -1.78331383935245 0.63920174511036  1.55138788214830
      O     -4.26503025396537 -0.34909503799747 -0.92207012811381
      H     -5.51010935187655 -0.74217062536852 1.23090411825626
      H     -4.73535855708971 0.69270459427568  1.94483490189412
      H     -4.00836533805300 -0.90053479171010 2.18528502549463
      H     -2.26407506910942 -1.24618470037008 -1.85594804118186
      H     -0.53969364927853 -3.42179349330129 -1.74667558182801
      H     1.45486067322730  -4.19905111718341 -0.53177238198199
      H     4.33139678945366  -4.02953962889870 1.63262678380737
      H     3.71162311207529  -4.24366529511606 -0.02952887055969
      H     2.67892995857962  -4.65892949642054 1.37539153985311
      H     2.06398401138224  -0.26135458425609 1.09832602297785
```

H	0.60092396138754	1.36218634210141	-2.20929648985975
H	-0.07689099043788	2.74144452953562	-1.34298776424724
H	2.62914637068522	1.34841406674043	-0.83404663188557
H	4.32367534523621	2.51511303601755	0.14901841355763
H	3.77596393232850	3.63075641551594	1.42044711681136
H	4.23029451287305	4.26379633442983	-0.15703603670977
H	1.91921838286317	5.37821536226476	-0.29793518696747
H	1.38622575698996	4.66738778955149	1.21940876479237
H	0.44571824004705	4.39177255191676	-0.25606506668761
H	-0.87650294541662	2.51510156830806	0.95849306617671
H	0.25896149926165	1.41348841032482	1.76146442473733
H	-2.41805089780964	1.19216768255290	2.25130765552764
H	-1.45090289745636	-0.28914799718269	2.03961281933501

Coordinates of (S,S)-14, Conformer ID# 27
charge: 0, multiplicity: 1
46

C	-4.71555406245208	1.59484541478703	0.14487137638086
C	-3.45455532604261	1.14014437445869	-0.55434008424499
N	-2.44013880314386	0.71661262197541	0.24397553243888
C	-1.16025601326154	0.33546649214460	-0.33532078687951
H	-0.86346582212406	1.08188731750004	-1.08312952322217
N	-1.13671430289417	-1.02025238273790	-0.91317536686934
C	0.08724918801487	-1.60638093646593	-0.55235406577369
C	0.68315917127128	-2.75256760484359	-1.05701478203547
C	1.89382395483122	-3.19013381290045	-0.50308818429951
C	2.48632718617802	-2.48845125917824	0.54756162108697
O	3.66924466383217	-2.83993531943824	1.14911228110451
C	4.32695919448670	-4.00623512256941	0.66452074281824
C	1.87693523403108	-1.33134761201572	1.05690145621479
C	0.69230994223051	-0.90053943967190	0.49798087446484
C	-0.18699961771978	0.26461332146694	0.86653717794170
C	0.54887049038176	1.59526454314486	1.12311482582360
C	1.37812001013615	2.06157508842419	-0.03574871492410
C	1.06382353474178	3.01507328300405	-0.92295502746071
C	1.99488799113503	3.35409464036103	-2.05440399440246
C	-0.21122227359769	3.81154419888613	-0.91109635596763
C	-1.14506321976998	-0.07523236693046	2.03901270893942
C	-2.43820485217266	0.67609617667850	1.71359252749537
O	-3.34456063039155	1.13999136772365	-1.78305773820655
H	-5.20312664196765	0.74770226588039	0.64066807322047
H	-5.39537704886135	2.01062750421416	-0.59887611379608
H	-4.50258502659253	2.34699970450860	0.91200894085269
H	-1.38470078143529	-1.05821279866130	-1.89309451716463
H	0.23083402928080	-3.30893446696687	-1.87325955902443
H	2.35867812514721	-4.08036220808839	-0.91065346793941
H	3.70039703184895	-4.90020015619326	0.78797443766773
H	5.23128446608062	-4.11241081051782	1.26640298451891
H	4.60427732524980	-3.90000644894004	-0.39323564531761
H	2.35860731510753	-0.79330290810592	1.86942697972027
H	-0.18993407252768	2.35079142865183	1.41397220204001
H	1.19552182100976	1.44646073464964	1.99888033303943
H	2.32118383095539	1.53270939441890	-0.17548797129795
H	1.49927475011993	3.20017563926983	-3.02191401782499
H	2.90279023148261	2.74549293031371	-2.02977247712354
H	2.28395553267653	4.41257248775514	-2.01640596102691
H	-0.77326638311010	3.63550945012937	-1.83812332326002
H	0.00867574829654	4.88639690533979	-0.87849015769629
H	-0.86952719101169	3.56971098673257	-0.07405611095037
H	-0.72485822829995	0.21211261424797	3.00763674150233
H	-1.33281767074175	-1.15399834063264	2.05090020281806

```
      H     -2.43282999737369 1.69382394652513  2.13125908002418
      H     -3.32191280303457 0.15709916166596  2.10079284659511

Coordinates of (S,S)-14, Conformer ID# 28
charge: 0, multiplicity: 1
46

      C     -4.67871221353003 1.60840503237859  0.26326807275402
      C     -3.44226935138777 1.17165125862830  -0.48924386607897
      N     -2.42281525170239 0.67558700071462  0.25960053730729
      C     -1.16521182844465 0.29968269426451  -0.36971009064728
      H     -0.86367908911933 1.08413228100903  -1.07559171991483
      N     -1.18820622247042 -1.01669939749623 -1.03336426897740
      C     0.02834475924126  -1.65140323715878 -0.73124103977496
      C     0.58247106680007  -2.78613331945512 -1.31981313277269
      C     1.78178717032860  -3.28050120308364 -0.81649720173052
      C     2.41968684261606  -2.66505859344594 0.26570804099345
      O     3.59708110091754  -3.24653224555682 0.66657310783174
      C     4.28158835746030  -2.64470992530065 1.75990952428115
      C     1.85852221383214  -1.52842976836690 0.86091390136655
      C     0.67030787085859  -1.03250753999678 0.34241667112437
      C     -0.17027702959954 0.12822938551562  0.80436897860889
      C     0.60331369092131  1.42237544672051  1.12843980281402
      C     1.42709901497699  1.93669806564668  -0.01382170757960
      C     1.12582068667904  2.95105050303880  -0.83560304642457
      C     2.04766818596824  3.33308859152912  -1.96086194342858
      C     -0.12598719173694 3.77959199335964  -0.75310203696690
      C     -1.11424993544144 -0.26131502192368 1.97327850854311
      C     -2.39370792592189 0.54067111003148  1.72325572102691
      O     -3.35620660559713 1.24677310913338  -1.71748675338344
      H     -5.18177131974546 0.74010816974550  0.70408313895261
      H     -5.35848758705270 2.09466937382067  -0.43653119276091
      H     -4.43365743818626 2.29648363093512  1.07932117616643
      H     -1.44560458310621 -0.98166339223605 -2.01118715347553
      H     0.09847150722167  -3.27854034146004 -2.15873260931268
      H     2.24618920180624  -4.15577697715319 -1.26087405713594
      H     5.17857961766846  -3.24562450675990 1.92012838108325
      H     3.66909805927198  -2.65583802668945 2.67216685729553
      H     4.57339095812290  -1.61039681991161 1.53048991356349
      H     2.33701785284719  -1.02863845528415 1.69682842460368
      H     -0.11136549622310 2.17737005187091  1.47553808106749
      H     1.25846682777982  1.20631680862920  1.98393605231676
      H     2.35205260029586  1.39134686111144  -0.20338391194133
      H     1.53260977935522  3.25062296430021  -2.92691794374898
      H     2.93905950897854  2.70064451897354  -1.98890699122491
      H     2.36571288040930  4.37952360441506  -1.86477868969323
      H     -0.70572909056119 3.67737665548934  -1.68030690500755
      H     0.12445592837831  4.84394987837516  -0.65716279153383
      H     -0.77911761604443 3.50360021666839  0.07743067606643
      H     -0.66908316662442 -0.04685134469682 2.94973653400547
      H     -1.32966081209072 -1.33368875102551 1.91999330197681
      H     -2.35397683088078 1.52903927828758  2.20483330019848
      H     -3.28296909726883 0.01969038240881  2.09486034956670

Coordinates of (S,S)-14, Conformer ID# 29
charge: 0, multiplicity: 1
46

      C     -4.98146749086189 0.26925570843529  0.23137762094050
      C     -3.67170986378118 0.61595945562348  -0.44297140516708
      N     -2.54160824703345 0.44888569812912  0.29381096183560
      C     -1.22477363034473 0.64878564352048  -0.31766062340337
```

H	-1.21349405120385	1.61159782303175	-0.83816749113229
N	-0.86701173091022	-0.43815527185698	-1.23404316156907
C	0.20756632854463	-1.15857053535440	-0.70254574840449
C	0.86919735975985	-2.25020260706244	-1.24883639951629
C	1.89115996127478	-2.86357235461639	-0.51408658742508
C	2.22741581202900	-2.39529324894888	0.75772027001655
O	3.20264083190190	-2.94084028912563	1.55511742606222
C	3.91657978414222	-4.06028209458948	1.04017743480813
C	1.55868397428683	-1.28848685524949	1.30024725776517
C	0.56444816270153	-0.67900939627308	0.56197082799697
C	-0.22479553590356	0.56923535753382	0.88247853600871
C	0.69182183790495	1.81284251829947	0.96251829200046
C	1.53042905058798	2.00026333122172	-0.26513764841899
C	1.39682629556825	2.93945038814498	-1.21105091752132
C	2.31195039142813	2.96079707708911	-2.40528255728192
C	0.36637815673604	4.03421988047198	-1.20737602547975
C	-1.11659390970958	0.43359519798418	2.13620196089937
C	-2.42004289871720	-0.16923391358222	1.61553300927186
O	-3.62438653576250	1.04230758786405	-1.59894250327971
H	-4.98987006759689	-0.77576182638005	0.56001691930444
H	-5.78937166524742	0.43127957267727	-0.48230540188878
H	-5.14930961844982	0.89636930171659	1.11379220452835
H	-0.85216972747885	-0.20874765606412	-2.21682656486860
H	0.60386442367790	-2.63392597654791	-2.22995216464784
H	2.40503022297728	-3.71311735960737	-0.94878820796460
H	4.63573291596076	-4.33831852488009	1.81272181333548
H	4.45478263050553	-3.80265705472276	0.11770335202397
H	3.24593898575201	-4.90827625134802	0.84507604312952
H	1.85185820190640	-0.92171141251395	2.28107963805506
H	0.06149342085607	2.68657514946036	1.16022298438800
H	1.34442607062515	1.68625343945191	1.83885695260037
H	2.31472798895583	1.25538877075015	-0.40394712193107
H	2.85608105116922	3.91273011506425	-2.46222281286571
H	1.73529413493193	2.87522137016701	-3.33575803176483
H	3.04054993283233	2.14625084396582	-2.37363507165697
H	-0.28472842981397	4.02128949162046	-0.33150755149200
H	-0.26737398306319	3.95915511351511	-2.10089797564374
H	0.85553063500309	5.01575290547184	-1.25586057905125
H	-1.31401555529715	1.42704401977049	2.55695023423635
H	-0.64626186378565	-0.18148323190454	2.90888926120705
H	-3.27791608284097	0.06993345396540	2.24935353143307
H	-2.34472767421754	-1.26517335431829	1.53042602052756

Coordinates of (S,S)-14, Conformer ID# 30
charge: 0, multiplicity: 1
46

C	-4.25324396563213	1.94998665065125	0.70165161725134
C	-3.40096667932724	1.07997692602082	-0.19378287112499
N	-2.13594398746983	0.83223597023848	0.23308093671149
C	-1.22553156801750	0.05509483444882	-0.59275131114838
H	-1.27859361327077	0.42167445201783	-1.62828105032661
N	-1.47227971889439	-1.39567535093124	-0.53923211685781
C	-0.23008812071343	-2.03780039135164	-0.43869253911406
C	0.06650325814214	-3.38369452944888	-0.59552603181421
C	1.38230699723324	-3.81889684010411	-0.38832537735651
C	2.37740051018685	-2.91285334038432	-0.02009662918152
O	3.69017930294242	-3.24650487801516	0.20293130313851
C	4.05126602228211	-4.61516437656771	0.04741469855482
C	2.07084700196189	-1.55234454657285	0.13935711468105
C	0.77582892114348	-1.13016365143060	-0.07768810872483
C	0.17287644415884	0.24576089357121	0.05227800175230

C	1.01669761447760	1.34287288445826	-0.62950464352329
C	0.36616773591828	2.69330135912682	-0.64622337416374
C	0.71714103450391	3.78409293062107	0.04713371537895
C	-0.07366705918081	5.05829005668136	-0.07565268008758
C	1.87523500184214	3.86746227985169	1.00146813444245
C	-0.17560178422795	0.58947031035728	1.52712870682566
C	-1.52972245248298	1.30603368159550	1.48497035857644
O	-3.82228580208253	0.61125578264928	-1.25485210252237
H	-5.19433852106934	2.15817037120864	0.19249113736205
H	-3.74801698808175	2.89099004074401	0.94510283134107
H	-4.46348003557740	1.43570402766521	1.64640660468380
H	-2.11338257471025	-1.73953652508028	-1.24153840173550
H	-0.70060392491180	-4.09922895898033	-0.87824664085613
H	1.60790596447688	-4.87043812517189	-0.52279176917764
H	3.50198540591504	-5.25704600001477	0.74980024741421
H	5.11911422663314	-4.67023745907205	0.26661106317360
H	3.87216232882450	-4.96289272706022	-0.97924068264909
H	2.86254004901957	-0.86278542326729	0.42041569455613
H	1.99000487009836	1.37645821174884	-0.12957533946043
H	1.20511972374611	1.01720869968306	-1.66211675478385
H	-0.50743450258002	2.77818858582362	-1.29542581846046
H	0.56534356873224	5.88438514438509	-0.41405650707021
H	-0.47787315660690	5.35813098400247	0.90084326259455
H	-0.90598082990233	4.95507607250539	-0.77738388198143
H	2.55535111010585	4.67613205243835	0.70416938360300
H	2.45290706000948	2.94406613263226	1.06981172137976
H	1.51682383665695	4.11957277779413	2.00854348991888
H	0.60028553621109	1.20625107384213	1.99071478937754
H	-0.26063951948228	-0.33993606763145	2.09922510242939
H	-1.40393874962125	2.39652168708965	1.46080471642415
H	-2.15244997137923	1.04570431723225	2.34895000054948

Coordinates of (S,S)-14, Conformer ID# 31
charge: 0, multiplicity: 1
46

C	-4.40709204712168	1.00926567965915	1.37469834417788
C	-3.51591606228696	0.82443848346029	0.16565689326016
N	-2.19434260969643	0.61531180849801	0.40692952361750
C	-1.28198643998448	0.31501721684309	-0.69888668478936
H	-1.43282175087970	1.05602168241602	-1.48963048951858
N	-1.47179971121164	-1.04231300807532	-1.22094810576826
C	-0.34378441825016	-1.81356974489297	-0.92332414680690
C	-0.10811182176047	-3.15220556513503	-1.23636083892636
C	1.06475977297193	-3.74647348366324	-0.78442668246276
C	1.98754349976163	-3.02855763994646	-0.01498279827861
O	3.09823448009151	-3.73045826633950	0.38321836719036
C	4.06672606795756	-3.03182719998363	1.15762361063425
C	1.74906566393291	-1.68532021981158	0.29651384218340
C	0.58327129808276	-1.09356632061591	-0.17461732824391
C	0.14202303957385	0.34622909751720	-0.04510233045821
C	1.13819127875007	1.27311638827278	-0.79154288512155
C	0.71545672823776	2.70929559760246	-0.86932017071569
C	1.19194464904745	3.74121327862457	-0.15930967674318
C	0.65836885965525	5.13245734717974	-0.36448031502119
C	2.26908777382600	3.63995925401599	0.88410081548836
C	-0.10138697222882	0.78756904881819	1.41311654308326
C	-1.54767417661196	0.39031324338663	1.70109366394901
O	-3.95605084372823	0.87452007416808	-0.98496842963815
H	-4.36101350551607	0.13561517337391	2.03408297251615
H	-5.43142931028599	1.14894336800047	1.02893347318554
H	-4.10152613438433	1.88362295746982	1.95971423257887

```
      H     -1.88254267496924 -1.11992591117249 -2.13954633656496
      H     -0.82405257434705 -3.72747457384193 -1.81668159146238
      H     1.27949740663505  -4.78609774854314 -1.01315263916917
      H     4.85839112380981  -3.75254430969190 1.37013076145270
      H     3.63992360541115  -2.67143825085899 2.10397709725819
      H     4.48813474616816  -2.18257840468988 0.60204858316085
      H     2.45625904834269  -1.10318996075231 0.87881020554520
      H     2.11463003105932  1.16756921229500  -0.30665810074615
      H     1.25537311958262  0.87370924359348  -1.80837340153681
      H     -0.07427004111461 2.92839005244910  -1.58904705889304
      H     0.24070142855055  5.52950159020619  0.57007167734224
      H     -0.12054460915233 5.16108868585449  -1.13108192239995
      H     1.46382799971769  5.81681082229655  -0.66172497235447
      H     1.89885484056011  4.02146148393233  1.84477478727903
      H     3.12580779189053  4.26942305450426  0.61039372995645
      H     2.62841551517217  2.62141814423660  1.04168732085711
      H     -0.00178184517565 1.87656566540692  1.48373293721503
      H     0.60885560513282  0.32305981643278  2.10397952780182
      H     -2.00311007646487 1.00071312774845  2.48530355953173
      H     -1.62104774875064 -0.66870999024828 1.99653443635456

Coordinates of (S,S)-14, Conformer ID# 32
charge: 0, multiplicity: 1
46

      C     -5.21509134055209 0.96413239071229  0.50208227148487
      C     -3.99468243868557 0.67580993137337  -0.34255133238887
      N     -2.82660296282065 0.48750407135874  0.32474458324184
      C     -1.59105543679427 0.25915942300229  -0.41502450948865
      H     -1.50862107449683 1.01567516679687  -1.20640178012934
      N     -1.47258081808330 -1.09466027622567 -0.97927575617335
      C     -0.16118860802598 -1.53756317816462 -0.74373716136997
      C     0.48744600220974  -2.64536049564291 -1.28552372850328
      C     1.77688306465400  -2.94005999375978 -0.85375049300911
      C     2.41096820347425  -2.15421783249039 0.11422557541488
      O     3.68080725735943  -2.54917220246318 0.45834637480566
      C     4.34872004901200  -1.79103529270579 1.46040796698142
      C     1.75657553675575  -1.04337510301968 0.65970901268360
      C     0.47800782538428  -0.74532184329289 0.20988729367323
      C     -0.46436251487987 0.34290911942872  0.65153215770984
      C     0.16713301772616  1.74489172719591  0.77825005413077
      C     0.85404841273833  2.22480190313996  -0.46424720023894
      C     2.15656030650700  2.48130068848290  -0.63907631362327
      C     2.67557492130278  2.94119402438162  -1.97444035843653
      C     3.21927229900505  2.34242336145408  0.41379931306393
      C     -1.22219432685393 -0.04170893887269 1.95192755393140
      C     -2.62442258710712 0.54234583863804  1.77826869916237
      O     -4.04593305146476 0.60079968540487  -1.57273626188591
      H     -6.03962751242216 1.23258151581993  -0.15854867812012
      H     -5.03296273153230 1.77504708393732  1.21508117404250
      H     -5.49589559686091 0.07448338867049  1.07751736976810
      H     -1.82389515784668 -1.18621868485428 -1.92322456348723
      H     0.00523464965128  -3.27034124253797 -2.03208400335132
      H     2.31401852188310  -3.79031799831046 -1.26339098603695
      H     5.32250035437467  -2.26494775450124 1.59703081875689
      H     3.79811917554217  -1.80989717142789 2.41130581928806
      H     4.49371039986933  -0.74805073252266 1.14617045841589
      H     2.23132942458568  -0.41279512088668 1.40358216510954
      H     -0.63354712567245 2.45125035218515  1.04766274381756
      H     0.85500976980635  1.72396364372812  1.62997271043054
      H     0.20538432496384  2.37685126819039  -1.32747310873075
      H     3.17614228091502  3.91425787957439  -1.88511074359909
```

```
      H     1.87541801864480  3.02966191615832  -2.71416008310975
      H     3.42533921058292  2.23706717203181  -2.35875707703205
      H     3.96661312847545  1.60364123696454  0.09537353576651
      H     2.83471024863614  2.03789745806603  1.38869818320076
      H     3.75419161878488  3.29265119489575  0.53985579077149
      H     -0.71797015425086 0.33480678849958  2.84716605450675
      H     -1.28094025104265 -1.13296177419046 2.02241442647636
      H     -2.68094898127019 1.57907369141849  2.14263865475492
      H     -3.38161535218185 -0.04476628564073 2.30897337732476
```

Coordinates of (S,S)-14, Conformer ID# 33
charge: 0, multiplicity: 1
46

```
      C     -4.42805514294215 0.98360793331768  1.30870091101305
      C     -3.50817420163162 0.78531800144709  0.12352387617528
      N     -2.18414912241135 0.64145512416083  0.39455555412364
      C     -1.23999647452479 0.33498494074848  -0.68363222361835
      H     -1.40227985837646 1.04156015838044  -1.50316534659557
      N     -1.36868558718156 -1.04473443508741 -1.15874554681201
      C     -0.22802965374446 -1.77103877575399 -0.80834764314527
      C     0.05992953405729  -3.10534682437899 -1.06264772571063
      C     1.24693141079591  -3.65251499479046 -0.56015466842923
      C     2.11883733059754  -2.87213905512647 0.20132038585417
      O     3.29500928327000  -3.32497146710831 0.74580362469682
      C     3.64015621362567  -4.68686933860629 0.51365658293435
      C     1.82309707233268  -1.52396877488348 0.45039207272060
      C     0.65966996232296  -0.98695361324384 -0.06310469848724
      C     0.16825021858940  0.44148464237242  -0.00617257756027
      C     1.14931768233504  1.36384890315041  -0.77770415138633
      C     0.68674604933037  2.78215137546662  -0.92631499604765
      C     1.11814730110743  3.85641701506410  -0.25114451049949
      C     0.55021790597559  5.22167597376677  -0.52697379605574
      C     2.17382152764573  3.83043743632548  0.81837158349082
      C     -0.11682179283339 0.93923493481069  1.42534454550972
      C     -1.55191584901750 0.49976374032170  1.70780424276837
      O     -3.92951706328603 0.76850326743022  -1.03540918557150
      H     -4.17108094754010 1.89552489640080  1.85873011997995
      H     -4.35606070527195 0.14278194020881  2.00725071629332
      H     -5.45114508560101 1.06290868220524  0.94090451042988
      H     -1.77647491984692 -1.17960608829205 -2.07144233578417
      H     -0.62120459694350 -3.72694867708412 -1.63693081986901
      H     1.46608974295376  -4.69388251699735 -0.76556902860337
      H     3.76030148726406  -4.89299965486387 -0.55876445894096
      H     2.88728449652321  -5.36841343507387 0.93274266852150
      H     4.59349748364067  -4.84519596234576 1.02101707669563
      H     2.52410924133018  -0.92805748650996 1.02985132956689
      H     2.11996248444354  1.30776433479398  -0.27380995543229
      H     1.29506277315765  0.92360286045603  -1.77377893482690
      H     -0.09222209010628 2.94744623587245  -1.67162226649216
      H     1.34256133944090  5.91614650671379  -0.83566078771523
      H     0.10039784327180  5.64524928049613  0.38061836133177
      H     -0.21166675891783 5.19542250569976  -1.31059302381297
      H     2.56272406912688  2.83131525098596  1.02275645422142
      H     1.76962795270586  4.23524509865399  1.75552829000593
      H     3.01538503350918  4.47756111416078  0.53909894377887
      H     -0.06048248223520 2.03337379528785  1.44565021653372
      H     0.59858698063528  0.53543427523630  2.14793674205096
      H     -2.04387436058043 1.12611034163713  2.45655036016560
      H     -1.59007572699604 -0.54704946542575 2.04975951253407
```

Coordinates of (S,S)-14, Conformer ID# 36

```
charge: 0, multiplicity: 1
46

C     -4.59147359449641  0.70625204766393  0.63681267733337
C     -3.37416156413374  0.78398346053610  -0.25940397835466
N     -2.16902272151765  0.55858887782382  0.32501378743892
C     -0.94801167969870  0.53384734202019  -0.48767371021570
H     -0.92712461084015  1.43078555878840  -1.11302657325315
N     -0.84433640647448  -0.67792322009640 -1.30055515376351
C     0.16983897791135  -1.49536285089868 -0.79707934967902
C     0.58744991205858  -2.73879133420271 -1.25227076620347
C     1.59116362454132  -3.41782703493365 -0.55035451433282
C     2.15109906405230  -2.86028079863129 0.60061614076603
O     3.12561489659025  -3.45851963530101 1.36045429903580
C     3.59151235874441  -4.73569066707290 0.93649170043487
C     1.72925675693488  -1.60051064998615 1.05065842498199
C     0.75103431261786  -0.92984966549396 0.34456791977668
C     0.20978619264566  0.46529855562936  0.56011177438016
C     1.33223839015526  1.50195657445339  0.28136498041142
C     0.87294659402317  2.92927144388239  0.26641785007143
C     0.62473198889052  3.68797658922052  -0.81073786638713
C     0.15314466520046  5.10828677676195  -0.66226375552819
C     0.77801219516038  3.23577540359635  -2.23625784656532
C     -0.47991336272313  0.65763294642687  1.92499028354659
C     -1.90303406712386  0.14706984374195  1.70452573228289
O     -3.46257160157769  1.05006870064765  -1.46064515112369
H     -5.47994292476968  0.86180174043114  0.02456980429971
H     -4.55417958916268  1.47458928393727  1.41700278181922
H     -4.65575833004140  -0.26831938265328 1.13277449165178
H     -0.95668232253886  -0.58009649136720 -2.29796895195313
H     0.14405856753067  -3.19148601970536 -2.13475687702086
H     1.91273015325019  -4.38739306806124 -0.91276900108251
H     4.35183339715563  -5.03302517504470 1.66097973145450
H     4.04209289705621  -4.68609581793553 -0.06429127218966
H     2.78118635807818  -5.47757042538840 0.93416142776141
H     2.19321334011932  -1.17325954611564 1.93640794053744
H     2.09623357937578  1.36770188439426  1.05941688639052
H     1.80890958882618  1.22720545004759  -0.66548457304474
H     0.71889768398951  3.39214857695764  1.24136578389290
H     0.05990080461159  5.39919182046697  0.38757160149556
H     -0.82193654393059 5.24336614405215  -1.14833960455957
H     0.84714631679345  5.80161274236150  -1.15520221225475
H     1.47861298057853  3.89227148012026  -2.76832195183527
H     -0.18264282960225 3.31555258911250  -2.76231523672665
H     1.13213808952547  2.20758563476047  -2.33343121728111
H     -0.51559306349097 1.72181448704307  2.18129297343074
H     0.04211983508024  0.12392261211046  2.72488191864568
H     -2.62127317609281 0.59042139484428  2.39928513833181
H     -1.95259513328231 -0.94908817894431 1.80381351318347
```

Table S32: Electronic energy, correction to Gibbs free energy, and respective Boltzmann weights of the conformers of ***(S,S)*-16** at the ωB97M-V/def2-QZVPP/C-PCM(Hexane)//r2SCAN-3c/C-PCM(Hexane) level of theory for the calculation of the ECD-spectrum.

Conformer ID #	Electronic energy [E_h]	Correction to Gibbs free energy [E_h]	Boltzmann weight [%]
1	-729.122382	0.256857	60.44
2	-729.121382	0.256989	18.21
3	-729.120394	0.256634	9.31
4	-729.120279	0.256472	9.8
5	-729.119391	0.257345	1.52
6	-729.116772	0.256228	0.31
7	-729.116339	0.256851	0.1
8	-729.117320	0.256779	0.31

```
Coordinates of (S,S)-16, Conformer ID# 1
charge: 0, multiplicity: 1
35

      C     -3.45928088478847 0.44400679462764  1.46612775034911
      C     -3.04408057105420 0.59612681486245  0.01490465707661
      N     -1.78328768304992 0.19376442518682  -0.31129995593438
      C     -0.80124578393469 -0.38316946654699 0.61292287866598
      H     -1.30013137057920 -1.06613172949100 1.31023003052170
      N     -0.00792358591586 0.60650223535521  1.35874863687479
      C     1.18269365184145  0.84268816698588  0.66866188444586
      C     2.11468165640924  1.85496954612841  0.88423813765320
      C     3.27796638047990  1.84884789288685  0.11356427447134
      C     3.50883009745888  0.86047934329066  -0.84265938574151
      C     2.55952661164830  -0.14597996157881 -1.04944663440870
      C     1.39994883463103  -0.14600517249237 -0.29436234414123
      C     0.23124687664085  -1.10690211141461 -0.31919633579299
      C     0.61738660392926  -2.51115622143881 0.17043107071005
      C     1.28669305467324  -2.54277701052220 1.54213390290987
      C     -0.46300894785049 -1.13814186076121 -1.69511902457757
      C     -1.32712066373745 0.11824849992391  -1.70591275368881
      O     -3.82184170236950 1.03966355261170  -0.82932199771551
      H     -2.72225653261832 0.83929125077214  2.17118738224623
      H     -3.60835206475030 -0.61569288421808 1.70434025931777
      H     -4.40491613029083 0.96866684560934  1.60309819636595
      H     -0.50773330770160 1.41600571879471  1.70147360253644
      H     1.94431292874332  2.62696523491291  1.62960062220535
      H     4.01575604019319  2.63221188841714  0.26430925627947
      H     4.42135367430281  0.87604233447262  -1.43039354316318
      H     2.73045081019399  -0.91482287680470 -1.80006003325483
      H     -0.28990532587912 -3.13101465765773 0.17915153698464
      H     1.28929321028929  -2.95183252111189 -0.57885471039791
      H     0.62709445083682  -2.14402434686813 2.32051381563991
      H     2.20429125281917  -1.94545398455473 1.54613461465885
      H     1.54831501479834  -3.56895375326259 1.81851558735583
      H     -1.10053381579863 -2.02815961523612 -1.76910564341764
      H     0.26270995520734  -1.16245413910520 -2.51344593486382
      H     -2.19193899950571 0.06172651488904  -2.37061497429671
      H     -0.74031373527213 1.00887525333771  -1.97234482587416

Coordinates of (S,S)-16, Conformer ID# 2
charge: 0, multiplicity: 1
35

      C     -3.48560793349119 0.84601227733484  1.33878586609548
```

C	-3.00209977833724	0.72709887646953	-0.09403319332383
N	-1.75696351212532	0.20595798371477	-0.28446684700837
C	-0.85828202430593	-0.28035141167896	0.77284054688485
H	-1.42177567891672	-0.87000083428706	1.50547453797303
N	-0.07746425206468	0.75566130211033	1.46072550483548
C	1.11598706312217	0.94362389597544	0.76021930903999
C	2.01487393652357	2.00099155200990	0.86369708602211
C	3.16740187143786	1.96007970857915	0.07771023961575
C	3.41166326930798	0.89819938624379	-0.79110646674543
C	2.49671220463994	-0.15668276540078	-0.88310044928151
C	1.35703613716957	-0.13632659494657	-0.09627646169496
C	0.21068222484316	-1.12450813808689	0.00327801371163
C	0.58139579812867	-2.39181380281261	0.79306822297753
C	1.62767259573391	-3.27469907387729	0.11998840972167
C	-0.44297039066619	-1.40203379300801	-1.36038022040783
C	-1.27131215169641	-0.14862649339671	-1.62685369699871
O	-3.71308684399610	1.06690705960679	-1.03941401448331
H	-3.61722130511823	-0.14714376008473	1.78331493512434
H	-4.44780975910079	1.35804425456735	1.32814963703893
H	-2.79362950889121	1.40351864225137	1.97706242693986
H	-0.57621285631262	1.58696316904138	1.74643019313851
H	1.82186764978282	2.83973501744410	1.52697154146579
H	3.87875184000223	2.77921469585595	0.13848420698763
H	4.30717417321813	0.89353196560835	-1.40468599617386
H	2.67626849905406	-0.97253866823973	-1.57853798057167
H	0.93776629476473	-2.08866045207300	1.78672970455903
H	-0.34151332704881	-2.96840418487712	0.94746189037864
H	2.58711126093275	-2.75688115269381	0.02987617639900
H	1.30888018102108	-3.58160841475515	-0.88241255439581
H	1.79472123644941	-4.18258965252050	0.70802461435115
H	-1.09954559688965	-2.27882525283154	-1.28727183100129
H	0.29591689640337	-1.58834243908765	-2.14471109719648
H	-2.12218258028791	-0.30960054593443	-2.29297137310621
H	-0.65288563328638	0.65918764377953	-2.04274088087110

Coordinates of (S,S)-16, Conformer ID# 3
charge: 0, multiplicity: 1
35

C	-3.36116597108874	0.56740559002726	1.47714838422584
C	-2.93639643075941	0.57662229409769	0.02069465761585
N	-1.66773222259875	0.16047279440713	-0.25367526970348
C	-0.68610694779811	-0.30634890739555	0.73356335375488
H	-1.17741141779174	-0.93576371054837	1.48260367427817
N	0.07274825774212	0.75966938644141	1.40463738061397
C	1.22927637026057	1.01531505091904	0.66548580735654
C	2.09854525752776	2.09792715022724	0.76962909502047
C	3.23844389335617	2.10173083407338	-0.03581469431636
C	3.50604236804626	1.05520508691074	-0.91733301857037
C	2.61992363213794	-0.02360166278553	-1.01103295283010
C	1.48375192154106	-0.03404197348902	-0.22137592553656
C	0.38082379208679	-1.06944414170219	-0.12366778100811
C	0.91482266830067	-2.34311122513925	0.55765807766476
C	-0.09549681437638	-3.47390506596242	0.75006163063781
C	-0.31513971015460	-1.29197192736024	-1.47802342298562
C	-1.20873234316782	-0.06487379974494	-1.63237256557506
O	-3.71254305473983	0.92130185789744	-0.86992556033546
H	-3.44422810574965	-0.46300765525167	1.84199454143997
H	-4.33897242605600	1.04413954273100	1.54632449097379
H	-2.65959434757752	1.09293582550798	2.13186337956164
H	-0.45737054769111	1.55989372988408	1.72194886863232
H	1.89753119893526	2.91698049723648	1.45472679484660

```
H     3.92708405521657  2.93992105322330  0.02678451383295
H     4.39840142401902  1.08028018501200  -1.53496655116496
H     2.82008022122536  -0.83784905095143 -1.70426584308754
H     1.76295671139004  -2.70705383490179 -0.03673564517773
H     1.33083591130417  -2.04699205306367 1.52990567628973
H     -0.40469407433087 -3.91431864256257 -0.20230548743283
H     -0.99774794574422 -3.13556439771499 1.27180129110553
H     0.34866172121894  -4.27346981477672 1.35129190197948
H     -0.93871563985662 -2.19183507678935 -1.44748570335513
H     0.40764462728279  -1.39798421396533 -2.29264064432085
H     -2.07175961779764 -0.22038170589636 -2.28363425322415
H     -0.64441641431244 0.80289798140525  -2.00193820120600
```

Coordinates of (S,S)-16, Conformer ID# 4
charge: 0, multiplicity: 1
35

```
C     -3.25517568454816 0.51526793745189  1.73737407776835
C     -3.18163086276685 0.13549695760380  0.27194069032642
N     -1.94256746082071 -0.02579946132966 -0.26825828742868
C     -0.68121171823061 0.01119711501685  0.45034885359767
H     -0.80519532533411 -0.42926603122936 1.44872077590695
N     -0.05521160574623 1.34838428470679  0.54789299512554
C     1.31997470702843  1.19553599146142  0.34114261511808
C     2.33395555268730  2.11354462490453  0.59925662641197
C     3.64119549619264  1.76024276872280  0.25844214108692
C     3.92963311796782  0.52992381582831  -0.32975422625693
C     2.89919990425703  -0.38182101666842 -0.58628323317660
C     1.60222711535405  -0.04750716790565 -0.24087479397013
C     0.30732016956578  -0.78927563540644 -0.44347395886488
C     0.34453379153729  -2.28955961016911 -0.13241006795390
C     0.83736294449976  -2.65030801894747 1.26739680584683
C     -0.27746106911674 -0.52553554297382 -1.85926535664696
C     -1.79200802704933 -0.47253650561503 -1.66332048205917
O     -4.19566498986637 -0.02773977289278 -0.40607975258019
H     -4.29097850763980 0.76220419387314  1.97113881839551
H     -2.61786056958155 1.37144958267736  1.97847995033583
H     -2.94375196385348 -0.32500658772612 2.36854531161022
H     -0.32410448683126 1.90774740272275  1.34602387296964
H     2.11826391970612  3.07666524004938  1.05388547219873
H     4.44711214836987  2.46070187203120  0.45983427300889
H     4.95435799117453  0.27680855289631  -0.58349511793569
H     3.11946281232675  -1.34567880051199 -1.03980666812256
H     -0.65907246371616 -2.70667385482141 -0.29501659122487
H     0.99433087933160  -2.76305637812791 -0.88025526363402
H     0.16355689502744  -2.28055106440778 2.04754202025480
H     1.83027293564543  -2.22951705409486 1.45631135823151
H     0.90142812291237  -3.73705184705221 1.38078011590600
H     0.02670923295424  -1.29952554392492 -2.56998100324743
H     0.08934751097008  0.43840017019379  -2.22665075187155
H     -2.27210380677152 -1.45174136082875 -1.79076609580628
H     -2.28485670563568 0.22570074449340  -2.34746512332001
```

Coordinates of (S,S)-16, Conformer ID# 5
charge: 0, multiplicity: 1
35

```
C     -3.19649674947285 0.52876724821052  1.76551678480055
C     -3.08894377921239 0.08697973628645  0.31960570469922
N     -1.83871250822305 -0.08272114523075 -0.18945350882209
C     -0.59113326796492 0.05290363907541  0.53598492337174
H     -0.71226435818035 -0.30320542121077 1.56968497406243
```

N	-0.02144691596267	1.41759527777875	0.51301184466632
C	1.35679875954572	1.31122825664192	0.31030814610267
C	2.32654893875641	2.29661157782618	0.47217961947013
C	3.64810941433806	1.97491651011011	0.15650602612978
C	3.99253118708921	0.70885783576786	-0.31287737015610
C	3.00580617204638	-0.27138992714043	-0.47209827471825
C	1.69471318382580	0.03291193358243	-0.15229221429695
C	0.43943311377321	-0.79818182898092	-0.25958351000566
C	0.61282726103552	-2.21708440600326	0.29710864428791
C	-0.64718304339765	-3.08132950177934	0.31112289184804
C	-0.14217012839573	-0.76306796690788	-1.70302208086391
C	-1.65856269001485	-0.58582101905787	-1.55994516812742
O	-4.08801477742308	-0.12564782771896	-0.36746213189946
H	-4.24054872279075	0.76778903775060	1.96874653152653
H	-2.57779532331484	1.40475948831868	1.98293642835759
H	-2.88410582428580	-0.27888811706592	2.43771222468682
H	-0.31994571161435	2.03875694677599	1.25255882389040
H	2.06592649962867	3.28734331152552	0.83448291421429
H	4.42010116218227	2.72904961951151	0.28313509948087
H	5.02704708305313	0.48062979364748	-0.54986617495549
H	3.27268562073356	-1.26110646722227	-0.83496340516131
H	1.38675269418154	-2.71741188820354	-0.29977104262542
H	1.01538213968426	-2.13281934379302	1.31518432756473
H	-1.45443367243239	-2.61747316760784	0.88868681437924
H	-0.43135353594683	-4.05375085562137	0.76463788374197
H	-1.02321747945453	-3.26700559700937	-0.69974644041381
H	0.11708033571549	-1.67009756497684	-2.25766226990574
H	0.28727781955199	0.08880073075334	-2.23879330001106
H	-2.21544951110147	-1.52300832042257	-1.67597517994647
H	-2.06467338595272	0.12609942239019	-2.28732853537209

Coordinates of (S,S)-16, Conformer ID# 6
charge: 0, multiplicity: 1
35

C	-3.27915365039377	0.54819773089757	1.71295569527284
C	-3.17821680823704	0.22841843428537	0.23625279987777
N	-1.92752144608317	0.03845288876468	-0.27812392333596
C	-0.71168154178847	-0.09020462103738	0.51066532313710
H	-0.92848266636670	-0.64002527856514	1.43571187000611
N	-0.05096517788483	1.19006270896847	0.86618291615392
C	1.28703315571598	1.13332468399489	0.45491296890825
C	2.27315122994788	2.09693404773908	0.64731624807716
C	3.56806790668175	1.80929159429965	0.21193538449224
C	3.87467167287270	0.59065931362803	-0.39165667361249
C	2.87015113759624	-0.36221146279739	-0.59391102964841
C	1.58210189778915	-0.08338028115660	-0.17217316330692
C	0.29914325265689	-0.84482428354572	-0.39882446081689
C	0.36153490081977	-2.35144602280565	-0.12533867157156
C	0.88753164515414	-2.73153990988730	1.25735763650563
C	-0.25326754199308	-0.54613509298981	-1.82230841002378
C	-1.76620637391743	-0.45538443493876	-1.65648992818189
O	-4.17665427102669	0.13179954792063	-0.47524172653864
H	-3.28283355774924	-0.38933601521479	2.28220753266347
H	-4.22948214795928	1.05408914222896	1.88809285774745
H	-2.45623334368652	1.16304627203395	2.08637551693206
H	-0.56826764995792	2.02984033793423	0.64085787310737
H	2.04574892426332	3.04135600033604	1.13404422098662
H	4.35183230517573	2.54720749751619	0.35987993547514
H	4.89173889209850	0.38358848514078	-0.71008452747741
H	3.09862066431229	-1.30401273025370	-1.08825901557113
H	-0.64036364339443	-2.77740971316924	-0.27769373520508

H	1.00324608086318	-2.80253164032139	-0.89368827430477
H	0.24545396595726	-2.35436134461428	2.06020774816093
H	1.89236379275114	-2.32806283811982	1.41853258290413
H	0.93716205730837	-3.81993320997588	1.36170067376650
H	0.04614976963256	-1.31392182910106	-2.54181474451202
H	0.14360835355139	0.41466990781425	-2.16741489855052
H	-2.26213705496006	-1.43023188531432	-1.76044085114852
H	-2.23594472974959	0.22950400030544	-2.36889575036870

Coordinates of (S,S)-16, Conformer ID# 7
charge: 0, multiplicity: 1
35

C	-3.20227447716547	0.57821786349705	1.75141006546000
C	-3.07563447996246	0.17450435388915	0.29740549824508
N	-1.81599088463654	-0.02643987147092	-0.18813697768425
C	-0.60507520008985	-0.04229052891147	0.61228049113399
H	-0.80480463945359	-0.51142721607875	1.58617567925284
N	0.00212071700079	1.29174576359580	0.84462484511848
C	1.33509989171144	1.25981721890048	0.42053006244764
C	2.27493283708162	2.28362637128390	0.50467795306586
C	3.57841939335033	2.01664626914905	0.08286467537739
C	3.93874970375465	0.76000443707957	-0.40075133460593
C	2.98016677989253	-0.25520108397563	-0.49415947751438
C	1.68271047896074	0.00237408578372	-0.08688148536565
C	0.44058895694367	-0.84973471984333	-0.20930939609922
C	0.63014449923203	-2.28100923448756	0.30632833539318
C	-0.61926401635138	-3.15962744721102	0.25873780220062
C	-0.11883141489860	-0.77739118812065	-1.66164910496412
C	-1.63142029548206	-0.58522342828222	-1.53805157392871
O	-4.06220345326435	0.01664493649906	-0.42017018695844
H	-2.41872948188366	1.26390233139283	2.08453769562040
H	-3.15058712324405	-0.31916522620982	2.38009400218151
H	-4.18121831121779	1.03739028444922	1.89342361206662
H	-0.55676722036981	2.08486624871919	0.55806211641680
H	2.00561488616371	3.25942723486274	0.89934322661397
H	4.32631067988007	2.80234772399270	0.14662192741942
H	4.96171222982128	0.57050925333002	-0.71099784650151
H	3.25195647864100	-1.22775407938283	-0.89803318487673
H	1.42692515715812	-2.75218446565770	-0.28396290364643
H	1.00372696912725	-2.22122104955947	1.33713559818144
H	-1.45438150316434	-2.71217897127442	0.80990772701182
H	-0.41355169739660	-4.13639950939059	0.70740325333594
H	-0.95218033432988	-3.33444932967077	-0.76918218201204
H	0.13863141730210	-1.67268135053288	-2.23597210634058
H	0.32755916951845	0.08236338765800	-2.17087353579472
H	-2.19043577304923	-1.52552789719201	-1.61952306823251
H	-2.03225993958010	0.09795883316956	-2.29436020201779

Coordinates of (S,S)-16, Conformer ID# 8
charge: 0, multiplicity: 1
35

C	-3.31577105633670	1.05161838172501	1.50027173883814
C	-3.20021531516771	0.29358778049208	0.19375171957911
N	-1.95490274563285	-0.09755247731165	-0.19083051115174
C	-0.73041722944959	0.02920080037570	0.58604966973713
H	-0.89635459578568	-0.32911022205263	1.61344822357427
N	-0.13462887782463	1.37507170266551	0.59656049703731
C	1.24705960227142	1.23719266096546	0.43265794801289
C	2.21780397528712	2.21792980511106	0.61512178715927
C	3.54112833210445	1.89873792628603	0.31098543295062

C	3.88183377956851	0.64048337055661	-0.18006875859431
C	2.89517089674022	-0.33536819160020	-0.35954220999768
C	1.58056689138516	-0.04712450165826	-0.02552771838896
C	0.31135031693467	-0.85650380288158	-0.16318045574496
C	0.30505649972555	-2.26894256001824	0.44839017357904
C	1.22625623757286	-3.30505457126980	-0.19273541374772
C	-0.23208391922557	-0.82389812235136	-1.61619129100919
C	-1.75062701914586	-0.79693989455530	-1.46894272079628
O	-4.18383678001894	0.07050902874815	-0.51209099013590
H	-4.36869674197300	1.08864080968341	1.78096284950129
H	-2.95740356935555	2.07840552906130	1.36256169484055
H	-2.73907730174476	0.59737208689699	2.31184803115461
H	-0.43544327846326	1.98697659161412	1.34318680568480
H	1.95179375867066	3.20754087670690	0.97671713659595
H	4.31382889608662	2.64938241262841	0.45280162275022
H	4.91490340896013	0.41462433641409	-0.42554240027460
H	3.16873524003877	-1.30485521376564	-0.76256132506427
H	0.55846441668216	-2.17507639274930	1.51233220426834
H	-0.73056479498252	-2.63664877430111	0.41143781168913
H	1.01450378517547	-4.29608437595674	0.22105099899174
H	2.27869650420700	-3.08907903408748	0.00690383656640
H	1.09006796401242	-3.36843604090671	-1.27698331029978
H	0.11625920787753	-1.67034076369236	-2.21282233096189
H	0.11250487246364	0.09467733920776	-2.10295816393315
H	-2.18777982467574	-1.80392463446304	-1.42157705676585
H	-2.25066153598203	-0.26008186551719	-2.28079552564454

Table S33: Electronic energy, correction to Gibbs free energy, and respective Boltzmann weights of the conformers of (***S,S***)**-14-Me** at the ωB97M-V/def2-QZVPP/C-PCM(Hexane)//r2SCAN-3c/C-PCM(Hexane) level of theory for the calculation of the ECD-spectrum.

Conformer ID #	Electronic energy [E_h]	Correction to Gibbs free energy [E_h]	Boltzmann weight [%]
2	-999.668538	0.368989	8.12
4	-999.667535	0.368256	6.1
5	-999.668696	0.369057	8.93
9	-999.666983	0.368292	3.27
11	-999.667905	0.368864	4.74
13	-999.668332	0.36882	7.81
14	-999.666979	0.368725	2.06
15	-999.667995	0.369224	3.56
18	-999.668126	0.368473	9.06
20	-999.666883	0.36902	1.36
22	-999.666364	0.368726	1.07
23	-999.667384	0.369126	2.07
24	-999.667627	0.368434	5.57
28	-999.666532	0.368984	0.98
29	-999.667525	0.368091	7.19
31	-999.667140	0.368708	2.49
32	-999.667272	0.368139	5.23
33	-999.667359	0.367932	7.14
34	-999.668063	0.369245	3.74
38	-999.667065	0.368019	4.76
39	-999.666560	0.368691	1.37
40	-999.667505	0.369186	2.21
41	-999.664712	0.367879	0.46
47	-999.665483	0.368215	0.73

```
Coordinates of (S,S)-14-Me, Conformer ID# 2
charge: 0, multiplicity: 1
49

      C     -3.46087865244506 -3.65634315960030 0.69264656226239
      C     -3.17977321799057 -2.43902343346071 -0.16487697903832
      N     -2.08015554065039 -1.69774429170821 0.15348786212966
      C     -1.79909471044448 -0.42350229450647 -0.55490816701693
      H     -2.75435976469614 0.06203973017978  -0.78629849509857
      N     -0.99439364975488 -0.54705748031383 -1.76553123612308
      C     -1.42118231779215 -1.42081680688326 -2.84353944927294
      C     0.35111248120094  -0.47801371728908 -1.39889056194445
      C     1.48118189166367  -0.83169574007242 -2.13515470911098
      C     2.73684727269840  -0.60858539100176 -1.57321736553395
      C     2.86944295372532  -0.05946510005599 -0.29596097396914
      O     4.15903002192464  0.10765113742285  0.15050883023573
      C     4.32930562015810  0.67921596762644  1.44205280261135
      C     1.73126006936931  0.27700275414391  0.44873202760393
      C     0.48525303381841  0.06833085193555  -0.12137414581170
      C     -0.88969779889465 0.36143228901562  0.43917044633430
      C     -1.21104635825341 1.87454473096001  0.42364383766010
      C     -0.38001235172097 2.67130793830996  1.38239686321295
      C     0.68407907035180  3.43291748762527  1.09545718444920
      C     1.42708330268620  4.16041783779407  2.18215492873056
      C     1.25100838835072  3.63048182198916  -0.28099370819817
      C     -1.13129450795799 -0.31749627126093 1.79301540012914
```

```
      C     -1.33318147146083 -1.78447493937235 1.41911403282303
      O     -3.90986050614827 -2.14311525215242 -1.11212284273067
      H     -2.55602615830009 -4.23367865948503 0.90448452759241
      H     -4.18228654153159 -4.28135090975988 0.16558658641665
      H     -3.90053740259667 -3.35376648609440 1.65031537093285
      H     -0.85817190356565 -1.18028025945413 -3.75171601569161
      H     -2.48344346810269 -1.26182346731163 -3.03753265348328
      H     -1.26931382386099 -2.48827482583010 -2.61018791069864
      H     1.40261606684604  -1.27554452999713 -3.12252065627548
      H     3.63724343635254  -0.87240126965510 -2.12015008065985
      H     3.89193968514789  1.68639621229115  1.49667462879827
      H     5.40702497158092  0.74432524069795  1.60294891543813
      H     3.88256172483972  0.04920899996923  2.22384629806321
      H     1.80493697374583  0.70247497839509  1.44295132033955
      H     -1.09853278013257 2.23506283661254  -0.60419746108139
      H     -2.27272992253572 1.98593534420510  0.68679485520679
      H     -0.67603838915625 2.59821197223974  2.43038688017915
      H     2.47667253571017  3.83726226465531  2.21566603214534
      H     0.98126572799532  3.99191677734636  3.16644891292115
      H     1.44247722111164  5.24087193348134  1.98807973736426
      H     0.71705545995688  3.07667213877008  -1.05477837852362
      H     2.29829778675876  3.30071630971615  -0.30356782902998
      H     1.25069418103723  4.69603565988711  -0.54575510246082
      H     -2.04440425924314 0.08121985088292  2.25448645739728
      H     -0.29880351455624 -0.17079664701409 2.48734012642388
      H     -1.89548665592427 -2.33508034067666 2.17608455284918
      H     -0.36672420931479 -2.28581179319680 1.26628874150313

Coordinates of (S,S)-14-Me, Conformer ID# 4
charge: 0, multiplicity: 1
49

      C     -3.75518050853541 -3.08539647996129 0.03247237350734
      C     -3.18012940911682 -1.73238215344017 -0.33552409358493
      N     -1.93502685828822 -1.44114690158754 0.13614284824359
      C     -1.33064341717564 -0.10735575483431 -0.08252696182722
      H     -2.13213886982060 0.63995562299354  -0.09371529901295
      N     -0.52777260589287 0.00176136978904  -1.29598127452154
      C     -1.09274101660979 -0.35159432504083 -2.58319757156033
      C     0.80014120532479  -0.27721006387678 -0.98948496366163
      C     1.86464275745408  -0.54028641462506 -1.84278339580892
      C     3.14549785919685  -0.71127369364272 -1.29550954385107
      C     3.34621860743948  -0.63019420274219 0.08123031077426
      O     4.55992810213751  -0.78753635753081 0.70513517117166
      C     5.68944605596149  -1.04090312044244 -0.12349070133791
      C     2.26054043599907  -0.38427802893622 0.93857015158214
      C     1.00666862755154  -0.21538900816373 0.39558502863291
      C     -0.31110384952092 0.04812693288224  1.09091424568184
      C     -0.36395337856338 1.44222777273871  1.75241923789489
      C     -0.08440252913319 2.55972623498314  0.79482947726163
      C     -0.94575579963770 3.47638493051696  0.33621705896886
      C     -0.50375117407821 4.51168060766515  -0.66193071324975
      C     -2.39579485415065 3.57410851160332  0.72058196491483
      C     -0.67773317070813 -1.08044782299330 2.07116969350122
      C     -1.20059815315437 -2.19150513161128 1.16442058391723
      O     -3.81120077918694 -0.93899601093605 -1.03646447896910
      H     -4.63282933302851 -3.25983399248545 -0.59041016189643
      H     -4.06657723236116 -3.09688795887074 1.08348484989617
      H     -3.03285360917082 -3.89436369570848 -0.11314982559149
      H     -0.47732790500055 0.07444152706622  -3.38302214564866
      H     -2.10060219317359 0.06161280962855  -2.65971861415067
      H     -1.15466080446619 -1.44305014673301 -2.73512283600527
```

H	1.72829595255326	-0.61569250723038	-2.91693254772787
H	3.97134895649369	-0.91511402194049	-1.96735776338457
H	6.54326628070153	-1.12781673220331	0.55098161967970
H	5.86372250653397	-0.21511876801552	-0.82699562445225
H	5.57264978171570	-1.97789398921502	-0.68536835898094
H	2.43336193816449	-0.33800803918830	2.01116206814248
H	-1.34277462258661	1.55508836666153	2.23054663665825
H	0.38628085209556	1.44981175247673	2.55697098551722
H	0.93614442983596	2.59131961962368	0.41118444887538
H	0.55399267784993	4.40472761864512	-0.91699794984067
H	-0.66805263215310	5.52527415896428	-0.27298782412835
H	-1.09245724839421	4.43154652086516	-1.58544609614631
H	-2.61663620880601	4.57065972550206	1.12453357985488
H	-2.70555784456036	2.83150914821658	1.45802326670188
H	-3.02889164014291	3.45167107032913	-0.16820659510377
H	-1.47804819583859	-0.74483300787313	2.74310170384444
H	0.17844807252321	-1.39287112685138	2.67652092449240
H	-1.85273744876325	-2.89181145690090	1.68990025454150
H	-0.37088180751341	-2.75638338757035	0.71480685618594

Coordinates of (S,S)-14-Me, Conformer ID# 5
charge: 0, multiplicity: 1
49

C	-3.44062669336912	-3.71560887004946	0.67721090533529
C	-3.14703450093936	-2.50518294867828	-0.18591617336831
N	-2.08606548926328	-1.72902885272498	0.17656332670912
C	-1.80114593521827	-0.45933772783000	-0.53878151410343
H	-2.75506351988062	0.00056593782562	-0.82247153670917
N	-0.93770796979238	-0.58010531105181	-1.70765895367568
C	-1.29831928250321	-1.45929270017745	-2.80448367270549
C	0.38774479038653	-0.48021395953599	-1.28448492982880
C	1.55319512547145	-0.82111219397960	-1.95725988691470
C	2.78869948746260	-0.56721189399120	-1.34154527661169
C	2.84268562660930	-0.00200576678388	-0.06915500259929
O	4.00252324214192	0.27688366283967	0.61355764128138
C	5.23035290737174	-0.03536948209476	-0.03560127987898
C	1.65815359813822	0.31976007626458	0.61352251058966
C	0.44885918194938	0.08781911590642	-0.00340106690862
C	-0.95772826211083	0.36095700942145	0.48374037558595
C	-1.30616248960365	1.86711084470557	0.42341485013826
C	-0.52985302201755	2.69554935171443	1.40082818947242
C	0.54588756589560	3.45246555099428	1.14737186347872
C	1.24039427346410	4.20022497015148	2.25203202518429
C	1.17865760378131	3.61842382314813	-0.20456956940146
C	-1.25075605105459	-0.29918440062513	1.83616977413177
C	-1.40203688111341	-1.77667015195041	1.47963250204931
O	-3.83407355819188	-2.24434495161843	-1.17486588570036
H	-2.53325330267419	-4.26482842768915	0.94555873561265
H	-4.11728768417965	-4.36825601978116	0.12484571503299
H	-3.93694204145094	-3.40928153674266	1.60555666510421
H	-0.70322529889881	-1.20220977807920	-3.68762063928839
H	-2.35517569757483	-1.32556833825559	-3.04200368485241
H	-1.13017918714062	-2.52347072602953	-2.56725237763265
H	1.53004964364365	-1.28069508342311	-2.94027206885286
H	3.69546090971751	-0.83399134676791	-1.87221405978595
H	5.34043006562649	0.52340561141298	-0.97527837839547
H	5.31283647070654	-1.11145908484058	-0.24179832538605
H	6.02040010074802	0.26164908403518	0.65659872206445
H	1.71916350268546	0.75331994716901	1.60740974092832
H	-1.15540306274101	2.20949191604206	-0.60589539454199
H	-2.38015852760867	1.96246210231974	0.63794703142735

```
H      -0.87474328882549 2.64483792190976  2.43504950609099
H      2.28094566915225  3.86188256943643  2.34879852386707
H      0.74085560202979  4.06355862651919  3.21522026851867
H      1.28138327735102  5.27472372354892  2.03063430806605
H      2.22159055319766  3.27666737795591  -0.17006571915637
H      1.20234518120331  4.67895514580620  -0.48784668860062
H      0.67489254584898  3.05616141945142  -0.99254612602110
H      -2.19424159342899 0.08686594087219  2.24412071442232
H      -0.45680372339654 -0.12139333813884 2.56719673463812
H      -1.98999600638000 -2.32667054530047 2.21736731057869
H      -0.41856385522495 -2.25891829331105 1.38489027061176
```

Coordinates of (S,S)-14-Me, Conformer ID# 9
charge: 0, multiplicity: 1
49

```
C      -3.74699492974666 -3.06995063755945 0.15638622596222
C      -3.18414016073742 -1.71920697737071 -0.23787373903374
N      -1.90501399060531 -1.44644114015643 0.14640578849408
C      -1.30459935767957 -0.11560397930622 -0.09759887520220
H      -2.09873398331912 0.63796522783458  -0.04851959652939
N      -0.58214548414984 -0.00032569706207 -1.36131605341908
C      -1.23143330470689 -0.36440809122170 -2.60585430698186
C      0.76190701204966  -0.28786782186524 -1.13812765352837
C      1.77271664156247  -0.54222111345283 -2.06616210425177
C      3.07404985045448  -0.71826425857630 -1.60086828294915
C      3.37171271892977  -0.65191504424617 -0.23743663839606
O      4.69161400702339  -0.84243083776525 0.09364183278292
C      5.03235974093658  -0.77539086938098 1.47355379246785
C      2.35057664718092  -0.41528090976021 0.69340644613913
C      1.05812748290104  -0.24237724464941 0.22411052797148
C      -0.20892508886191 0.01929233780429  1.00838701093634
C      -0.21034929317124 1.40494690077061  1.68952605498899
C      0.01851275820347  2.53205525078163  0.72972468774118
C      -0.86127408295340 3.46173126913581  0.33704670169635
C      -0.47376082005953 4.50336254216238  -0.67696139834538
C      -2.28281414166224 3.56813825684880  0.81426818821812
C      -0.51850436532128 -1.11963677260155 1.99687092591860
C      -1.10872545860014 -2.21521054723451 1.11303444532829
O      -3.85447404368682 -0.91144068011891 -0.88354311075122
H      -4.66951056966768 -3.22727463109256 -0.40296478307520
H      -3.98149601237574 -3.09022689958318 1.22703707967958
H      -3.04696788969884 -3.88521186476053 -0.05028045898381
H      -2.23902300057184 0.05636314280160  -2.62206682649080
H      -1.31132489489251 -1.45690189629299 -2.74104959083498
H      -0.66658517230081 0.04810552894665  -3.44856498260767
H      1.56720502291498  -0.60428179315576 -3.13016707029432
H      3.88305633797191  -0.91725743064629 -2.29753844470155
H      4.51855832217278  -1.55511196627860 2.05320819046756
H      4.79416394794888  0.20955847871133  1.89883642316128
H      6.11021905901199  -0.93971526655067 1.52551641212477
H      2.55126291439965  -0.37088724885657 1.75923688967804
H      -1.15678089093043 1.51853384968062  2.22887222965902
H      0.58785953456663  1.39753102407289  2.44683396858777
H      1.01242886269103  2.55817486030710  0.28117917459757
H      -1.12192272394699 4.43903844359761  -1.56103618199126
H      0.56393832070845  4.38834156840463  -1.00138634188048
H      -0.60169935696400 5.51430497225888  -0.26801381416545
H      -2.46931169621225 4.56389585133136  1.23699247917554
H      -2.55051467789839 2.82344846199348  1.56596087201618
H      -2.97251747227571 3.45591666574319  -0.03275231565851
H      -1.26948306459439 -0.78788528380168 2.72536006981056
```

```
      H     0.37365191579366  -1.44617698550999 2.53997280860759
      H     -1.72900228013898 -2.91867891772742 1.67178504003301
      H     -0.31498288969182 -2.77857182660326 0.60097830382824

Coordinates of (S,S)-14-Me, Conformer ID# 11
charge: 0, multiplicity: 1
49

      C     -3.60929762898396 -3.59740067962722 0.09696908641691
      C     -3.25082719052764 -2.18158182283838 -0.30719216281855
      N     -2.05113925451825 -1.69954027085498 0.12560160822342
      C     -1.66468446927587 -0.29019062133504 -0.12644049761508
      H     -2.57223663497802 0.32395351271597  -0.09918147789704
      N     -0.94433454932077 -0.06750185794118 -1.37609756346341
      C     -1.51452701132724 -0.50642621624014 -2.63600004780405
      C     0.42151260746410  -0.18753671883234 -1.12686119384888
      C     1.47414907381778  -0.31147361520747 -2.03410363222406
      C     2.77751535133570  -0.32761384910793 -1.54173520428974
      C     3.03575950000642  -0.23489424529932 -0.17189812990344
      O     4.36218690106549  -0.25984223737125 0.18773255998981
      C     4.66181637093585  -0.12528335724670 1.57209670325918
      C     1.97448423392439  -0.13213291863427 0.73720864183565
      C     0.68175289124666  -0.11005152834654 0.24089540141183
      C     -0.62287802565192 0.02174525226574  0.99415082356770
      C     -0.78846107618693 1.42808783115309  1.61577230371861
      C     -0.72345468255001 2.53590273158755  0.60769700798653
      C     0.30554948178182  3.35483030013700  0.35688703911377
      C     0.22109250192654  4.37942640006483  -0.74120428006374
      C     1.61880530775529  3.34069305910064  1.08634250453089
      C     -0.81863715260612 -1.10482316259456 2.02049765157603
      C     -1.20594756404175 -2.30752604014421 1.16394236830919
      O     -4.00978345047433 -1.50448132211482 -1.00266694472323
      H     -4.45472864866425 -3.91813521466651 -0.51225068828818
      H     -3.90902574859486 -3.63013375493076 1.15097039932054
      H     -2.77340660318809 -4.29061668230802 -0.03716410470717
      H     -0.98690455567372 -0.02143241130608 -3.46411231939490
      H     -2.56674693062504 -0.21766771413998 -2.67517693263677
      H     -1.45169306633860 -1.59939059029943 -2.77386570211102
      H     1.30038236636200  -0.39191056631092 -3.10249226414099
      H     3.61925143096729  -0.41990543945701 -2.22177790242957
      H     5.75062052051917  -0.14352865903872 1.64769149956975
      H     4.24433423319245  -0.95639963797938 2.15762951854794
      H     4.28535459188590  0.82640957486425  1.97320854687266
      H     2.14669297507400  -0.06476552700075 1.80674132343434
      H     -1.76182348501666 1.45230643389552  2.12642901482667
      H     -0.02055031171576 1.54210038714865  2.38945325921516
      H     -1.61126981915844 2.64602451852520  -0.01611896628708
      H     1.01134919824155  4.20978466100283  -1.48460387937898
      H     0.37511239323934  5.39200890496725  -0.34556552771628
      H     -0.74482724523478 4.34900343750122  -1.25262844035050
      H     1.86341715026629  4.34716655982552  1.44989934284062
      H     2.42469234966319  3.04598262990989  0.40083099989548
      H     1.63683993891963  2.65177970258739  1.93234076636596
      H     -1.64215282820387 -0.85162296731051 2.70056476174141
      H     0.08281975399292  -1.28120045485170 2.61547135712296
      H     -1.75213207458266 -3.06633188583259 1.72794077825807
      H     -0.31649111614421 -2.77501392808383 0.71732259414156

Coordinates of (S,S)-14-Me, Conformer ID# 13
charge: 0, multiplicity: 1
49
```

```
      C     -3.61561496401256 -3.60648698613963 -0.00555899895711
      C     -3.23489689295667 -2.19830673586844 -0.41603198424712
      N     -2.08301247855616 -1.69142552870852 0.10798810662602
      C     -1.68670597482194 -0.28538453812144 -0.14956718852489
      H     -2.59753723319328 0.32115877208998  -0.21440236326113
      N     -0.86598854105226 -0.08826982124236 -1.33906083122662
      C     -1.32981485626269 -0.53987782319217 -2.63682859828304
      C     0.47517254153734  -0.19351255315819 -0.98036940323683
      C     1.59477472877071  -0.33904253659775 -1.78945438594136
      C     2.86508469847820  -0.34402323414100 -1.19296302434974
      C     3.00100015922967  -0.22030807488424 0.18857411802685
      O     4.20009475718120  -0.21855073555707 0.85961222121635
      C     5.38306830818150  -0.34732437546967 0.07818449833333
      C     1.86194257334610  -0.09349571475263 1.00029280747612
      C     0.61965527849487  -0.07958166211999 0.40872342566305
      C     -0.74465137933347 0.06548864955116  1.04489330655152
      C     -0.96687002447884 1.48882920252008  1.60670626760880
      C     -0.82597746501250 2.56572376051574  0.57284507697238
      C     0.21681908950491  3.38256812203097  0.37884569954006
      C     0.21489508010420  4.37260457389431  -0.75385095321189
      C     1.46880608355028  3.39839854406488  1.20879150551747
      C     -1.02060749356661 -1.03209599583843 2.08348013287210
      C     -1.32651705855692 -2.26209280892747 1.23260327339271
      O     -3.93461594066309 -1.54873878187520 -1.19505270901716
      H     -2.76453050560011 -4.29314303856354 -0.04255298154703
      H     -4.39897357481324 -3.95431014028600 -0.67949169337961
      H     -4.01002140489299 -3.61284081627596 1.01729356074688
      H     -1.24351406594729 -1.63280126985731 -2.76190403142196
      H     -0.74290546990828 -0.05361122631548 -3.42359299386035
      H     -2.37888917808074 -0.26416291294624 -2.76097506649182
      H     1.50985306355831  -0.44751518399319 -2.86607380100486
      H     3.73482504132085  -0.45622209479990 -1.83013157470329
      H     6.21451286400998  -0.31874863343485 0.78484633171409
      H     5.48377876146098  0.48139075192654  -0.63623683672530
      H     5.40169599421223  -1.30091352026129 -0.46737810605728
      H     1.98710459251812  -0.00511359750712 2.07686425501082
      H     -1.97900249678556 1.52091441883761  2.03488990208179
      H     -0.26524028110241 1.63222179232979  2.43623403748365
      H     -1.66207001967049 2.65166918893067  -0.12223588456593
      H     0.33307983186659  5.39780490198861  -0.37890215862773
      H     -0.70733300242733 4.32034044770462  -1.33895917411515
      H     1.06237124838076  4.18541071829922  -1.42673070936733
      H     2.32725577472545  3.08700110375105  0.59879983555061
      H     1.42436300039353  2.73689178162998  2.07519206460299
      H     1.68096798648340  4.41733819438426  1.55773160663898
      H     -1.90101001063589 -0.76590190057097 2.68247633720780
      H     -0.17237693180137 -1.18344093941931 2.75798534971105
      H     -1.91453496230846 -3.00880476424977 1.77010008734779
      H     -0.39932925086802 -2.73511697937431 0.87792164423134

Coordinates of (S,S)-14-Me, Conformer ID# 14
charge: 0, multiplicity: 1
49

      C     -3.71860044069922 -2.52045249816724 0.71232941338657
      C     -3.03336250598855 -1.58711030759828 -0.26516349993775
      N     -1.80125634362390 -1.11988789561267 0.08524518309416
      C     -1.11253066322502 -0.10813063928721 -0.75224247138489
      H     -1.87301113644414 0.54468009192362  -1.19241284614331
      N     -0.25648858746687 -0.65466050123312 -1.80091741213019
      C     -0.80010564511532 -1.57439615987463 -2.78266105353918
      C     1.01892887336135  -0.85050194615488 -1.27283513359917
```

C	2.07578020176054	-1.59905433844501	-1.77387829965881
C	3.30324968028483	-1.57735939239546	-1.09398621604113
C	3.45649639387991	-0.82638566410588	0.07006083759780
O	4.61548458653341	-0.74856157264526	0.80265660079492
C	5.73881725457262	-1.48080837229759	0.32409438568576
C	2.37591007159987	-0.08765247600352	0.58098341238205
C	1.17442978019282	-0.11287437516256	-0.09117001469494
C	-0.12616494537962	0.58800169709905	0.23726322680400
C	0.01057634692447	2.11335956688948	0.00488721053224
C	-1.26682502415421	2.88485948321616	0.15852676863085
C	-1.66576858797351	3.59341766971953	1.22361456792522
C	-2.98420175549277	4.31719651239224	1.22217454725206
C	-0.87854768841241	3.74179537180248	2.49522056760485
C	-0.65141739886392	0.21081651470123	1.62800037757049
C	-1.20780865676682	-1.19696259229081	1.42945798166721
O	-3.56739549270261	-1.27560642176336	-1.33114514522425
H	-4.11993807960298	-1.95523104701137	1.56159957400797
H	-3.03821948489833	-3.28164214091652	1.10576121161138
H	-4.54917750657599	-3.00084069542753	0.19444501235452
H	-0.11130732597238	-1.65119472551289	-3.63097008089428
H	-1.75819491845384	-1.19395098375644	-3.14174769027651
H	-0.96264882227617	-2.58531783436788	-2.37167080967263
H	1.97380511570284	-2.19776830833756	-2.67346124109282
H	4.12422914192500	-2.16364479363751	-1.49032055443882
H	6.03935077478822	-1.14405743124047	-0.67757778995144
H	5.53276228373131	-2.55972377866643	0.29794754925754
H	6.54802846969958	-1.28436393647141	1.02978564198944
H	2.51039842371942	0.47957123566249	1.49885580261896
H	0.78654918330662	2.47938148848572	0.68595702008385
H	0.40059111641657	2.25473344934119	-1.01189740688181
H	-1.94100127896471	2.85447707042635	-0.69805253115744
H	-3.52391570632075	4.17853527552797	0.28155892134785
H	-2.83622288756820	5.39320476748474	1.38266792951880
H	-3.62174981447785	3.96423387221244	2.04348616818385
H	-0.68559390638070	4.80224799970172	2.70279209929102
H	0.07738038204745	3.21521879332679	2.47832103983274
H	-1.46014940656203	3.36046432051378	3.34500863367713
H	-1.46037090321965	0.89413699112003	1.91274885945310
H	0.13290483201085	0.25350952119703	2.39019303421585
H	-1.95605376314519	-1.46073996018642	2.17961092571891
H	-0.40458423573001	-1.94751090417413	1.45685569262830

Coordinates of (S,S)-14-Me, Conformer ID# 15
charge: 0, multiplicity: 1
49

C	-3.67051896370769	-2.64858957364845	0.27366766736740
C	-2.98399770790929	-1.44250986755770	-0.33490914627235
N	-1.73508185130388	-1.14383411723497	0.12338209047662
C	-1.04919132361184	0.09408558753065	-0.32283428190854
H	-1.80614444868877	0.87073335887262	-0.46783978803995
N	-0.23130986452847	-0.04838924980512	-1.52244252084508
C	-0.81819499487661	-0.53609554324600	-2.75655231042078
C	1.04747127137344	-0.44963696349398	-1.13578254956271
C	2.07210679676124	-0.99008799513846	-1.90094688400237
C	3.31008141933992	-1.24550993600011	-1.29113136494568
C	3.50522860073940	-0.97048729691797	0.06120277610910
O	4.67710173504745	-1.19189096640091	0.74196420592088
C	5.76754880215184	-1.73267380215635	0.00314641722808
C	2.45732986865307	-0.43948736291834	0.83247888059925
C	1.24503496696679	-0.19194072629366	0.22833885827353
C	-0.02572202322590	0.38475960932030	0.81453499694968

C	0.15410996007488	1.89784740567436	1.09362252080090
C	-1.09182920898101	2.60063291795471	1.54640004820036
C	-1.92544416186719	3.32980898249852	0.79119400730376
C	-3.14767199828270	3.97138716584195	1.38790720597786
C	-1.74964217889931	3.58196371922870	-0.68018962550634
C	-0.54138859815556	-0.43154577908291	2.00644879508455
C	-1.12538370606200	-1.68130483984234	1.35055871348366
O	-3.53127705428056	-0.77196034106319	-1.21221527939078
H	-4.05132899501535	-2.40600390944329	1.27268614422282
H	-2.99710854409826	-3.50564539380605	0.36865472906072
H	-4.51595019272791	-2.91259660875008	-0.36228733707175
H	-0.99965729290019	-1.62416997913162	-2.73857939724842
H	-0.14852261370611	-0.30953052542584	-3.59341367322400
H	-1.77283258930174	-0.03386047284121	-2.92444497645709
H	1.93694622830279	-1.22066235794170	-2.95296496508519
H	4.10547697548181	-1.66878282630362	-1.89364542049382
H	5.52349715241419	-2.72329901620920	-0.40464396628074
H	6.59283183972184	-1.82636487011485	0.71133390903115
H	6.06555615671730	-1.06549085573766	-0.81730350088586
H	2.62479721017197	-0.24421560140727	1.88888806517593
H	0.93066985648863	1.99306419380084	1.86444618642714
H	0.56430253780178	2.35506714051843	0.18676606203457
H	-1.34109460822885	2.49630436195870	2.60277124172805
H	-3.23244644792586	3.76951702702754	2.45922201106672
H	-4.05636719698159	3.60585856844146	0.89196846614901
H	-3.12664719327347	5.05901605570418	1.23962173977184
H	-1.68446322262120	4.66035500618575	-0.87500068749058
H	-2.62776040634409	3.21940503521076	-1.23106393038364
H	-0.86595764101565	3.10047135571636	-1.10375281045663
H	-1.33792149184880	0.11729359971415	2.52230519980350
H	0.25191319736420	-0.65988664987324	2.72499069122749
H	-1.86780825183269	-2.17611394942889	1.98003845352470
H	-0.33423980337001	-2.40474371398498	1.10646433297302

Coordinates of (S,S)-14-Me, Conformer ID# 18
charge: 0, multiplicity: 1
49

C	-4.64414255865044	-1.47448189251526	0.44569685032187
C	-3.48493704640034	-0.85132265493297	-0.30048221457130
N	-2.24470013506305	-1.08406840750794	0.21879104227048
C	-1.06510400990042	-0.39366179003002	-0.28320290636970
H	-1.36421919311617	0.58189225784863	-0.69443369978190
N	-0.27948709675374	-1.15643360489472	-1.27245346250076
C	-0.74286881458434	-1.12661245232444	-2.65208726890058
C	1.07066573800296	-0.89326469360994	-1.00510872081687
C	2.17806168687042	-1.09593052144820	-1.81670111501614
C	3.45403037401710	-0.80442206625742	-1.31075162486445
C	3.60874487207655	-0.31318738544605	-0.01560343224737
O	4.81406031136217	0.00591120528929	0.55878687571065
C	5.98376765408243	-0.18768383937106	-0.22981540333862
C	2.48350866662187	-0.09770763059292	0.79757488564949
C	1.23438002333135	-0.38138626642065	0.29285048150602
C	-0.12147253455956	-0.28655100368016	0.93570983908859
C	-0.37639731766295	0.96928953090338	1.79039905335639
C	-0.21366784808878	2.25741549689643	1.04074686816636
C	-1.18952255175667	3.03869301920958	0.55876934284306
C	-0.86703569546772	4.29381705798893	-0.20457972375408
C	-2.65963755185042	2.75473626135596	0.68951679949423
C	-0.48086861509865	-1.57699808921726	1.71819354874140
C	-1.98732386861242	-1.75705824089734	1.50626913955414
O	-3.65455665658539	-0.17529072933129	-1.31600045724504

```
H     -4.74953794217128 -1.02557191321374 1.44023192312226
H     -4.50073147997350 -2.55094469925827 0.58544947667652
H     -5.55643774764726 -1.29778657455714 -0.12390772563829
H     -1.80729924676951 -1.37039427050767 -2.67976711348801
H     -0.20274106217615 -1.87421751519360 -3.24173432710021
H     -0.59958040595774 -0.13655878602806 -3.11876952908206
H     2.07952605370663  -1.46811621984616 -2.83162797027993
H     4.31284879614847  -0.95991860601820 -1.95342964492132
H     6.10441780165228  -1.24171185809501 -0.51538628758826
H     6.82300393498613  0.11519769002965  0.39894892314274
H     5.96421146973001  0.43422215431310  -1.13529148479734
H     2.62214325123140  0.30234883596229  1.79876686793361
H     -1.37662939016765 0.89006435537789  2.23151772614406
H     0.33161453918822  0.94076189554434  2.63034980993962
H     0.81640026031030  2.55730256628698  0.84655056824407
H     0.21143216687369  4.45165898232711  -0.29095740025037
H     -1.30945091999055 5.17124644165575  0.28510768385183
H     -1.29457003880037 4.25123860133477  -1.21494583013114
H     -2.88120886088242 1.86975917009534  1.29001302914776
H     -3.10260272845701 2.59735616301160  -0.30282263925042
H     -3.17775527324686 3.61389962443165  1.13441893861851
H     -0.21717355907682 -1.49831796076818 2.77728020835148
H     0.06491971301861  -2.42334978329074 1.28975449138049
H     -2.56792781548413 -1.28155358684888 2.30861520656995
H     -2.26841934825831 -2.81584826775937 1.46833040210857

Coordinates of (S,S)-14-Me, Conformer ID# 20
charge: 0, multiplicity: 1
49

C     -3.60100003127538 -3.14720570328683 0.82394721256570
C     -3.10018341731964 -2.18141123707548 -0.23094244399532
N     -1.98112619829621 -1.46274622026752 0.06928075179474
C     -1.48890901587242 -0.40987229573409 -0.85454594590070
H     -2.35429844706086 0.07516072742401  -1.32106244449952
N     -0.55892872552017 -0.86746986186722 -1.88081119935380
C     -0.94069619902803 -1.92272700486973 -2.80098187699804
C     0.73051845061802  -0.82337799268910 -1.35011260469116
C     1.88846219917416  -1.44058400590043 -1.80316128192973
C     3.09198396052679  -1.20719071196403 -1.12043531772315
C     3.12016895561794  -0.38417056170306 0.00349402277507
O     4.24506882719456  -0.10885520547819 0.74176534089563
C     5.46453600239501  -0.71002217218917 0.31942988545290
C     1.94010902205709  0.22259225105897  0.46598099724629
C     0.76409222039461  0.00407067400394  -0.21793834469398
C     -0.63357336181447 0.51802011839221  0.06156083755080
C     -0.81007562456036 2.00566734667102  -0.32139858203850
C     0.03643553832383  2.93663173370294  0.49235968504550
C     -0.35529487927645 3.73351476393212  1.49546067064430
C     0.63839117648040  4.59917664137782  2.22191343099246
C     -1.76484464787819 3.85597636197119  2.00283035063789
C     -1.10259057106287 0.18579917469716  1.48323532159592
C     -1.40097036129085 -1.31005370329838 1.41411907089979
O     -3.67732124013408 -2.06264173813760 -1.31316269136393
H     -4.31404927476462 -3.82333391666570 0.35139010352283
H     -4.11755905484553 -2.60381956912339 1.62381658853416
H     -2.78946850396452 -3.72366601799557 1.27796823609597
H     -1.95042085574170 -1.73259756484102 -3.16929825665856
H     -0.92703035189566 -2.92061007122081 -2.33065864729803
H     -0.25100419912625 -1.93047363810022 -3.65202394765352
H     1.88170853008142  -2.10223830219100 -2.66349620380401
H     3.99238718830191  -1.69279555362008 -1.47863112104687
```

H	6.22655669576297	-0.36966272294954	1.02292944361899
H	5.73806993614705	-0.39126617057683	-0.69580789841850
H	5.40333124723126	-1.80660371015409	0.35145566079773
H	1.97861866839844	0.84353240093539	1.35678432947301
H	-0.54562667127548	2.10819291053799	-1.38304528365672
H	-1.87474693248167	2.24900364101702	-0.23168505732703
H	1.09491343135029	2.94448077666096	0.23278937192430
H	0.64585601211140	4.36492666740405	3.29477922363297
H	0.36654647956845	5.65952280655347	2.13652733212531
H	1.65201523942587	4.46960571538580	1.83280611518861
H	-2.08209881502892	4.90668170971174	1.99271077413177
H	-1.82000727701521	3.52536198765261	3.04862787020101
H	-2.48756251277916	3.27508503868907	1.42721344819699
H	-2.02205834957183	0.74029957750629	1.71049163596969
H	-0.35219654237724	0.43685059394444	2.23801959093709
H	-2.10032328842028	-1.63322944235070	2.18793333440185
H	-0.47745443148337	-1.89923852498044	1.50828851220178

Coordinates of (S,S)-14-Me, Conformer ID# 22
charge: 0, multiplicity: 1
49

C	-3.74236913477815	-2.48371425267647	0.74802733678849
C	-3.06582278345404	-1.56106681660853	-0.24549626588270
N	-1.80631002195499	-1.13744421362418	0.06117860395802
C	-1.11964887283352	-0.13754925830490	-0.79152609679070
H	-1.87666798282460	0.54038889042505	-1.19832991652859
N	-0.31784225275790	-0.69900218510527	-1.87598531353982
C	-0.92294878689149	-1.61383201497821	-2.82697153500960
C	0.97094185449592	-0.92836357490692	-1.39204638312475
C	1.99490124600613	-1.69839405240443	-1.94353218122996
C	3.23542977403972	-1.70712338121516	-1.30937521124490
C	3.45789767818925	-0.97104179133568	-0.14284911368777
O	4.72181783586269	-1.06426990797791	0.38700163476343
C	4.98873023893253	-0.31975679613636	1.56994336251113
C	2.42110886729470	-0.21232059401755	0.41832881256231
C	1.18895561888575	-0.21037392590714	-0.21638532535799
C	-0.07884364228861	0.52147437627587	0.16758725343463
C	0.08905768940604	2.04544275900138	-0.05432384656935
C	-1.16006786505565	2.84906102190259	0.15669705050947
C	-1.49708343250420	3.55823331308706	1.24253158761944
C	-2.79423769276019	4.31730620869590	1.29993009572584
C	-0.65611285561277	3.67331188184385	2.48288283630703
C	-0.56334622481327	0.14337449500205	1.57318249426723
C	-1.16577900313952	-1.24620178856761	1.38126582816301
O	-3.63030798076614	-1.22042109309714	-1.28644389345940
H	-3.07203620686673	-3.27049392249116	1.10682269607195
H	-4.60701141394564	-2.93171993152822	0.25763627119378
H	-4.09277348708682	-1.91607675738076	1.61799947592244
H	-1.88195521024551	-1.20926973758871	-3.15609786272075
H	-1.09978788610373	-2.61523017113512	-2.39896756699274
H	-0.26848057039798	-1.71930922711867	-3.69872649305075
H	1.84537037544766	-2.28447204296531	-2.84479959461958
H	4.05335352767425	-2.29683387449567	-1.71253698693438
H	4.84996560617073	0.75797025365275	1.40608633326216
H	6.03333848105556	-0.51531831868942	1.81887782533219
H	4.35048937507247	-0.64576470361273	2.40313334853604
H	2.56312995173373	0.35683808798733	1.33151588311763
H	0.90036517229591	2.38451187355135	0.59918556549601
H	0.44280185002800	2.18731902071173	-1.08423254823366
H	-1.86818974773051	2.84436086770507	-0.67252937819987
H	-2.61083913157256	5.38713382539733	1.46513265085616

H	-3.40894584368379	3.97262335175240	2.14190487643765
H	-3.37353808875005	4.20356503567669	0.37977128109069
H	-1.21696143378196	3.30833108986517	3.35350631807795
H	-0.41807129722826	4.72539987590271	2.68640200276368
H	0.27934959757535	3.11369530215634	2.42630979779081
H	-1.34136664721571	0.84563000660620	1.89573246365007
H	0.24931979783508	0.15550707525775	2.30652367066822
H	-1.89263958551832	-1.49778284946329	2.15623495869598
H	-0.38334945543886	-2.01878142912409	1.37045319760379

Coordinates of (S,S)-14-Me, Conformer ID# 23
charge: 0, multiplicity: 1
49

C	-3.66333865544918	-2.61668537861629	0.42547227552513
C	-2.99628894267421	-1.42507182673873	-0.23137077598626
N	-1.71368452656788	-1.14954568475995	0.14135986571495
C	-1.03665705681144	0.07464446038323	-0.35344189483638
H	-1.78866280178142	0.86294333765817	-0.45491402502123
N	-0.29868495691918	-0.08798123061297	-1.60213093787197
C	-0.97116543974226	-0.59161712883027	-2.78584570122523
C	0.99636190729687	-0.50470911109559	-1.28864269724911
C	1.96865690189634	-1.06169212198185	-2.11874876269522
C	3.22791235716936	-1.32498072005171	-1.58344961934632
C	3.51913971928350	-1.04672210131555	-0.24562962287865
O	4.79462920662561	-1.35379577703984	0.16133559773077
C	5.13042509693763	-1.07659014420108	1.51622261414403
C	2.53442999787155	-0.50081828570192	0.59000045271275
C	1.28281714722663	-0.24574187269351	0.05225368488233
C	0.06130977969673	0.35291666292511	0.71580755753308
C	0.28101052552203	1.86415826451543	0.97761287471334
C	-0.92290373420244	2.58791076395043	1.50477211851554
C	-1.79069603556348	3.32781923632162	0.80010554821695
C	-2.96364826490069	3.98967701662872	1.46905365685829
C	-1.70282080644182	3.57290648407151	-0.68027747579033
C	-0.39127096499157	-0.45115180617979	1.94156695212344
C	-1.03600365565972	-1.69319087541469	1.32941661517797
O	-3.58800443868387	-0.74611644855356	-1.07235628336924
H	-3.97263986075591	-2.36497133267426	1.44668613095403
H	-3.00141214922973	-3.48622038693018	0.47832752281192
H	-4.55384219642947	-2.86580606518093	-0.15232415450563
H	-1.17183190561687	-1.67509042213585	-2.73177536921088
H	-0.35197126461586	-0.39650217545716	-3.66799856358053
H	-1.92474666212681	-0.07370549339710	-2.90542305762774
H	1.76504534044908	-1.29408227693250	-3.15930014538862
H	4.00671248068635	-1.76084800430237	-2.20219325339713
H	5.03273708979613	-0.00584726749411	1.74313615652895
H	6.17308307609528	-1.37694566355837	1.63490436570294
H	4.50541306001330	-1.65404284538416	2.21168293617733
H	2.73098688384983	-0.28661167809036	1.63562796728527
H	1.10571837581074	1.95005037501794	1.69812184469888
H	0.64057521223411	2.31192830337697	0.04494781559647
H	-1.10772744226013	2.49036289515048	2.57496808968333
H	-2.98465821732911	3.79250480467792	2.54439120454103
H	-3.90671968311684	3.63631368861308	1.03200565229162
H	-2.93572241377831	5.07636886245285	1.31522690686343
H	-1.63289087874558	4.64954389256057	-0.88293054614965
H	-2.61913255513410	3.22287364935685	-1.17404592470226
H	-0.85503635495458	3.07604556499912	-1.15617175592877
H	-1.14382975042917	0.11217562365389	2.50562450528550
H	0.44223423084022	-0.69091685441290	2.60937415364402
H	-1.74483488237115	-2.17354712292850	2.00701770343931

```
      H     -0.27398189201852 -2.43040578364784 1.03847779740851

Coordinates of (S,S)-14-Me, Conformer ID# 24
charge: 0, multiplicity: 1
49

      C     -4.59140154234591 -1.48123334583057 0.59425980827648
      C     -3.47266518448711 -0.84833600065203 -0.20375714944512
      N     -2.20640187357788 -1.09811752171144 0.23913370508221
      C     -1.05192295490464 -0.40848464317477 -0.31938878878673
      H     -1.36542527873932 0.57762919472394  -0.69315419249691
      N     -0.33413242539623 -1.15656227381921 -1.37004964341547
      C     -0.87277186339306 -1.07316368874682 -2.72072304146507
      C     1.03241955528658  -0.91081819714136 -1.17117003557235
      C     2.09346700816188  -1.11747946941695 -2.05142990439661
      C     3.38770108521175  -0.84998292185182 -1.61063639864880
      C     3.63013507229912  -0.38226743726698 -0.31652608598733
      O     4.94732884168076  -0.14949695172616 -0.00631481490014
      C     5.23266186499861  0.33824642978036  1.30011302468838
      C     2.56237613735573  -0.16733234617920 0.56586950234383
      C     1.27717924636496  -0.42691610711576 0.11709933435723
      C     -0.03653168453721 -0.33262407226701 0.84272926182477
      C     -0.22932280349833 0.91007623245215  1.73231666185684
      C     -0.08779567758968 2.20944818145892  0.99796381690713
      C     -1.07562554511278 3.01454974822794  0.58439805953572
      C     -0.77482385573086 4.27810279239781  -0.17385554359970
      C     -2.54087949506258 2.75105505577211  0.79068226264905
      C     -0.36236220751154 -1.63377490946222 1.62275330691798
      C     -1.88032362440245 -1.79715329272200 1.49672273654689
      O     -3.69557443089160 -0.15168727942375 -1.19475695002031
      H     -4.63382818919315 -1.05858507944376 1.60476507653590
      H     -4.44960670814922 -2.56214874188315 0.69674890673742
      H     -5.53409894965450 -1.28296666177714 0.08442253442056
      H     -0.37214200691242 -1.80140430300675 -3.36645069176207
      H     -0.74879677389918 -0.06725716096211 -3.15804851401968
      H     -1.93880343724624 -1.31021797682884 -2.69796669422607
      H     1.93270869750524  -1.47280201696975 -3.06444078169380
      H     4.23414509447993  -0.99302433331727 -2.27574008185492
      H     4.90969379925977  -0.37237306951556 2.07354910296103
      H     4.75511255678788  1.31171333454771  1.47863432639183
      H     6.31694856393792  0.45445356661453  1.34711762033369
      H     2.72099918109654  0.20872666369332  1.57123300969828
      H     -1.20613974544984 0.83590762199161  2.22397052577387
      H     0.52030241186670  0.85747805555543  2.53444196829170
      H     0.93461912982377  2.49673329842846  0.75094163064319
      H     -1.25654784383000 4.25970738193858  -1.16023736592951
      H     0.29975565356128  4.42148642370378  -0.31590487971215
      H     -1.17649427640183 5.15318259140016  0.35365023809158
      H     -3.01821765193841 3.60698644439284  1.28474267980107
      H     -2.74445013556785 1.85477482537507  1.38060849776006
      H     -3.04094611657764 2.62650948971601  -0.17886110404164
      H     -0.03690323709449 -1.57743258105828 2.66605668161047
      H     0.14914522660023  -2.47660981711420 1.14756913055266
      H     -2.40809276840640 -1.33181551575293 2.34048214219309
      H     -2.17327083877630 -2.85263961603294 1.45628710919147

Coordinates of (S,S)-14-Me, Conformer ID# 28
charge: 0, multiplicity: 1
49

      C     -3.61765408087867 -3.11219426891468 0.89723175591260
      C     -3.14014288969400 -2.15052276459228 -0.17213760832363
```

N	-1.98299439421728	-1.47293821686899	0.07652270929075
C	-1.50609525054953	-0.42432130305830	-0.85978883569478
H	-2.37900473565051	0.08897572404789	-1.27965582807366
N	-0.63944472240278	-0.89838676529088	-1.93400665284783
C	-1.09249037000650	-1.94925662711125	-2.82744424960801
C	0.67485667561850	-0.88796681150717	-1.46266505450415
C	1.79938936593629	-1.52807166718944	-1.98244188629526
C	3.02855201528798	-1.32384245244393	-1.35887853225421
C	3.13927537610643	-0.51040524447633	-0.22883985280977
O	4.40152855950838	-0.39447465657211	0.30100802817453
C	4.55271927806480	0.44146489453572	1.44279365122621
C	2.00373966816572	0.11776340461537	0.30101971776426
C	0.78557309338427	-0.07700259085177	-0.33214742106490
C	-0.58181277472078	0.47014569896365	0.02280490300245
C	-0.73738541311908	1.96691767552384	-0.33348608688339
C	0.19216531266276	2.86283199109955	0.42783848252839
C	-0.09922071118947	3.64841845618585	1.47315663893304
C	0.96971474274029	4.47665183111521	2.13350951997355
C	-1.46100730954306	3.79303436960096	2.09237768306820
C	-0.98923133873574	0.13238609774235	1.46247408480282
C	-1.33110042052449	-1.35392144608417	1.39141212915467
O	-3.76852177964233	-1.99948252476800	-1.22105856120658
H	-4.07492972187724	-2.56184793846766	1.72785450133365
H	-2.80359420187058	-3.72001336316831	1.30321207597623
H	-4.37564379345001	-3.75921593486246	0.45484240964099
H	-0.44287463233814	-1.98314212577261	-3.70854870892641
H	-2.11169576971347	-1.73166021227342	-3.15198734470797
H	-1.08724924499353	-2.94393328005826	-2.35025218819891
H	1.73515878552130	-2.17933987219296	-2.84834424782674
H	3.92339184475484	-1.80855317360279	-1.73792242832976
H	5.61470815851756	0.41818334068585	1.69398143575346
H	3.96985213535113	0.06716194964737	2.29591683373505
H	4.25286783005287	1.47632433585836	1.22535933683741
H	2.05886144503372	0.73859504494793	1.18852862412882
H	-0.54171679515365	2.07317137759158	-1.40942573566567
H	-1.78536218660136	2.24250634301559	-0.16945859524957
H	1.22661292338288	2.85270996590618	0.08453818173214
H	1.94620380874278	4.33004455796235	1.66349323647638
H	1.05479694591301	4.22371153486814	3.19880390417591
H	0.71966995543287	5.54469017657394	2.08637564896840
H	-1.44527830140330	3.43344317543356	3.12993735209075
H	-2.24166116678201	3.24811712750883	1.55882842956210
H	-1.74829701214042	4.85145306501718	2.13521933776336
H	-1.88109411477134	0.70758577794716	1.74207506561686
H	-0.19626614410322	0.35381081262464	2.18248573178978
H	-1.99892562685116	-1.66833624925670	2.19605900185478
H	-0.42062301725475	-1.96896923963458	1.43082940720365

Coordinates of (S,S)-14-Me, Conformer ID# 29
charge: 0, multiplicity: 1
49

C	-3.89206023912439	-3.09111271937377	0.08572399743968
C	-3.24097275546172	-1.78330633639867	-0.31276943602327
N	-2.09454937304098	-1.44843525541866	0.33277899660657
C	-1.35843100198385	-0.22313156969248	-0.01235138929971
H	-2.06626176370248	0.61035622640465	-0.08513229395087
N	-0.59912565122351	-0.34951879456767	-1.26077767483658
C	-1.04015434094019	0.40712617685782	-2.42624040365172
C	0.76236395605147	-0.41305185841120	-0.95415066166739
C	1.83872035752979	-0.53434582869235	-1.82548561378518
C	3.13354742188042	-0.62995309172732	-1.29937227796971

C	3.34237378366253	-0.60449408691046	0.08017432307450
O	4.57100984565675	-0.70270594268838	0.68465050673823
C	5.70605911382529	-0.84408960879314	-0.16368974688719
C	2.25450091779128	-0.45426765491745	0.95332151495158
C	0.98399219891309	-0.35257851862079	0.42696757395022
C	-0.31682266473791	-0.06930069636302	1.14074606919664
C	-0.32391866360886	1.35479671398438	1.74597345863609
C	-0.01008581405087	2.41728518336474	0.73612937685938
C	-0.85629869451678	3.29831244216703	0.18635492091549
C	-0.37726734943429	4.27924112160858	-0.84909627041498
C	-2.32066660825917	3.41086260948123	0.50654699013977
C	-0.70144096513818	-1.14351234797433	2.17533820341396
C	-1.38462894715976	-2.22985024228823	1.34800451893129
O	-3.73325136724383	-1.05030756635306	-1.17453605310016
H	-4.74528117148813	-3.26231732577357	-0.57081530806468
H	-4.24271204063416	-3.05341757095154	1.12320854516996
H	-3.19139251452223	-3.92843593624753	0.00103598335458
H	-0.61353478237303	-0.02467623135554	-3.33753209016256
H	-0.74075521367182	1.46801728017214	-2.36877064925663
H	-2.12768752603056	0.33638110257812	-2.49821364658866
H	1.69555744693566	-0.56410996540666	-2.90124897443189
H	3.96582097683011	-0.72866802620037	-1.98676486692977
H	5.82032370344854	0.02314858882091	-0.82848105345283
H	5.64320731355190	-1.75946535152475	-0.76787157295159
H	6.57037301435002	-0.90715960179062	0.49990568083405
H	2.43873066013756	-0.39699230842930	2.02356205939064
H	-1.29946719938947	1.51864740873179	2.21672918834485
H	0.42674512150557	1.37666872322669	2.54934837638892
H	1.02494421062889	2.43538026729117	0.39339745901004
H	-0.53571590832499	5.31166750957120	-0.51038872260440
H	-0.94688302052371	4.16629507587131	-1.78115892846290
H	0.68447030180616	4.14698677468649	-1.07453316597165
H	-2.56272765140727	4.43322888481001	0.82449103211949
H	-2.65342150570962	2.72348604587203	1.28639440698068
H	-2.91886425878544	3.21573125489029	-0.39375105417482
H	-1.41600802936250	-0.72483466989552	2.89480215116577
H	0.16758848710474	-1.51477707121906	2.72656802075641
H	-2.07377213408482	-2.83861640500406	1.93839141168087
H	-0.64298967567522	-2.89672680740112	0.88055708858947

Coordinates of (S,S)-14-Me, Conformer ID# 31
charge: 0, multiplicity: 1
49

C	-4.54101554581846	-0.65412466346830	0.14599871814987
C	-3.29997878770179	-0.60307772855172	-0.71695673397987
N	-2.10793650076065	-0.66343940258242	-0.05656545542614
C	-0.84979616969409	-0.41649523376509	-0.74021726268308
H	-1.04408872953986	0.18930423424033	-1.63961061040070
N	-0.10763701417596	-1.64223855712141	-1.08638022919017
C	-0.51859103402636	-2.34629780539494	-2.29098622132396
C	1.24972130186758	-1.37392477392235	-0.87569333829589
C	2.36010288030765	-2.06508721878222	-1.33929009316181
C	3.63844237167559	-1.63727025465402	-0.94906063440708
C	3.79220273162357	-0.53127573895417	-0.11535340564738
O	5.00000909402144	-0.03703966344860	0.31036609905919
C	6.17325092858790	-0.71138620771296	-0.13259520945974
C	2.66477200676833	0.17335224085120	0.34034119998285
C	1.41369756065407	-0.25046351810749	-0.04845659211249
C	0.05107693725312	0.28568806386337	0.30326254935352
C	-0.04525307604306	1.82331884121676	0.25730629895352
C	-1.44504273524531	2.35041290173753	0.37495813280401

C	-2.01185114204225	2.95640934021116	1.42609831983061
C	-3.44996904508608	3.39633515687110	1.37414844993123
C	-1.32560405256305	3.24337687705076	2.73198695037706
C	-0.47542575253878	-0.31324253487176	1.63718584518557
C	-1.96280022550438	-0.61869169528218	1.40982667056466
O	-3.36647921040941	-0.50471412679489	-1.94332319945090
H	-5.40940337399350	-0.76403890556294	-0.50356708875011
H	-4.63938681727575	0.27654358111314	0.71799119256269
H	-4.50773443069817	-1.47978353752254	0.86383348787342
H	-0.05270044933233	-3.33643154323384	-2.32209546107159
H	-0.24608575024565	-1.79778684455561	-3.20932290815440
H	-1.60334615959876	-2.47836399589284	-2.27664614587203
H	2.26239452340586	-2.92283266705660	-1.99733861808639
H	4.50048982674763	-2.17972810279830	-1.31960353918593
H	6.26175831686968	-0.68456848843788	-1.22734869078465
H	6.18757846061388	-1.75592788608339	0.20768433440627
H	7.01354346091633	-0.17462954499521	0.31140025529052
H	2.80673787765454	1.04211469082621	0.97764938099373
H	0.60068912303221	2.22506896715593	1.04505366129096
H	0.38312807217537	2.15102871133390	-0.69979306694172
H	-2.07010425036385	2.20045308845362	-0.50727228712623
H	-3.91297302733864	3.16090579762186	0.41169643786058
H	-3.53509893393838	4.47744220886861	1.54525143404011
H	-4.03264155351304	2.90993913420538	2.16857730608665
H	-0.26774302540837	2.97485681675180	2.73684397785660
H	-1.81854651092334	2.69605404413662	3.54696658651675
H	-1.41048366256374	4.30916038115888	2.98002669980582
H	-0.32543762700011	0.37654871689092	2.47279502440347
H	0.07183941293280	-1.23500900280383	1.85544541514428
H	-2.59860036696025	0.17215737033912	1.82446079378926
H	-2.25197992680419	-1.57383152254068	1.86527156939908

Coordinates of (S,S)-14-Me, Conformer ID# 32
charge: 0, multiplicity: 1
49

C	-3.85835034128960	-3.09186282834318	0.21440939985387
C	-3.23197819034785	-1.78165032862910	-0.21462945159051
N	-2.04571457680001	-1.45340136506952	0.35903852343782
C	-1.32817576290889	-0.23093898697217	-0.02859575889045
H	-2.03435220069571	0.60684203669800	-0.05187099315530
N	-0.65534655357758	-0.35776291766499	-1.32795521062821
C	-1.15988907175940	0.43901478917853	-2.44166878677736
C	0.72457138379340	-0.42229949687097	-1.10462358061817
C	1.74387110462970	-0.55062956029456	-2.05046727569683
C	3.05892546403260	-0.64332925887523	-1.60778388963292
C	3.36708286379743	-0.61237652862816	-0.24333641029833
O	4.69976953090928	-0.72536237593673	0.06562541949082
C	5.05463533635127	-0.67912763302021	1.44334811868419
C	2.34640096466804	-0.46391589780654	0.70289004379549
C	1.03744938426821	-0.36220217577977	0.25135646586377
C	-0.21152245102501	-0.08603678801049	1.05413573540761
C	-0.17995634191927	1.33024815319598	1.67657508248389
C	0.07104892381959	2.40568053183119	0.66318627274847
C	-0.80623330373745	3.29629902990022	0.18170644900525
C	-0.39231846906146	4.29026144172853	-0.86923683128356
C	-2.24660562378112	3.40703440206433	0.59726331802630
C	-0.52582366412030	-1.17187656150427	2.10141893417043
C	-1.26941498087452	-2.24769217535853	1.31353838740133
O	-3.77697495057097	-1.04080098667136	-1.03670384669720
H	-4.75536128804264	-3.25409816934860	-0.38339744723184
H	-4.13608875489862	-3.06477616280581	1.27404275715510

H	-3.16760726122286	-3.92969923334062	0.07221009025867
H	-0.87691222967940	1.50235572068524	-2.35354899736804
H	-2.24833271124797	0.35148063143063	-2.46992682665650
H	-0.76514537550947	0.05208728503008	-3.38639946172069
H	1.52837771216366	-0.58530261817366	-3.11400639696410
H	3.87269324567852	-0.74747970289180	-2.31936225265037
H	6.14006512484849	-0.78861705838963	1.47796128903889
H	4.58838794971169	-1.50026524094833	2.00542839632754
H	4.77152432676646	0.27994370040054	1.89889136717747
H	2.55944934482020	-0.40394846020924	1.76562755271053
H	-1.12379797855776	1.48775512021474	2.20996854837213
H	0.61895977523627	1.34248467180505	2.43256654439775
H	1.08139608889874	2.42546742645085	0.25330365058008
H	0.65178163121284	4.15801810838571	-1.16579418784953
H	-0.52424602197976	5.31823550696075	-0.50647651504739
H	-1.02231440221947	4.19227844786667	-1.76335607612556
H	-2.46513456276796	4.42550010689672	0.94350418231077
H	-2.53034899180418	2.70985996705832	1.38772434523861
H	-2.90186787092258	3.22491794250744	-0.26522939103734
H	-1.18585202041151	-0.76009715780916	2.87500532257793
H	0.37891841420556	-1.55189429565118	2.58553457628527
H	-1.91783087752092	-2.85994426523586	1.94510106773248
H	-0.56533174055775	-2.91222679004986	0.78822774738772

Coordinates of (S,S)-14-Me, Conformer ID# 33
charge: 0, multiplicity: 1
49

C	-3.52138646736873	-3.78043019527537	-0.15253561685600
C	-3.12376967446984	-2.37768173830384	-0.56102589195758
N	-2.20293792112968	-1.75172505878317	0.21498985666800
C	-1.72212152680884	-0.39956989114234	-0.12563687236006
H	-2.58858456725341	0.24553903715489	-0.31259298155113
N	-0.82123156061707	-0.38818191706523	-1.27724721030192
C	-1.23837461905169	0.20121469114530	-2.53989598490739
C	0.48922630819701	-0.24874008339404	-0.82677041915564
C	1.66461971182962	-0.22686089027588	-1.56805654810853
C	2.89066478170000	-0.13463032428267	-0.89434979449219
C	2.93178466376699	-0.06863903896643	0.49839603715147
O	4.08350362704092	0.01421958291340	1.24268709955690
C	5.31827363387788	0.02036719498519	0.53377428603556
C	1.73941640334752	-0.05701397809611	1.23773102363776
C	0.53775433565811	-0.12994325131808	0.56821465716975
C	-0.86516099915679	0.01058468864480	1.11181033848216
C	-1.12717684646958	1.47071581492639	1.56368900901633
C	-1.02767770343312	2.46320492132961	0.44283545460845
C	0.00308606502086	3.26056600962616	0.13492450383634
C	-0.05100249140481	4.15018660348306	-1.07708112044587
C	1.28878024057987	3.34671749678603	0.90554068861253
C	-1.21467011699404	-1.01908982825705	2.19670637179472
C	-1.54195468409906	-2.28567653150806	1.40883554586069
O	-3.61798916381071	-1.82351146691481	-1.54642609224397
H	-2.64554381682200	-4.42614847224384	-0.02984886618871
H	-4.17270925954497	-4.19049742396982	-0.92459118956470
H	-4.06310159735666	-3.76903482057275	0.80004468619020
H	-2.30190486968505	0.00109254460218	-2.68522081284301
H	-0.69682438860337	-0.26386738360728	-3.37139005652978
H	-1.05450695734031	1.28885302928720	-2.57209528403324
H	1.65204219487280	-0.28986640868771	-2.65185528537445
H	3.80311438715479	-0.12328386957680	-1.47945624043555
H	6.09989745201870	0.08735755909369	1.29273372213366
H	5.38991074464737	0.88585571758330	-0.13941084552407

```
H     5.45204605809929  -0.90296275765086 -0.04642190154304
H     1.78918004523511  0.04483039163587  2.31943022583370
H     -2.13973358776055 1.50751304505465  1.98991737152994
H     -0.42823662594651 1.70190630373164  2.37562771455963
H     -1.89437224559242 2.50035065530373  -0.21905488528630
H     -1.00117924014174 4.05718935218450  -1.61041725031150
H     0.76178022688848  3.89777524184218  -1.77146477952287
H     0.09097170371152  5.20240772294308  -0.79791456234223
H     1.52539109407393  4.39408585247280  1.13305404248379
H     2.11625655615486  2.95769640051516  0.29708180311948
H     1.27372453591980  2.78067248202051  1.83792419286741
H     -2.10202853365713 -0.68798543482908 2.75030057370735
H     -0.39704160878682 -1.16526289562791 2.90895187623700
H     -2.19986021983199 -2.96349285096634 1.95904098835908
H     -0.62419347665853 -2.83155582794984 1.13972842242783

Coordinates of (S,S)-14-Me, Conformer ID# 34
charge: 0, multiplicity: 1
49

C     -4.49146052796230 -0.74931746288676 0.45613465545552
C     -3.28292444018980 -0.39877244565219 -0.38304145085633
N     -2.06928197977492 -0.72573975712224 0.14685869083147
C     -0.83445512627393 -0.24498094317499 -0.44935684885773
H     -1.05716937287647 0.64495554374591  -1.05709014016196
N     -0.12598373367216 -1.26228753935147 -1.24704103574540
C     -0.59583041022550 -1.47982090058510 -2.60587487733421
C     1.24228721583488  -1.09260847198103 -1.00972941595982
C     2.32208868913498  -1.57150907337201 -1.73820006114481
C     3.62166827135951  -1.31867423437936 -1.27289845667871
C     3.82622396864651  -0.59200256061505 -0.10153981817370
O     5.05782892412955  -0.29108861487531 0.42489756038107
C     6.20132882735351  -0.75986458516668 -0.28213795535515
C     2.72956249043719  -0.09722761520143 0.62480821463178
C     1.45704478652624  -0.34675886797201 0.16129088738564
C     0.11855623968157  0.03490382504783  0.73752644738801
C     0.05569191064172  1.49542251174778  1.23016879694415
C     -1.30927545679288 1.93782346024875  1.67020489007146
C     -2.17939360767021 2.69220966664713  0.98529975181992
C     -3.54020735373445 2.99980312641422  1.54779021100305
C     -1.93282211357091 3.27919846317443  -0.37554892125181
C     -0.36945821843151 -0.99727993150503 1.79098329151619
C     -1.86934065626262 -1.19828540421057 1.52839767606176
O     -3.39556802830572 0.14869291754745  -1.48153657773887
H     -4.45244817973755 -1.78020324927710 0.82184855953344
H     -5.38619014331845 -0.60600069337493 -0.14985539469520
H     -4.54556271339216 -0.09009093174240 1.33073651276664
H     -0.34991367734446 -0.63589707852998 -3.27370439962839
H     -1.68153622448608 -1.60537983493718 -2.59354450510335
H     -0.14996795283117 -2.39232214708119 -3.01448768357679
H     2.18409528466077  -2.13129181087865 -2.65793859526523
H     4.45948966137773  -1.69215450120686 -1.85019497277293
H     7.06721963983560  -0.42390339757001 0.29119933333552
H     6.24823841599657  -0.33573751131552 -1.29454037412354
H     6.21066930791256  -1.85658579511359 -0.34683684859015
H     2.91139893800008  0.47995218740117  1.52744670849623
H     0.75502173903028  1.58768630633263  2.07262503286968
H     0.44231823123396  2.13497475782031  0.43021649002162
H     -1.62762218352264 1.57683781906815  2.64947258063928
H     -3.69137294652321 2.54080891110487  2.52928694460304
H     -4.32300020725984 2.63718156534689  0.86772907798781
H     -3.68877320449992 4.08317468820667  1.64436260189670
```

```
      H     -2.61122748012278 2.82061741315709  -1.10670277194058
      H     -0.91029957493975 3.13998581202550  -0.73159786562616
      H     -2.15141306421053 4.35463577597551  -0.36799668033465
      H     -0.17441475972036 -0.65590444905340 2.81229600138406
      H     0.16804888180344  -1.93850398136619 1.64272614677507
      H     -2.48135270006033 -0.60706010198026 2.21961518538415
      H     -2.15711538588405 -2.25154085953384 1.63338340173224

Coordinates of (S,S)-14-Me, Conformer ID# 38
charge: 0, multiplicity: 1
49

      C     -3.60465694163252 -3.65721002272376 0.04362126197908
      C     -3.20118982296604 -2.25394294766149 -0.35712627832120
      N     -2.14921331617480 -1.70708479796482 0.30408777580990
      C     -1.65087242333323 -0.36615680083480 -0.04957096216031
      H     -2.49937498606747 0.32685848955495  -0.08765711500734
      N     -0.92392842618643 -0.34961879011745 -1.32092621280317
      C     -1.48251646608981 0.35509418903416  -2.46644585373212
      C     0.44311224070165  -0.27858328328031 -1.04986868619841
      C     1.50579200564836  -0.28027989604333 -1.95524263354424
      C     2.80541040388613  -0.26024477781386 -1.45809088720091
      C     3.05380685584218  -0.24366405358554 -0.08193527796293
      O     4.37844174120377  -0.23728513294204 0.28219916673755
      C     4.66892277993400  -0.16726827343155 1.67354058054606
      C     1.98664936087278  -0.21617992809604 0.82319023826115
      C     0.69433711478322  -0.21623728737970 0.31964956388540
      C     -0.60781907220235 -0.05028460491172 1.06778447763365
      C     -0.73984933366282 1.39134025398578  1.62411549505014
      C     -0.75117386986835 2.44091520267672  0.55239458726537
      C     0.26155392952317  3.21656015691576  0.14502418101824
      C     0.08207082158424  4.17371460572065  -1.00150693889181
      C     1.64273624887352  3.21284585552962  0.73401540089005
      C     -0.84304003510762 -1.12184256685417 2.14429866623239
      C     -1.34527085630916 -2.33063180218923 1.35808612368655
      O     -3.80348419975648 -1.63047028051457 -1.23487211675801
      H     -4.38164274403473 -3.99877602865332 -0.64050825650345
      H     -3.99976868412295 -3.67160708486009 1.06570327928256
      H     -2.75376280705215 -4.34545288125938 0.00564022285044
      H     -1.05873333987959 -0.04276000662929 -3.39483024016534
      H     -1.28084487639899 1.43951602545095  -2.43014030351183
      H     -2.56024155931534 0.18046691073184  -2.49233111544562
      H     1.33699935546056  -0.30456943577928 -3.02747406672004
      H     3.65336555444763  -0.26432503702949 -2.13668260619981
      H     4.27046219694974  0.75437122739836  2.12060702830904
      H     5.75748857567128  -0.16585439688889 1.75397638110584
      H     4.26628393877231  -1.03614528590608 2.21265754603042
      H     2.15141859238480  -0.15916642158842 1.89468018335636
      H     -1.68101889983446 1.44238216142355  2.18971543529485
      H     0.07364225669475  1.55073860591860  2.34132415054416
      H     -1.69780852376433 2.54676097495052  0.02023913256364
      H     -0.93553203447840 4.14654291818679  -1.40101645022610
      H     0.77954402826312  3.93003857966207  -1.81425289877748
      H     0.30824564410486  5.20258913302409  -0.69252783819288
      H     1.72652191028400  2.60148021764786  1.63388565729866
      H     1.95622228195648  4.23703565951287  0.97358789033167
      H     2.36119049107367  2.82225905951176  0.00082854508523
      H     -1.62058110574513 -0.78450911173081 2.84083225874975
      H     0.06318942966461  -1.33972972740697 2.71776567420101
      H     -1.94651353876419 -3.00826538139558 1.96993959123499
      H     -0.50589989583348 -2.89956418136490 0.92849624308888
```

```
Coordinates of (S,S)-14-Me, Conformer ID# 39
charge: 0, multiplicity: 1
49

      C     -4.51530730016951 -0.57002779601792 0.24794622210426
      C     -3.30503511541039 -0.55920548088244 -0.65901700666219
      N     -2.09277686303974 -0.66396678889083 -0.04241594533283
      C     -0.85240728933620 -0.45799751894940 -0.77081749779185
      H     -1.05814903228882 0.15891600666476  -1.66014412692615
      N     -0.16691887403545 -1.70671599534357 -1.15120758480952
      C     -0.63869480170981 -2.37287042597781 -2.35613245562714
      C     1.20582433851833  -1.48330223728440 -0.98324753776586
      C     2.27801569871814  -2.22016533956703 -1.48361357184865
      C     3.57111130114528  -1.83843300320856 -1.13239131987604
      C     3.80075347389594  -0.74028733802994 -0.29992921700391
      O     5.11880456001030  -0.46215728025032 -0.03410884641748
      C     5.39345158166893  0.65409184426618  0.80518855562022
      C     2.72209342688753  0.00598814221033  0.19582009523697
      C     1.43888028468917  -0.37548450882470 -0.16346181976278
      C     0.11022101547722  0.20753063560539  0.24112580104557
      C     0.06581653884554  1.74808407347492  0.20353374586616
      C     -1.30956860921894 2.32338426369609  0.37207635845575
      C     -1.81863378861011 2.94190872291755  1.44524175336094
      C     -3.24168312154904 3.43117698104350  1.44552541619174
      C     -1.07837907954717 3.19682475921736  2.72808092736348
      C     -0.38900022161625 -0.37747755594508 1.59205514576940
      C     -1.89341799101570 -0.63038821306719 1.41774560537564
      O     -3.41217128487338 -0.45254221661119 -1.88179945552397
      H     -5.40946357313795 -0.65377686011549 -0.36979105576933
      H     -4.56336737498037 0.36531224067269  0.81889138139286
      H     -4.48272676987387 -1.39403259917351 0.96759833719852
      H     -0.20314338818038 -3.37468306321892 -2.42420522156794
      H     -0.38282242092538 -1.81211106487596 -3.27183140675679
      H     -1.72567308138380 -2.47488234648992 -2.30662395374427
      H     2.12683700985411  -3.07311282522406 -2.13780446337408
      H     4.42742626767091  -2.38803511800717 -1.51166416419262
      H     5.02409642907228  1.58979768847762  0.36303160807179
      H     6.48020440738011  0.70214871477047  0.89476644475743
      H     4.95221192413773  0.52441019139124  1.80318611391420
      H     2.87451262232612  0.86975189271208  0.83409909214036
      H     0.75199021026299  2.12428240900633  0.96987452756417
      H     0.47155376932308  2.06496860965666  -0.76703458617837
      H     -1.96903353843652 2.20018413105033  -0.48894207296151
      H     -3.74506902189542 3.21823117079639  0.49826005665636
      H     -3.28404466664379 4.51325797505545  1.62601697094828
      H     -3.81309838389058 2.95896463561954  2.25652425916026
      H     -1.11592354883709 4.26365585151613  2.98329673184549
      H     -0.03143677107495 2.88995828966000  2.69574656119313
      H     -1.56290851159953 2.66340770637642  3.55718546983415
      H     -0.18563749453340 0.30270636883848  2.42435222591010
      H     0.13311862812603  -1.31883231030066 1.78729960395255
      H     -2.48630178148354 0.18007015307636  1.85737265196589
      H     -2.19865978871264 -1.57694557151620 1.88058164699762

Coordinates of (S,S)-14-Me, Conformer ID# 40
charge: 0, multiplicity: 1
49

      C     -4.44069502749486 -0.71326382651206 0.59847743106437
      C     -3.27114937987280 -0.37608212131816 -0.29968208714203
      N     -2.03776318806565 -0.73063627233930 0.16278563984246
      C     -0.82698151771959 -0.26999663362648 -0.49560967421569
```

H	-1.06403403813149	0.62842548367038	-1.08533205159327
N	-0.18216394497450	-1.29363277952457	-1.33847505707248
C	-0.71989118442671	-1.46964047494084	-2.67902960488399
C	1.20016830015969	-1.15171545697409	-1.16508316530525
C	2.23493520176799	-1.65907022926322	-1.94944070460971
C	3.54934994147206	-1.43837639066507	-1.54382664517033
C	3.83672045511442	-0.72268290336239	-0.37897212387482
O	5.17036067479505	-0.56848232708305	-0.09080674464633
C	5.50344504356885	0.15875756974855	1.08665023881063
C	2.79574402056584	-0.20414266175440	0.40433490059289
C	1.49060159467142	-0.42122373982063	-0.00917861180990
C	0.19295313280837	-0.01593768407512	0.64028166047096
C	0.18345635551234	1.44319092247410	1.14180714264846
C	-1.14777298445956	1.90854404086245	1.65559576376307
C	-2.03852705907735	2.68241007870306	1.02078292276608
C	-3.36220051346876	3.01124843172046	1.65514666683051
C	-1.85212490053107	3.27270236756036	-0.34826138127950
C	-0.25963237532719	-1.04353480093305	1.71441984568112
C	-1.77461558473291	-1.21568342097486	1.52924440939329
O	-3.43102691232863	0.18377613745231	-1.38590692461587
H	-4.44001213075424	-0.05910981646387	1.47855270030735
H	-4.39945515162529	-1.74706212690515	0.95566539745659
H	-5.36286073939005	-0.55190557906634	0.03992646963412
H	-0.30754046584755	-2.37680007420060	-3.13184556037260
H	-0.49520652019394	-0.61162179431009	-3.33630432040399
H	-1.80543474986449	-1.58174313623476	-2.61710864355071
H	2.03827466353671	-2.21289329057653	-2.86218758164742
H	4.37725794972977	-1.81518642332975	-2.13701673024779
H	5.10090349420934	-0.32737935159235	1.98619364894001
H	5.13649205486958	1.19333400377850	1.03723917074771
H	6.59377500460873	0.16493330394707	1.13721913723278
H	2.99304206085424	0.36528142332298	1.30631361897069
H	0.92764360711555	1.51880790370115	1.94704659761878
H	0.53885255127085	2.07959680053306	0.32492965501099
H	-1.42098218794643	1.54728613904796	2.64835737867644
H	-3.48623965344027	4.09664637837247	1.76325729261042
H	-3.46971550915252	2.55042456585662	2.64154730856206
H	-4.18594132811712	2.66558346738939	1.01582543017775
H	-0.85432222746295	3.11092657165542	-0.76044655714569
H	-2.04352926128465	4.35309767576617	-0.32289721162215
H	-2.58034548239696	2.83553505462713	-1.04371036048371
H	-0.00650442180459	-0.71080477288366	2.72595919298269
H	0.25183947134408	-1.99382430151078	1.53471735663879
H	-2.33880236183718	-0.61799720585543	2.25484577318947
H	-2.07564477624557	-2.26435872409301	1.64327899107275

Coordinates of (S,S)-14-Me, Conformer ID# 41
charge: 0, multiplicity: 1
49

C	-4.40960606220246	-0.85515832512480	0.59216299858848
C	-3.30999977265486	-0.54948046142627	-0.40288769333654
N	-2.03200394393588	-0.79014134371343	0.00289868574591
C	-0.87821870679630	-0.40194112872487	-0.78847001610567
H	-1.20288886171572	0.28596703177110	-1.58097009378556
N	-0.17471639507403	-1.56419915697589	-1.35239983469330
C	-0.65331273544774	-2.05519885199640	-2.63457480606408
C	1.19525260426967	-1.38552834475281	-1.13670964274721
C	2.26215481169304	-2.08493021486918	-1.70039640272747
C	3.55525533030135	-1.77772990982124	-1.28453422244384
C	3.79030430382115	-0.78982552906759	-0.32457025125722
O	5.10775515361787	-0.57920932716428	0.00026830098089

C	5.38737988072254	0.42428218591495	0.97026874316883
C	2.71763288989958	-0.07897763340012	0.23055359238265
C	1.43288011643221	-0.38374846217711	-0.19417128087616
C	0.12092208564188	0.22597054832065	0.23023010539242
C	0.12805187489331	1.77200475247340	0.16379447857026
C	-1.23537845494066	2.37169688587915	0.33598843523201
C	-1.70466465257952	3.07691089258775	1.37395903879570
C	-3.12230587260932	3.58188646719989	1.37833402321275
C	-0.91624505886382	3.43913246341489	2.60157782604991
C	-0.38116084498838	-0.27902611635189	1.61768883724587
C	-1.57244201682795	-1.21033141149141	1.32932315748128
O	-3.56471487708158	-0.10057181911359	-1.52220901563424
H	-4.33085829929449	-1.87144346706040	0.99061974966649
H	-5.36719740033960	-0.73528890918630	0.08531621338467
H	-4.36613830663577	-0.15754071820474	1.43678255390495
H	-0.24054799652290	-3.04851388851726	-2.83739869728393
H	-0.38436679419171	-1.38385323535968	-3.46866783757993
H	-1.74318289724788	-2.14383269594922	-2.59740815204215
H	2.10482757112382	-2.85564247831327	-2.44861876013560
H	4.40709731629669	-2.30216932891533	-1.70708443144227
H	5.05131136229001	1.41439070303763	0.63236405818770
H	6.47236251623189	0.43293542844917	1.08912470898247
H	4.91804067021339	0.19097813329500	1.93611112143894
H	2.87458635054391	0.70317926897541	0.96601863464916
H	0.83147075671534	2.14293628998672	0.91693843560840
H	0.53340916242524	2.05988568886495	-0.81599887916144
H	-1.92842202288800	2.18701662736579	-0.48648702923749
H	-3.66141264034376	3.28571690332776	0.47419383078261
H	-3.14637980174817	4.67693799033601	1.45531386607228
H	-3.66786233942764	3.19676997104035	2.25011541030136
H	-1.40977912589670	3.04433358043564	3.49950893681927
H	-0.88266063765103	4.52978192342336	2.72209217548847
H	0.10972654159570	3.06674848473563	2.58726168866676
H	-0.71684576456168	0.57494939886455	2.21432714996550
H	0.41157803247933	-0.79512588056081	2.16549301757037
H	-2.36630694202103	-1.11773319966909	2.07228674442220
H	-1.25094010671933	-2.26038978179277	1.29364052779553

Coordinates of (S,S)-14-Me, Conformer ID# 47
charge: 0, multiplicity: 1
49

C	-4.69005262694518	-2.21522916571049	0.42311747324421
C	-3.63942731091780	-1.49216666955796	-0.39084228139662
N	-2.38128367576277	-1.47341612609804	0.13818258941349
C	-1.31682405333348	-0.67326586246320	-0.45285766480571
H	-1.76748216575475	0.18802957240013	-0.96754799863712
N	-0.43176877428972	-1.41328068534594	-1.37217178710405
C	-0.89116178566883	-1.57517027145423	-2.74476201845649
C	0.87086157910949	-0.94641864866390	-1.14348953583776
C	2.00405180646693	-1.10114223341694	-1.94050118509236
C	3.21989814475092	-0.60641948361869	-1.47398636765834
C	3.31329513127321	0.03339906953454	-0.23554130833962
O	4.56618502145407	0.47812921967026	0.10998031519147
C	4.70524571818953	1.11507726097155	1.37482622552084
C	2.17139443731237	0.19868318402734	0.55997230015977
C	0.96352781552219	-0.28586007228458	0.08438486710043
C	-0.39280871104072	-0.30077511531955	0.73309817613355
C	-0.80520586842505	0.99239542785743	1.46109654072680
C	-0.75030577145659	2.22489404964961	0.61024284021003
C	0.02967731746225	3.30146481440973	0.76866580637943
C	-0.04113135824841	4.44980791971957	-0.20101026933037

C	1.03187332766692	3.49405011562611	1.87133946925840
C	-0.56926792654619	-1.53883783320396	1.65459737220866
C	-2.03270503311029	-1.95484241236022	1.48749188210418
O	-3.90514187589089	-0.95725478166502	-1.46770354743786
H	-5.61741329964603	-2.23449460405700	-0.14918366203301
H	-4.86589050317821	-1.70081539081790	1.37484173574480
H	-4.38103957596525	-3.23958609802666	0.65600155589848
H	-1.91022414124339	-1.96821441639254	-2.73936596481907
H	-0.24928528129531	-2.28985143145806	-3.26922049483630
H	-0.88818440978684	-0.62194776583261	-3.30136203381783
H	1.95766493953294	-1.59120130808631	-2.90797156882073
H	4.11938618879416	-0.70589425473321	-2.07425208847825
H	4.09808692848720	2.02903810954800	1.43418019102573
H	5.76097685579127	1.37749864500774	1.46487686620805
H	4.42659573898304	0.44078759743305	2.19667651461756
H	2.21362984976717	0.70673502206189	1.51722032922952
H	-1.83275198129245	0.85326427938799	1.83266201416683
H	-0.17142730704600	1.09177435011634	2.34861745955374
H	-1.43353065543868	2.23535335431880	-0.23943626640391
H	0.93538725385436	4.61343395362013	-0.67568344681482
H	-0.29830224524274	5.38318827692157	0.31679844715280
H	-0.78078384268092	4.27436710716708	-0.98672676474987
H	1.06445896944151	2.66858002196974	2.58464634495046
H	0.81189701116231	4.41506563522568	2.42667070560642
H	2.03670227156572	3.61939854257130	1.44665096232880
H	-0.31854783380693	-1.31234564610902	2.69532915492749
H	0.09160734630427	-2.33939335938081	1.30823637516070
H	-2.67633462532729	-1.48062842333689	2.24108605533857
H	-2.15832101355112	-3.04057346982186	1.57065568530888

Additional references

[1] P. Schneider, B. Henßen, B. Paschold, B. P. Chapple, M. Schatton, F. P. Seebeck, T. Classen, J. Pietruszka, "Biocatalytic C3-Indole Methylation—A Useful Tool for the Natural-Product-Inspired Stereoselective Synthesis of Pyrroloindoles" *Angew. Chem. Int. Ed.* **2021**, *60*, 23412–23418.
[2] D. A. Amariei, M. Haase, M. K. T. Klischan, M. Wäscher, J. Pietruszka, "High-Throughput Colorimetric Detection and Quantification of Indoles and Pyrroloindoles for Enzymatic Activity Determination" *ChemCatChem* **2024**, *16*, e202400052.
[3] C. O. Wilke, *cowplot: Streamlined Plot Theme and Plot Annotations for "ggplot2,"* **2020**.
[4] H. Wickham, R. François, L. Henry, K. Müller, D. Vaughan, *dplyr: A Grammar of Data Manipulation*, **2023**.
[5] H. Wickham, *forcats: Tools for Working with Categorical Variables (Factors)*, **2023**.
[6] H. Wickham, *ggplot2: Elegant Graphics for Data Analysis*, Springer-Verlag New York, **2016**.
[7] K. Slowikowski, *ggrepel: Automatically Position Non-Overlapping Text Labels with "ggplot2,"* **2024**.
[8] C. O. Wilke, B. M. Wiernik, *ggtext: Improved Text Rendering Support for "ggplot2,"* **2022**.
[9] B. Auguie, *gridExtra: Miscellaneous Functions for "Grid" Graphics*, **2017**.
[10] J. Ooms, *magick: Advanced Graphics and Image-Processing in R*, **2024**.
[11] T. L. Pedersen, *patchwork: The Composer of Plots*, **2024**.
[12] R Core Team, *R: A Language and Environment for Statistical Computing*, R Foundation For Statistical Computing, Vienna, Austria, **2023**.
[13] E. Neuwirth, *RColorBrewer: ColorBrewer Palettes*, **2022**.
[14] H. Wickham, J. Hester, J. Bryan, *readr: Read Rectangular Text Data*, **2024**.
[15] H. Wickham, T. L. Pedersen, D. Seidel, *scales: Scale Functions for Visualization*, **2023**.
[16] Shuangbin Xu, M. Chen, T. Feng, L. Zhan, L. Zhou, G. Yu, "Use ggbreak to effectively utilize plotting space to deal with large datasets and outliers." *Front. Genet.* **2021**, *12*, 774846.
[17] H. Wickham, M. Averick, J. Bryan, W. Chang, L. D. McGowan, R. François, G. Grolemund, A. Hayes, L. Henry, J. Hester, M. Kuhn, T. L. Pedersen, E. Miller, S. M. Bache, K. Müller, J. Ooms, D. Robinson, D. P. Seidel, V. Spinu, K. Takahashi, D. Vaughan, C. Wilke, K. Woo, H. Yutani, "Welcome to the tidyverse" *J. Open Source Softw.* **2019**, *4*, 1686.
[18] A. Garre, J. Koomen, H. M. W. Den Besten, M. H. Zwietering, "Modeling Population Growth in R with the biogrowth Package" *J. Stat. Softw.* **2023**, *107*, DOI 10.18637/jss.v107.i01.
[19] Posit team, *RStudio: Integrated Development Environment for R*, Posit Software, PBC, Boston, MA, **2024**.
[20] C. A. Schneider, W. S. Rasband, K. W. Eliceiri, "NIH Image to ImageJ: 25 years of image analysis" *Nat. Methods* **2012**, *9*, 671–675.
[21] J. Schindelin, I. Arganda-Carreras, E. Frise, V. Kaynig, M. Longair, T. Pietzsch, S. Preibisch, C. Rueden, S. Saalfeld, B. Schmid, J.-Y. Tinevez, D. J. White, V. Hartenstein, K. Eliceiri, P. Tomancak, A. Cardona, "Fiji: an open-source platform for biological-image analysis" *Nat. Methods* **2012**, *9*, 676–682.
[22] F. W. Studier, B. A. Moffatt, "Use of bacteriophage T7 RNA polymerase to direct selective high-level expression of cloned genes" *J. Mol. Biol.* **1986**, *189*, 113–130.
[23] L. Gericke, D. Mhaindarkar, L. C. Karst, S. Jahn, M. Kuge, M. K. F. Mohr, J. Gagsteiger, N. V. Cornelissen, X. Wen, S. Mordhorst, H. J. Jessen, A. Rentmeister, F. P. Seebeck, G. Layer, C. Loenarz, J. N. Andexer, "Biomimetic S-Adenosylmethionine Regeneration Starting from Multiple Byproducts Enables Biocatalytic Alkylation with Radical SAM Enzymes" *ChemBioChem* **2023**, *24*, e202300133.
[24] M. M. Bradford, "A rapid and sensitive method for the quantitation of microgram quantities of protein utilizing the principle of protein-dye binding" *Anal. Biochem.* **1976**, *72*, 248–254.
[25] A. Marbach, K. Bettenbrock, "lac operon induction in Escherichia coli: Systematic comparison of IPTG and TMG induction and influence of the transacetylase LacA" *J. Biotechnol.* **2012**, *157*, 82–88.
[26] U. K. Laemmli, "Cleavage of structural proteins during the assembly of the head of bacteriophage T4" *Nature* **1970**, *227*, 680–685.
[27] H. T. S. Britton, R. A. Robinson, "LXI.—The use of the antimony–antimonous oxide electrode in the determination of the concentration of hydrogen ions and in potenliometric titrations. The Prideaux–Ward universal buffer mixture" *J. Chem. Soc. Resumed* **1931**, 458–473.
[28] S. Singh, J. Zhang, T. D. Huber, M. Sunkara, K. Hurley, R. D. Goff, G. Wang, W. Zhang, C. Liu, J. Rohr, S. G. Van Lanen, A. J. Morris, J. S. Thorson, "Facile Chemoenzymatic Strategies for the Synthesis and Utilization of S-Adenosyl-L-Methionine Analogues" *Angew. Chem. Int. Ed.* **2014**, *53*, 3965–3969.
[29] J. L. L. Rakels, A. J. J. Straathof, J. J. Heijnen, "A simple method to determine the enantiomeric ratio in enantioselective biocatalysis" *Enzyme Microb. Technol.* **1993**, *15*, 1051–1056.
[30] J. Pietruszka, A. C. M. Rieche, T. Wilhelm, A. Witt, "Kinetic Enzymatic Resolution of Cyclopropane Derivatives" *Adv. Synth. Catal.* **2003**, *345*, 1273–1286.
[31] C. J. Sih, S.-H. Wu in *Top. Stereochem.*, John Wiley & Sons, Ltd, **1989**, pp. 63–125.
[32] C. S. Chen, Y. Fujimoto, G. Girdaukas, C. J. Sih, "Quantitative analyses of biochemical kinetic resolutions of enantiomers" *J. Am. Chem. Soc.* **1982**, *104*, 7294–7299.
[33] P. Pracht, F. Bohle, S. Grimme, "Automated exploration of the low-energy chemical space with fast quantum chemical methods" *Phys. Chem. Chem. Phys.* **2020**, *22*, 7169–7192.
[34] S. Grimme, F. Bohle, A. Hansen, P. Pracht, S. Spicher, M. Stahn, "Efficient Quantum Chemical Calculation of Structure Ensembles and Free Energies for Nonrigid Molecules" *J. Phys. Chem. A* **2021**, *125*, 4039–4054.
[35] D. Bykov, T. Petrenko, R. Izsák, S. Kossmann, U. Becker, E. Valeev, F. Neese, "Efficient implementation of the analytic second derivatives of Hartree–Fock and hybrid DFT energies: a detailed analysis of different approximations" *Mol. Phys.* **2015**, *113*, 1961–1977.
[36] N. Mardirossian, M. Head-Gordon, "ωB97M-V: A combinatorially optimized, range-separated hybrid, meta-GGA density functional with VV10 nonlocal correlation" *J. Chem. Phys.* **2016**, *144*, 214110.
[37] F. Weigend, R. Ahlrichs, "Balanced basis sets of split valence, triple zeta valence and quadruple zeta valence quality for H to Rn: Design and assessment of accuracy" *Phys. Chem. Chem. Phys.* **2005**, *7*, 3297–3305.
[38] V. Barone, M. Cossi, "Quantum Calculation of Molecular Energies and Energy Gradients in Solution by a Conductor Solvent Model" *J. Phys. Chem. A* **1998**, *102*, 1995–2001.
[39] S. Hirata, M. Head-Gordon, "Time-dependent density functional theory within the Tamm–Dancoff approximation" *Chem. Phys. Lett.* **1999**, *314*, 291–299.
[40] T. Yanai, D. P. Tew, N. C. Handy, "A new hybrid exchange–correlation functional using the Coulomb-attenuating method (CAM-B3LYP)" *Chem. Phys. Lett.* **2004**, *393*, 51–57.

[41] E. Gasteiger, C. Hoogland, A. Gattiker, S. Duvaud, M. R. Wilkins, R. D. Appel, A. Bairoch in *Proteomics Protoc. Handb.* (Ed.: J.M. Walker), Humana Press, Totowa, NJ, **2005**, pp. 571–607.
[42] A. J. J. Straathof, J. A. Jongejan, “The enantiomeric ratio: origin, determination and prediction” *Enzyme Microb. Technol.* **1997**, *21*, 559–571.
[43] C. Dalhoff, G. Lukinavičius, S. Klimašauskas, E. Weinhold, “Direct transfer of extended groups from synthetic cofactors by DNA methyltransferases” *Nat. Chem. Biol.* **2006**, *2*, 31–32.
[44] B. J. C. Law, A.-W. Struck, M. R. Bennett, B. Wilkinson, J. Micklefield, “Site-specific bioalkylation of rapamycin by the RapM 16-O-methyltransferase” *Chem. Sci.* **2015**, *6*, 2885–2892.
[45] C. Zhang, R. L. Weller, J. S. Thorson, S. R. Rajski, “Natural Product Diversification Using a Non-natural Cofactor Analogue of S-Adenosyl-l-methionine” *J. Am. Chem. Soc.* **2006**, *128*, 2760–2761.
[46] K. Hartstock, B. S. Nilges, A. Ovcharenko, N. V. Cornelissen, N. Püllen, A.-M. Lawrence-Dörner, S. A. Leidel, A. Rentmeister, “Enzymatic or In Vivo Installation of Propargyl Groups in Combination with Click Chemistry for the Enrichment and Detection of Methyltransferase Target Sites in RNA” *Angew. Chem. Int. Ed.* **2018**, *57*, 6342–6346.
[47] C. Sommer-Kamann, A. Fries, S. Mordhorst, J. N. Andexer, M. Müller, “Asymmetric C-Alkylation by the S-Adenosylmethionine-Dependent Methyltransferase SgvM” *Angew. Chem. Int. Ed.* **2017**, *56*, 4033–4036.
[48] Q. Tang, C. W. Grathwol, A. S. Aslan-Üzel, S. Wu, A. Link, I. V. Pavlidis, C. P. S. Badenhorst, U. T. Bornscheuer, “Directed Evolution of a Halide Methyltransferase Enables Biocatalytic Synthesis of Diverse SAM Analogs” *Angew. Chem. Int. Ed.* **2021**, *60*, 1524–1527.
[49] H. Stecher, M. Tengg, B. J. Ueberbacher, P. Remler, H. Schwab, H. Griengl, M. Gruber-Khadjawi, “Biocatalytic Friedel-Crafts Alkylation Using Non-natural Cofactors” *Angew. Chem. Int. Ed.* **2009**, *48*, 9546–9548.
[50] J. Schlesier, J. Siegrist, S. Gerhardt, A. Erb, S. Blaesi, M. Richter, O. Einsle, J. N. Andexer, “Structural and functional characterisation of the methionine adenosyltransferase from Thermococcus kodakarensis” *BMC Struct. Biol.* **2013**, *13*, 22.
[51] T. D. Huber, J. A. Clinger, Y. Liu, W. Xu, M. D. Miller, G. N. Phillips, J. S. Thorson, “Methionine Adenosyltransferase Engineering to Enable Bioorthogonal Platforms for AdoMet-Utilizing Enzymes” *ACS Chem. Biol.* **2020**, *15*, 695–705.
[52] M. Erguven, D. N. V. Cornelissen, A. Peters, A. P. D. E. Karaca, P. D. A. Rentmeister, “Enzymatic Generation of Double-Modified AdoMet Analogues and Their Application in Cascade Reactions with Different Methyltransferases” *ChemBioChem* **2022**, *23*, e202200511.
[53] L. L. Bengel, B. Aberle, A. Egler-Kemmerer, S. Kienzle, B. Hauer, S. C. Hammer, “Engineered Enzymes Enable Selective N-Alkylation of Pyrazoles with Simple Haloalkanes” *Angew. Chem. Int. Ed.* **2021**, *60*, 5554–5560.
[54] K. H. Schülke, J. S. Fröse, A. Klein, M. Garcia-Borràs, S. C. Hammer, “Efficient Transferase Engineering for SAM Analog Synthesis from Iodoalkanes” *ChemBioChem* **2024**, *25*, e202400079.
[55] C.-Y. Gao, G.-Y. Yang, X.-W. Ding, J.-H. Xu, X. Cheng, G.-W. Zheng, Q. Chen, “Engineering of Halide Methyltransferase BxHMT through Dynamic Cross-Correlation Network Analysis” *Angew. Chem. Int. Ed.* **2024**, *63*, e202401235.
[56] G.-Y. Yang, G.-W. Zheng, B.-B. Zeng, J.-H. Xu, Q. Chen, “Engineering of halide methyltransferases for synthesis of SAE and its application in biosynthesis of ethyl vanillin” *Mol. Catal.* **2023**, *550*, 113533.
[57] K. H. Schülke, F. Ospina, K. Hörnschemeyer, S. Gergel, S. C. Hammer, “Substrate Profiling of Anion Methyltransferases for Promiscuous Synthesis of S-Adenosylmethionine Analogs from Haloalkanes” *ChemBioChem* **2022**, *23*, e202100632.
[58] A. Hoffmann, K. H. Schülke, S. C. Hammer, A. Rentmeister, N. V. Cornelissen, “Comparative S-adenosyl-L-methionine analogue generation for selective biocatalytic Friedel-Crafts alkylation” *Chem. Commun.* **2023**, *59*, 5463–5466.
[59] M. K. F. Mohr, R. Saleem-Batcha, N. V. Cornelissen, J. N. Andexer, “Enzymatic Synthesis of l-Methionine Analogues and Application in a Methyltransferase Catalysed Alkylation Cascade” *Chem Eur J* **2023**, *29*, e202301503.
[60] S. Mordhorst, J. Siegrist, M. Müller, M. Richter, J. N. Andexer, “Catalytic Alkylation Using a Cyclic S-Adenosylmethionine Regeneration System” *Angew. Chem. Int. Ed.* **2017**, *56*, 4037–4041.
[61] S. Ju, K. P. Kuzelka, R. Guo, B. Krohn-Hansen, J. Wu, S. K. Nair, Y. Yang, “A biocatalytic platform for asymmetric alkylation of α-keto acids by mining and engineering of methyltransferases” *Nat. Commun.* **2023**, *14*, 5704.
[62] D. A. Amariei, N. Pozhydaieva, B. David, P. Schneider, T. Classen, H. Gohlke, O. H. Weiergräber, J. Pietruszka, “Enzymatic C3-Methylation of Indoles Using Methyltransferase PsmD─Crystal Structure, Catalytic Mechanism, and Preparative Applications” *ACS Catal.* **2022**, *12*, 14130–14139.
[63] M. T. Salinger, D. Castellano Garrido, E. D. Lamming, J. M. Ward, T. S. Moody, J. W. E. Jeffries, H. C. Hailes, “An Alternative Cascade for the Selective Methylation of Catechols and Tetrahydroisoquinolines by O-Methyltransferases” *ChemCatChem* **2024**, *16*, e202400492.
[64], “Price listing for methyl iodide (ID: 67692) from Sigma-Aldrich,” can be found under https://www.sigmaaldrich.com/DE/de/product/sial/67692(accessed 7 May 2021), **2025**.
[65], “Price listing for SAH (ID: BD103799) from Biosynth,” can be found under https://www.bldpharm.com/products/979-92-0.html(accessed 7 May 2021), **2025**.
[66], “Price listing for L-Met (ID: BD41905) from Biosynth,” can be found under https://www.bldpharm.com/products/63-68-3.html(accessed 7 May 2021), **2025**.
[67], “Price listing for ATP disodium salt (ID: BD112724) from BLDpharm,” can be found under https://www.bldpharm.com/products/987-65-5.html(accessed 7 May 2021), **2025**.
[68], “Price listing for SAM toluene sulfonate (ID: NA08266) from Biosynth,” can be found under https://www.biosynth.com/p/NA08266/97540-22-2-s-adenosyl-l-methionine-disulfate-tos(accessed 7 May 2021), **2025**.
[69] J.-C. Yi, C. Liu, L.-X. Dai, S.-L. You, “Synthesis of C3-Methyl-Substituted Pyrroloindolines and Furoindolines via Cascade Dearomatization of Indole Derivatives with Methyl Iodide” *Chem. Asian J.* **2017**, *12*, 2975–2979.
